# Catalytic Access to 4-(sec-Alkyl)Anilines via 1,6-Conjugate Addition of Grignard Reagents to *in Situ* Generated aza-*p*-Quinone Methides

**DOI:** 10.1021/acs.orglett.2c02786

**Published:** 2022-09-02

**Authors:** Mercedes Zurro, Luo Ge, Syuzanna R. Harutyunyan

**Affiliations:** † Stratingh Institute for Chemistry University of Groningen Institution Nijenborgh 4, 9747 AG, Groningen, The Netherlands

## Abstract

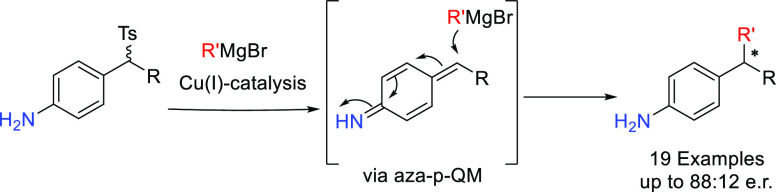

The synthesis of aniline derivatives, common building blocks in many pharmaceuticals, agrochemicals, dyes or polymers, has been limited to reactions based on benzene-toluene-xylene derivatives (BTX) due to their ample availability. Despite the large number of existing methodologies, the synthesis of chiral 4-(sec-alkyl)anilines has not been accomplished so far. In this work, a tandem strategy based on the generation of a reactive aza-*p*-quinone methide (aza-*p*-QM) intermediate followed by Cu(I)-catalyzed addition of Grignard reagents has been developed.

Anilines are common building blocks present in many pharmaceuticals, agrochemicals, dyes, or polymers.^1^ Most existing methods for the synthesis of simple anilines make use of readily available BTX derivatives or other simple benzene compounds, which through different classic strategies, such as nitration–reduction pathways or aromatic substitution, yield simple aniline derivatives. Unfortunately, these methodologies are less suitable for the synthesis of more complex aniline derivatives.^2^ For that purpose, CH-activation methodologies based on palladium- or copper-catalyzed cross-coupling reactions have been developed, as they allow the introduction of amino groups in specific positions of functionalized aromatics.^3^ Very recently, a photochemical dehydrogenative methodology consisting of a coupling between amines and cyclohexanones has been reported for the synthesis of anilines.^4^ A wide variety of *o*- and *p*-substituted anilines could be synthesized using this robust methodology. However, despite all these existing methodologies for the synthesis of anilines, a suitable procedure for the synthesis of *sec*-alkyl anilines has not been reported yet.

In this context, nucleophilic additions to aza-*ortho*- and aza-*para*-quinone methides (aza-*o*-QMs and aza-*p*-QMs) offer an interesting path to access aniline derivatives. The reactivity of aza-*o*-QMs has been studied in depth and a wide variety of starting materials serve as precursors for their synthesis,^5^ while the study of aza-*p*-QM is underdeveloped.^6^ Although aza-*p*-QMs have been used efficiently in material^7^ and medicinal chemistry,^8^ the development of catalytic methodologies for their derivatization has been limited, probably due to the instability of these reactive intermediates. As a result, applications of aza-*p*-QMs have been restricted to their use as linkers in self-immolative polymers^7^ or prodrugs,^8^ and not as substrates for the synthesis of useful products. Conjugate additions to aza-*p*-quinone methides are mechanistically similar to additions to *p*-quinone methides (*p*-QM). The latter are typically limited to *p*-QM that feature *tert*-butyl substituents at the 2- and 6-positions (Scheme 1A), since they are stable and do not require generation *in situ* but rather posterior modification in order to remove the *tert*-butyl groups.^9^

**Scheme 1:**
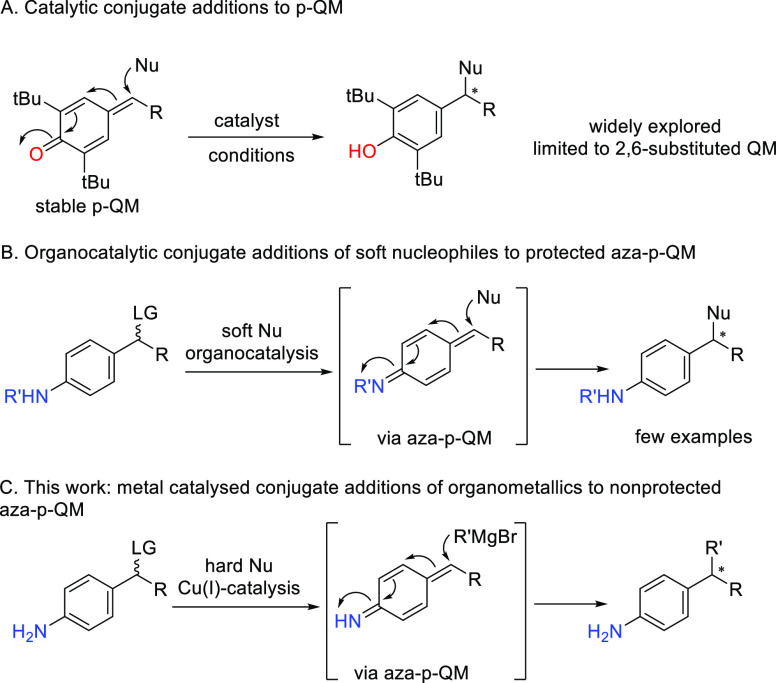
(A) Catalytic Conjugate Additions to *p*-QM; (B) Organocatalytic Conjugate Additions of Soft Nucleophiles to in Situ Generated *N*-Protected aza-*p*-QM; (C) This Work: Copper Catalyzed Conjugate Addition to in Situ Generated aza-*p*-QM

However, *in situ* generation of *p*-QM from the corresponding phenol has also been also reported, in which case enantioselective reactions require substituents at the 2- and 6- positions.^10^

To the best of our knowledge, only organocatalytic methodologies have been reported for additions to aza-*p*-QM: a 1,6-addition for the N-alkylation of indoles using a chiral phosphoric acid as an organocatalyst (Scheme 1B),^11^ and two examples of 1,8-additions to *in situ* generated aza-*p*-QM for the construction of anilines with an appended allene group.^12^ However, no enantioselective organometallic catalytic approach has been described so far for the synthesis of enantioenriched anilines from aza-*p*-QM.

Over the past years, our research group has developed a number of metal-catalyzed methodologies for additions of Grignard reagents to imines, Michael acceptors, and, very recently, indole-derived vinylogous imines.^13,14^ To take this one step further, we decided to study 1,6-conjugate addition of Grignard reagents to *in situ* generated unstable and reactive aza-*p*-QM intermediates in order to access aniline derivatives (Scheme 1C). We envisioned that, when using 2 equiv of Grignard reagent, the first equivalent would serve as a base for the *in situ* formation of aza-*p*-QM, while the second equivalent would enable the 1,6-addition of the Grignard reagent. This process should be amenable to copper catalysis, since copper(I)-salts are known to promote conjugate addition reactions to activated, electrophilic double bonds. Furthermore, choosing an appropriate chiral ligand to bind the copper and the leaving group at the precursor of aza-*p*-QM should enable enantioselective catalytic synthesis, thus yielding enantioenriched chiral aniline derivatives.

We started our studies by selecting sulfone **1a** as the model substrate to generate the corresponding aza-*p*-QM precursor (Table 1). While the majority of reports makes use of amino alcohols as substrate to generate QM,^10,11^ we found that in our case these compounds are quite unstable if the N atom is not protected, and therefore not suitable for catalysis.

**Table 1:**
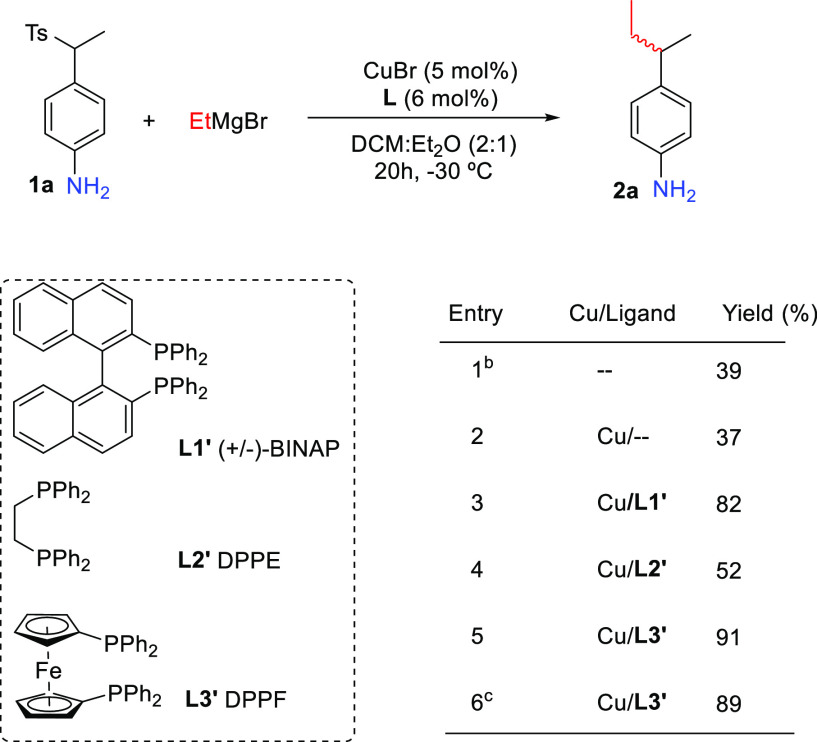
Influence of Catalyst Structure on the Reaction Outcome^a^

a General conditions: **1a** (0.2 mmol), CuBr (5 mol %), **L** (6 mol %), EtMgBr (3 equiv), DCM/Et_2_O = 2:1 (3.0 mL), −30 °C.

b Without CuBr.

c Reaction time 3.5 h

Instead, sulfone **1a** is readily accessible (in two steps) from commercially available chemicals and can be stored for long periods. Furthermore, it was also shown that the Ts group is a viable leaving group for such transformations.^14,15^ EtMgBr was chosen as the nucleophile, and the initial studies were carried out at −30 °C using DCM/Et_2_O (2:1). The background reaction (with and without copper salt) afforded the desired aniline derivative **2a** in moderate yield (Table 1, entries 1 and 2). However, when we combined the copper salt with a bidentate phosphine ligand, a significant increase in the product yield was obtained (Table 1, entries 3–6). Among various ligands screened, DPPF (**L3′**) afforded the best yield of 91% (Table 1, entry 5). Additionally, the reaction time could be decreased from 20 to 3.5 h without a major drop in the yield of **2a** (Table 1, entry 6). Nevertheless, all remaining experiments were performed by stirring reaction mixtures for 20 h.

With the optimal reaction conditions in hands, the Grignard scope was explored next (Scheme 2). The use of MeMgBr afforded the symmetric aniline **2b** in a good yield (71%), while different linear alkyl chains afforded the corresponding 4-substituted anilines in good to excellent yields (**2c**–**2e**). Similarly, a cyclic Grignard reagent led to the aniline **2f** in a quantitative yield. The addition of aryl Grignard reagent afforded the anilines **2g**–**2i** in good yields as well. Finally, a vinyl, benzyl, phenethyl, and allyl Grignard reagent were tested as well, all providing the corresponding aniline derivatives (**2j**–**2m**) in good yields.

**Scheme 2:**
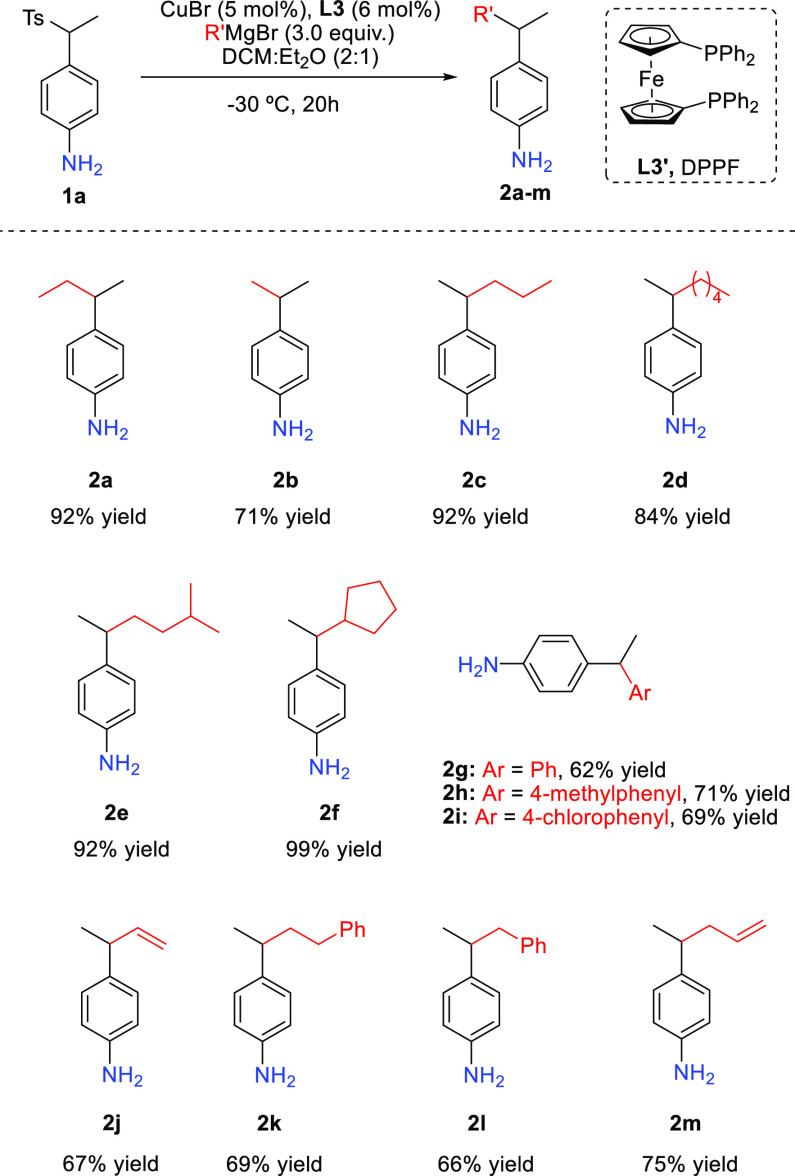
Grignard Scope Racemic Transformation General conditions: **1a** (0.2 mmol), CuBr (5 mol %), **L3′** (6 mol %), R′MgBr (3 equiv), DCM/Et_2_O = 2:1 (3.0 mL), −30 °C, 20 h. Yields of isolated products are given experiments were performed by stirring reaction mixtures for 20 h.

Having established the racemic synthesis of aniline derivatives, we then moved to the development of the asymmetric catalytic version of this reaction. For this purpose, we performed optimization studies by varying the chiral ligand and the temperature (Table 2). To slow down the rate of the uncatalyzed reaction, we carried out the enantioselective reactions at a lower temperature, namely at −50 °C and using DCM as a solvent. However, even at this temperature racemic **2a** is formed (Table 2, entries 1 and 2). Various chiral diphosphine and phosphoramidite ligands were screened in combination with CuBr·SMe_2_. Unfortunately, most of the chiral ligands tested, **L1**–**L6**, resulted in products that were either racemic or had low enantiomeric ratio, although in some cases the reaction was significantly accelerated providing the product in good isolated yield (Table 2, entry 3, see SI).

**Table 2:**
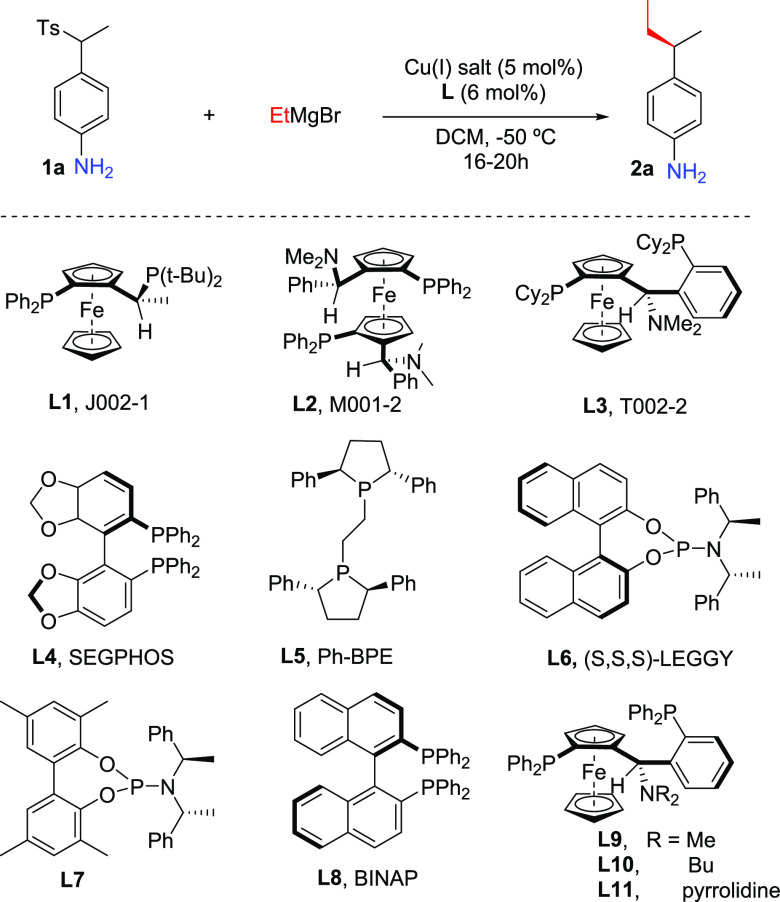
Optimization Studies for Catalytic Asymmetric Version^a^

entry	L	Cu salt	yield^b^ (%)	e.r. (**2a**)^c^
1	–	–	59	50:50
2	–	CuBr·SMe_2_	52	50:50
3	**L1–L6**	CuBr·SMe_2_	59–99	50:50
4	**L7**	CuBr·SMe_2_	99	30:70
5	**L8**	CuBr·SMe_2_	99	30:70
6	**L9**	CuBr·SMe_2_	57	85:15
7^d^	**L10**	CuBr·SMe_2_	60	15:85
8^e^	**L11**	CuBr·SMe_2_	63	15:85
9^f^	**L9**	CuBr·SMe_2_	60	85:15
10^g^	**L9**	CuBr·SMe_2_	48	50:50
11	**L9**	CuCl	30	86:14
12	**L9**	CuBr	69	87:13
13^h^	**L9**	CuBr	78	87:13

a Reaction conditions: Cu(I) salt (5.0 mol %), **L** (6 mol %) in dry solvent (2 mL), then substrate **1a** (0.2 mmol, 1.0 equiv) in 1 mL of solvent, EtMgBr (3.0 equiv. 3.0 M solution in Et_2_O), reaction time (16–20 h).

b Isolated yield.

c The enantiomeric ratio was determined by analytical chiral HPLC.

d (*R,R*)-**L10** was used in this case.

e (*R,R*)-**L11** was used in this case.

f At −70 °C.

g At rt.

h 2:1 DCM/Et_2_O mixture of solvents was used in this case.

On the other hand, using chiral phosphoramidite ligand **L7** resulted in product **2a** with an enantiomeric ratio of 30:70 and a quantitative yield (Table 2, entry 4). Similar results were obtained using BINAP ligand **L8** instead (Table 2, entry 5), while a further increase in enantioselectivity was observed with Taniaphos ligand **L9** (Table 2, 85:15 e.r., entry 6). On the contrary, combining copper salt with other Taniaphos ligands, **L10** and **L11**, did not improve the enantiomeric ratio of the final product (Table 2, entries 7 and 8). Having established the best performing ligand for this transformation, we explored the effects of other variables, such as the copper(I) source, solvent, and temperature (Table 2, entries 9–14) on the reaction outcome. While no improvement in the enantioselectivity and product yield was observed at −70 °C, racemic product was obtained at room temperature in parallel with a decrease in the yield of **2a** (Table 2, entries 9 and 10). Replacing CuBr·SMe_2_ by CuCl (Table 2, entry 11) resulted in a lower yield, while using CuBr instead (Table 2, entry 12) led to a slightly improved yield and enantioselectivity (87:13 e.r., 69% yield). Interestingly, we found that using a 2:1 DCM/Et_2_O mixture of solvents was beneficial for the reaction, allowing product **2a** to be obtained with a 78% isolated yield (Table 2, entry 13).

On the other hand, no product was obtained when using Et_2_O instead of DCM as the solvent of the reaction.

Finally, we investigated the effect of the sulfonyl leaving group on the outcome of the reaction catalyzed by the Cu(I)/**L9** catalytic system (Scheme 3). As expected, the nature of the leaving sulfonyl group had a minimal impact on the reaction outcome, leading to a slight decrease in enantioselectivity when using a less or more bulky sulfonyl group than tosyl.

**Scheme 3:**
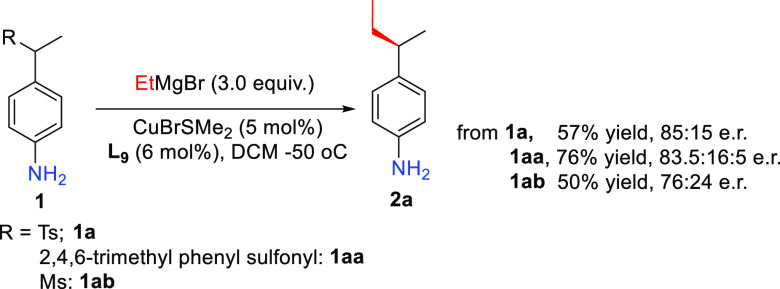
Effect of the Nature of Sulfonyl Leaving Group on the Reaction Outcome

Having optimized the conditions for the enantioselective reaction (CuBr 5 mol %, Taniaphos **L9** (6 mol %), in DCM/Et_2_O (2:1) for the substrate with the tosyl leaving group, we explored the substrate scope of this transformation (Scheme 4). To this end, different aniline sulfone derivatives were synthesized. First, we studied the effect of a longer alkyl substituent on the reaction outcome. Sulfones with either a pentyl or a propyl substituent afforded the chiral anilines **2n** and **2o**, respectively, in good yields and nearly the same e.r. Similarly, isobutyl substituted sulfone led to the aniline **2q** in high yield (91%) albeit with slightly lower enantioselectivity (79:21 e.r.).

**Scheme 4:**
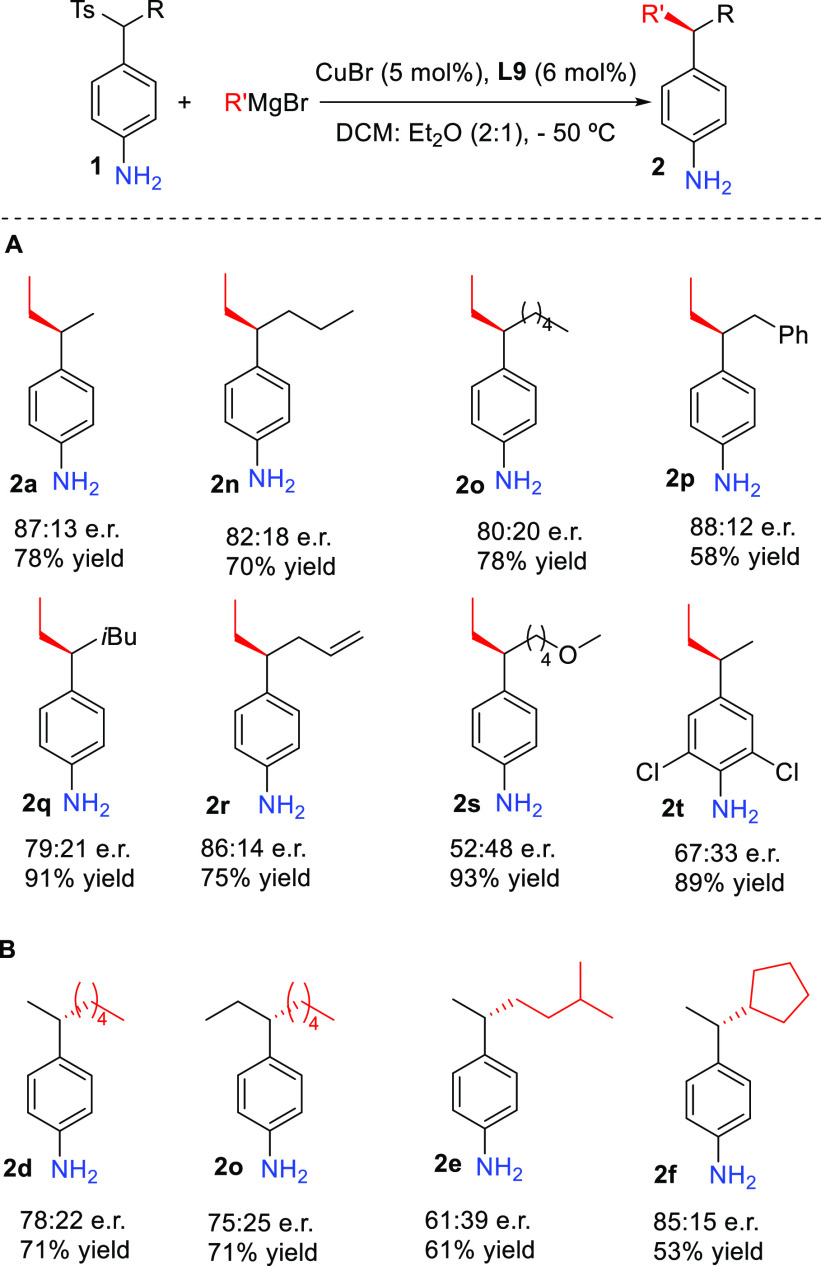
Substrate (A) and Grignard (B) Scopes for the Enantioselective Transformation General conditions: **1** (0.2 mmol), CuBr (5 mol %), **L9** (6 mol %), R′MgBr (3 equiv), DCM/Et_2_O = 2:1 (3.0 mL), −50 °C, 20 h. Yields of isolated products are given experiments were performed by stirring reaction mixtures for 20 h.

An allyl or benzyl substituent on the sulfone was also well tolerated, resulting in the corresponding aniline products **2p** and **2r**. On the other hand, methoxy-substituted sulfone resulted in racemic product **2s**. Finally, the influence of substitution at the aniline ring was studied. A 2,6- chloro-substituted sulfone derivative was synthesized and tested in the reaction to afford the corresponding product **2t** with 67:33 enantiomeric ratio. The low enantioselectivity observed in this case might be indicative of the substituent interfering with the possible binding of the copper catalyst to the aromatic ring of aza-*p*-QM, forming a σ-complex. Consequently, the presence of substituents in the aromatic ring of the original sulfone might be disruptive for the formation of the intermediate complex and therefore lead to a drop in the enantioselectivity of the reaction. Next, the scope of the Grignard reagent was studied. First, we found that Grignard reagents with a longer alkyl chain (Scheme 4) afford the corresponding sec-alkyl anilines **2d** and **2o** in moderate to good yields and moderate enantioselectivities. Similar results were obtained when using isopentyl Grignard reagent (Scheme 4, **2e**). A cyclopentyl substituent was installed, affording **2f** with higher enantioselectivity but moderate yield.

The catalytic reaction between **1a** and EtMgBr was scaled up to 1–1.5 mmol (Scheme 5), which hardly affected the overall outcome of the reaction in terms of yield and selectivity (85%–79% yield, 87:13 e.r.). Finally, the chiral aniline derivative **2a** was derivatized to the sulfonamide **3**, an X-ray analysis of which allowed us to determine the absolute configuration of the chiral sulfonamide.

**Scheme 5:**
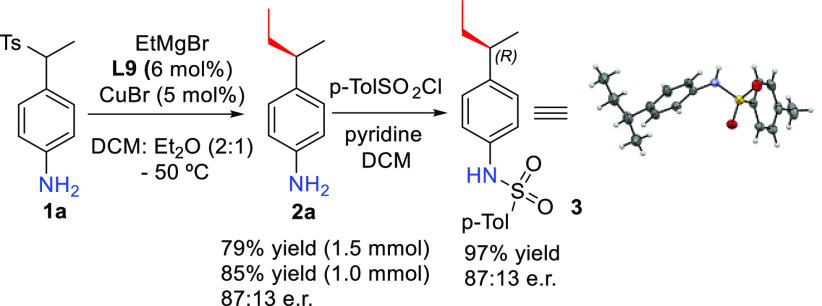
Derivatization and X-ray Analysis

In this work, the first example of metal catalyzed addition of organometallic reagents to aza-*p-*QM has been established. In particular, we have demonstrated that 1,6-addition of Grignard reagents to *in situ* generated aza-*p-*QM proceeds efficiently to afford various aniline derivatives under copper catalysis. While the use of achiral DPPF ligand allowed us to obtain a wide variety of 4-sec-(alkyl) aniline derivatives in good to excellent yields, the enantioselective version, based on a catalytic system using chiral ferrocenyl diphosphine ligand Taniaphos **L9** and copper(I) salt, affords the chiral aniline derivatives with an e.r. up to 88:12. The enantioselective transformation is scalable, and the resulting products can be easily derivatized into chiral sulfonamide **3**.

## Supporting Information Available

Experimental procedures and characterization data, Xray structure determination, and NMR spectra (PDF)
