# Supplementary material for: Catalytic Access to 4-(sec-Alkyl)Anilines via 1,6-Conjugate Addition of Grignard Reagents to in Situ Generated aza-p-Quinone Methides
Source: Org Lett. 2022 Sep 2;24(36):6686–91. doi: 10.1021/acs.orglett.2c02786 (PMC9486948; doi:10.1021/acs.orglett.2c02786)

Catalytic access to 4-(sec-alkyl)anilines via 1,6-conjugate addition of Grignard reagents to in situ generated aza-p-quinone methides

Mercedes Zurro [a], Luo Ge [a] and Syuzanna R. Harutyunyan [a]*

[a] Stratingh Institute for Chemistry, University of Groningen, Nijenborgh 4, 9747 AG, Groningen, The Netherlands.

Content

1. General Experimental Information
2. General Chemicals
3. Catalytic reactions for the synthesis of racemic 4-(sec alkyl)anilines
4. Enantioselective Catalytic reaction for the Synthesis of chiral anilines
4.1. Screening of ligands
4.2. Synthesis of chiral 4-(sec alkyl)anilines
5. Derivatizations
6. Synthesis of the substrates
6.1 *Method A*
6.1.1. Synthesis of an alcohol derivative
6.1.2. Synthesis of an amino sulfone derivative
6.2. *Method B*
6.2.1. Synthesis of alcohols
6.2.2. Synthesis of sulfones
7. X-ray crystallography
8. NMR spectra

1. General Experimental Information

All reactions using oxygen- and/or moisture-sensitive materials were carried out with anhydrous solvents under a nitrogen atmosphere using standard Schlenk techniques. Flash column chromatography was performed using Merck 60 Å 230-400 mesh silica gel. Thin layer chromatography was performed using 0.25 mm E. Merck silica plates (60F-254). Unless otherwise indicated, the anilines were visualized using UV lamp, and other common staining solutions as phosphomolibdic acid and $KMnO_4$ staining. NMR data was collected on Varian VXR400 (^1H at 400.0 MHz; ^{13}C at 100.58 MHz) equipped with a 5 mm z-gradient broadband probe. Chemical shifts are reported in parts per million (ppm) relative to residual solvent peak ($CDCl_3$, ^1H: 7.26 ppm; ^{13}C: 77.16 ppm). Coupling constants are reported in Hertz. Multiplicity is reported with the usual abbreviations (s: singlet, d: doublet, dd: doublet of doublets, t: triplet, dt: doublet of triplets, q: quartet, dq: doublet of quartets, m: multiplet). Exact mass spectra were recorded on a LTQ Orbitrap XL apparatus with ESI ionization. Enantiomeric excess (*ee*) were determined by chiral HPLC analysis using a Shimadzu LC-10ADVP HPLC equipped with a Shimadzu SPD-M10AVP diode array detector.

2. General Chemicals

Unless otherwise indicated, reagents and substrates were purchased from commercial sources and used as received. Solvents not required to be dry were purchased as technical grade and used as received. Other dry solvents were freshly collected from a dry solvent purification system prior to use. Inert atmosphere experiments were performed with standard Schlenk techniques with dried (P_2O_5) nitrogen gas. Grignard reagents were purchased from Sigma-Aldrich unless noted: EtMgBr, MeMgBr (3.0 M in Et_2O); *i*BuMgBr, *i*PentMgBr, *n*HexMgBr, cyclopentylMgBr (2.0 M in Et_2O). Chiral ligands (**L1-L6**, **L8**, **L9**, **L12-L28**, **L30**, **L32**, **L33**) were purchased from Sigma Aldrich and Solvias. All reported compounds were characterized by ^1H and ^{13}C NMR and compared with literature data. All new compounds were fully characterized by ^1H and ^{13}C NMR and HRMS techniques. Chiral ligands (**L7**, **L29**, **L31**) [1] and (**L10**, **L11**) [2] were synthesized following the literature procedures.

3. Catalytic reactions for the synthesis of racemic 4-(sec alkyl)anilines

General procedure

To a previously dried Schlenk flask under nitrogen, CuBr (5.0 mol%) and ligand DPPF (6.0 mol%) were added in dry Et_2O (1 mL) and DCM (1 mL). After stirring for 10 min, the solution was cooled down to -30 ºC, and the substrate (1.0 equiv.) was added in 1 mL of DCM, followed by the addition of Grignard reagent (3 equiv.). The reaction mixture was stirred for 20h. The reaction was quenched with NH_4Cl sat sol. (5 mL) and extracted with DCM (x3).

4-(sec-butyl)aniline (2a)

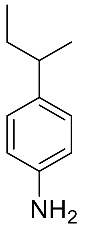

To a previously dried Schlenk flask under nitrogen, CuBr (1.4 mg, 0.010 mmol, 5.0 mol%) and ligand DPPF (6.7 mg, 0.012 mmol, 6.0 mol%) were added in dry Et_2O (1 mL) and DCM (1 mL). After stirring for 10 min, the solution was cooled down to -30 ºC, and the substrate (55.1 mg, 0.20 mmol, 1.0 equiv.) was added in 1 mL of DCM, followed by the addition of ethyl magnesium bromide (Aldrich, 3.0 M solution in Et_2O, (0.2 mL, 0.6 mmol, 3.0 equiv)). The reaction mixture was stirred for 20h. The reaction was quenched with NH_4Cl sat sol (5 mL) and extracted with DCM (x3). The crude was purified by flash column chromatography using DCM as eluent to afford the corresponding product **2a** as a colourless liquid (27.46 mg, 0.184 mmol, 92%). The spectroscopic data matched with those reported in the literature.[3]

^1H NMR (400 MHz, $CDCl_3$) δ 6.98 (d, J = 8.2 Hz, 2H), 6.65 (d, J = 8.1 Hz, 2H), 3.52 (br s, 2H), 2.53 – 2.44 (m, 1H), 1.57 – 1.50 (m, 2H), 1.19 (d, J = 7.0 Hz, 3H), 0.81 (t, J = 7.4 Hz, 3H).

^{13}C NMR (151 MHz, $CDCl_3$) δ 144.2, 138.1, 127.9, 115.4, 40.9, 31.5, 22.2, 12.4.

---

[1] (a) Duursma, A.; Boiteau, J.-G.; Lefort, L.; Boogers, J. A. F.; de Vries, A. H. M.; de Vries, J. G.; Minnaard, A. J.; Feringa, B. L. *J. Org. Chem.* **2004**, *69*, 8045–8052. (b) Alexakis, A.; Polet, D.; Rosset, S.; March, S. *J. Org. Chem.* **2004**, *69*, 5660–5667.
[2] Ireland T.; Tappe K.; Grossheimann G.; Knochel P. *Chem. Eur. J.* **2002**, *8*, 843-852.
[3] Joshi-Pangu, A.; Ganesh, M.; Biscoe, M. R. *Org. Lett.* **2011**, 13, 1218-1221.

HRMS (ESI-TOF): m/z $[M+H]^+$ Calcd. for $C_{10}H_{15}NH$ 150.1283, found 150.1276.

4-isopropylaniline (2b)

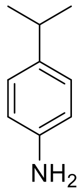

To a previously dried Schlenk flask under nitrogen, CuBr (1.4 mg, 0.01 mmol, 5.0 mol%) and ligand DPPF (6.7 mg, 0.012 mmol, 6.0 mol%) were added in dry Et_2O (1 mL) and DCM (1 mL). After stirring for 10 min, the solution was cooled down to -30ºC, and the substrate (55.1 mg, 0.2 mmol, 1.0 equiv.) was added in 1 mL of DCM, followed by the addition of methyl magnesium bromide (Aldrich, 3.0 M solution in Et_2O, (0.2 mL, 0.2 mmol, 3.0 equiv)). The reaction mixture was stirred for 20h. The reaction was quenched with NH_4Cl sat sol (5 mL) and extracted with DCM (x3). The crude was purified by flash column chromatography using DCM as eluent to afford the corresponding product **2b** as a colourless liquid (19.2 mg, 0.142 mmol, 71%). The spectroscopic data matched with those reported in the literature.[3]

^1H NMR (600 MHz, $CDCl_3$) δ 7.04 (d, J = 8.3 Hz, 2H), 6.65 (d, J = 8.4 Hz, 2H), 3.39 (br. s, 2H), 2.84 – 2.82 (m, 1H), 1.22 (d, J = 7.0 Hz, 6H).

^{13}C NMR (151 MHz, $CDCl_3$) δ 144.1, 139.3, 127.2, 115.4, 33.3, 24.4.

HRMS (ESI-TOF): m/z $[M+H]^+$ Calcd. for $C_9H_{13}NH$ 136.1121, found 136.1118.

4-(hexan-3-yl)aniline (2c)

To a previously dried Schlenk flask under nitrogen, CuBr (1.4 mg, 0.01 mmol, 5.0 mol%) and ligand DPPF (6.7 mg, 0.012 mmol, 6.0 mol%) were added in dry Et_2O (1 mL) and DCM (1 mL). After stirring for 10 min, the solution was cooled down to -30 ºC, and the substrate (55.1 mg, 0.2 mmol, 1.0 equiv.) was added in 1 mL of DCM, followed by the addition of propyl magnesium bromide (Aldrich, 2.0 M solution in Et_2O, (0.3 mL, 0.6 mmol, 3.0 equiv)). The reaction mixture was stirred for 20h. The reaction was quenched with NH_4Cl sat sol (5 mL) and extracted with DCM (x3). The crude was purified by flash column chromatography using DCM as eluent to afford the corresponding product **2c** as a colourless liquid (30.2 mg, 0.185 mmol, 92%).

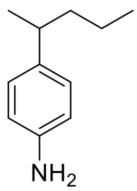

^1H NMR (600 MHz, $CDCl_3$) δ 7.12 – 6.82 (m, 2H), 6.72 – 6.54 (m, 2H), 3.27 (br s, 2H), 2.63 – 2.57 (m, 1H), 1.70 – 1.40 (m, 2H), 1.36 – 1.10 (m, 2H), 1.19 (d, J = 7.0 Hz, 3H), 0.87 (t, J = 7.4 Hz, 3H).

^{13}C NMR (151 MHz, $CDCl_3$) δ 144.1, 138.4, 127.8, 115.4, 41.0, 38.9, 22.6, 21.0, 14.3.

HRMS (ESI-TOF): m/z $[M+H]^+$ Calcd. for $C_{11}H_{17}NH$ 164.1439, found 164.1433.

4-(heptan-2-yl)aniline (2d)

To a previously dried Schlenk flask under nitrogen, CuBr (1.4 mg, 0.01 mmol, 5.0 mol%) and ligand DPPF (6.7 mg, 0.012 mmol, 6.0 mol%) were added in dry Et_2O (1 mL) and DCM (1 mL). After stirring for 10 min, the solution was cooled down to -30 ºC, and the substrate (55.1 mg, 0.2 mmol, 1.0 equiv.) was added in 1 mL of DCM, followed by the addition of pentyl magnesium bromide (Aldrich, 2.0 M solution in Et_2O, (0.3 mL, 0.6 mmol, 3.0 equiv)). The reaction mixture was stirred for 20h. The reaction was quenched with NH_4Cl sat sol (5 mL) and extracted with DCM (x3). The crude was purified by flash column chromatography using DCM as eluent to afford the corresponding product **2d** as a colourless liquid (32.0 mg, 0.167 mmol, 84%).

^1H NMR (600 MHz, $CDCl_3$) δ 6.98 (d, J = 8.3 Hz, 2H), 6.65 (d, J = 8.3 Hz, 2H), 3.38 (br s, 2H), 2.60 – 2.55 (m, 1H), 1.55 – 1.46 (m, 2H), 1.30 – 1.13 (m, 9H), 0.87 (t, J = 6.9 Hz, 3H).

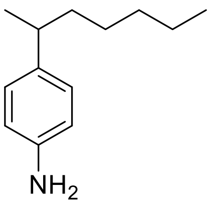

^{13}C NMR (151 MHz, $CDCl_3$) δ 144.1, 138.4, 127.8, 115.4, 39.2, 38.8, 32.0, 29.6, 27.8, 22.7, 14.2.

HRMS (ESI-TOF): m/z $[M+H]^+$ Calcd. for $C_{13}H_{21}NH$ 192.1752, found 192.1747

4-(5-methylhexan-2-yl)aniline (2e)

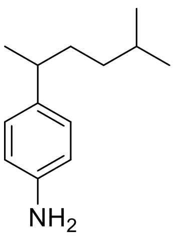

To a previously dried Schlenk flask under nitrogen, CuBr (1.4 mg, 0.01 mmol, 5.0 mol%) and ligand DPPF (6.7 mg, 0.012 mmol, 6.0 mol%) were added in dry Et_2O (1 mL) and DCM (1 mL). After stirring for 10 min, the solution was cooled down to -30 ºC, and the substrate (55.1 mg, 0.2 mmol, 1.0 equiv.) was added in 1 mL of DCM, followed by the addition of isopentyl magnesium bromide (Aldrich, 2.0 M solution in Et_2O, (0.3 mL, 0.6 mmol, 3.0 equiv)). The reaction mixture was stirred for 20h. The reaction was quenched with NH_4Cl sat sol (5 mL) and extracted with DCM (x3). The crude was purified by flash column chromatography using DCM as eluent to afford the corresponding product **2e** as a colourless liquid (35.1 mg, 0.183 mmol, 92%).

^{1}H NMR (600 MHz, $CDCl_3$) δ 6.98 (d, J = 8.3 Hz, 2H), 6.64 (d, J = 8.3 Hz, 2H), 3.29 (br. s, 2H), 2.59 – 2.50 (m, 1H), 1.56 – 1.37 (m, 3H), 1.19 (d, J = 6.9 Hz, 3H), 1.16 – 1.00 (m, 2H), 0.84 (d, J = 2.3 Hz, 3H), 0.82 (d, J = 2.3 Hz, 3H).

^{13}C NMR (151 MHz, $CDCl_3$) δ 144.0, 138.5, 127.8, 115.4, 39.5, 37.2, 36.5, 28.3, 22.8, 22.7.

HRMS (ESI-TOF): m/z $[M+H]^+$ Calcd. for $C_{13}H_{21}NH$ 192.1752, found 192.1742.

4-(1-cyclopentylethyl)aniline (2f)

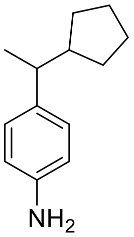

To a previously dried Schlenk flask under nitrogen, CuBr (1.4 mg, 0.01 mmol, 5.0 mol%) and ligand DPPF (6.7 mg, 0.012 mmol, 6.0 mol%) were added in dry Et_2O (1 mL) and DCM (1 mL). After stirring for 10 min, the solution was cooled down to -30 ºC, and the substrate (55.1 mg, 0.2 mmol, 1.0 equiv.) was added in 1 mL of DCM, followed by the addition of cyclopentyl magnesium bromide (Aldrich, 2.0 M solution in Et_2O, (0.3 mL, 0.6 mmol, 3.0 equiv)). The reaction mixture was stirred for 20h. The reaction was quenched with NH_4Cl sat sol (5 mL) and extracted with DCM (x3). The crude was purified by flash column chromatography using DCM as eluent to afford the corresponding product **2f** as colourless liquid (37.7 mg, 0.199 mmol, >99%).

^{1}H NMR (600 MHz, $CDCl_3$) δ 6.97 (d, J = 8.3 Hz, 2H), 6.64 (d, J = 8.3 Hz, 2H), 3.36 (br s, 2H), 2.33 (dq, J = 9.4, 6.9 Hz, 1H), 1.91 – 1.85 (m, 2H, CH_2), 1.65 – 1.39 (m, 5H), 1.22 – 1.17 (m, 4H), 1.06 – 0.91 (m, 1H).

^{13}C NMR (151 MHz, $CDCl_3$) δ 143.9, 138.7, 128.1, 115.4, 47.9, 45.4, 31.9, 31.5, 25.5, 25.2, 21.7.

HRMS (ESI-TOF): m/z $[M+H]^+$ Calcd. for $C_{13}H_{19}NH$ 190.1590, found 190.1591

4-(1-phenylethyl)aniline (2g)

To a previously dried Schlenk flask under nitrogen, CuBr (1.4 mg, 0.01 mmol, 5.0 mol%) and ligand DPPF (6.7 mg, 0.012 mmol, 6.0 mol%) were added in dry Et_2O (1 mL) and DCM (1 mL). After stirring for 10 min, the solution was cooled down to -30 ºC, and the substrate (55.1 mg, 0.2 mmol, 1.0 equiv.) was added in 1 mL of DCM, followed by the addition of phenyl magnesium bromide (Aldrich, 3.0 M solution in Et_2O, ((0.2 mL, 0.6 mmol, 3.0 equiv)). The reaction mixture was stirred for 20h. The reaction was quenched with NH_4Cl sat sol (5 mL) and extracted with DCM (x3). The crude was purified by flash column chromatography using DCM as eluent to afford the corresponding product **2g** as a colourless liquid (24.4 mg, 0.124 mmol, 62%). The spectroscopic data matched with those reported in the literature. [4]

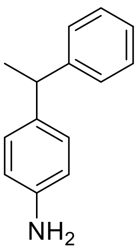

^{1}H NMR (600 MHz, $CDCl_3$) δ 7.33 – 7.27 (m, 2H), 7.26 – 7.23 (m, 2H), 7.22 – 7.17 (m, 1H), 7.07 – 7.03 (m, 2H), 6.66 (d, J = 8.0 Hz, 2H), 4.10 (q, J = 7.2 Hz, 1H), 3.25 (br s, 2H), 1.63 (d, J = 7.2 Hz).

^{13}C NMR (151 MHz, $CDCl_3$) δ 147.2, 136.9, 129.7, 128.5, 128.4, 127.6, 125.9, 115.5, 115.4, 44.0, 22.2.

HRMS (ESI-TOF): m/z $[M+H]^+$ Calcd. for $C_{14}H_{15}NH$ 198.1277, found 198.1274

4-(1-(p-tolyl)ethyl)aniline (2h)

[4] Li, X.; Feng, Y.; Lin, L.; Zou, G. *J. Org. Chem.* **2012**, *77*, 10991–10995.

To a previously dried Schlenk flask under nitrogen, CuBr (1.4 mg, 0.01 mmol, 5.0 mol%) and ligand DPPF (6.7 mg, 0.012 mmol, 6.0 mol%) were added in dry Et_2O (1 mL) and DCM (1 mL). After stirring for 10 min, the solution was cooled down to -30 ºC, and the substrate (55.1 mg, 0.2 mmol, 1.0 equiv.) was added in 1 mL of DCM, followed by the addition of *p*-tolyl magnesium chloride (Aldrich, 0.5 M solution in Et_2O, (1.2 mL, 0.6 mmol, 3.0 equiv)). The reaction mixture was stirred for 20h. The reaction was quenched with NH_4Cl sat sol (5 mL) and extracted with DCM (x3). The crude was purified by flash column chromatography using DCM as eluent to afford the corresponding product **2h** as a colourless liquid (30.1 mg, 0.142 mmol, 71%). The spectroscopic data matched with those reported in the literature.[5]

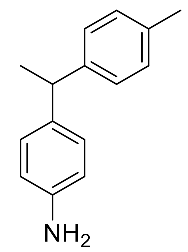

^1H NMR (600 MHz, $CDCl_3$) δ 7.14 – 7.06 (m, 4H), 7.04 – 6.97 (m, 2H), 6.66 – 6.57 (m, 2H), 4.03 (q, J = 7.2 Hz, 1H), 3.28 (br s, 2H), 2.31 (s, 3H), 1.60 (d, J = 7.2 Hz, 3H).

^{13}C NMR (151 MHz, $CDCl_3$) δ 144.2, 137.1, 135.4, 130.2, 129.1, 128.5, 127.5, 115.5, 43.6, 22.3, 21.1.

HRMS (ESI-TOF): m/z $[M+H]^+$ Calcd. for $C_{15}H_{17}NH$ 212.1434, found 212.1430

4-(1-(4-chlorophenyl)ethyl)aniline (2i)

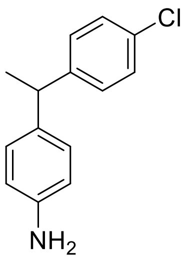

To a previously dried Schlenk flask under nitrogen, CuBr (1.4 mg, 0.01 mmol, 5.0 mol%) and ligand DPPF (6.7 mg, 0.012 mmol, 6.0 mol%) were added in dry Et_2O (1 mL) and DCM (1 mL). After stirring for 10 min, the solution was cooled down to -30 ºC, and the substrate (55.1 mg, 0.2 mmol, 1.0 equiv.) was added in 1 mL of DCM, followed by the addition of 4-chlorophenyl magnesium bromide (Aldrich, 1.0 M solution in Et_2O, (0.6 mL, 0.6 mmol, 3.0 equiv)). The reaction mixture was stirred for 20h. The reaction was quenched with NH_4Cl sat sol (5 mL) and extracted with DCM (x3). The crude was purified by flash column chromatography using DCM as eluent to afford the corresponding product **2i** as a colourless liquid (32.0 mg, 0.138 mmol, 69%).

^1H NMR (600 MHz, $CDCl_3$) δ 7.24 (d, J = 8.5 Hz, 2H), 7.14 (d, J = 8.5 Hz, 2H), 6.99 (d, J = 8.4 Hz, 2H), 6.63 (d, J = 8.4 Hz, 2H), 4.03 (q, J = 7.2 Hz, 1H), 3.45 (br s, 2H), 1.57 (d, J = 7.2 Hz, 3H).

^{13}C NMR (151 MHz, $CDCl_3$) δ 145.7, 136.2, 131.6, 129.0, 128.5, 128.5, 116.8, 115.5, 43.5, 22.1.

HRMS (ESI-TOF): m/z $[M+H]^+$ Calcd. for $C_{14}H_{14}ClNH$ 232.0884, found 232.0887

4-(but-3-en-2-yl)aniline (2j)

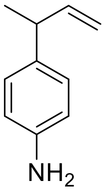

To a previously dried Schlenk flask under nitrogen, CuBr (1.4 mg, 0.010 mmol, 5.0 mol%) and ligand DPPF (6.7 mg, 0.012 mmol, 6.0 mol%) were added in dry Et_2O (1 mL) and DCM (1 mL). After stirring for 10 min, the solution was cooled down to -30 ºC, and the substrate (55.1 mg, 0.2 mmol, 1.0 equiv.) was added in 1 mL of DCM, followed by the addition of vinyl magnesium bromide (Aldrich, 1.0 M solution in Et_2O, (0.6 mL, 0.6 mmol, 3.0 equiv)). The reaction mixture was stirred for 20h. The reaction was quenched with NH_4Cl sat sol (5 mL) and extracted with DCM (x3). The crude was purified by flash column chromatography using DCM as eluent to afford the corresponding product **2j** (19.7 mg, 0.134 mmol, 67%) as a colourless liquid. The spectroscopic data matched with those reported in the literature.[6]

^1H NMR (600 MHz, $CDCl_3$) δ 7.09 – 6.93 (m, 2H), 6.66 – 6.64 (m, 2H), 5.98 (ddd, J = 16.9, 10.3, 6.4 Hz, 1H), 5.07 – 4.84 (m, 2H), 3.44 – 3.29 (m, 1H), 3.38 (br s, 2H), 1.32 (d, J = 7.0 Hz, 3H).

^{13}C NMR (151 MHz, $CDCl_3$) δ 144.5, 144.0, 135.9, 128.1, 115.4, 112.6, 42.4, 20.9.

HRMS (ESI-TOF): m/z $[M+H]^+$ Calcd. for $C_{10}H_{13}NH$ 148.1121, found 148.1117

4-(4-phenylbutan-2-yl)aniline (2k)

[5] Wang, X.; Wang, H.; Zhao, K.; Li, T.; Liu, S.; Yuan, H.; Shi, F. *J. Catal.* **2021**, *394*, 18–29.
[6] Movahhed, S.; Westphal, J.; Dindaroğlu, M.; Falk, A.; Schmalz, H. G. *Chem. - Eur. J.* **2016**, *22*, 7381–7384.

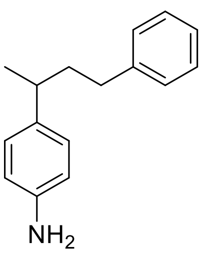

To a previously dried Schlenk flask under nitrogen, CuBr (1.4 mg, 0.010 mmol, 5.0 mol%) and ligand DPPF (6.7 mg, 0.012 mmol, 6.0 mol%) were added in dry Et_2O (1 mL) and DCM (1 mL). After stirring for 10 min, the solution was cooled down to -30 ºC, and the substrate (55.1 mg, 0.2 mmol, 1.0 equiv.) was added in 1 mL of DCM, followed by the addition of of phenethyl magnesium bromide Aldrich, 1.0 M solution in THF, (0.6 mL, 0.6 mmol, 3.0 equiv)). The reaction mixture was stirred for 20h. The reaction was quenched with NH_4Cl sat sol (5 mL) and extracted with DCM (x3). The crude was purified by flash column chromatography using DCM as eluent to afford the corresponding product **2k** as a colourless liquid (31.0 mg, 0.138 mmol, 69%).

^1H NMR (600 MHz, $CDCl_3$) δ 7.29 – 7.24 (m, 2H), 7.18 – 7.15 (m, 1H), 7.15 – 7.12 (m, 2H), 7.01 (d, J = 8.3 Hz, 2H), 6.66 (d, J = 7.9 Hz, 2H), 3.19 (br s, 2H), 2.63 (d, J = 7.1 Hz, 1H), 2.51 (t, J = 8.0 Hz, 2H), 1.92 – 1.80 (m, 2H), 1.23 (d, J = 6.9 Hz, 3H).

^{13}C NMR (151 MHz, $CDCl_3$) δ 142.9, 137.8, 128.5, 128.5, 128.3, 127.9, 125.7, 115.6, 40.3, 38.8, 34.1, 22.9.

HRMS (ESI-TOF): m/z $[M+H]^+$ Calcd. for $C_{16}H_{19}NH$ 226.1590, found 226.1589

4-(1-phenylpropan-2-yl)aniline (2l)

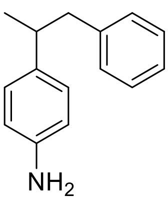

To a previously dried Schlenk flask under nitrogen, CuBr (1.4 mg, 0.01 mmol, 5.0 mol%) and ligand DPPF (6.7 mg, 0.012 mmol, 6.0 mol%) were added in dry Et_2O (1 mL) and DCM (1 mL). After stirring for 10 min, the solution was cooled down to -30 ºC, and the substrate (55.1 mg, 0.2 mmol, 1.0 equiv.) was added in 1 mL of DCM, followed by the addition of benzyl magnesium bromide (Aldrich, 2.0 M solution in Et_2O, (0.3 mL, 0.6 mmol, 3.0 equiv)). The reaction mixture was stirred for 20h. The reaction was quenched with NH_4Cl sat sol (5 mL) and extracted with DCM (x3). The crude was purified by flash column chromatography using DCM as eluent to afford the corresponding product **2l** as a colourless liquid (28.0 mg, 0.133 mmol, 66%).

^1H NMR (600 MHz, $CDCl_3$) δ 7.29 – 7.24 (m, 2H), 7.18 – 7.15 (m, 1H), 7.15 – 7.12 (m, 2H), 7.01 (d, J = 8.3 Hz, 2H), 6.66 (d, J = 7.9 Hz, 2H), 3.19 (br s, 2H), 2.63 (d, J = 7.1 Hz, 1H), 2.51 (t, J = 8.0 Hz, 2H), 1.92 – 1.80 (m, 2H), 1.23 (d, J = 6.9 Hz, 3H).
^{13}C NMR (151 MHz, $CDCl_3$) δ 144.3, 141.2, 137.4, 129.3, 128.1, 127.9, 125.8, 115.3, 45.4, 41.1, 21.5.

HRMS (ESI-TOF): m/z $[M+H]^+$ Calcd. for $C_{15}H_{17}NH$ 212.1439, found 212.1435

4-(hex-5-en-3-yl)aniline (2m)

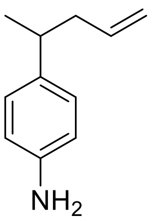

To a previously dried Schlenk flask under nitrogen, CuBr (1.4 mg, 0.01 mmol, 5.0 mol%) and ligand DPPF (6.7 mg, 0.012 mmol, 6.0 mol%) were added in dry Et_2O (1 mL) and DCM (1 mL). After stirring for 10 min, the solution was cooled down to -30 ºC, and the substrate (55.1 mg, 0.2 mmol, 1.0 equiv.) was added in 1 mL of DCM, followed by the addition of allyl magnesium bromide (Aldrich, 3.0 M solution in Et_2O, (0.2 mL, 0.6 mmol, 3.0 equiv)). The reaction mixture was stirred for 20h. The reaction was quenched with NH_4Cl sat sol (5 mL) and extracted with DCM (x3). The crude was purified by flash column chromatography using DCM as eluent to afford the corresponding product **2m** as a colourless liquid (24.3 mg, 0.151 mmol, 75%).

^1H NMR (600 MHz, $CDCl_3$) δ 7.02 – 6.95 (m, 2H), 6.65 (d, J = 8.4 Hz, 2H), 5.75 – 5.68 (m, 1H), 5.14 – 4.76 (m, 2H), 3.23 (br s, 2H), 2.71 – 2.68 (m, 1H), 2.41 – 2.08 (m, 2H), 1.21 (d, J = 7.0 Hz, 3H).
^{13}C NMR (151 MHz, $CDCl_3$) δ 144.2, 137.6, 137.5, 127.9, 115.7, 115.4, 43.1, 39.0, 21.8.

HRMS (ESI-TOF): m/z $[M+H]^+$ Calcd. for $C_{11}H_{15}NH$ 162.1283, found 162.1277.

4. Enantioselective Catalytic reaction for the Synthesis of chiral anilines

4.1. Screening of ligands

General procedure

To a previously dried Schlenk flask under nitrogen, CuBr•SMe$_2$ (5.0 mol%) and ligand (6.0 mol%) were added and dissolved in dry DCM (2 mL). After 15 min stirring at room temperature the solution was cooled down to -50 °C and the substrate (0.2 mmol, 1.0 equiv.) was added in 1mL of DCM. After stirring for an additional 10 min, EtMgBr (Aldrich, 3.0M solution in Et$_2$O, (3.0 equiv.) was added. The reaction mixture was stirred overnight (16-20h). The reaction was quenched with NaHCO$_3$ sat. sol., and extracted with DCM (3 x 5 mL), dried over Na$_2$SO$_4$. filtered and removed under vaccum. The resulting residue was purified by flash column chromatography using the appropriate eluent (generally DCM). The enantiomeric ratio was determined by analytical HPLC using a chiral column.

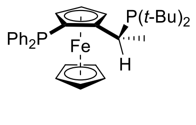

L1, SL-J002-1

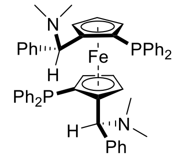

L2, SL-M001-2

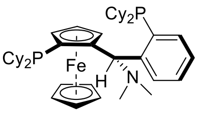

L3, SL-T002-2

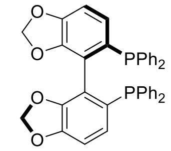

L4, *(R)*-SEGPHOS

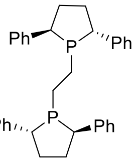

L5, (*R*,*R*)-Ph-BPE

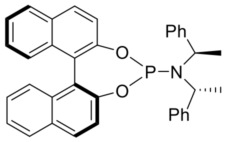

L6, *(S,S,S)*-LEGGY

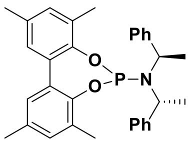

L7

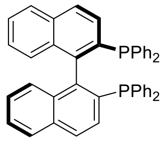

L8, (R)-BINAP

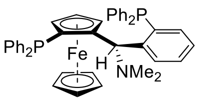

L9, SL-T001-2

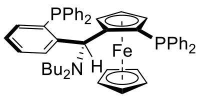

L10

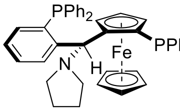

L11

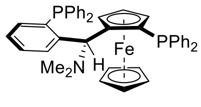

L12, SL-T001-1

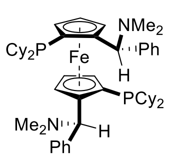

L13, SL-M002-1

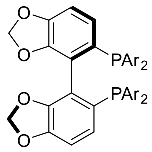

Ar = 3,5-dimethylphenyl
L14, *(R)*-DM-SEGPHOS

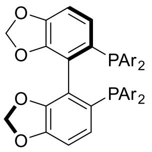

Ar = 3,5-di-tert-butyl-4-methoxyphenyl
L15, *(R)*-DTBM-SEGPHOS

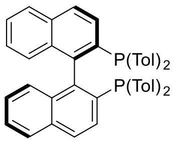

L16, (*R*)-Tol-BINAP

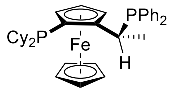

L17, SL-J004-1

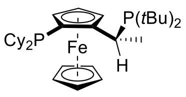

L18, SL-J009-1

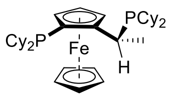

L19, SL-J003-1

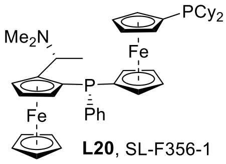

L20, SL-F356-1

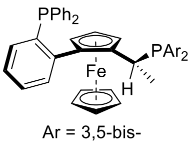

Ar = 3,5-bis-(trifluoromethyl)phenyl,
L21, SL-W001-1

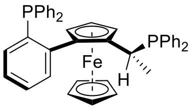

L22, SL-W002-1

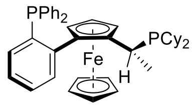

L23, SL-W003-1

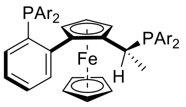

Ar = 3,5-bis-(methyl)phenyl
L24, SL-W009-1

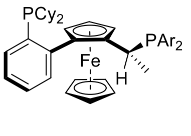

Ar = 3,5-bis-(trifluoromethyl)phenyl
L25, SL-W008-1

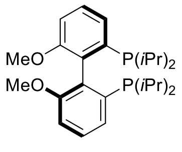

L26, SL-A116-1

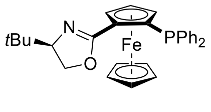

L27, SL-N004-1

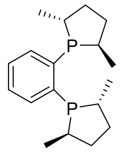

L28, (*R,R*)-Me-DUPHOS

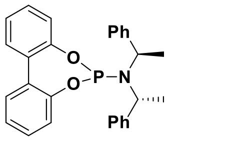

L29

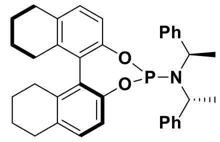

L30

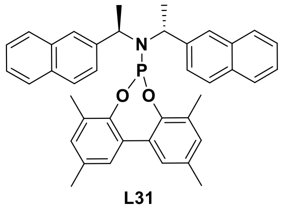

L31

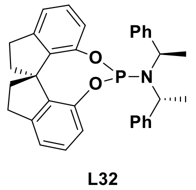

L32

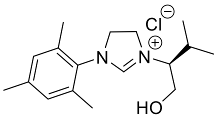

L33

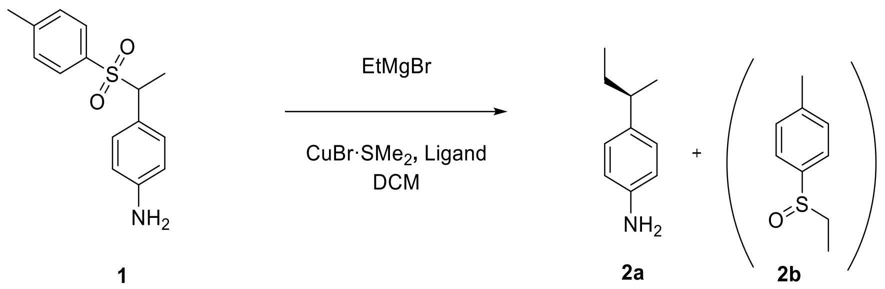

Entry	Time	Ligand	Temp. (ºC)	Conversion (%)	Yield[a] (%)	e.r.[b]
**1**	16h	--	-50	99 (2a: 2b: 92:8)	52	50:50
**2**	20h	**L17**, SL-J004-1	-50	Full (2a: 2b: 83: 17)	91	60:40
**3**	**20h**	**L12**, SL-**T001-2**	**-50**	**Full (2a: 2b: 87: 13)**	**57**	**85:15**
**4**	20h	**L5**, (*R*,*R*)-BPE	-50	Full (2a: 2b: 90: 10)	97	50:50
**5**	16h	**L3**, SL-T002-2	-50	Full (2a: 2b: 100: 0)	>99	50:50
**6**	16h	**L10**	-50	>99 (2a:2b: 99:1)	60	15:85
**7**	16h	**L11**	-50	>99 (2a:2b: 99:1)	63	15:85
**8**	16h	**L13**, SL-M002-1	-50	Full (2a: 2b: 99: 1)	>99	50:50
**9**	16h	**L4**, (*R*)-SEGPHOS	-50	77 (2a: 2b: 86:14)	80	50:50
**10**	16h	**L14**, (*R*)-DM-SEGPHOS	-50	93 (2a: 2b: 92:8)	60	28:72
**11**	16h	**L15**, (*R*)-DTBM-SEGPHOS	-50	96 (2a: 2b: 93:7)	85	33:66
**12**	16h	**L8**, (*R*)-BINAP	-50	full (2a: 2b: 99:1)	>99	30:70
**13**	16h	**L16**, (*R*)-Tol-BINAP	-50	73 (2a: 2b: 99:1)	56	28.5:71.5

**14**	16h	**L1**, SL-J002-1	-50	94 (2a: 2b: 100:0)	59	33:66
**15**	16h	**L18**, SL-J009-1	-50	99 (2a: 2b: 97:3)	90	66:34
**16**	16h	**L19**, SL-J003-1	-50	99 (2a: 2b: 93:7)	90	40:60
**17**	16h	**L20**, SL-F356-1	-50	Full (2a: 2b: 82:18)	89	50:50
**18**	16h	**L21**, SL-W001-1	-50	Full (2a: 2b: 93:7)	52	37.5:62.5
**19**	16h	**L22**, SL-W002-1	-50	88 (2a: 2b: 99:1)	52	48.5:51.5
**20**	16h	**L23**, SL-W003-1	-50	Full (2a: 2b: 98:2)	60	47.5:52.5
**21**	16h	**L24**, SL-W009-1	-50	mixture	66	37:63
**22**	16h	**L25**, SL-W008-1	-50	Full (2a: 2b: 83:17)	61	50:50
**23**	16h	**L26**, SL-A116-1	-50	89 (2a: 2b: 97:3)	36	54:46
**24**	16h	**L27**, SL-N004-1	-50	Complex mixture	7	46:54
**25**	16h	**L12**, SL-T001-1	-50	Full (2a: 2b: 95:5)	64	17:83
**26**	16h	**L28**, (*R,R*)-Me-DUPHOS	-50	Full (2a: 2b: 96:4)	42	51:49
**27**	16h	**L7**	-50	>99	>99	30:70
**28**	16h	**L29**	-50	>99	80	28:72
**29**	16h	**L31**	-50	>99 (2a: 2b: 92:8)	75	28.5:71.5
**30**	16h	**L6**	-50	88 (2a: 2b: 87:13)	59	50:50
**31**	16h	**L30**	-50	>99 (2a: 2b: 77:33)	77	61:39
**32**	16h	**L32**	-50	mixture	traces	50:50
**33**	16h	**L33**	-50	>99	30	50:50

Reaction conditions: $CuBr{\bullet}SMe_2$ (5.0 mol%), ligand **L** (6.0 mol%) in dry DCM (2 mL). The, substrate **1a** (0.2 mmol, 1.0 equiv.) in 1 mL of DCM. After 10 min, EtMgBr (Aldrich, 3.0 M solution in Et_2O, (3.0 equiv.) Reaction time (16-20h).
[a] Determined by NMR
[b] Isolated yield
[c] The enantiomeric ratio was determined by analytical chiral HPLC using a OJ-H column and 98:2 Heptane: iPropanol, 1mL/min.

4.2. Synthesis of chiral 4-(sec alkyl)anilines

General procedure for the catalytic reaction

To a previously dried Schlenk flask under nitrogen, CuBr (5.0 mol%) and ligand (6.0 mol%) were added and dissolved in dry DCM (1 mL) and Et_2O (1 mL). After 15 min stirring at room temperature the solution was cooled down to -50 °C and the substrate (0.2 mmol, 1.0 equiv.) was added in 1mL of DCM. After stirring for an additional 10 min, EtMgBr (Aldrich, 3.0 M solution in Et_2O, (3.0 equiv.) was added. The reaction mixture was stirred overnight (16-20h). The reaction was quenched with $NaHCO_3$ sat. sol., and extracted with DCM (3 x 5 mL), dried over Na_2SO_4 anh. filtered and removed under vacuum. The resulting residue was purified by flash column chromatography using DCM. The enantiomeric ratio was determined by analytical HPLC.

General procedure for the synthesis of the racemic product (HPLC separation)

To a previously dried Schlenk flask under nitrogen, CuBr (5.0 mol%) was added and dissolved in dry DCM (1 mL) and Et_2O (1 mL). The solution was cooled down and the substrate (0.2 mmol, 1.0 equiv.) was added in 1mL of solvent. After stirring for an additional 10 min, EtMgBr (Aldrich, 3.0 M solution in Et_2O, (3.0 equiv.) was added. The reaction mixture was stirred overnight (16-20h). The reaction was quenched with $NaHCO_3$ sat. sol., and extracted with DCM (3 x 5 mL), dried over Na_2SO_4 anh. filtered and removed under vacuum. The resulting residue was purified by flash column chromatography using DCM.

(*R*)-4-(sec-butyl)aniline ((*R*)-2a)

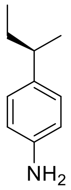

To a previously dried Schlenk flask under nitrogen, CuBr (5.0 mol%) and ligand (6.0 mol%) were added and dissolved in dry DCM (1 mL) and Et_2O (1 mL). After 15 min stirring at room temperature the solution was cooled down to -50 °C and the substrate (55.1 mg, 0.2 mmol, 1.0 equiv.) was added in 1mL of solvent. After stirring for an additional 10 min, EtMgBr (Aldrich, 3.0 M solution in Et_2O, (3.0 equiv.) was added. The reaction mixture was stirred overnight (16-20h). The reaction was quenched with $NaHCO_3$ sat. sol., and extracted with DCM (3 x 5 mL), dried over Na_2SO_4 anh., filtered and removed under vaccum. The resulting residue was purified by flash column chromatography using DCM to afford the corresponding aniline **2a** as a colourless liquid (23.2 mg, 0.156 mmol, 78%). The enantiomeric ratio was found to be 87:13 e.r. by analytical HPLC OD-H 98:2 iPrOH Heptane 1 mL/min, λ= 242 nm, tr (major): 14. 8 min, tr (minor): 16.3 min.). The spectroscopic data matched with those reported in the literature.[3]

^1H NMR (400 MHz, $CDCl_3$) δ 6.98 (d, J = 8.2 Hz, 2H), 6.65 (d, J = 8.1 Hz, 2H), 3.52 (br s, 2H), 2.53 – 2.44 (m, 1H), 1.57 – 1.50 (m, 2H), 1.19 (d, J = 7.0 Hz, 3H), 0.81 (t, J = 7.4 Hz, 3H).

^{13}C NMR (151 MHz, $CDCl_3$) δ 144.2, 138.1, 127.9, 115.4, 40.9, 31.5, 22.2, 12.4.

HRMS (ESI-TOF): m/z $[M+H]^+$ Calcd. for $C_{10}H_{15}NH$ 150.1283, found 150.1276.

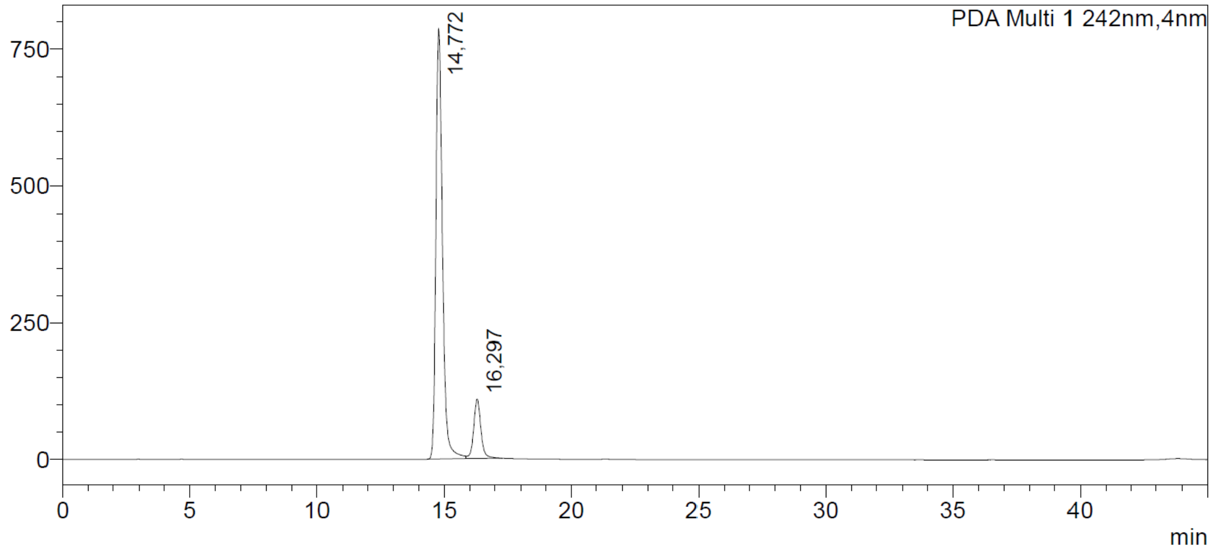

PDA Ch1 242nm

Peak#	Ret. Time	Area	Height	% Area
1	14,772	14246992	785898	86.749
2	16,297	2176137	109159	13,250
Total		16423129	895056	100,000

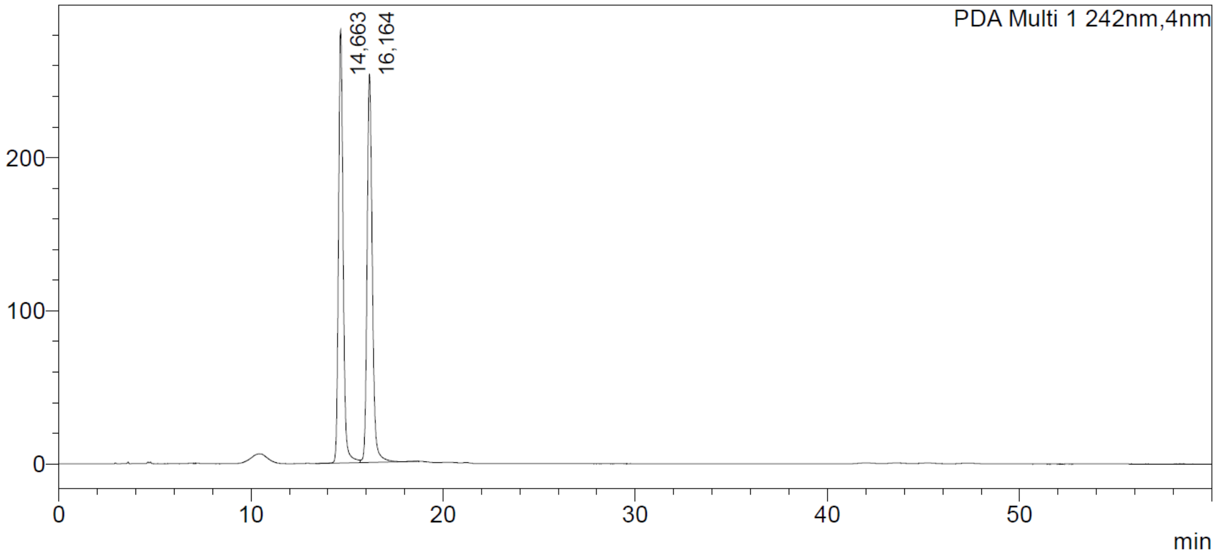

PDA Ch1 242nm

Peak#	Ret. Time	Area	Height	% Area
1	14,663	4761397	283156	49,800
2	16,164	4799622	253885	50,200
Total		9561019	537041	100,000

Scale up: To a previously dried Schlenk flask under nitrogen, CuBr (5.0 mol%) and ligand (6.0 mol%) were added and dissolved in dry DCM (5 mL) and Et_2O (5 mL). After 15 min stirring at room temperature the solution was cooled down to -50 °C and the substrate (275 mg, 1.0 mmol, 1.0 equiv.) was added in 5 mL of DCM. After stirring for an additional 10 min, EtMgBr (Aldrich, 3.0 M solution in Et_2O, (1.0 mL, 3.0 equiv.) was added. The reaction mixture was stirred overnight (16-20h). The reaction was quenched with $NaHCO_3$ sat. sol., and extracted with DCM (3 x 5 mL), dried over Na_2SO_4 anh., filtered and removed under vaccum. The resulting residue was purified by flash column chromatography using DCM to afford the corresponding aniline **2a** as a colourless liquid (126.4 mg, 0.847 mmol, 85%). The enantiomeric ratio was found to be 87:13 e.r. by analytical HPLC OD-H 98:2 *i*PrOH Heptane 1 mL/min, λ= 242 nm, tr (major): 14. 8 min, tr (minor): 16.3 min.).

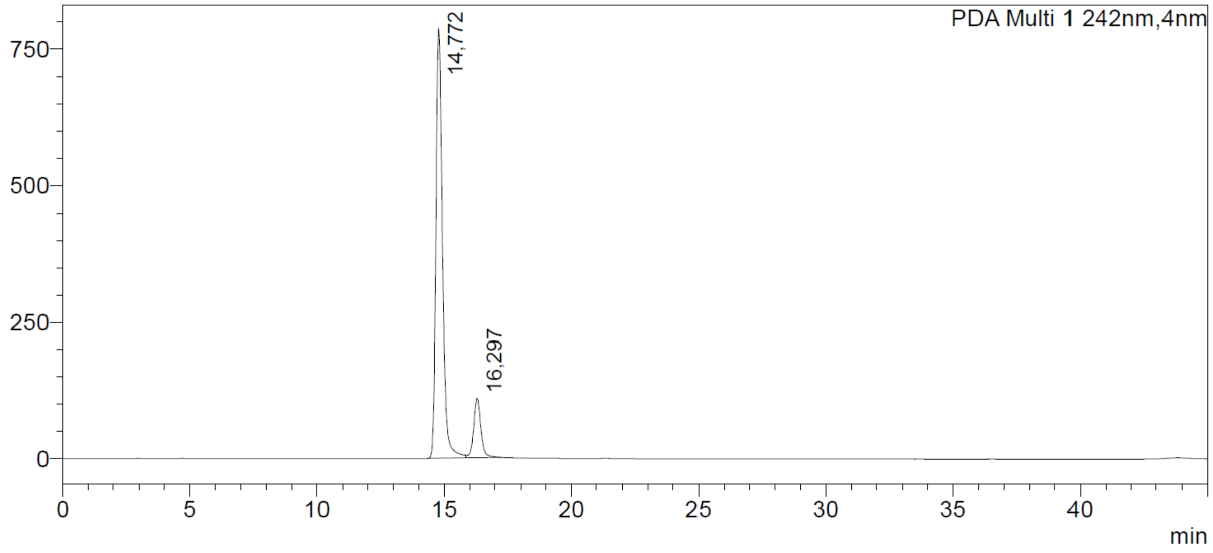

PDA Ch1 242nm

Peak#	Ret. Time	Area	Height
1	14,772	14246992	785898
2	16,297	2176137	109159
Total		16423129	895056

% Area
86.749
13,250
100,000

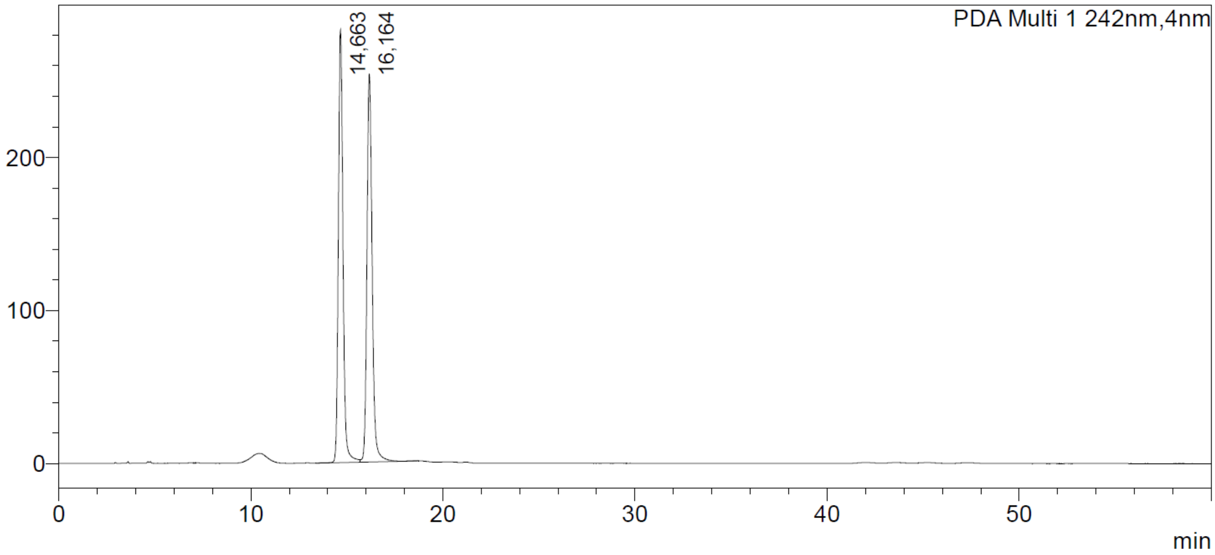

PDA Ch1 242nm

Peak#	Ret. Time	Area	Height	% Area
1	14,663	4761397	283156	49,800
2	16,164	4799622	253885	50,200
Total		9561019	537041	100,000

Retention Time : 14,772 min
Compound Name :
Spectrum Operation : None

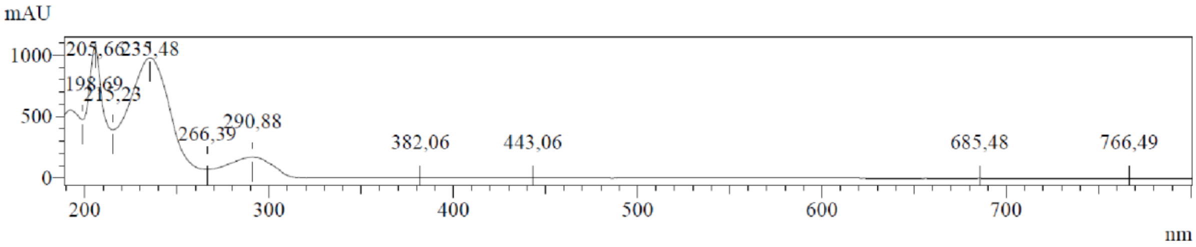

Retention Time : 16,297 min
Compound Name :
Spectrum Operation : None

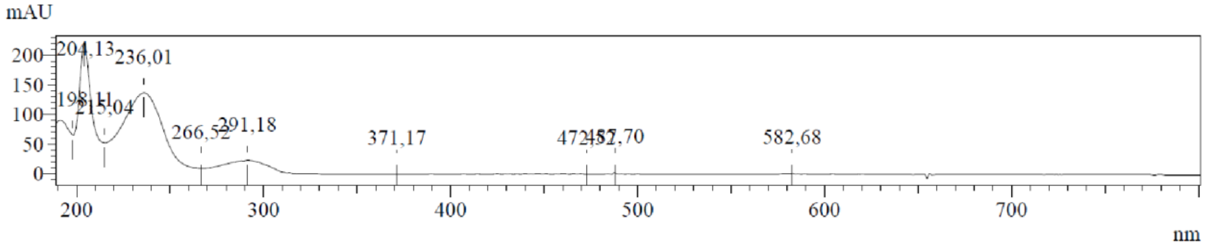

Substrate Scope

(*S*)-4-(hexan-3-yl)aniline ((*S*)-2n)

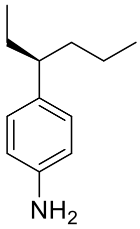

CuBr (1.4 mg, 0.01 mmol, 5.0 mol%) and ligand Taniaphos T001-2 (8.3 mg, 0.012 mmol, 6.0 mol%) were dissolved in dry DCM (1 mL) and Et_2O (1 mL). After 15 min stirring at room temperature, the solution was cooled down to -50 °C and the substrate (60.7 mg, 0.2 mmol, 1.0 equiv.) was added in 1mL of DCM. After stirring for an additional 10 min, EtMgBr (Aldrich, 3.0M solution in Et_2O, (0.2 mL, 3.0 equiv.) was added. The reaction mixture was stirred overnight (16-20h). The reaction is quenched with $NaHCO_3$ sat sol. (5 mL) and extracted with DCM (x3). The residue was purified by flash column chromatography in DCM to afford the corresponding aniline **2n** as a colourless liquid (24.9 mg, 0.14 mmol, 70%). The enantiomeric ratio was found to be 82:18 e.r. by analytical HPLC OB-H 95:5 heptane: *i*PrOH 0.5 mL/min, λ= 245 nm, tr (*minor*): 19.1 min, tr (*major*): 21.8 min.(*Due to the poor separation of the product* ***(S)-2n*** *in the HPLC, the ee of this compound was determined from the corresponding acetamide derivative.)*

^{1}H NMR (600 MHz, $CDCl_3$) δ 6.92 (d, J = 8.3 Hz, 2H), 6.64 (d, J = 8.3 Hz, 2H), 3.44 (br s, 2H), 2.32 - 2.27 (m, 1H), 1.65 – 1.52 (m, 2H), 1.51 – 1.44 (m, 2H), 1.21 – 1.10 (m, 2H), 0.84 (t, J = 7.4 Hz, 3H), 0.76 (t, J = 7.4 Hz, 3H).

^{13}C NMR (151 MHz, $CDCl_3$) δ 144.1, 136.4, 128.6, 115.3, 46.8, 39.1, 30.0, 20.9, 14.3, 12.4.

HRMS (ESI-TOF): m/z $[M+H]^+$ Calcd. for $C_{12}H_{19}NH$ 178.1596, found 178.1590.

<Chromatogram>

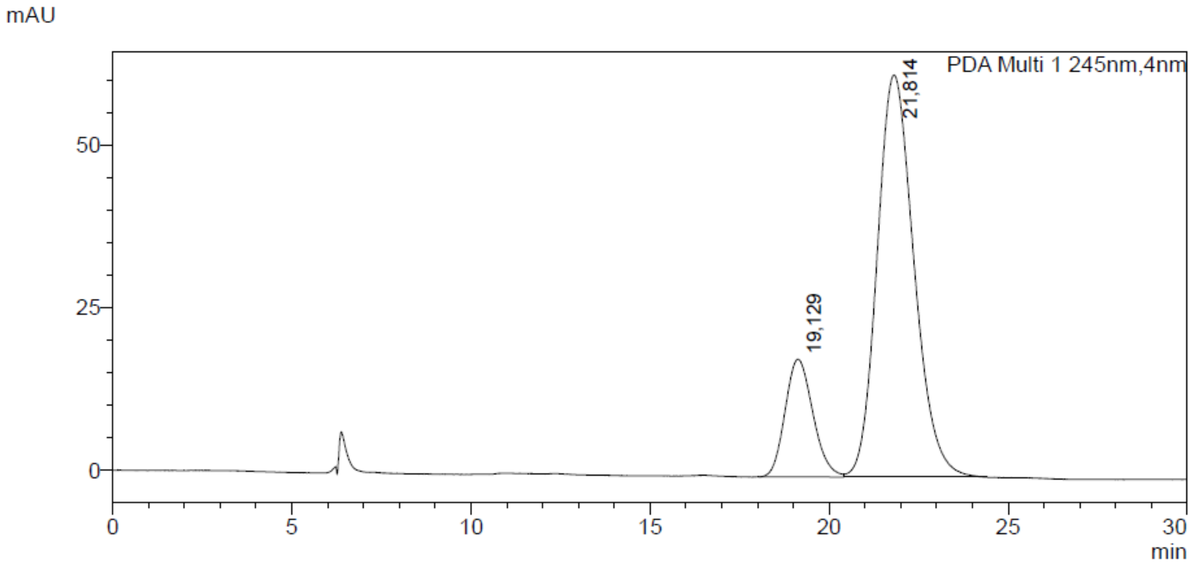

<Peak Table>

PDA Ch1 245nm

Peak#	Ret. Time	Area	Height	Area%
1	19,129	978688	18088	18,121
2	21,814	4422109	61863	81,879
Total		5400797	79950	100,000

<Chromatogram>

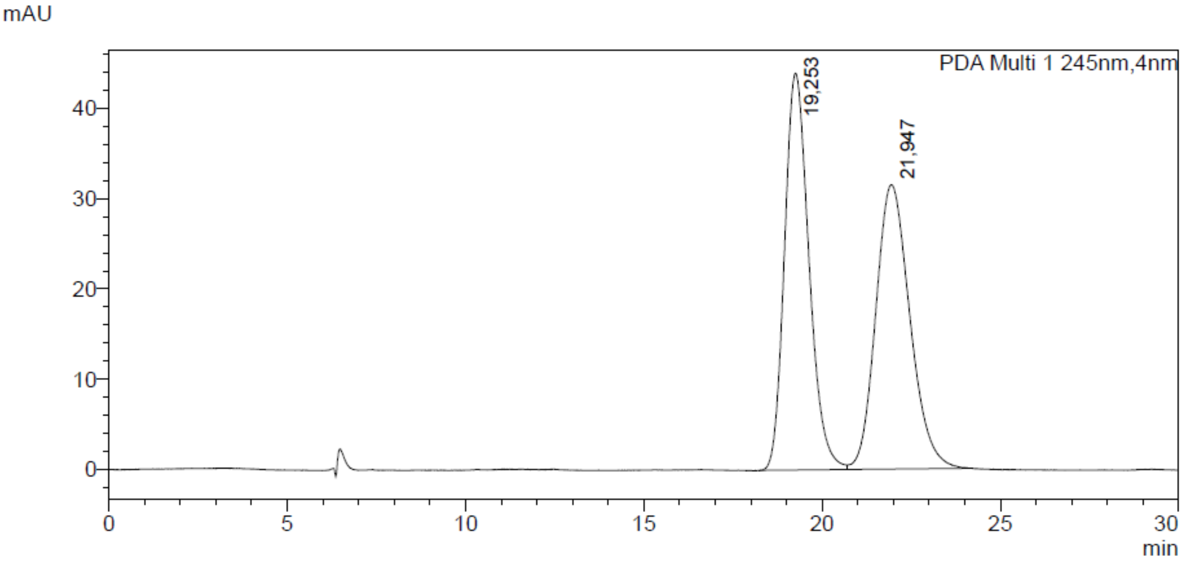

<Peak Table>

PDA Ch1 245nm

Peak#	Ret. Time	Area	Height	Area%
1	19,253	2096829	43919	49,999
2	21,947	2096928	31467	50,001
Total		4193757	75385	100,000

UV Spectrum

Peak# : 1
Retention Time : 19,129 min
Compound Name :
Spectrum Operation:

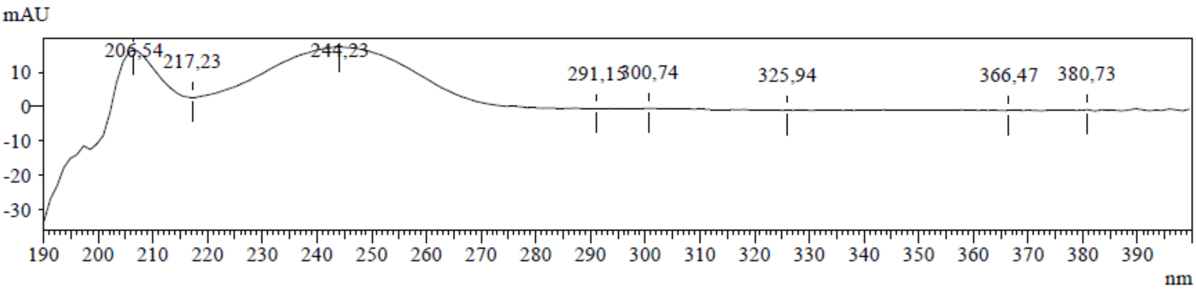

Peak# : 2
Retention Time : 21,814 min
Compound Name :
Spectrum Operation:

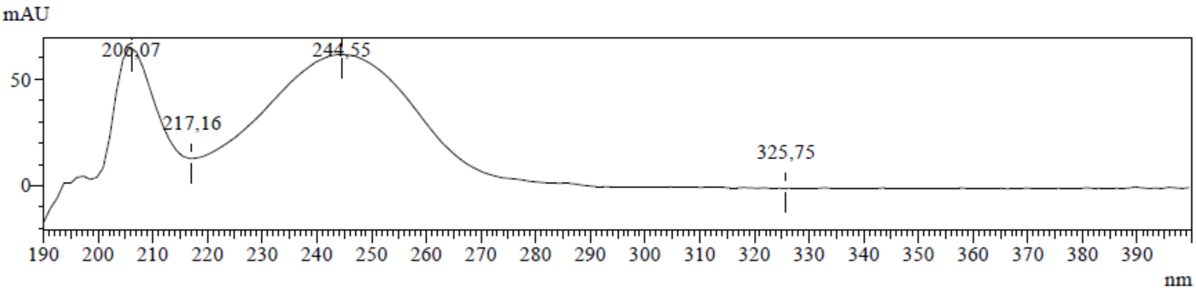

(*S*)-4-(octan-3-yl)aniline ((*S*)-2o)

CuBr (1.4 mg, 0.01 mmol, 5.0 mol%) and ligand Taniaphos T001-2 (8.3 mg, 0.012 mmol, 6.0 mol%) were dissolved in dry DCM (1 mL) and Et_2O (1 mL). After 15 min stirring at room temperature, the

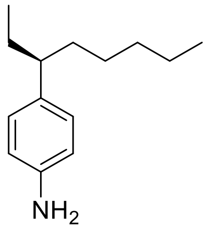

solution was cooled down and the substrate (66.3 mg, 0.2 mmol, 1.0 equiv.) was added in 1mL of DCM. After stirring for an additional 10 min, EtMgBr (Aldrich, 3.0M solution in Et_2O, (0.2 mL, 3.0 equiv.) was added. The reaction mixture was stirred overnight (16-20h). The reaction is quenched with $NaHCO_3$ sat sol. (5 mL) and extracted with DCM (x3). The residue was purified by flash column chromatography in DCM to afford the corresponding aniline **2o** as a colourless liquid (31.9 mg, 0.155 mmol, 78%). Enantiomeric ratio was found to be 80:20 e.r. by analytical HPLC in OB-H column 99.5:0.5 Heptane: *i*PrOH, 0.5 mL/min, λ= 239 nm, tr (major): 20.2 min, tr (minor): 21.8 min.

^{1}H NMR (400 MHz, $CDCl_3$) δ 6.98 (d, J = 8.2 Hz, 2H), 6. 65 (d, J = 8.2 Hz, 2H), 3.38 (br s, 2H), 2.60 – 2.55 (m, 1H), 1. 66 – 1. 46 (m, 4H), 1. 30 –1.13 (m, 11H), 0.87(t, J = 7.0 Hz, 3H).

^{13}C NMR (151 MHz, $CDCl_3$) δ 144.2, 138.4, 127.8, 115.4, 39.2, 38.8, 32.0, 29.6, 27.8, 22.8, 22.7, 14.2.

HRMS (ESI-TOF): m/z $[M+H]^+$ Calcd. for $C_{14}H_{23}NH$ 206.1909, found 206.1903

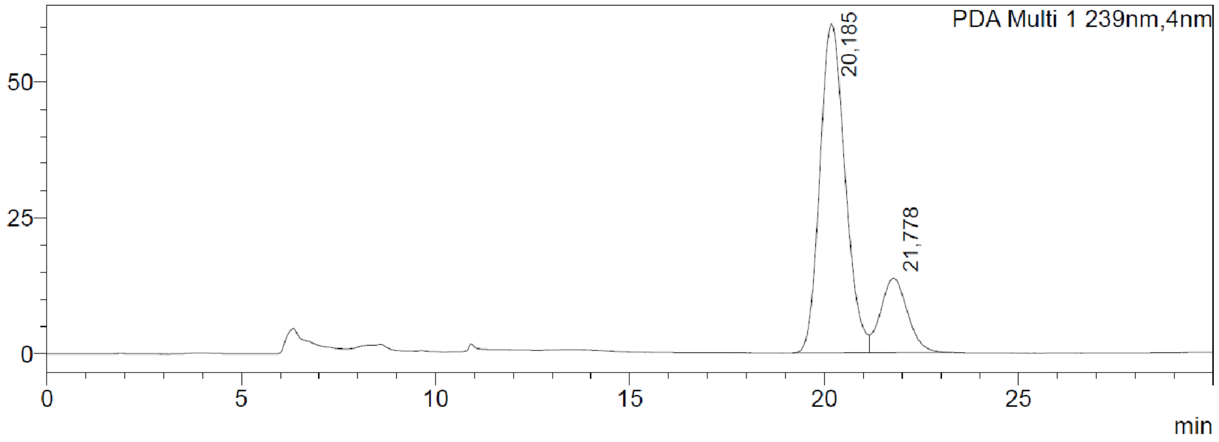

PDA Ch1 239nm

Peak#	Ret. Time	Area	Height	% Area
1	20,185	2732861	60534	80,374
2	21,778	667286	13724	19,625
Total		3400147	74258	100,000

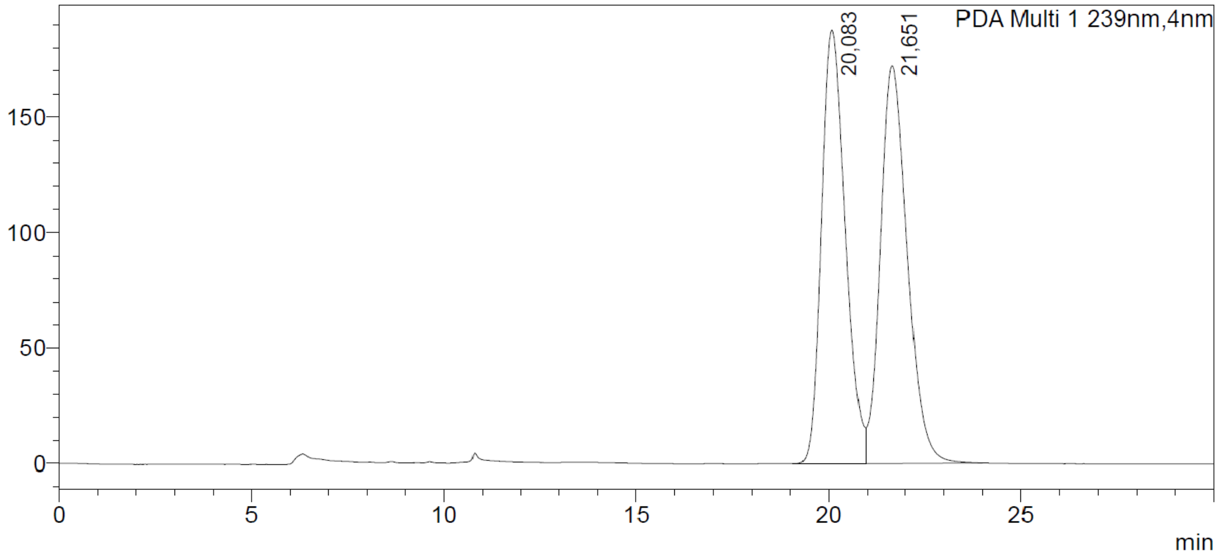

PDA Ch1 239nm

Peak#	Ret. Time	Area	Height	% Area
1	20,083	8173528	187992	49,152
2	21,651	8455630	172276	50,848
Total		16629157	360268	100,000

Retention Time : 20,185 min
Compound Name :
Spectrum Operation : None

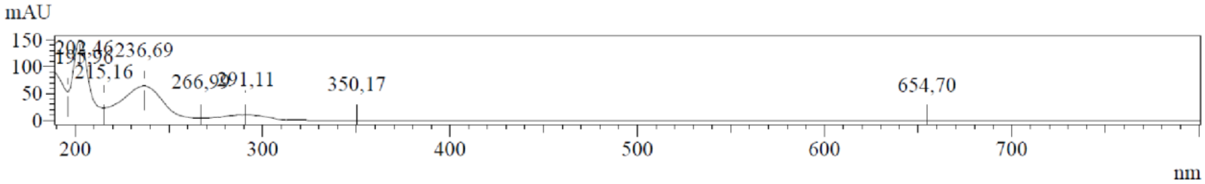

Retention Time : 21,778 min
Compound Name :
Spectrum Operation : None

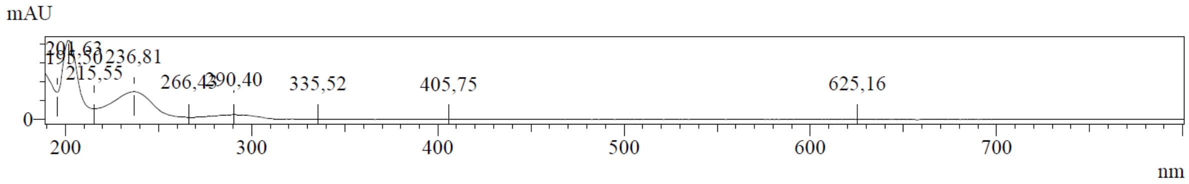

(*S*)-4-(1-phenylbutan-2-yl)aniline ((*S*)-2p)

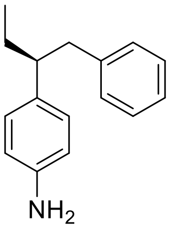

CuBr (1.4 mg, 0.01 mmol, 5.0 mol%) and ligand Taniaphos T001-2 (8.3 mg, 0.012 mmol, 6.0 mol%) were dissolved in dry DCM (1 mL) and Et_2O (1 mL). After 15 min stirring at room temperature, the solution was cooled down to -50 °C and the substrate (70.3 mg, 0.2 mmol, 1.0 equiv.) was added in 1mL of DCM. After stirring for an additional 10 min, EtMgBr (Aldrich, 3.0M solution in Et_2O, (0.2 mL, 3.0 equiv.) was added. The reaction mixture was stirred overnight (16-20h). The reaction is quenched with $NaHCO_3$ sat sol. (5 mL) and extracted with DCM (x3). The residue was purified by flash column chromatography in DCM to afford the corresponding aniline **2p** as a colourless liquid (26.3 mg, 0.117 mmol, 58%). The enantiomeric ratio was found to be 12:88 e.r. by analytical HPLC in OD-H 98:2 heptane: iPrOH 1 mL/min, λ= 239 nm, tr (*minor*): 19.7 min, tr (*major*): 21.6 min.

^{1}H NMR (400 MHz, $CDCl_3$) δ 7.23 – 7.19 (m, 2H), 7.16 – 7.09 (m, 1H), 7.03 (d, J = 7.1 Hz, 2H), 6.90 (d, J = 8.3 Hz, 2H), 6.61 (d, J = 8.3 Hz, 2H), 3.30 (br s, 2H), 2.91 – 2.74 (m, 2H), 2.65 – 2.57 (m, 1H), 1.73 – 1.63 (m, 1H), 1.59 – 1.48 (m, 1H), 0.76 (t, J = 7.4 Hz, 3H).

^{13}C NMR (101 MHz, $CDCl_3$) δ 144.3, 141.3, 135.4, 129.3, 128.7, 128.1, 125.7, 115.3, 49.0, 43.8, 28.6, 12.3.

HRMS (ESI-TOF): m/z $[M+H]^+$ Calcd. for $C_{16}H_{19}NH$ 226.1596, found 226.1590

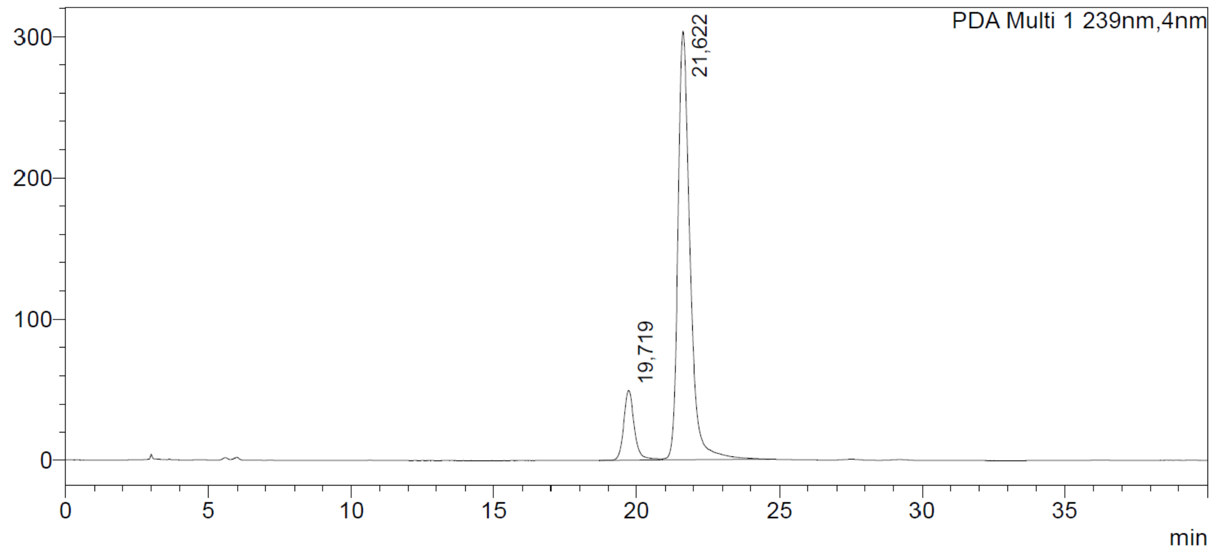

PDA Ch1 239nm

Peak#	Ret. Time	Area	Height
1	19,719	1300460	49541
2	21,622	9086960	303396
Total		10387419	352936

% Area
12,519
87,480
100,000

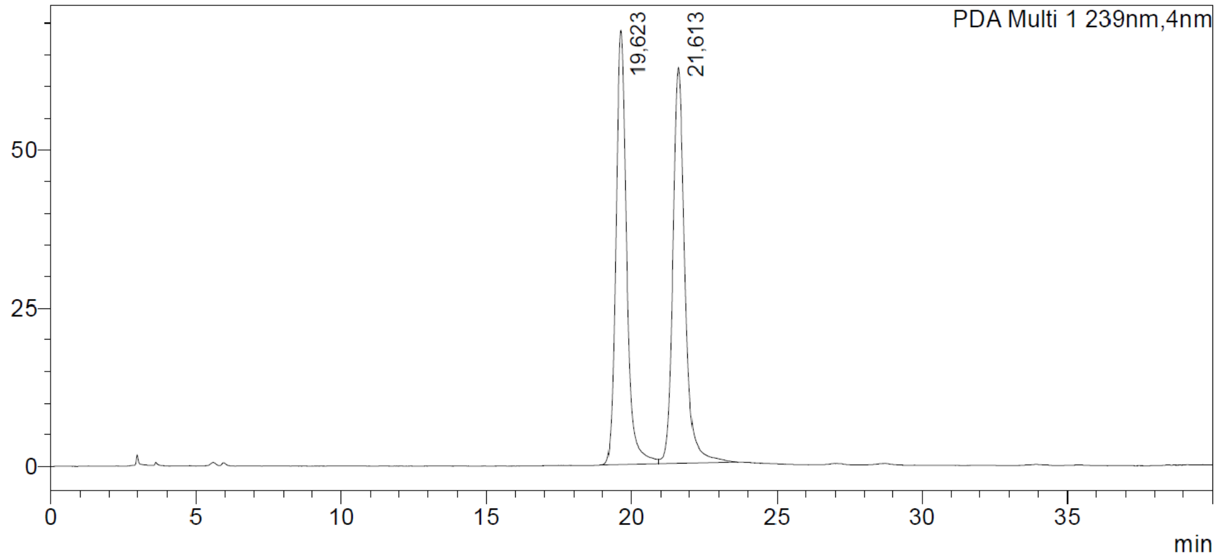

PDA Ch1 239nm

Peak#	Ret. Time	Area	Height
1	19,623	1782425	68648
2	21,613	1819807	62430
Total		3602232	131079

% Area
49,481
50,519
100,000

Retention Time : 19,719 min
Compound Name :
Spectrum Operation : None

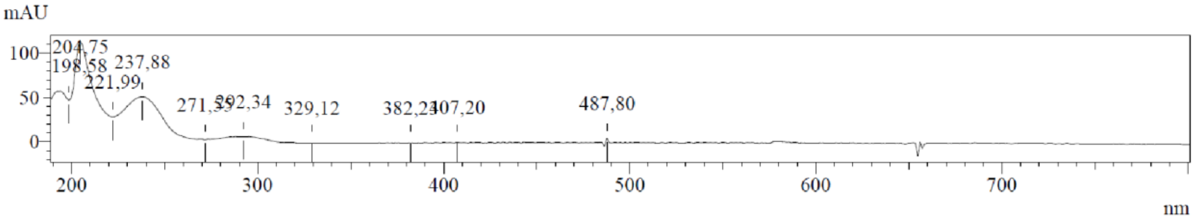

Retention Time : 21,622 min
Compound Name :
Spectrum Operation : None

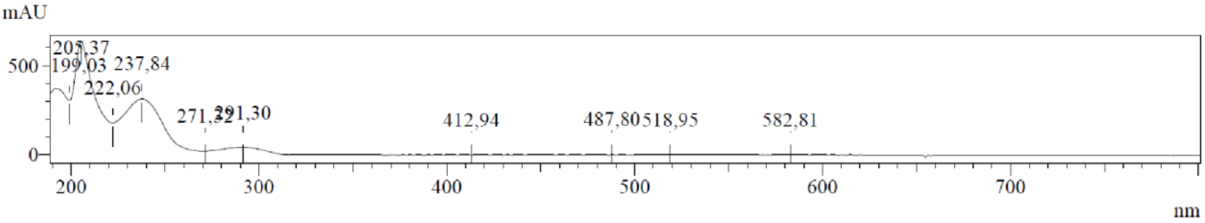

(*S*)-4-(5-methylhexan-3-yl)aniline ((*S*)-2q)

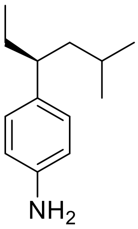

CuBr (1.4 mg, 0.01 mmol, 5.0 mol%) and ligand Taniaphos T001-2 (8.3 mg, 0.012 mmol, 6.0 mol%) were dissolved in dry DCM (1 mL) and Et_2O (1 mL). After 15 min stirring at room temperature, the solution was cooled down to -50 °C and the substrate (63.5 mg, 0.2 mmol, 1.0 equiv.) was added in 1mL of DCM. After stirring for an additional 10 min, EtMgBr (Aldrich, 3.0M solution in Et_2O, (0.2 mL, 3.0 equiv.)) was added. The reaction mixture was stirred overnight (16-20h). The reaction is quenched with $NaHCO_3$ sat sol. (5 mL) and extracted with DCM (x3). The residue was purified by flash column chromatography in DCM to afford the corresponding aniline **2q** as a colourless liquid (34.9 mg, 0.182 mmol, 91%). The enantiomeric ratio was found to be 79:21 e.r. by analytical chiral HPLC using as chiral column OB-H 98:2 heptane: *i*PrOH 1 mL/min, λ= 239 nm, tr (major): 6.1 min, tr (minor): 6.8 min.).

^{1}H NMR (400 MHz, $CDCl_3$) δ 6.94 (d, J = 7.9 Hz, 2H), 6.65 (d, J = 7.9 Hz, 2H), 3.54 (br s, 2H), 2.42-2.35 (m, 1H), 1.63 – 1.52 (m, 1H), 1.51 – 1.41 (m, 2H), 1.41 – 1.30 (m, 2H), 0.83 (dd, J = 11.1, 5.8 Hz, 6H), 0.75 (t, J = 7.3 Hz, 3H).

^{13}C NMR (151 MHz, $CDCl_3$) δ 144.0, 136.4, 128.6, 115.4, 46.2, 44.7, 30.5, 25.5, 23.8, 22.0, 12.3.

HRMS (ESI-TOF): m/z $[M+H]^+$ Calcd. for $C_{13}H_{21}NH$ 192.1752, found 192.1747

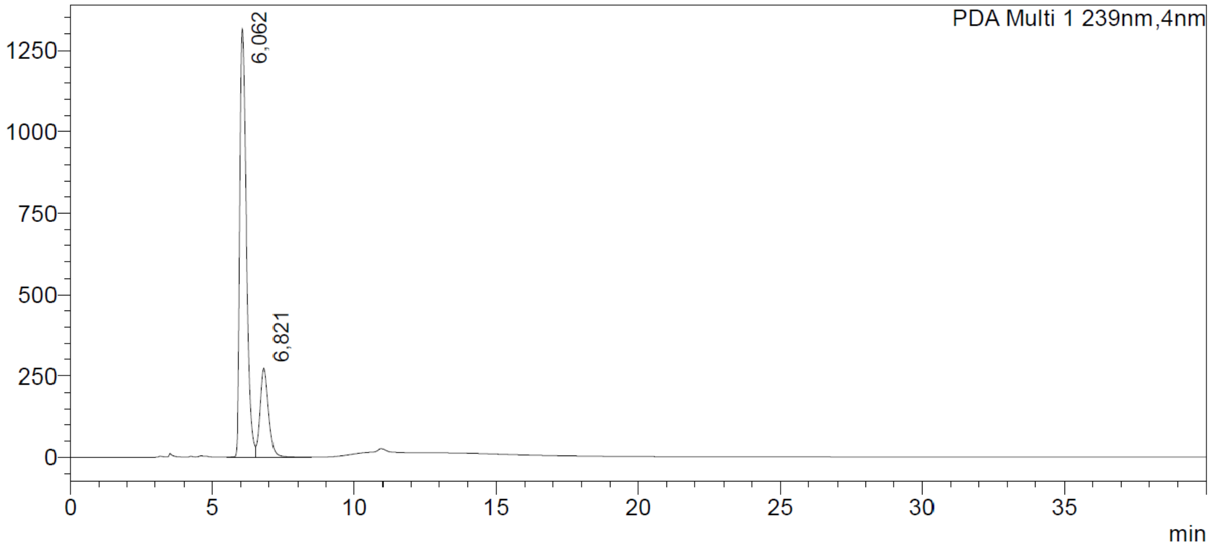

PDA Ch1 239nm

Peak#	Ret. Time	Area	Height
1	6,062	21349127	1314180
2	6,821	5642764	272976
Total		26991891	1587156

% Area
79,095
20,905
100,000

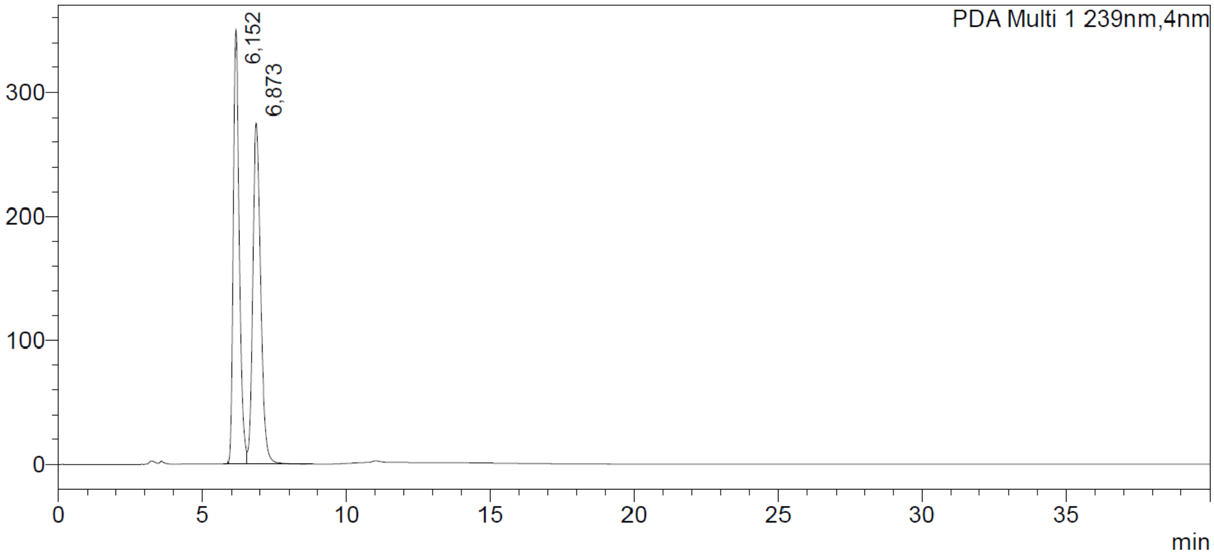

PDA Ch1 239nm

Peak#	Ret. Time	Area	Height
1	6,152	5175139	350303
2	6,873	5332153	275255
Total		10507292	625559

% Area
49,252
50,747
100,000

Retention Time : 6,062 min
Compound Name :
Spectrum Operation : None

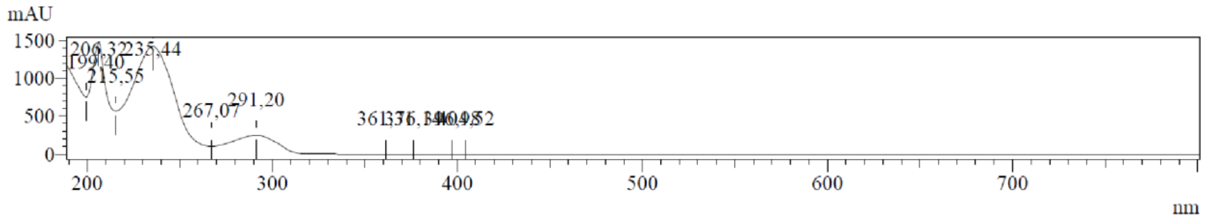

Retention Time : 6,821 min
Compound Name :
Spectrum Operation : None

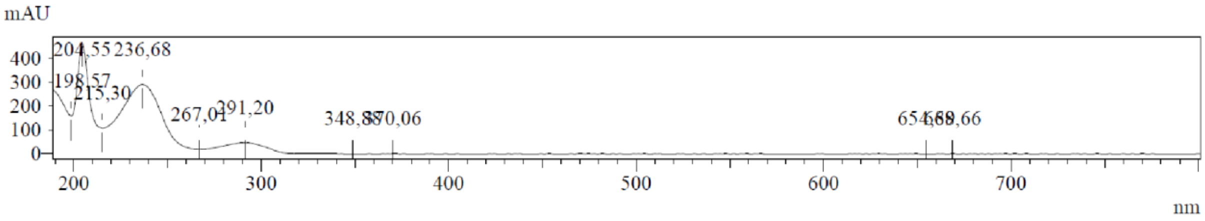

(*S*)-4-(hex-5-en-3-yl)aniline ((*S*)-2r)

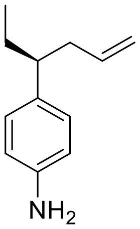

CuBr (1.4 mg, 0.01 mmol, 5.0 mol%) and ligand Taniaphos T001-2 (8.3 mg, 0.012 mmol, 6.0 mol%) were dissolved in dry DCM (1 mL) and Et_2O (1 mL). After 15 min stirring at room temperature, the solution was cooled down to -50 °C and the substrate (60.3 mg, 0.2 mmol, 1.0 equiv.) was added in 1mL of DCM. After stirring for an additional 10 min, EtMgBr (Aldrich, 3.0M solution in Et_2O, (0.2 mL, 3.0 equiv.)) was added. The reaction mixture was stirred overnight (16-20h). The reaction is quenched with $NaHCO_3$ sat sol. (5 mL) and extracted with DCM (x3). The residue was purified by flash column chromatography in DCM to afford the corresponding aniline **2r** as a colourless liquid (26.2 mg, 0.149 mmol, 75%). The enantiomeric ratio was found to be 86:14 e.r by analytical HPLC OJ-H column 98:2 Heptane: *i*PrOH, 1 mL/min, λ= 226 nm, tr (major): 23.2 min, tr (minor): 26.8 min.).

^{1}H NMR (600 MHz, $CDCl_3$) δ 6.94 (d, J = 8.3 Hz, 2H), 6.65 (d, J = 8.3 Hz, 2H), 5.80 – 5.56 (m, 1H), 4.95 (dt, J = 17.1, 1.8 Hz, 1H), 4.90 (ddt, J = 10.1, 2.2, 1.1 Hz, 1H), 3.65 (br s, 2H), 2.40 (m, 1H), 2.36 – 2.17 (m, 2H), 1.71 – 1.64 (m, 1H), 1.55 – 1.42 (m, 1H), 0.77 (t, J = 7.4 Hz, 3H).

^{13}C NMR (151 MHz, $CDCl_3$) δ 144.0, 137.7, 135.7, 128.6, 115.5, 115.4, 46.9, 41.2, 29.1, 12.2.

HRMS (ESI-TOF): m/z $[M+H]^+$ Calcd. for $C_{12}H_{17}NH$ 176.1439, found 176.1434

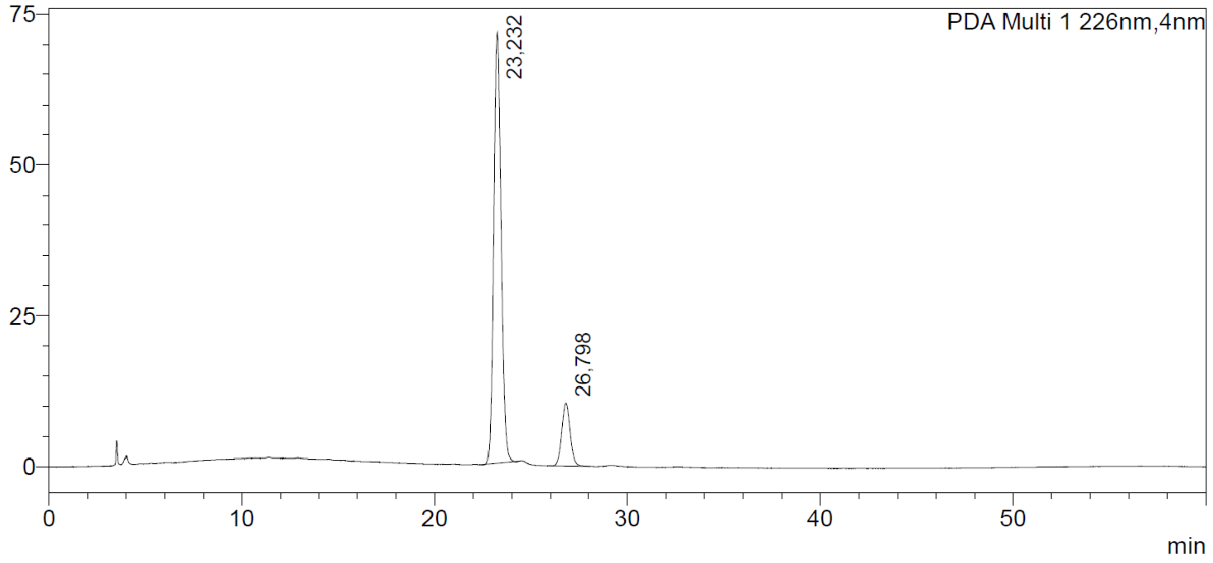

PDA Ch1 226nm

Peak#	Ret. Time	Area	Height
1	23,232	1901106	71233
2	26,798	319404	10409
Total		2220511	81643

% Area
85,615
14,384
100,000

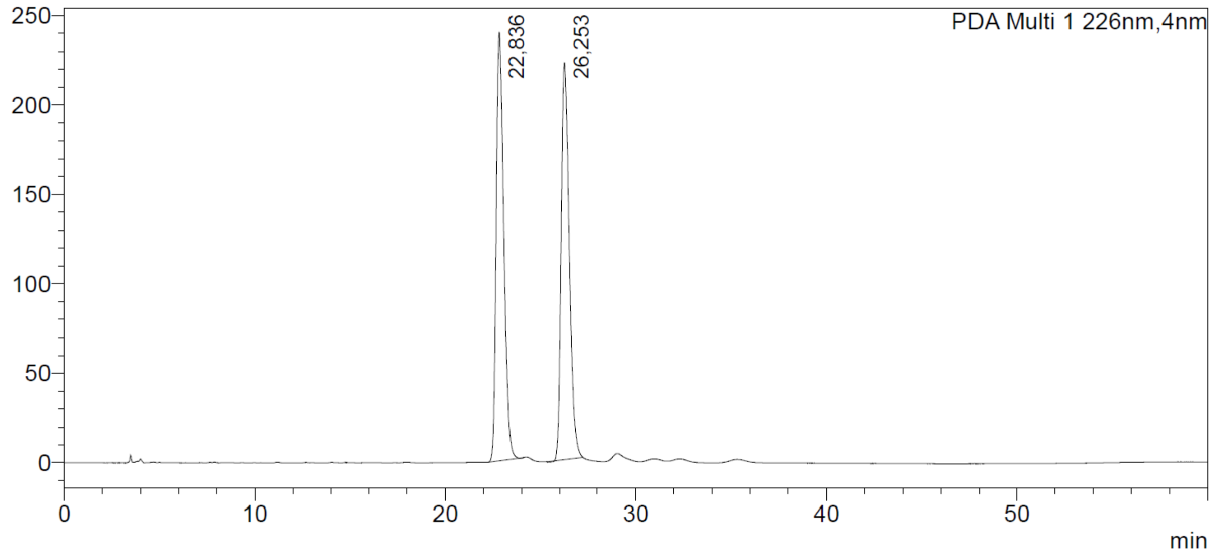

PDA Ch1 226nm

Peak#	Ret. Time	Area	Height
1	22,836	6576336	239409
2	26,253	6583583	222010
Total		13159919	461420

% Area
49,972
50,027
100,000

Retention Time : 23,232 min
Compound Name :
Spectrum Operation : None

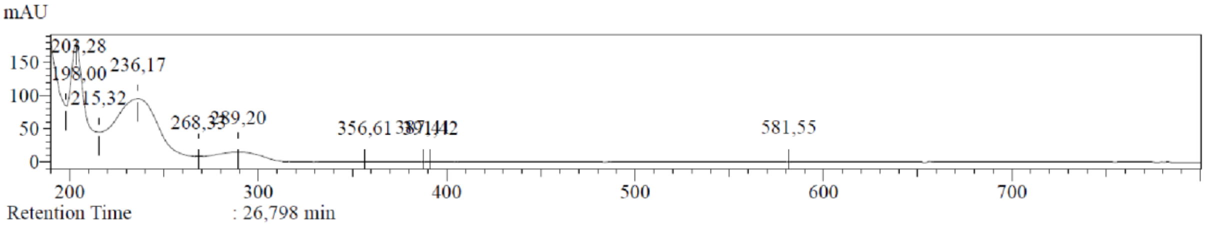

Retention Time : 26,798 min
Compound Name :
Spectrum Operation : None

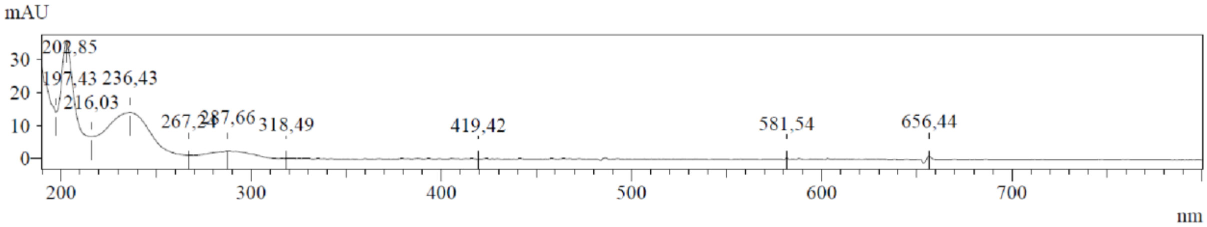

(*S*)-4-(7-methoxyheptan-3-yl)aniline ((*S*)-2s)

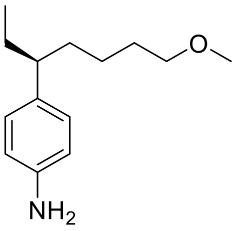

CuBr (1.4 mg, 0.01 mmol, 5.0 mol%) and ligand Taniaphos T001-2 (8.3 mg, 0.012 mmol, 6.0 mol%) were dissolved in dry DCM (1 mL) and Et_2O (1 mL). After 15 min stirring at room temperature, the solution was cooled down to -50 °C and the substrate (69.5 mg, 0.2 mmol, 1.0 equiv.) was added in 1mL of DCM. After stirring for an additional 10 min, EtMgBr (Aldrich, 3.0M solution in Et_2O, (0.2 mL, 3.0 equiv.)) was added. The reaction mixture was stirred overnight (16-20h). The reaction is quenched with $NaHCO_3$ sat sol. (5 mL) and extracted with DCM (x3). The residue was purified by flash column chromatography in DCM to afford the corresponding aniline **2s** as a colourless liquid (41.4 mg, 0.187 mmol, 93%). The enantiomeric ratio was found to be 48:52 e.r. by analytical HPLC in OB-H 98:2 heptane: iPrOH 1 mL/min, λ= 250 nm, tr (*enant*): 17.4 min, tr (*enant*): 19.2 min.).

^{1}H NMR (600 MHz, $CDCl_3$) δ 6.91 (d, J = 8.3 Hz, 2H), 6.64 (d, J = 8.3 Hz, 2H), 3.32-3.26 (m, 5H), 2. 42 – 2.26 (m, 1H), 1.64 – 1. 44 (m, 6H), 1.27 – 1.09 (m, 2H), 0.75 (t, J = 7.4 Hz, 3H).

^{13}C NMR (151 MHz, $CDCl_3$) δ 144.1, 136.2, 128.6, 115.4, 73.0, 58.6, 47.1, 36.7, 29.9, 29.9, 24.3, 12.3.

HRMS (ESI-TOF): m/z $[M+H]^+$ Calcd. for $C_{14}H_{23}NOH$ 222.1858, found 222.1850

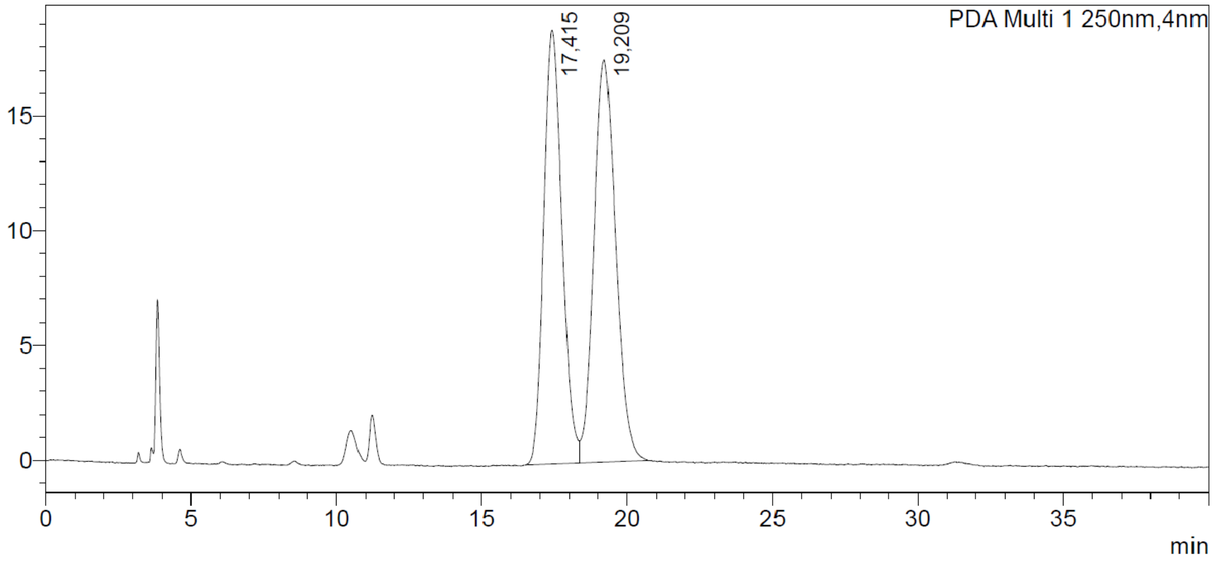

PDA Ch1 250nm

Peak#	Ret. Time	Area	Height	% Area
1	17,415	834267	18903	47,963
2	19,209	905125	17510	52,037
Total		1739391	36413	100,000

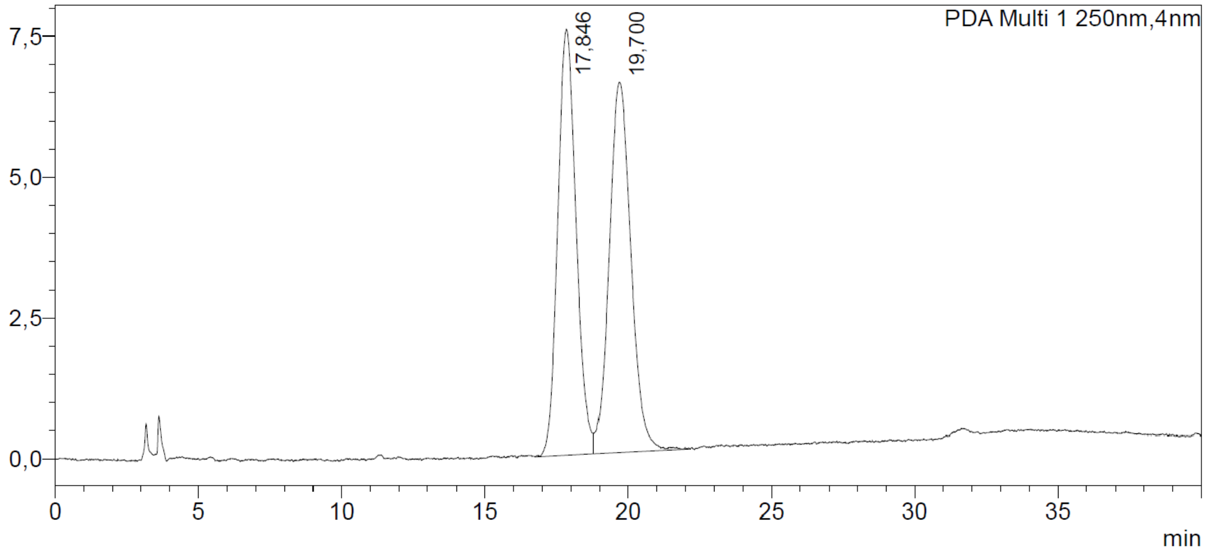

PDA Ch1 250nm

Peak#	Ret. Time	Area	Height	% Area
1	17,846	340635	7554	49,407
2	19,700	348813	6570	50,593
Total		689448	14124	100,000

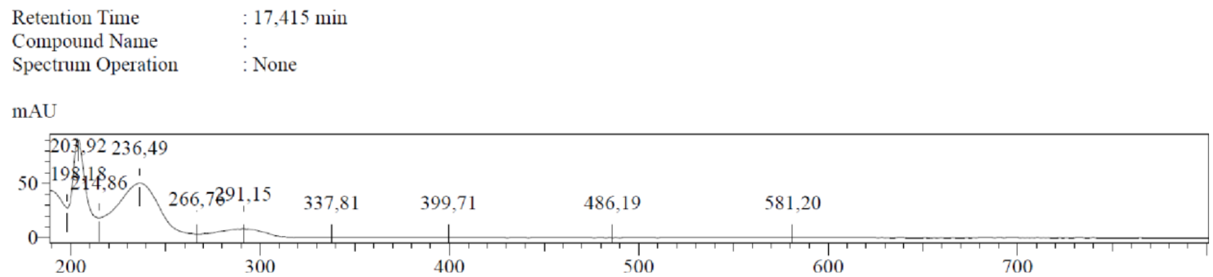

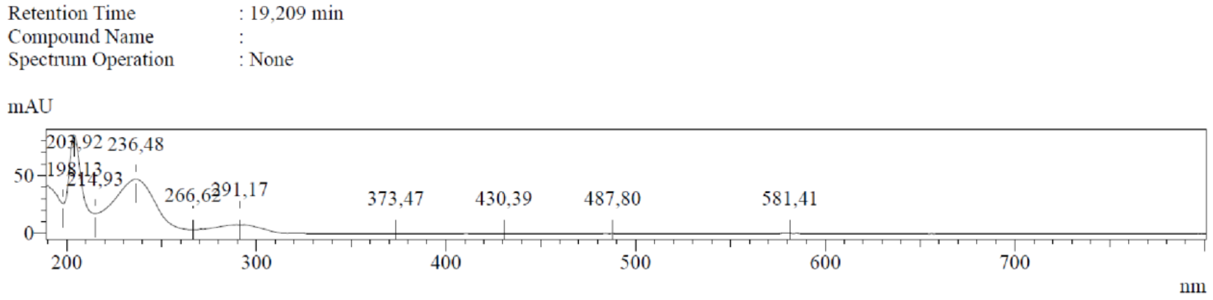

(*R*)-4-(sec-butyl)-2,6-dichloroaniline ((*R*)-2t)

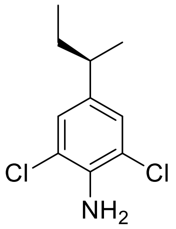

CuBr (1.4 mg, 0.01 mmol, 5.0 mol%) and ligand Taniaphos T001-2 (8.3 mg, 0.012 mmol, 6.0 mol%) were dissolved in dry DCM (1 mL) and Et_2O (1 mL). After 15 min stirring at room temperature, the solution was cooled down to -50 °C and the substrate (68.9 mg, 0.2 mmol, 1.0 equiv.) was added in 1mL of DCM. After stirring for an additional 10 min, EtMgBr (Aldrich, 3.0M solution in Et_2O, (0.2 mL, 3.0 equiv.)) was added. The reaction mixture was stirred overnight (16-20h). The reaction is quenched with $NaHCO_3$ sat sol. (5 mL) and extracted with DCM (x3). The residue was purified by flash column chromatography in DCM to afford the corresponding aniline **2t** as a colourless liquid (38.7 mg, 0.177 mmol, 89%). The enantiomeric ratio was found to be 67:33 e.r. by analytical HPLC in OJ-H 99.5:0.5 heptane: iPrOH 1 mL/min, λ= 252 nm, tr (*minor*): 12.8 min, tr (*major*): 17.7 min.

^{1}H NMR (600 MHz, $CDCl_3$) δ 7.00 (s, 2H), 4.28 (br s, 2H), 2.48 – 2.42 (m, 1H), 1.57 – 1.46 (m, 2H), 1.17 (d, J = 7.0 Hz, 3H), 0.81 (t, J = 7.4 Hz, 3H).

^{13}C NMR (151 MHz, $CDCl_3$) δ 138.4, 137.9, 126.5, 119.7, 40.8, 31.2, 22.0, 12.3.

HRMS (ESI-TOF): m/z $[M+H]^+$ Calcd. for $C_{10}H_{13}Cl_2NH$ 218.0503, found 218.0498

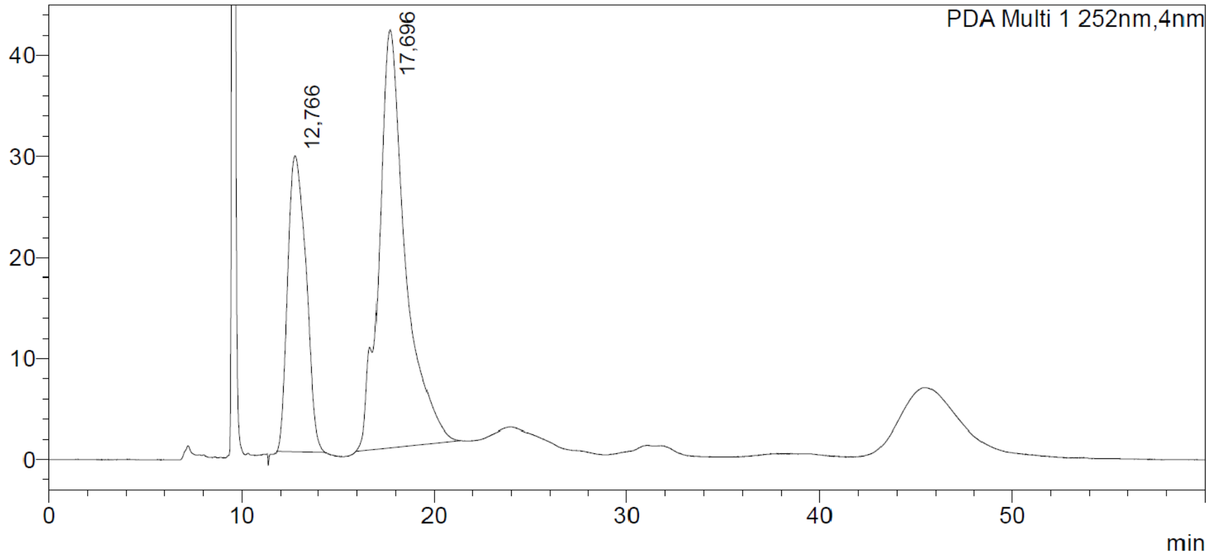

PDA Ch1 252nm

Peak#	Ret. Time	Area	Height	% Area
1	12,766	1970227	29281	33,275
2	17,696	3950803	41389	66,725
Total		5921029	70670	100,000

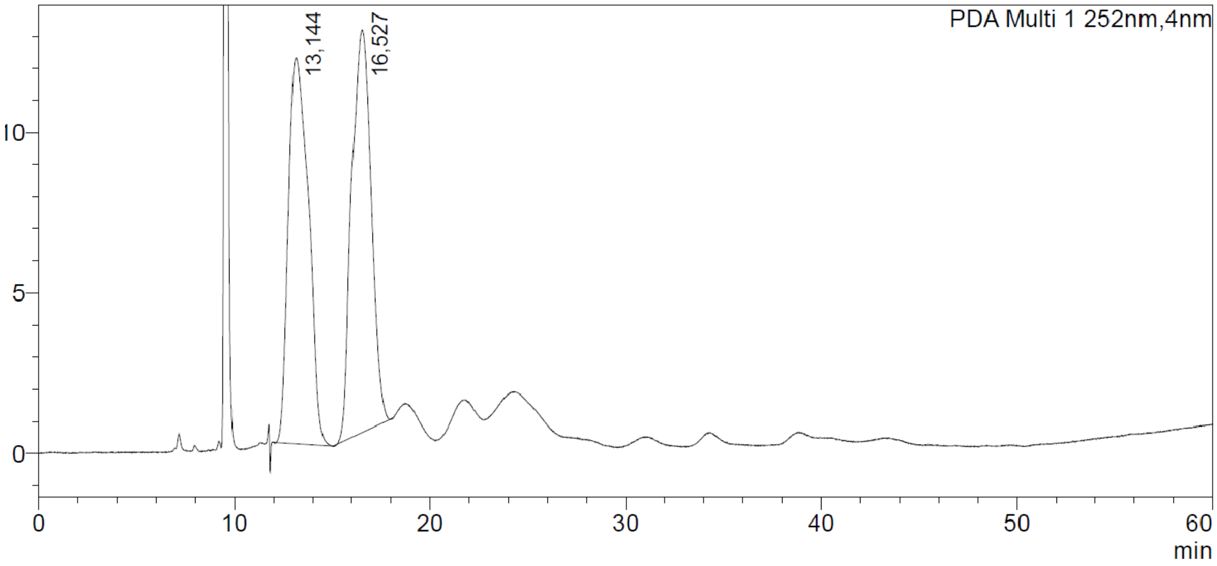

PDA Ch1 252nm

Peak#	Ret. Time	Area	Height	% Area
1	13,144	861863	12025	49,709
2	16,527	871937	12538	50,291
Total		1733800	24562	100,000

Retention Time : 12,766 min
Compound Name :
Spectrum Operation : None

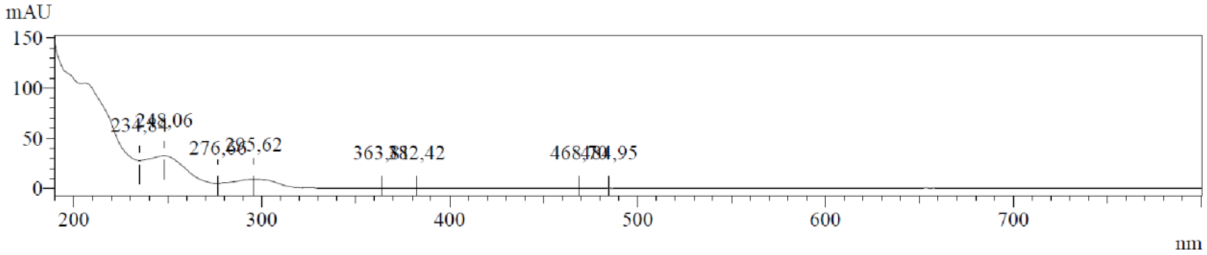

Retention Time : 17,696 min
Compound Name :
Spectrum Operation : None

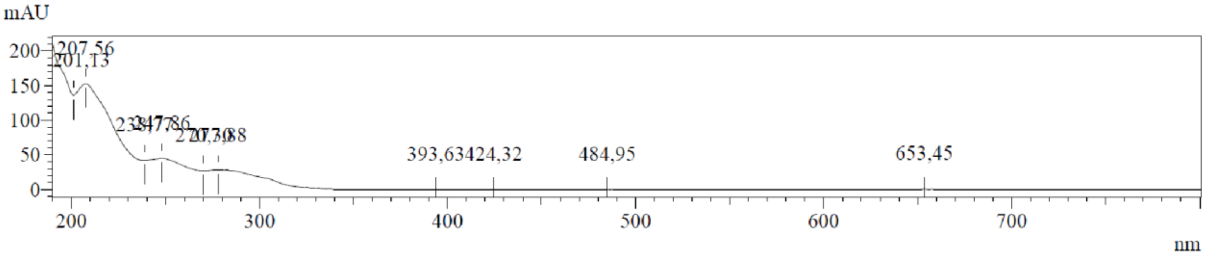

Grignard screening

(*R*)-4-(heptan-2-yl)aniline ((*R*)-2d)

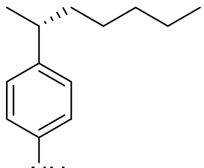

CuBr (1.4 mg, 0.01 mmol, 5.0 mol%) and ligand Taniaphos T001-2 (8.3 mg, 0.012 mmol, 6.0 mol%) were dissolved in dry DCM (1 mL) and Et_2O (1 mL). After 15 min stirring at room temperature, the solution was cooled down to -50 °C and the substrate (55.1 mg, 0.2 mmol, 1.0 equiv.) was added in 1mL of DCM. After stirring for an additional 10 min, pentyl MgBr (Aldrich, 2.0M solution in Et_2O, (0.3 mL, 3.0 equiv.)) was added. The reaction mixture was stirred overnight (16-20h). The reaction is quenched with $NaHCO_3$ sat sol. (5 mL) and extracted with DCM (x3). The residue was purified by flash column chromatography in DCM to afford the corresponding aniline **2d** as a colourless liquid (27.1 mg, 0.142 mmol, 71%). The enantiomeric ratio was found to be 22:78 e.r. by analytical HPLC in OB-H column 99.5:0.5 Heptane: *i*PrOH, 0.5 mL/min, λ= 239 nm, tr (*minor*): 26.8 min, tr (*major*): 31.4 min.

^{1}H NMR (600 MHz, $CDCl_3$) δ 6.98 (d, J = 8.3 Hz, 2H), 6.65 (d, J = 8.3 Hz, 2H), 3.38 (br s, 2H), 2.60 – 2.55 (m, 1H), 1.55 – 1.46 (m, 2H), 1.30 – 1.13 (m, 9H), 0.87 (t, J = 6.9 Hz, 3H).

^{13}C NMR (151 MHz, $CDCl_3$) δ 144.1, 138.4, 127.8, 115.4, 39.2, 38.8, 32.0, 29.6, 27.8, 22.7, 14.2.

HRMS (ESI-TOF): m/z $[M+H]^+$ Calcd. for $C_{13}H_{21}NH$ 192.1752, found 192.1747

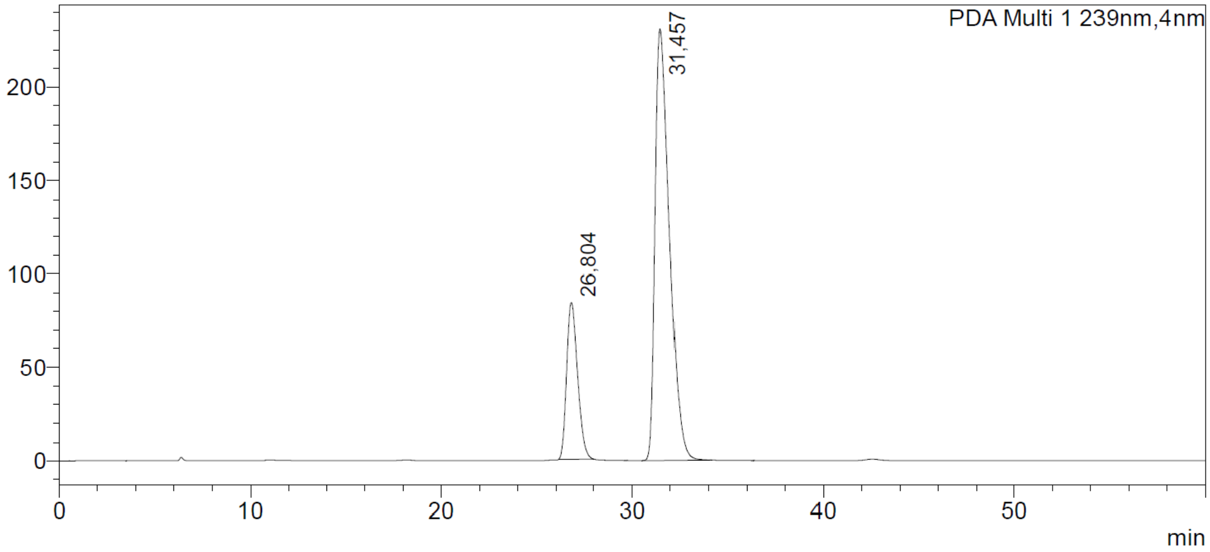

PDA Ch1 239nm

Peak#	Ret. Time	Area	Height
1	26,804	3380869	83914
2	31,457	12097263	230744
Total		15478132	314657

% Area
21,842
78,157
100,000

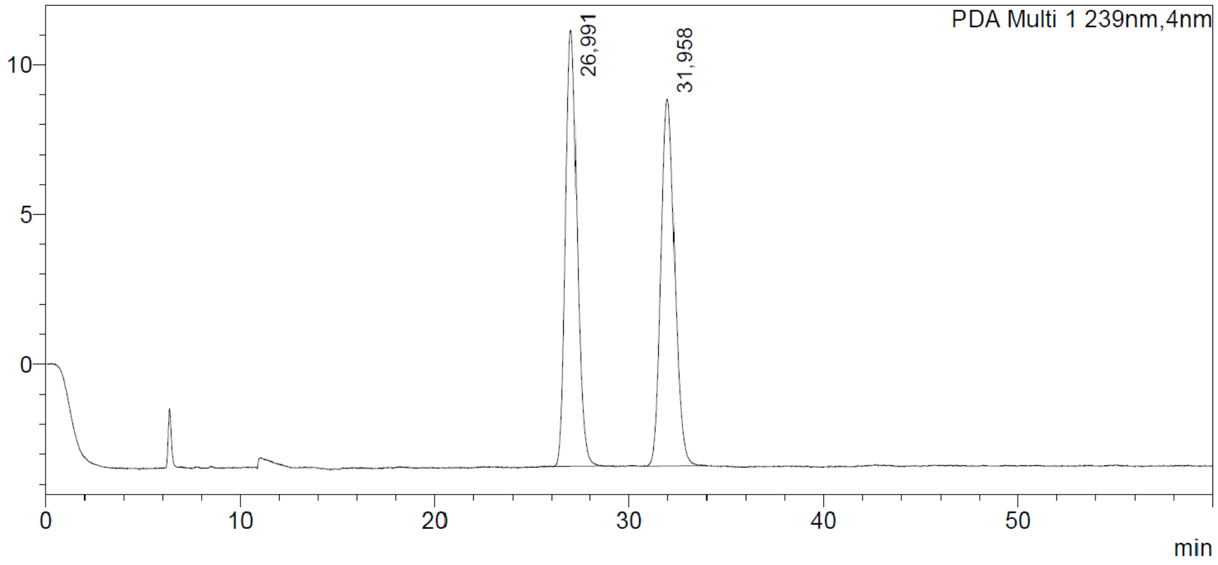

PDA Ch1 239nm

Peak#	Ret. Time	Area	Height
1	26,991	599167	14568
2	31,958	601817	12255
Total		1200983	26822

% Area
49,890
50,110
100,000

Retention Time : 26,804 min
Compound Name :
Spectrum Operation : None

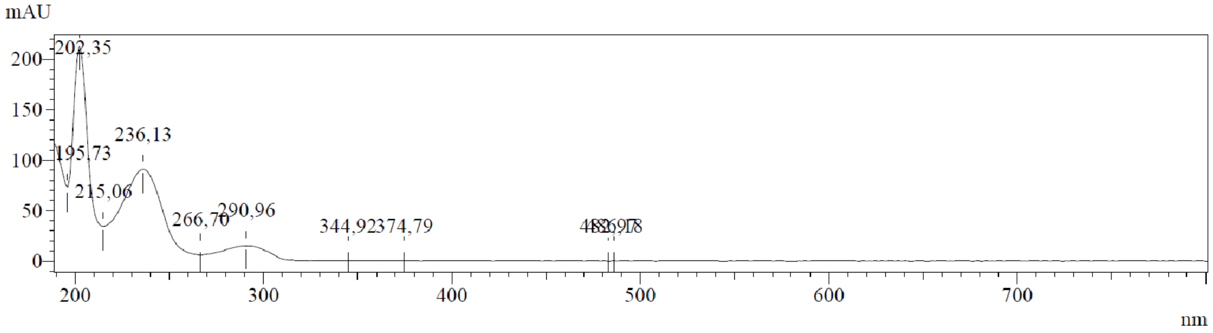

Retention Time : 31,457 min
Compound Name :
Spectrum Operation : None

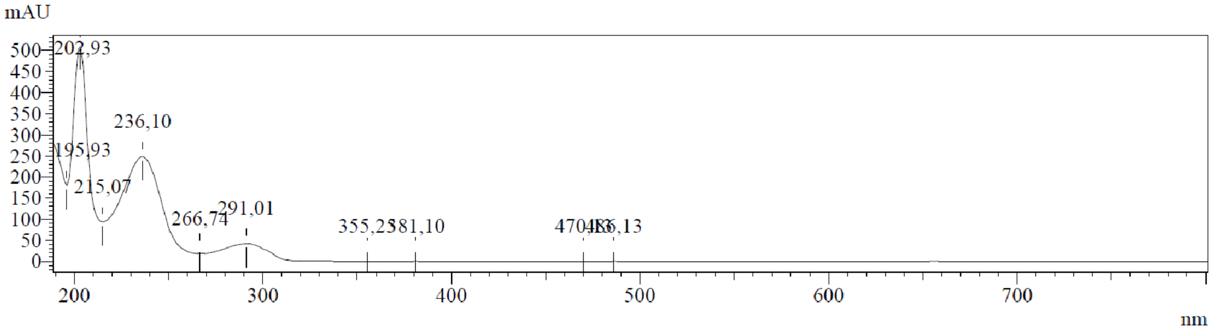

(*R*)-4-(octan-3-yl)aniline ((*R*)-2o)

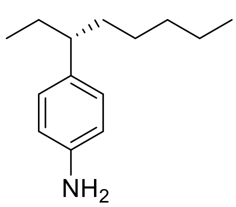

CuBr (1.4 mg, 0.01 mmol, 5.0 mol%) and ligand Taniaphos T001-2 (8.3 mg, 0.012 mmol, 6.0 mol%) were dissolved in dry DCM (1 mL) and Et_2O (1 mL). After 15 min stirring at room temperature, the solution was cooled down to -50 °C and the substrate (55.1 mg, 0.2 mmol, 1.0 equiv.) was added in 1mL of DCM. After stirring for an additional 10 min, pentyl MgBr (Aldrich, 2.0M solution in Et_2O, (0.3 mL, 0.6 mmol, 3.0 equiv.)) was added. The reaction mixture was stirred overnight (16-20h). The reaction is quenched with $NaHCO_3$ sat sol. (5 mL) and extracted with DCM (x3). The residue was purified by flash column chromatography in DCM to afford the corresponding aniline **2o** as a colourless liquid (27.1 mg, 0.142 mmol, 71%). The enantiomeric ratio was found to be 75:25 e.r. by analytical HPLC in OB-H column 99.5:0.5 Heptane: *i*PrOH, 0.5 mL/min, λ= 242 nm, tr (minor): 20.8 min, tr (major): 22.3 min.).

^{1}H NMR (400 MHz, $CDCl_3$) δ 6.98 (d, J = 8.2 Hz, 2H), 6.65 (d, J = 8.2 Hz, 2H), 3.38 (br s, 2H), 2.60 – 2.55 (m, 1H), 1.66 – 1.46 (m, 2H), 1.30 – 1.13 (m, 11H), 0.87(t, J = 7.0 Hz, 3H).

^{13}C NMR (151 MHz, $CDCl_3$) δ 144.2, 138.4, 127.8, 115.4, 39.2, 38.8, 32.0, 29.6, 27.8, 22.8, 22.7, 14.2.

HRMS (ESI-TOF): m/z $[M+H]^+$ Calcd. for $C_{14}H_{23}NH$ 206.1909, found 206.1903

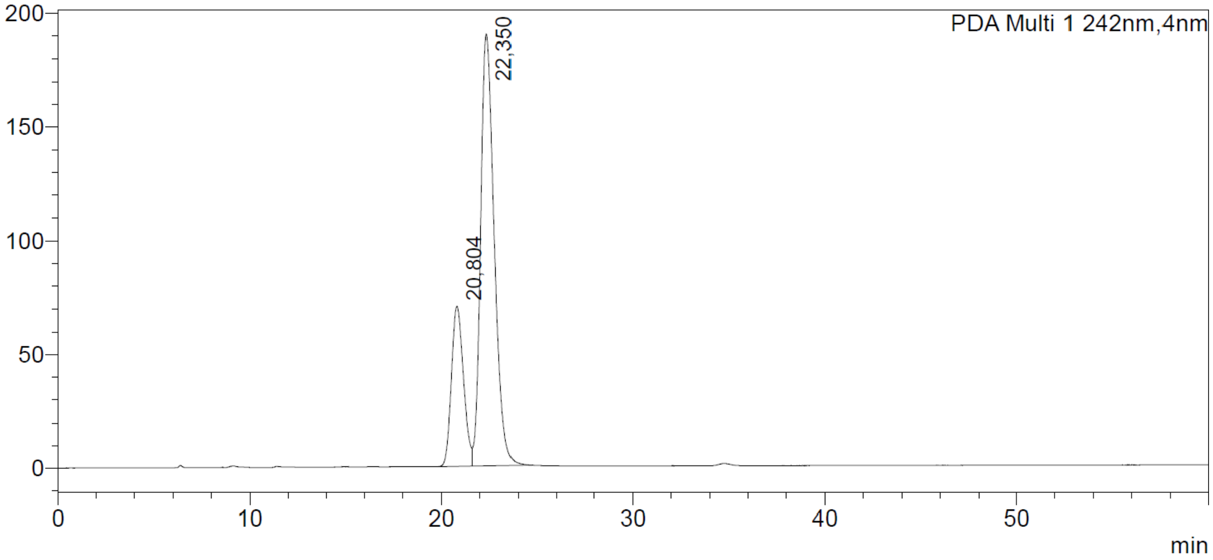

PDA Ch1 242nm

Peak#	Ret. Time	Area	Height	% Area
1	20,804	3109471	70297	24,787
2	22,350	9435262	189725	75,213
Total		12544733	260022	100,000

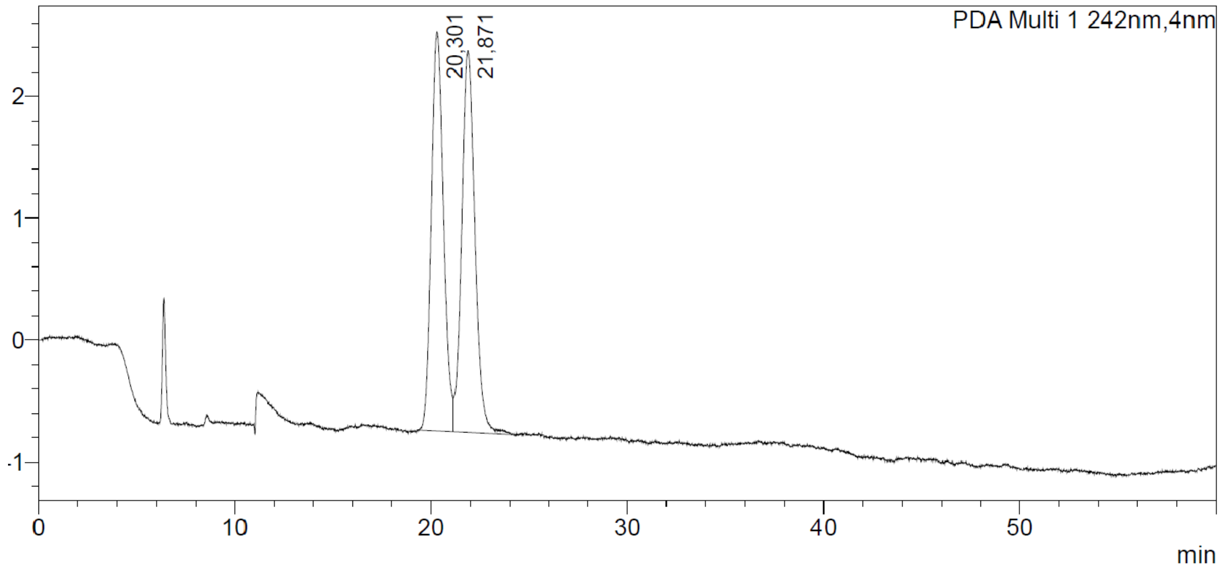

PDA Ch1 242nm

Peak#	Ret. Time	Area	Height	% Area
1	20,301	142072	3280	48,935
2	21,871	148253	3133	51,064
Total		290325	6413	100,000

Retention Time : 20,804 min
Compound Name :
Spectrum Operation : None

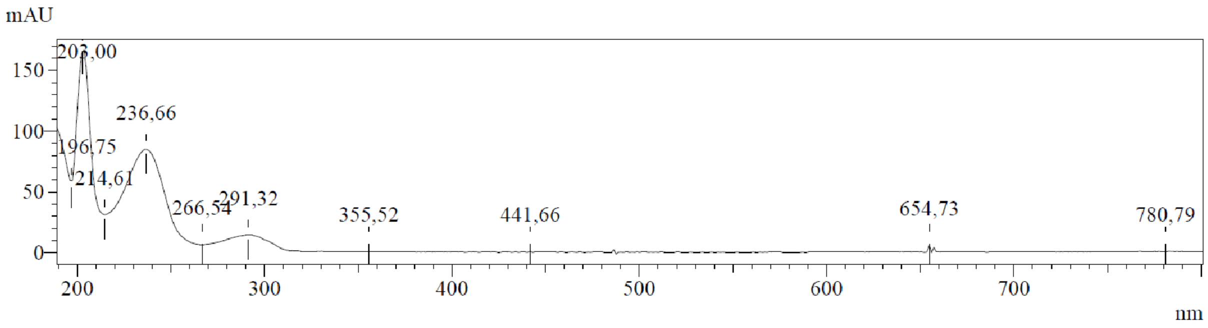

Retention Time : 22,350 min
Compound Name :
Spectrum Operation : None

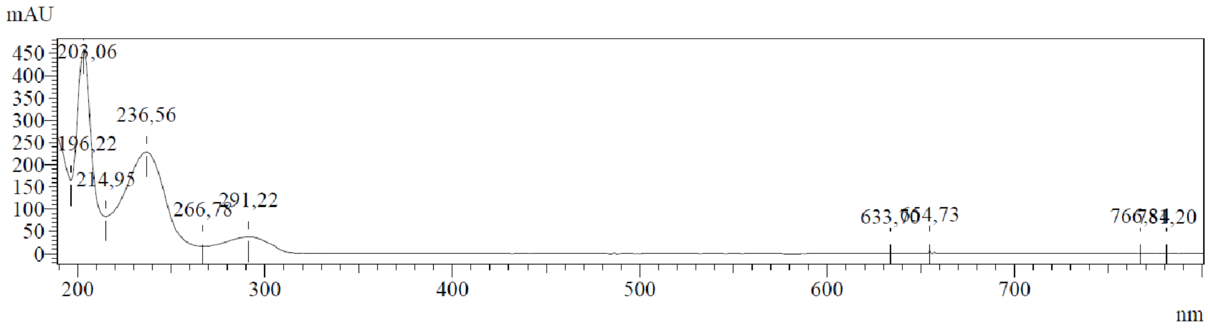

(*R*)-4-(5-methylhexan-2-yl)aniline ((*R*)-2e)

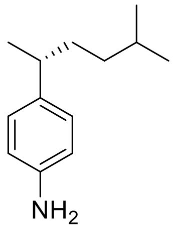

CuBr (1.4 mg, 0.01 mmol, 5.0 mol%) and ligand Taniaphos T001-2 (8.3 mg, 0.012 mmol, 6 mol%) were dissolved in dry DCM (1 mL) and Et_2O (1 mL). After 15 min stirring at room temperature, the solution was cooled down to -50 °C and the substrate (55.1 mg, 0.2 mmol, 1.0 equiv.) was added in 1mL of DCM. After stirring for an additional 10 min, Isopentyl MgBr (Aldrich, 2.0M solution in Et_2O, (0.3 mL, 3.0 equiv.)) was added. The reaction mixture was stirred overnight (16-20h). The reaction is quenched with $NaHCO_3$ sat sol. (5 mL) and extracted with DCM (x3). The residue was purified by flash column chromatography in DCM to afford the corresponding aniline **2e** as a colourless liquid (23.4 mg, 0.122 mmol, 61%). The enantiomeric ratio was found to be 39:61 e.r. by analytical HPLC in OB-H column 98:2 Heptane: *i*PrOH, 0.5 mL/min, λ= 226 nm, tr (*minor*): 19.6 min, tr (*major*): 24.4min.

^{1}H NMR (400 MHz, $CDCl_3$) δ 6.99 (d, J = 8.0 Hz, 2H), 6.64 (d, J = 8.0 Hz, 2H), 3.35 (br. s, 2H), 2.59 – 2.50 (m, 1H), 1.58 – 1.45 (m, 3H), 1.20 (d, J = 7.0 Hz, 3H), 1.18 – 0.96 (m, 2H), 0.85 (d, J = 3.0 Hz, 3H), 0.84 (d, J = 3.0 Hz, 3H).

^{13}C NMR (151 MHz, $CDCl_3$) δ 144.1, 138.5, 127.8, 115.4, 39.5, 37.2, 36.5, 28.3, 22.8, 22.7.

HRMS (ESI-TOF): m/z $[M+H]^+$ Calcd. for $C_{13}H_{21}NH$ 192.1752, found 192.1742.

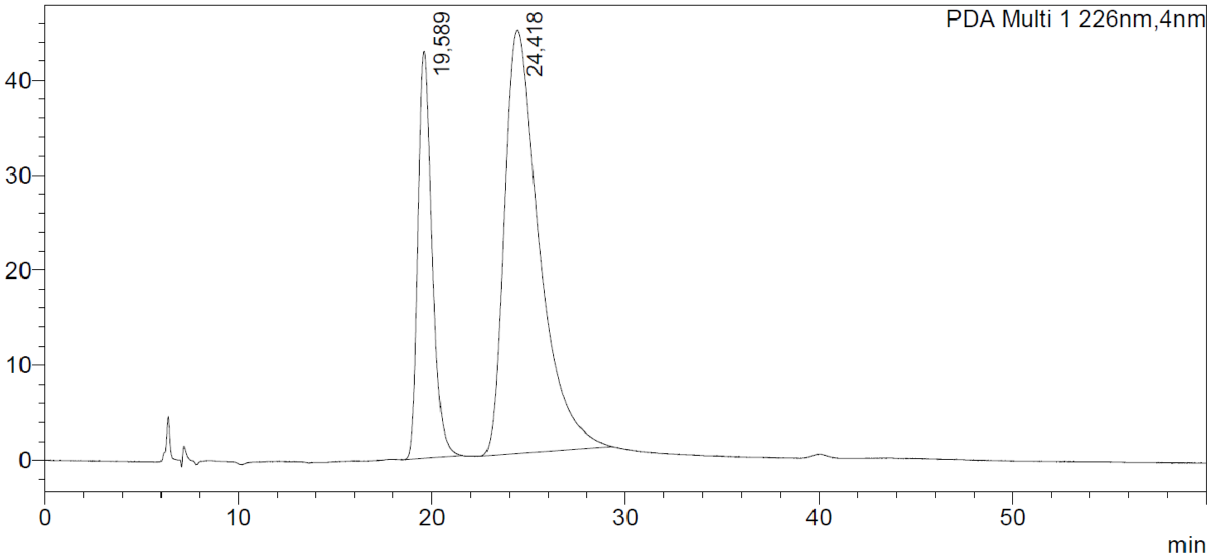

PDA Ch1 226nm

Peak#	Ret. Time	Area	Height	% Area
1	19,589	2192402	42803	39,191
2	24,418	5594132	44543	60,809
Total		7786534	87346	100,000

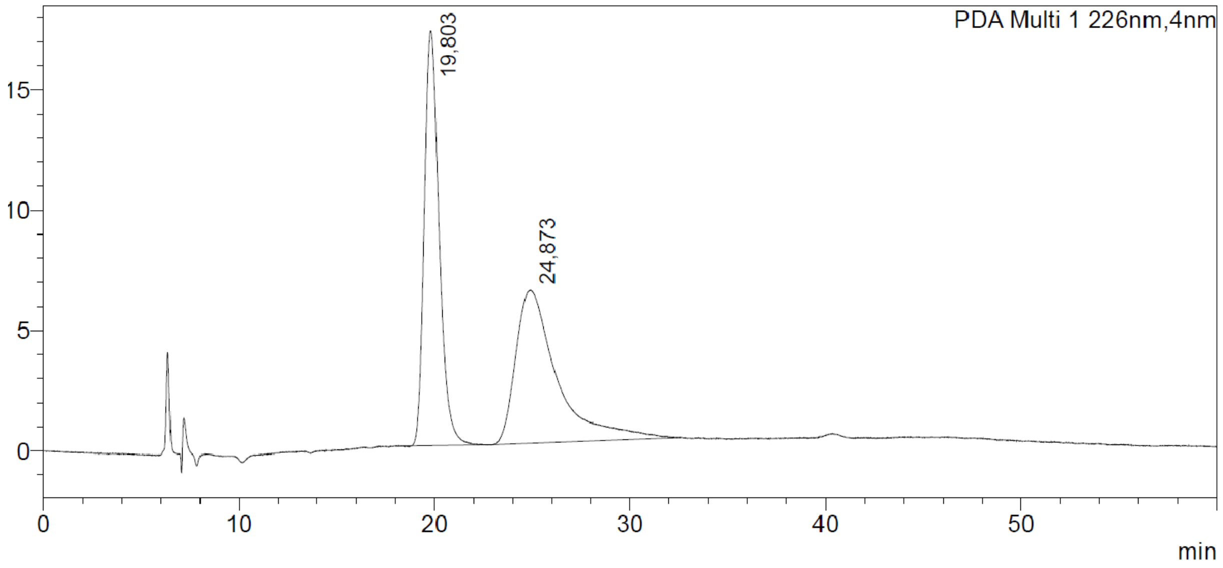

PDA Ch1 226nm

Peak#	Ret. Time	Area	Height
1	19,803	908015	17230
2	24,873	939576	6362
Total		1847590	23592

% Area
49,145
50,854
100,000

Retention Time : 19,803 min
Compound Name :
Spectrum Operation : None

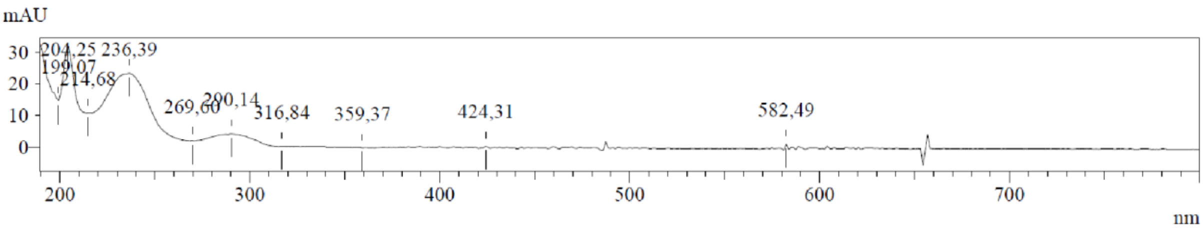

Retention Time : 24,873 min
Compound Name :
Spectrum Operation : None

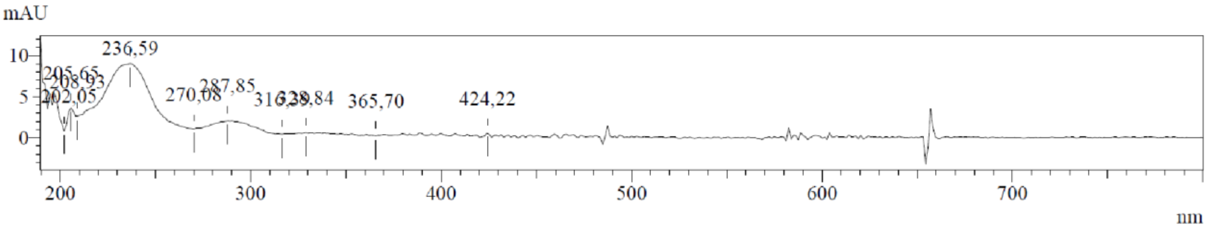

(*R*)-4-(1-cyclopentylethyl)aniline ((*R*)-2f)

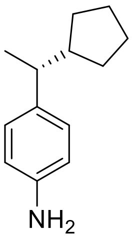

CuBr (1.4 mg, 0.01 mmol, 5.0 mol%) and ligand Taniaphos T001-2 (8.3 mg, 0.012 mmol, 6.0 mol%) were dissolved in dry DCM (1 mL) and Et_2O (1 mL). After 15 min stirring at room temperature, the solution was cooled down to -50 °C and the substrate (55.1 mg, 0.2 mmol, 1.0 equiv.) was added in 1mL of DCM. After stirring for an additional 10 min, cyclopentyl MgBr (Aldrich, 2.0M solution in Et_2O, (0.3 mL, 3.0 equiv.)) was added. The reaction mixture was stirred overnight (16-20h). The reaction is quenched with $NaHCO_3$ sat sol. (5 mL) and extracted with DCM (x3). The residue was purified by flash column chromatography in DCM to afford the corresponding aniline **2f** as a colourless liquid (20.1 mg,

0.106 mmol, 53%). The enantiomeric ratio was found to be 85:15 e.r. by analytical HPLC in OD-H column 98:2 Heptane: *i*PrOH, 1 mL/min, λ= 254 nm, tr (*major*): 15.2 min, tr (*minor*): 17.8 min.).

^{1}H NMR (600 MHz, $CDCl_3$) δ 6.97 (d, J = 8.3 Hz, 2H), 6.64 (d, J = 8.3 Hz, 2H), 3.36 (br s, 2H), 2.33 (dq, J = 9.4, 6.9 Hz, 1H), 1.91 – 1.85 (m, 2H), 1.65 – 1.39 (m, 5H), 1.22 – 1.17 (m, 4H), 1.06 – 0.91 (m, 1H).

^{13}C NMR (151 MHz, $CDCl_3$) δ 143.9, 138.7, 128.1, 115.4, 47.9, 45.4, 31.9, 31.5, 25.5, 25.2, 21.7.

HRMS (ESI-TOF): m/z $[M+H]^+$ Calcd. for $C_{13}H_{19}NH$ 190.1590, found 190.1591

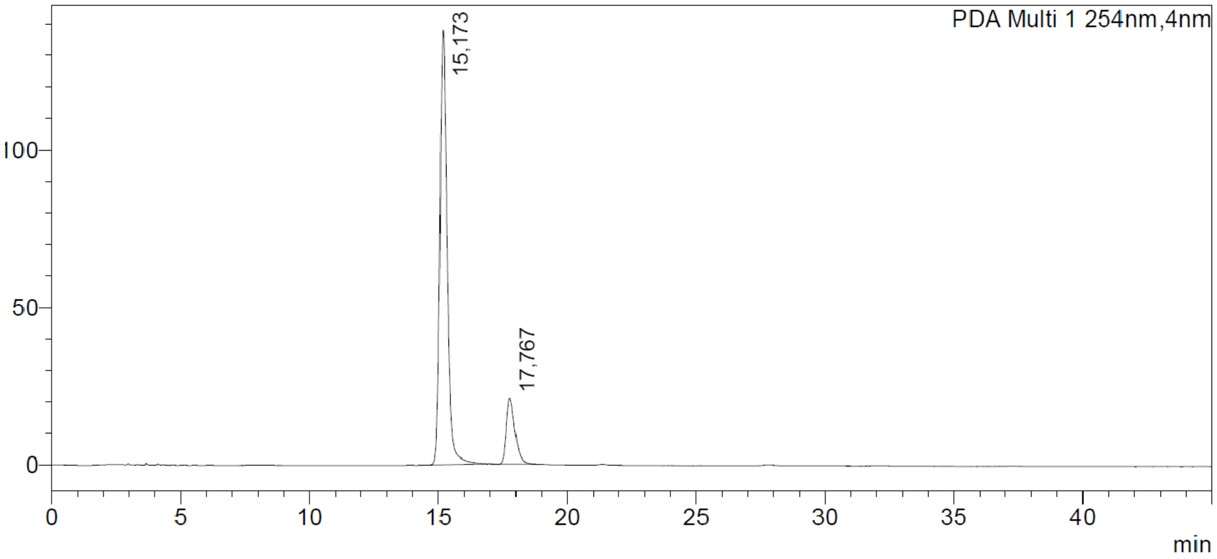

PDA Ch1 254nm

Peak#	Ret. Time	Area	Height
1	15,173	2623960	138001
2	17,767	478398	20836
Total		3102358	158837

% Area
15,420
84,580
100,000

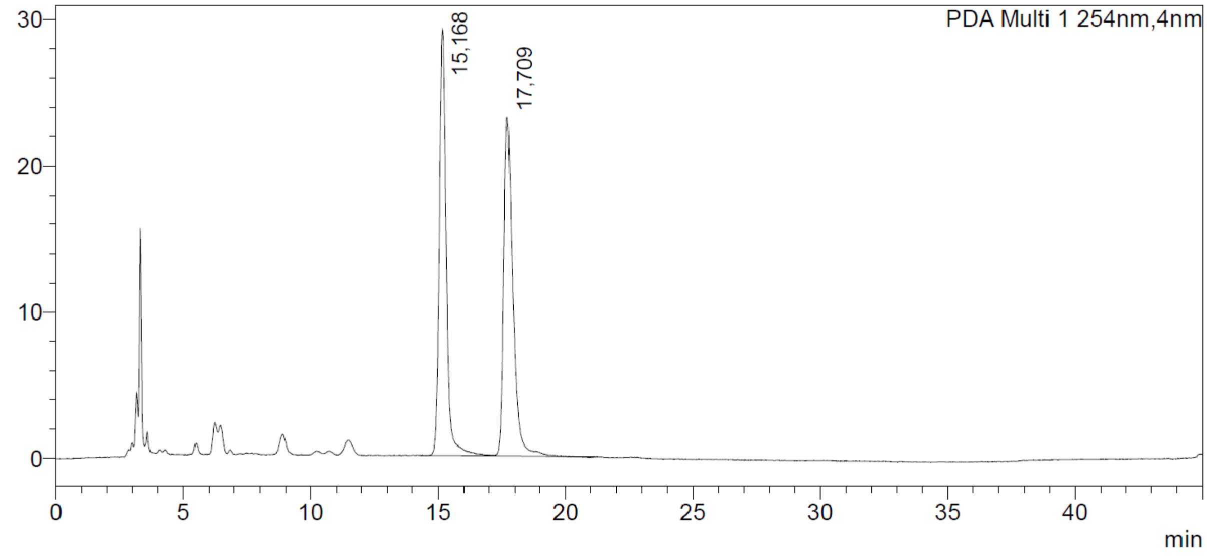

PDA Ch1 254nm

Peak#	Ret. Time	Area	Height	% Area
1	15,168	561432	29088	50,025
2	17,709	560851	23148	49,974
Total		1122283	52236	100,000

Retention Time : 15,173 min
Compound Name :
Spectrum Operation : None

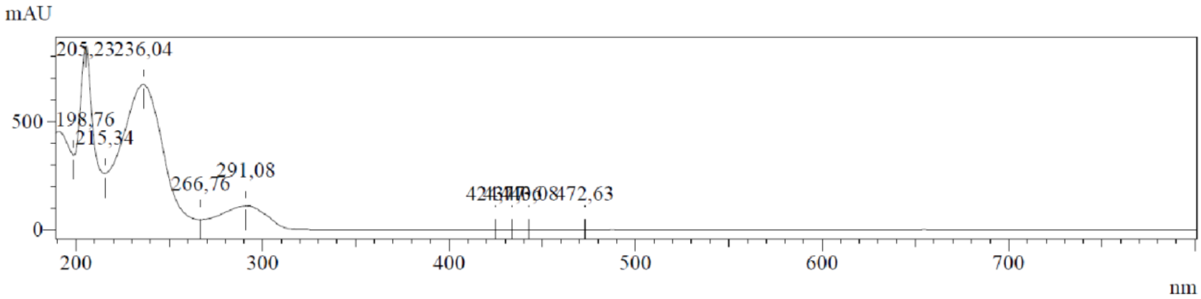

Retention Time : 17,767 min
Compound Name :
Spectrum Operation : None

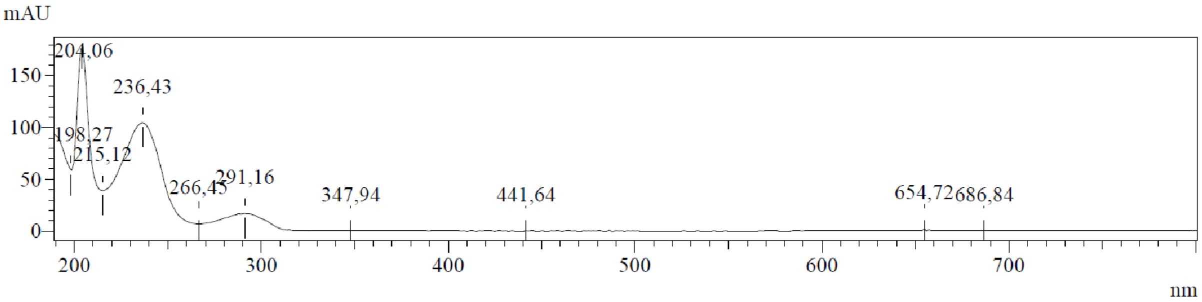

5. Derivatizations

(*R*)-N-(4-(sec-butyl)phenyl)-4-methylbenzenesulfonamide (3)

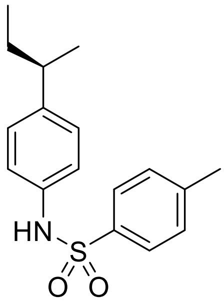

p-Toluenesulfonyl chloride (165.8 mg, 0.870 mmol, 1.1 equiv) is added to a solution of aniline (118.0 mg, 0.791 mmol, 1.0 equiv) in pyridine (3.2 mL, 39.534 mmol, 50.0 equiv) at 0 °C. The resulting solution is stirred at 25 °C overnight (24h). The solvent is removed in vacuo in order to remove the pyridine. The residue is dissolved in water and it is extracted with DCM (3x). The collected organic phases are dried over $MgSO_4$, filtered and the resulting residue is purified by silica gel column chromatography with pentane: ethyl acetate (7/3) as the eluent affording the desired sulfonamide **3** as a white solid (232.0 mg, 0.767 mmol, 97%). The enantiomeric ratio was found to be 87:13 e.r. by analytical HPLC in OD-H column 98:2 Heptane: *i*PrOH, 1 mL/min, λ= 227 nm, tr (*minor*): 40.6 min, tr (*major*): 43.1 min.).

^{1}H NMR (600 MHz, $CDCl_3$) δ 7.62 (d, J = 8.3 Hz, 2H), 7.24 – 7.16 (m, 2H), 7.04 (d, J = 8.4 Hz, 2H), 6.96 (d, J = 8.3 Hz, 2H), 6.43 – 6.39 (m, 1H), 2.55 – 2.49 (m, 1H), 2.38 (s, 3H), 1.62 – 1.42 (m, 2H), 1.17 (d, J = 6.9 Hz, 3H), 0.76 (t, J = 7.4 Hz, 3H).

^{13}C NMR (151 MHz, $CDCl_3$) δ 145.4, 143.8, 136.4, 134.1, 129.7, 128.0, 127.4, 122.5, 41.2, 31.3, 21.8, 21.7, 12.2.

HRMS (ESI-TOF): m/z $[M+H]^+$ Calcd. for $C_{17}H_{21}NO_2SH$ 304,1321, found 304.1322

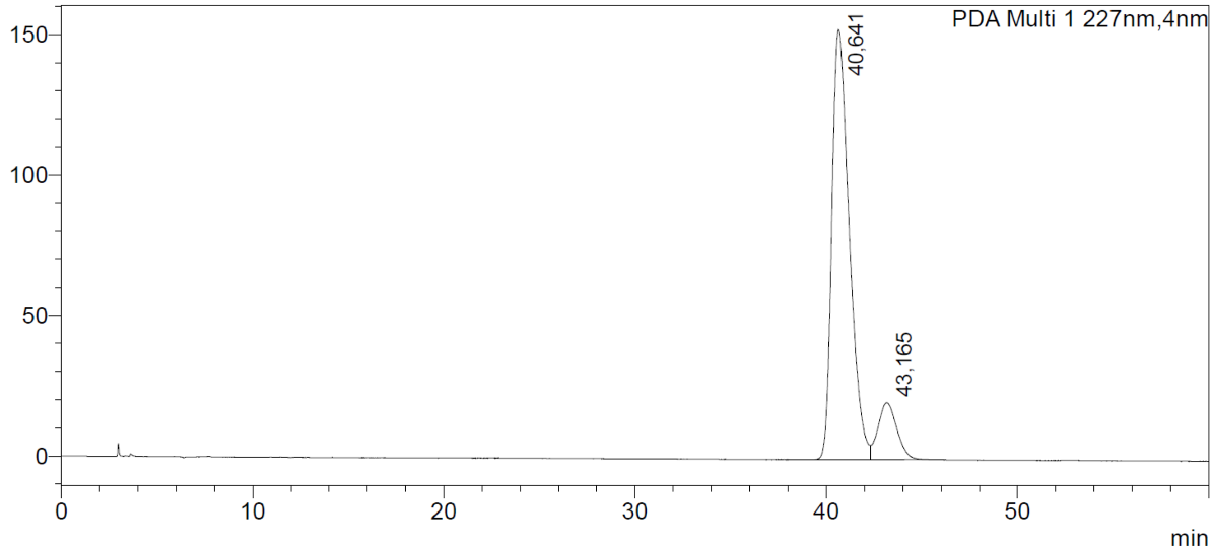

PDA Ch1 227nm

Peak#	Ret. Time	Area	Height	% Area
1	40,641	9694445	153082	87,366
2	43,165	1401873	20292	12,634
Total		11096318	173374	100,000

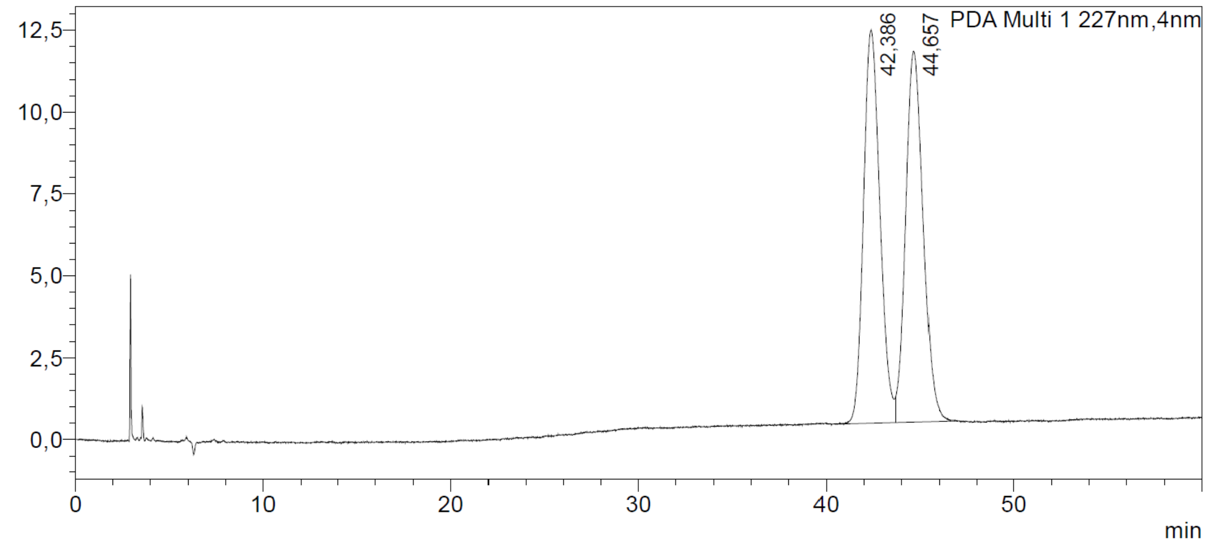

PDA Ch1 227nm

Peak#	Ret. Time	Area	Height	% Area
1	42,386	736086	11986	49,964
2	44,657	737120	11317	50,035
Total		1473206	23303	100,000

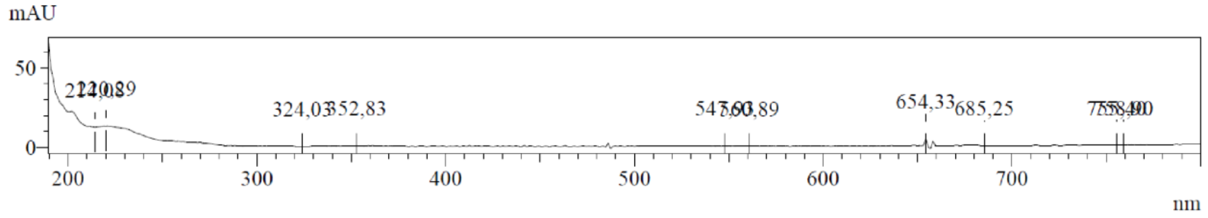

Retention Time : 44,657 min
Compound Name :
Spectrum Operation : None

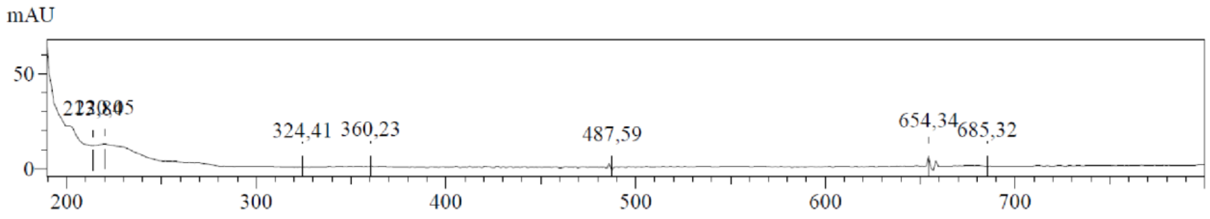

6. Synthesis of the substrates

6.1. Method A

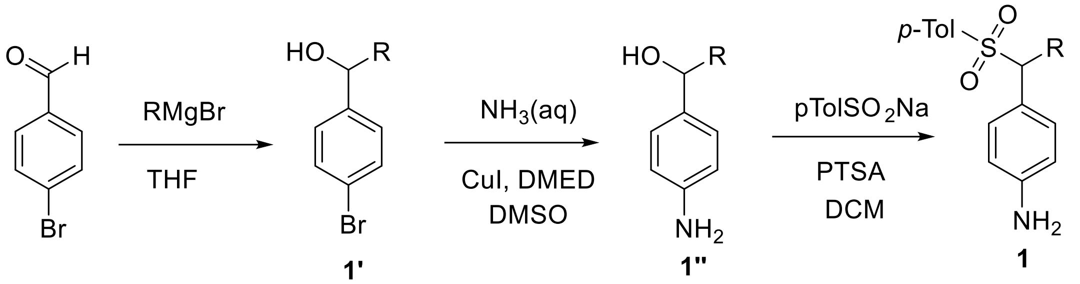

1' **1''** **1**

6.1.1. Synthesis of an alcohol derivative[7]

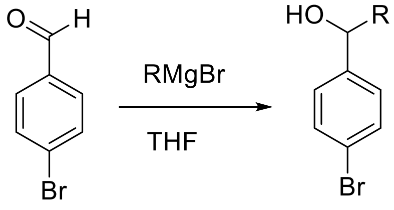

1'

General procedure: To a solution of 4-bromobenzaldehyde (1.0 equiv) in anhydrous THF (0.5 M) was added a solution of Grignard reagent (2.1 equiv) dropwise at room temperature. The resulting solution was stirred overnight, and the reaction mixture was quenched with saturated NH_4Cl aqueous solution (20 mL) and extracted with diethyl ether (x3). The combined organic layers were dried over anhydrous Na_2SO_4, then concentrated under vacuum to afford a crude, which was purified by column chromatography.

1-(4-bromophenyl)propan-1-ol (1'b)

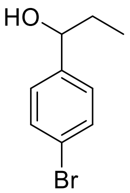

The product was synthesized following general procedure and it was obtained after flash column chromatography in silica gel (Pentane: EtOAc, 9:1) to afford a colourless liquid (2.15 g, 74%). The spectroscopic data matched with those reported in the literature.[8]

^{1}H NMR (400 MHz, $CDCl_3$) δ 7.44 (d, J = 8.4 Hz, 2H), 7.18 (d, J = 8.4 Hz, 2H), 4.53 (t, J = 6.5 Hz, 1H), 1.87 – 1.43 (m, 2H), 0.87 (t, J = 7.4 Hz, 3H).

^{13}C NMR (101 MHz, $CDCl_3$) δ 143.6, 131.6, 127.8, 121.3, 75.4, 32.0, 10.1.

1-(4-bromophenyl)butan-1-ol (1'n)

---

[7] It was not possible to obtain HRMS of those compounds. However, upon derivatization into the corresponding sulfones, the characterization was possible.
[8] Wei, D.; Roisnel, T.; Darcel, C.; Clot, E.; Sortais, J.-B.; *Chem. Cat. Chem.* **2017**, *9*, 80-83.

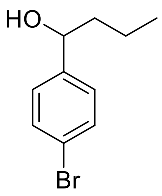

The product was synthesized following general procedure and it was obtained after flash column chromatography in silica gel (Pentane: EtOAc, 9:1) to afford the alcohol as a colourless liquid (1.01 g, 41%). The spectroscopic data matched with those reported in the literature.[9]

^{1}H NMR (400 MHz, $CDCl_3$) δ 7.46 (d, J = 8.3 Hz, 2H), 7.22 (d, J = 8.2 Hz, 2H), 4.77 – 4.43 (m, 1H), 1.84 – 1.54 (m, 3H), 1.51 – 1.14 (m, 2H), 0.93 (t, J = 7.4 Hz, 3H).

^{13}C NMR (151 MHz, CDCl3) δ 144.0, 131.6, 127.8, 121.3, 73.9, 41.4, 19.0, 14.0.

1-(4-bromophenyl)hexan-1-ol (1’o)

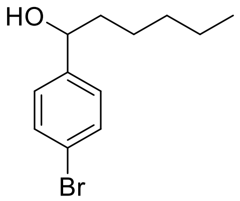

The product was synthesized following general procedure and it was obtained after flash column chromatography in silica gel (Pentane: EtOAc 20:1 - 9:1) as a white solid (0.55 g, 79%). The spectroscopic data matched with those reported in the literature. [10]

^{1}H NMR (400 MHz, $CDCl_3$) δ 7.46 (d, J = 8.4 Hz, 2H), 7.22 (d, J = 8.4 Hz, 2H), 4.63 (t, J = 6.5 Hz, 1H), 1.92 – 1.56 (m, 3H), 1.46 – 1.32 (m, 1H), 1.32 – 1.17 (m, 5H), 0.97 – 0.70 (m, 3H).

^{13}C NMR (101 MHz, $CDCl_3$) δ 146.6, 134.1, 130.3, 123.7, 76.5, 41.7, 34.3, 28.0, 25.2, 16.7.

1-(4-bromophenyl)-2-phenylethan-1-ol (1’p)

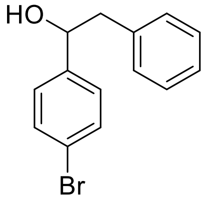

The product was synthesized following general procedure and it was obtained after flash column chromatography in silica gel (Pentane: EtOAc 9:1), affording the compound as a white solid (1.18 g, 39%). The spectroscopic data matched with those reported in the literature [11]

^{1}H NMR (400 MHz, $CDCl_3$) δ 7.56 – 7.36 (m, 2H), 7.30 (d, J = 7.4 Hz, 2H), 7.27 – 7.18 (m,3H), 7.19 – 7.08 (m, 2H), 4.87 (dd, J = 8.3, 5.1 Hz, 1H), 3.04 – 2.92 (m, 2H).

^{13}C NMR (101 MHz, $CDCl_3$) δ 145.4, 140.1, 134.1, 132.1, 131.2, 130.3, 129.4, 124.0, 77.3, 48.7.

1-(4-bromophenyl)-3-methylbutan-1-ol (1’q)

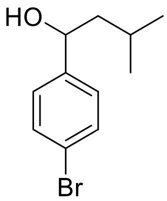

The product was synthesized following general procedure and it was obtained after flash column chromatography in silica gel (Pentane: EtOAc, 9:1) to afford a colourless liquid (0.59 g, 90%). The spectroscopic data matched with those reported in the literature.[8]

^{1}H NMR (400 MHz, $CDCl_3$) δ 7.44 (d, J = 8.3 Hz, 2H), 7.18 (d, J = 8.3 Hz, 2H), 4.66 (dd, J = 7.9, 5.3 Hz, 1H), 2.20 (br s, 1H), 1.82 – 1.54 (m, 2H), 1.50 – 1.40 (m, 1H), 0.93 (dd, J = 6.3, 2.0 Hz, 6H).

^{13}C NMR (151 MHz, $CDCl_3$) δ 144.3, 131.6, 127.7, 121.3, 72.2, 48.5, 24.9, 23.2, 22.3.

---

[9] Tanwar. L.; Börgel. J.; Ritter. T. *J. Am. Chem. Soc.* **2019**, *141*, 17983–17988.
[10] Patent: US2008/221220 A1, **2008**.
[11] Lovinger, G. J.; Aparece, M. D.; Morken, J. P. *J. Am. Chem. Soc.* **2017**, **139**, 3153–3160.

1-(4-bromophenyl)but-3-en-1-ol (1’r)

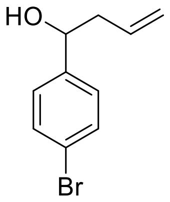

The product was synthesized following general procedure and it was obtained after flash column chromatography in silica gel (Pentane: EtOAc, 9:1), as a colourless liquid (2.19 g, 89%). The spectroscopic data matched with those reported in the literature.[12]

^{1}H NMR (400 MHz, $CDCl_3$) δ 7.44 (d, J = 8.4 Hz, 2H), 7.19 (dd, J = 8.5, 2.1 Hz, 2H), 5.92 – 5.60 (m, 1H), 5.24 – 4.98 (m, 2H), 4.68 – 4.63 (m, 1H), 2.56 – 2.30 (m, 2H), 2.18 (br s, 1H).

^{13}C NMR (75 MHz, $CDCl_3$) δ 175.4, 142.8, 133.9, 127.6, 121.2, 118.8, 72.6, 43.8.

1-(4-bromophenyl)-5-methoxypentan-1-ol (1’s)

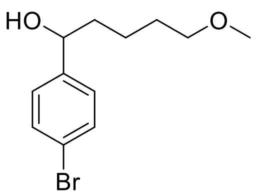

The product was synthesized following general procedure and using a solution of a freshly prepared Grignard reagent, it was obtained after flash column chromatography (Pentane: EtOAc, 9:1 --> 1:1), affording a yellow liquid (1.83 g, 96%).

^{1}H NMR (400 MHz, $CDCl_3$) δ 7.44 (d, J = 8.3 Hz, 2H), 7.19 (d, J = 8.3 Hz, 2H), 4.64 -4.60 (m, 1H), 3.35 – 3.32 (m, 2H), 3.29 (s, 3H), 1.87 (br s, 1H), 1.81 – 1.61 (m, 2H), 1.61 -1.51 (m, 2H), 1.51 – 1.24 (m, 2H).

^{13}C NMR (101 MHz, $CDCl_3$) δ 146.4, 134.1, 130.2, 123.9, 76.4, 75.2, 61.2, 41.5, 32.0, 25.0.

6.1.2. Synthesis of an amino sulfone derivative

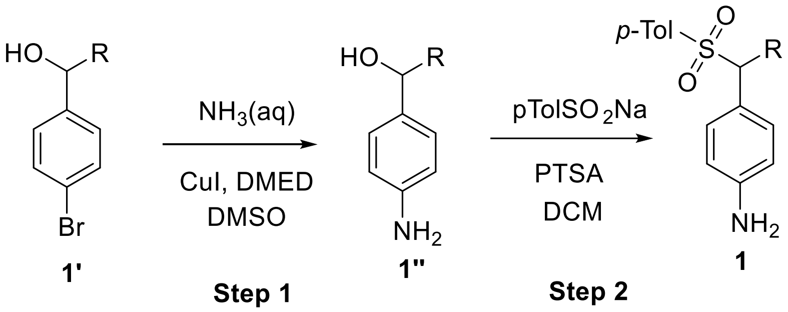

General procedure

Step 1: A 20 mL-screw capped vial was charged with aryl bromide **1’** (1.0 equiv), CuI (0.1 equiv) and DMEDA (1.5 equiv) in NH_3 (25 equiv. or completely full vial) and DMSO (2.5 mL). The vial was flushed with nitrogen and capped. The solution was stirred overnight at 130 °C. The resulting intense blue suspension was cooled down to room temperature, extracted with EtOAc (× 3). The organic layers were separated, and the organic layer is dried over Na_2SO_4, filtered, and concentrated. The residue was purified by flash column chromatography (Hexane/EtOAc =7/3-1/1) affording an aniline substrate **1’’** which was directly employed in the next step (decomposes easily).

Step 2: To a Schlenk under nitrogen, it was added a mixture of sodium p-toluensulfinate (1.0 equiv) and p-toluenesulfonic acid monohydrate (1.5 equiv) and dry DCM (0.5 M). Then, the aniline substrate **1’’**

[12] Yanagisawa, A.; Kageyama, H.; Nakatsuka, Y.; Asakawa, K.; Matsumoto, Y.; Yamamoto, H. *Angew. Chem., Int. Ed.* **1998**, *38*, 3701-3703.

was added to the mixture, and a precipitate was formed immediately. The resulting suspension was stirred either 2h or overnight, then quenched by adjusting to pH 8 with saturated Na_2CO_3 aqueous solution. The organic layer was separated, and the aqueous layer was extracted with DCM (× 3). The combined organic layers were dried over anhydrous Na_2SO_4, concentrated under vacuum to afford the corresponding sulfone **1**. The solid was purified by trituration with diethyl ether and hexane.

4-(1-tosylpropyl)aniline (1b)

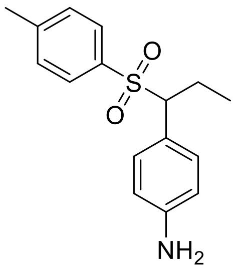

Step 1: The product was synthesized following general procedure and it was obtained after flash column chromatography in silica gel (hexane/EtOAc =7/3-1/1) to afford the corresponding aniline as a colourless oil **1''b** (0.96 g, 68%).

^{1}H NMR (400 MHz, $CDCl_3$) δ 7.12 (d, J = 8.4 Hz, 2H), 6.66 (d, J = 8.4 Hz, 2H), 4.47 (t, J = 6.7 Hz, 1H), 1.90 – 1.56 (m, 3H), 0.88 (t, J = 7.4 Hz, 3H).

Step 2: The product was synthesized following general procedure obtaining a solid that was purified by trituration with pentane and diethyl ether obtaining a white-yellow solid **1b** (0.65 g, 48%).

^{1}H NMR (400 MHz, $CDCl_3$) δ 7.42 (d, J = 8.1 Hz, 2H), 7.18 (d, J = 8.0 Hz, 2H), 6.89 (d, J = 8.1 Hz, 2H), 6.59 (d, J = 8.3 Hz, 2H), 4.32 (br s, 2H), 3.82 (dd, J = 11.7, 3.7 Hz, 1H), 2.43 – 2.36 (m, 4H), 2.08 – 2.00 (m,1H), 0.84 (t, J = 7.4 Hz, 3H).

^{13}C NMR (151 MHz, $CDCl_3$) δ 146.9, 144.2, 134.9, 131.1, 129.3, 129.3, 121.5, 115.0, 72.8, 21.7, 21.0, 11.6.

HRMS (ESI-TOF): m/z $[M+H]^+$ Calcd. for $C_{16}H_{19}NO_2SH$ 290.1247, found 290.1209

4-(1-tosylbutyl)aniline (1n)

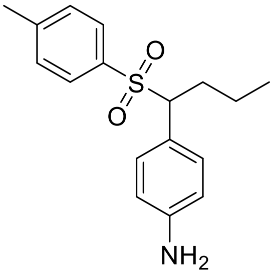

Step 1: The product was synthesized following general procedure and it was obtained after flash column chromatography (hexane/EtOAc =7/3-1/1) to afford the corresponding aniline **1''n** as a colourless oil (0.37 g, 73%).

^{1}H NMR (400 MHz, $CDCl_3$) δ 7.13 (d, J = 8.3 Hz, 2H), 6.66 (d, J = 8.4 Hz, 2H), 4.56 (t, J = 6.8 Hz, 1H), 1.85 – 1.70 (m, 1H), 1.70 – 1.54 (m, 1H), 1.47 – 1.34 (m, 1H), 1.30 – 1.20 (m, 1H), 1.26 (t, J = 7.1 Hz, 1H), 0.91 (t, J = 7.4 Hz, 3H).

Step 2: The product was synthesized following general procedure to afford a solid which was purified by trituration with $CHCl_3$ and pentane to yield a yellow solid **1n** (0.22 g, 34%).

^{1}H NMR (600 MHz, $CDCl_3$) δ 7.41 (d, J = 8.1 Hz, 2H), 7.17 (d, J = 8.0 Hz, 2H), 6.86 (d, J = 8.1 Hz, 2H), 6.53 (d, J = 8.1 Hz, 2H), 3.91 (dd, J = 11.7, 3.6 Hz, 1H), 3.65 (br s, 2H), 2.38 (s, 3H), 2.31 – 2.25 (m, 1H), 2.06 – 2.00 (m, 1H), 1.30 – 1.12 (m, 2H), 0.86 (t, J = 7.3 Hz, 3H, CH3).

^{13}C NMR (151 MHz, $CDCl_3$) δ 146.9, 144.2, 134.8, 131.0, 129.3, 129.2, 121.8, 115.0, 71.0, 29.4, 21.7, 20.1, 13.7.

HRMS (ESI-TOF): m/z $[M+Na]^+$ Calcd. for $C_{17}H_{21}NO_2SNa$ 326.1191, found 326.1189.

4-(1-tosylhexyl)aniline (1o)

Step 1: The product was synthesized following general procedure and it was obtained after flash column chromatography (hexane/EtOAc =7/3-1/1), to afford the corresponding aniline **1''o** (1.05 g, 88%).

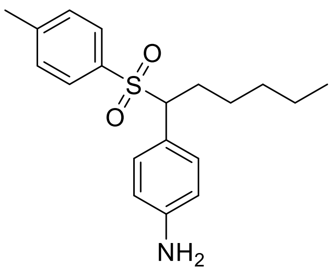

^1H NMR (400 MHz, $CDCl_3$) δ 7.13 (d, J = 8.2 Hz, 2H), 6.67 (d, J = 8.3 Hz, 2H), 4.54 (t, J = 6.9 Hz, 1H), 1.85 – 1.71 (m, 1H), 1.71 – 1.58 (m, 2H), 1.44 – 1.12 (m, 8H), 0.86 (t, J = 5.7 Hz, 3H).

Step 2: The product was synthesized following general procedure to afford a solid that was purified by trituration with pentane and diethyl ether afforded a pure sulfone as a pale yellow solid **1o** (0.06 g, 5%).

^1H NMR (400 MHz, $CDCl_3$) δ 7.41 (d, J = 8.0 Hz, 2H), 7.17 (d, J = 8.0 Hz, 2H), 6.88 (d, J = 8.1 Hz, 2H), 6.60 (d, J = 8.1 Hz, 2H), 4.34 (br s, 2H), 3.90 (dd, J = 11.7, 3.6 Hz, 1H), 2.39 (s, 3H), 2.36 – 2.28 (m, 1H), 2.08 – 1.98 (m, 1H), 1.32 – 0.96 (m, 6H), 0.83 – 0.79 (m, 3H).

^{13}C NMR (101 MHz, $CDCl_3$) δ 146.8, 144.2, 134.8, 131.0, 129.3, 129.2, 121.7, 115.0, 72. 4, 71.1, 58.6, 29.3, 27.2, 23.6, 21.7.

HRMS (ESI-TOF): m/z $[M+H]^+$ Calcd. for $C_{19}H_{25}NO_2SH$ 332.1684, found 332.1678

4-(2-phenyl-1-tosylethyl)aniline (1p)

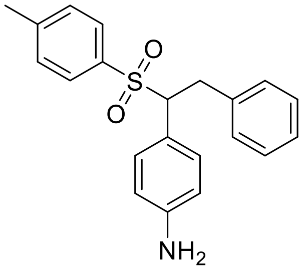

Step 1: The product was synthesized following general procedure and it was obtained after flash column chromatography (hexane/EtOAc =7/3-1/1) to yield the aniline **1''p** (0.39 g, 51%)

^1H NMR (400 MHz, $CDCl_3$) δ 7.31 – 7.14 (m, 7 H), 6. 67 – 6.65 (m, 2H), 4.79 (t, J = 6.7 Hz, 1H), 3.00 (d, J = 6.7 Hz, 2H).

Step 2: The product was synthesized following general procedure to afford the corresponding sulfone after trituration with pentane and diethyl ether as a yellow solid **1p** (0.23 g, 26%).

^1H NMR (400 MHz, DMSO-d^6) δ 7.52 (d, J = 8.3 Hz), 7.32 (d, J = 8.3 Hz), 7.21 – 6.96 (m, 5H), 6.78 (d, J = 8.5 Hz, 2H), 6.31 (d, J = 8.6 Hz, 2H), 5.08 (s, 2H), 4.58 (dd, J = 12.1, 3.5 Hz, 1H), 3.39 (dd, J = 14.0, 3.5 Hz, 1H), 3.28 – 3.17 (m, 1H), 2.35 (s, 3H).

^{13}C NMR (101 MHz, DMSO-d^6) δ 149.2, 144.4, 137.7, 135.3, 131.3, 129.8, 129.4, 129.1, 128.6, 126.6, 117.8, 113.7, 68.2, 32.3, 19.9.

HRMS (ESI-TOF): m/z $[M+K]^+$ Calcd. for $C_{21}H_{21}NO_2SK$ 389.1399, found 389.1332

4-(3-methyl-1-tosylbutyl)aniline (1q)

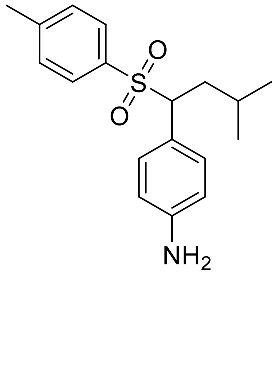

Step 1: The product was synthesized following general procedure and it was obtained after flash column chromatography (hexane/EtOAc =7/3-1/1) to afford the corresponding aniline **1''q** (0.35 g, 67%).

^1H NMR (400 MHz, $CDCl_3$) δ 7.13 (d, J = 8.3 Hz, 2H), 6.65 (d, J = 8.3 Hz, 2H), 4.62 (dd, J = 7.9, 6.0 Hz, 1H), 2.91 (br s, 2H), 1.74 - 1.59 (m, 2H), 1.54 – 1.51 (m, 1H, CH), 0.92 (dd, J = 6.4, 4.5 Hz, 6H).

Step 2: The product was synthesized following general procedure to afford a solid that was purified by trituration with diethyl ether and pentane to afford a white-yellow solid **1q** (0.53 g, 87%).

^{1}H NMR (600 MHz, $CDCl_3$) δ 7.33 (d, J = 8.3 Hz, 2H), 7.10 (d, J = 8.0 Hz, 2H), 6.78 (d, J = 8.0 Hz, 2H), 6.46 (d, J = 8.6 Hz, 2H), 3.91 (dd, J = 11.8, 3.9 Hz, 1H), 3.57 (br s, 2H), 2.31 (s, 3H), 2.15 – 1.64 (m, 2H), 2.05 – 1.93 (m, 1H), 0.81 (d, J = 6.6 Hz, 3H), 0.71 (d, J = 6.6 Hz, 3H).

^{13}C NMR (151 MHz, $CDCl_3$) δ 146.8, 144.1, 134.8, 131.0, 129.2, 129.2, 121.8, 115.0, 69.7, 35.7, 25.3, 23.7, 21.7, 20.9.

HRMS (ESI-TOF): m/z $[M+Na]^+$ Calcd. for $C_{18}H_{23}NO_2SNa$ 340.1347, found 340.1342.

4-(1-tosylbut-3-en-1-yl)aniline (1r)

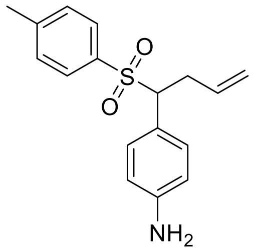

Step 1: The product was synthesized following general procedure and it was obtained after flash column chromatography (hexane/EtOAc =7/3-1/1) to afford the corresponding aniline **1''r** (1.17 g, 81%).

^{1}H NMR (400 MHz, $CDCl_3$) δ 7.14 (d, J = 8.3 Hz, 2H), 6.66 (d, J = 8.3 Hz, 2H), 5.79 (ddt, J = 17.2, 10.1, 7.1 Hz, 2H), 5.20 – 5.03 (m, 1H), 4.61 (t, J = 6.6 Hz, 1H), 2.99 (br s, 2H), 2.48 (t, J = 7.1 Hz, 2H).

Step 2: The product was synthesized following general procedure to afford a solid which was purified by trituration with pentane and diethyl ether to afford the corresponding sulfone as a white-beige solid **1r** (0.85g, 39%).

^{1}H NMR (400 MHz, $CDCl_3$) δ 7.46 – 7.37 (d, J = 8.1 Hz, 2H), 7.18 (d, J = 7.9 Hz, 2H), 6.87 (d, J = 7.4 Hz, 2H), 6.54 (d, J = 7.4 Hz, 2H), 5.63 – 5.41 (m, 1H), 5.04 (d, J = 17.0 Hz, 1H), 4.95 (d, J = 9.9 Hz, 1H), 3.98 (dd, J = 11.5, 3.6 Hz, 1H), 3.12 – 3.09 (m, 1H), 2.89 – 2.70 (m, 1H), 2.39 (s, 3H).

^{13}C NMR (101 MHz, $CDCl_3$) δ 146.9, 137.0, 136.0, 136.0, 133.7, 131.9, 131.9, 131.8, 120.7, 117.6, 73.1, 34.5, 24.2.

HRMS (ESI-TOF): m/z $[M+NH_4]^+$ Calcd. for $C_{17}H_{19}NO_2SNH_4$ 319.1480, found 319.1475.

4-(5-methoxy-1-tosylpentyl)aniline (1s)

Step 1: The product was synthesized following general procedure and it was obtained after flash column chromatography (hexane/EtOAc =7/3-1/1) to afford the aniline **1''s** as a yellow liquid (1.13 g, 99%).

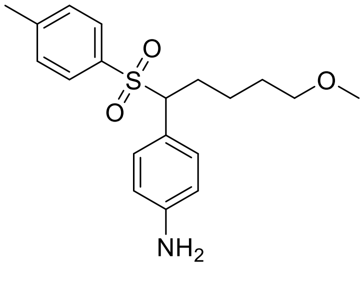

^{1}H NMR (400 MHz, $CDCl_3$) δ 7.09 (d, J = 8.2 Hz, 2H), 6.61 (d, J = 8.2 Hz, 2H), 4.49 (t, J = 6.8 Hz, 1H), 3.32 (t, J = 6.6 Hz, 2H), 3.27 (s, 3H), 3.13 (br s, 2H),1.81 – 1.71 (m, 1H), 1.70 – 1.60 (m, 1H), 1.55 (q, J = 7.0 Hz, 2H), 1.51 – 1.33 (m, 1H), 1.33 – 1.24 (m, 1H).

Step 2: The product was synthesized following general procedure to afford after trituration with diethyl ether and pentane the corresponding sulfone as a yellow solid **1s** (0.87 g, 53%).

^{1}H NMR (400 MHz, $CDCl_3$) δ 7.44 – 7.34 (m, 2H), 7.17 (dd, J = 8.2, 1.7 Hz, 2H), 6.85 (dd, J = 8.5, 2.1 Hz, 2H), 6.53 (dd, J = 8.6, 2.6 Hz, 2H), 3.89 (dd, J = 11.7, 3.6 Hz, 1H), 3.56 (br s, 2H), 3.29–3.25 (m,

2H), 3.24 (s, 3H), 2.37 (s, 3H), 2.58 – 2.17 (m, 1H), 2.15 – 1.90 (m, 1H), 1.72 – 1.34 (m, 2H), 1.34 – 1.04 (m, 2H).

^{13}C NMR (101 MHz, $CDCl_3$) δ 146.8, 144.2, 134.8, 131.0, 129.3, 129.2, 121.7, 115.1, 72.4, 71.1, 58.6, 29.3, 27.2, 23.6, 21.7.

HRMS (ESI-TOF): m/z $[M+Na]^+$ Calcd. for $C_{19}H_{25}NO_3SNa$ 370.1453, found 370.1446

6.2. Method B

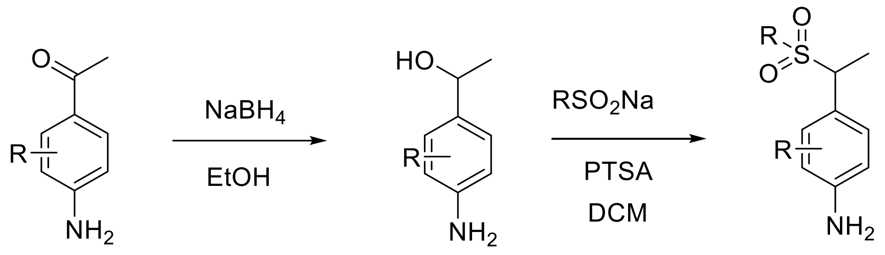

1" 1

6.2.1. Synthesis of alcohols

General Procedure

To a previously dried Schlenk flask, it is added the corresponding ketone (1.0 equiv) and it is dissolved in EtOH (0.2 M). Next, $NaBH_4$ (2.0 equiv) was added and the schlenk is open for 15 minutes, to release the overpressure due to the formation hydrogen gas and then closed with a septum. The resulting mixture was stirred at r.t. overnight. Then, the solvent was removed in vacuo, dissolved in EtOAc and water. extracted with EtOAc (3 x) and the collected organic phases were dried over Na_2SO_4, filtered affording the alcohol **1’’**.

1-(4-aminophenyl)ethan-1-ol (1’’a)

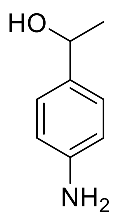

The product was synthesized following general procedure to afford the alcohol **1’’a** as a white solid (7.0 g, 98%). The spectroscopic data matched with those reported in the literature.[13]

^1H NMR (400 MHz, $CDCl_3$) δ 7.17 (d, J = 8.3 Hz, 2H), 6.67 (d, J = 8.4 Hz, 2H), 4.80 (q, J = 6.4 Hz, 1H), 3.65 (br s, 2H), 1.46 (d, J = 6.4 Hz, 3H)

^{13}C NMR (151 MHz, $CDCl_3$) δ 146.0, 136.0, 126.8, 115.2, 70.3, 24.9.

HRMS (ESI-TOF): m/z $[M+H]^+$ Calcd. for $C_8H_{11}NOH$ 138.0919, found 138.0913

1-(4-amino-3,5-dichlorophenyl)ethan-1-ol (1’’t)

[13] Li, Z.; Yazaki, R.; Ohshima, T. *Org. Lett.,* **2016**, *18*, 3350–3353.

The product was synthesized following general procedure to afford the alcohol as a grey solid **1''t** (2.46 g, 97%).

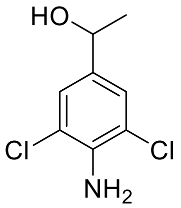

^{1}H NMR (400 MHz, $CDCl_3$) δ 7.20 (s, 2H), 4.75 (q, J = 6.4 Hz, 1H), 1.44 (d, J = 6.4 Hz, 3H).

^{13}C NMR (151 MHz, $CDCl_3$) δ 146.0, 136.0, 126.8, 115.2, 70.3, 24.9.

HRMS (ESI-TOF): m/z $[M+H]^+$ Calcd. for $C_8H_9Cl_2NOH$ 206.0139, found 206.0134

6.2.2. Synthesis of sulfones

General Procedure

To a Schlenk under nitrogen, it was added a mixture of sodium sulfinate (1.0 equiv) and p-toluenesulfonic acid monohydrate (1.5 equiv) and dry DCM (0.5 M). Then, the aniline substrate 1' was added to the mixture, and a precipitate was formed immediately. The resulting suspension was stirred either 2h or overnight, then quenched by adjusting to pH 8 with saturated Na_2CO_3 aqueous solution. The organic layer was separated, and the aqueous layer was extracted with DCM (× 3). The combined organic layers were dried over anhydrous Na_2SO_4, concentrated under vacuum to afford the corresponding sulfone **1**. The solid was purified by trituration with diethyl ether and hexane.

4-(1-tosylethyl)aniline (1a)

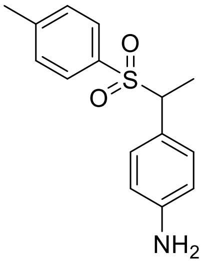

The product was synthesized following general procedure to afford a solid that was purified by trituration with diethyl ether and pentane to afford a white solid **1a** (1.2 g, 40%). The spectroscopic data matched with those reported in the literature.[14]

^{1}H NMR (600 MHz, $CDCl_3$) δ 7.44 (d, J = 8.3 Hz, 2H), 7.20 (d, J = 8.3 Hz, 2H), 6.92 (d, J = 8.4 Hz, 2H), 6.57 (d, J = 8.4 Hz, 2H), 4.12 (q, J = 7.2 Hz, 1H), 3.99 (br s, 2H), 2.40 (s, 3H), 1.69 (d, J = 7.2 Hz, 3H).

^{13}C NMR (151 MHz, $CDCl_3$) δ 146.5, 144.3, 134.3, 130.6, 129.4, 129.3, 123.67, 115.1, 65.6, 21.8, 14.3.

HRMS (ESI-TOF): m/z $[M+H]^+$ Calcd. for $C_{15}H_{17}NO_2SH$ 276.1058, found 276.1053

4-(1-(mesitylsulfonyl)ethyl)aniline (1aa)

The product was synthesized following general procedure to afford a solid that was washed with pentane to afford the corresponding sulfone 1aa as a white solid (1.61 g, 82%).

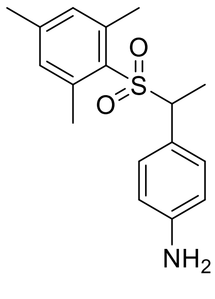

^{1}H NMR (600 MHz, $CDCl_3$) δ 6.90 – 6.86 (m, 2H), 6.84 (s, 2H), 6.56 – 6.46 (m, 2H), 4.18 (q, J = 7.2 Hz, 1H), 3.59 (br s, 2H), 2.33 (s, 6H), 2.27 (s, 3H), 1.75 (d, J = 7.2 Hz, 3H).

^{13}C NMR (151 MHz, $CDCl_3$) δ 147.0, 143.0, 140.9, 131.9, 131.8, 130.7, 123.1, 114.7, 64.6, 22.9, 21.1, 12.8.

HRMS (ESI-TOF): m/z $[M+H]^+$ Calcd. for $C_{17}H_{21}NO_2SH$ 304.1371, found 304.1358

[14] Yang, D. Y.; Han, O. S.; Liu, H. W. *J. Org. Chem.* **1989,** *54*, 5402-5406.

4-(1-(methylsulfonyl)ethyl)aniline (1ab)

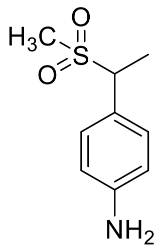

The product was synthesized following general procedure to afford the corresponding sulfone **1ab** as a white solid after trituration with diethyl ether (2.58 g, 89%).

^1H NMR (600 MHz, $CDCl_3$) δ 7.18 (d, J = 8.0 Hz, 2H), 6.66 (d, J = 8.0 Hz, 2H), 4.07 (q, J = 7.2 Hz, 1H), 3.75 (br s, 2H), 2.61 (s, 3H), 1.72 (d, J = 7.2 Hz, 3H).

^{13}C NMR (151 MHz, $CDCl_3$) δ 147.4, 130.0, 123.6, 115.3, 64.4, 37.6, 13.5.

HRMS (ESI-TOF): m/z [M+H]+ Calcd. for $C_9H_{13}NO_2SH$ 200.0745, found 200.0740

2,6-dichloro-4-(1-tosylethyl)aniline (1t)

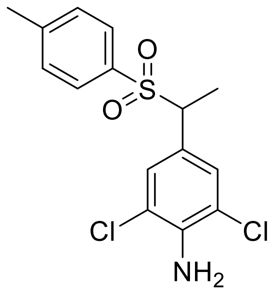

The product was synthesized following general procedure to afford the corresponding sulfone **1t** after trituration with diethyl ether and pentane (2.54 g, 76%) as a white-grey solid.

^1H NMR (600 MHz, $CDCl_3$) δ 7.49 (d, J = 7.7 Hz, 2H), 7.25 (d, J = 7.7 Hz, 2H), 6.95 (s, 2H), 4.51 (br s, 2H), 4.03 (q, J = 7.0 Hz, 1H), 2.42 (d, J = 2.2 Hz, 3H), 1.64 (dd, J = 7.1, 2.0 Hz, 3H).

^{13}C NMR (151 MHz, $CDCl_3$) δ 145.0, 140.5, 133.7, 129.6, 129.4, 128.8, 123.6, 119.2, 64.9, 21.7, 14.2.

HRMS (ESI-TOF): m/z $[M+H]^+$ Calcd. for $C_{15}H_{15}Cl_2NO_2SH$ 344.0279, found 344.0273

7. X-ray crystallography (3)

The single crystal was obtained by dissolved the appropriate amount of compound **3** in ethyl acetate followed by the addition of pentane to furnish a saturated solution. The solution was then allowed to stand at room temperature to form the white thin needle-like crystal.

A suitable crystal of compound **3** was mounted on top of a cryoloop and transferred into the cold (100 K) nitrogen stream of a Bruker D8 Venture diffractometer. Data collection and reduction was done using the Bruker software suite APEX3.[15] The final unit cell was obtained from the xyz centroids of 2996 reflections after integration. A multi-scan absorption correction was applied, based on the intensities of symmetry-related reflections measured at different angular settings (*SADABS*).[16] The structures were solved by direct methods using *SHELXT,*[17] and refinement of the structure was performed using *SHELXL*.[3] The hydrogen atoms were generated by geometrical considerations, constrained to idealized geometries and allowed to ride on their carrier atoms with an isotropic displacement parameter related to the equivalent displacement parameter of their carrier atoms, with the exception of the N-H hydrogen atoms which were allowed to refine freely. The Flack parameter indicated there was a small twin

[15] Bruker (2016) *APEX3, SAINT and SADABS.* Bruker AXS Inc., Madison, Wisconsin, USA.
[16] Sheldrick, G. M.; *Acta Cryst. A*, **2015**, *71*, 3-8.
[17] Sheldrick, G. M.; *Acta Cryst. C*, **2015**, *71*, 3-8.

component present, which was refined using the BASF/TWIN commands, where the BASF refined to 0.005. Crystal data and details on data collection and refinement are presented in Table S1.

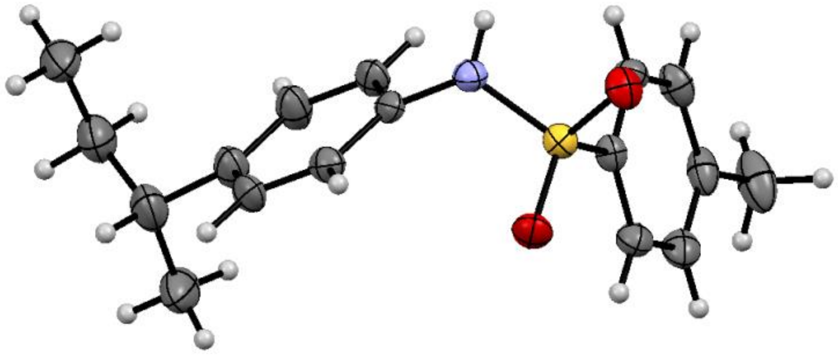

Figure 1. X-Ray Structure compound **3**, showing 50% probability ellipsoids.

Table S1. Crystallographic data for compound **3**

chem formula	$C_{17}H_{21}NO_2S$
M_r	303.41
cryst syst	monoclinic
color, habit	colorless, needle
size (mm)	0.311 x 0.141 x 0.036
space group	$P2_1$
a (Å)	11.1356(17)
b (Å)	6.3226(7)
c (Å)	23.245(3)
β (°)	100.343(10)
V (Å^3)	1610.0(4)
Z	4
〉$_{calc}$, g.cm^{-3}	1.252
Radiation [Å]	Cu K$_\alpha$ 1.54178
μ(Cu K$_\alpha$), mm^{-1}	1.812
F(000)	648
temp (K)	100(2)
⎝ range (°)	1.932 – 72.854
data collected (h,k,l)	-13:13; -6:7; -27:28
no. of rflns collected	19661
no. of indpndt reflns	5721
observed reflns F_o ε 2.0 σ (F_o)	5318
R(F) (%)	4.99
wR(F^2) (%)	12.90
GooF	1.024
weighting a,b	0.0698, 008566
params refined	394
min, max resid dens	-0.32, 0.734

8. NMR Spectra

1b^{1}H NMR (400 MHz, $CDCl_3$)

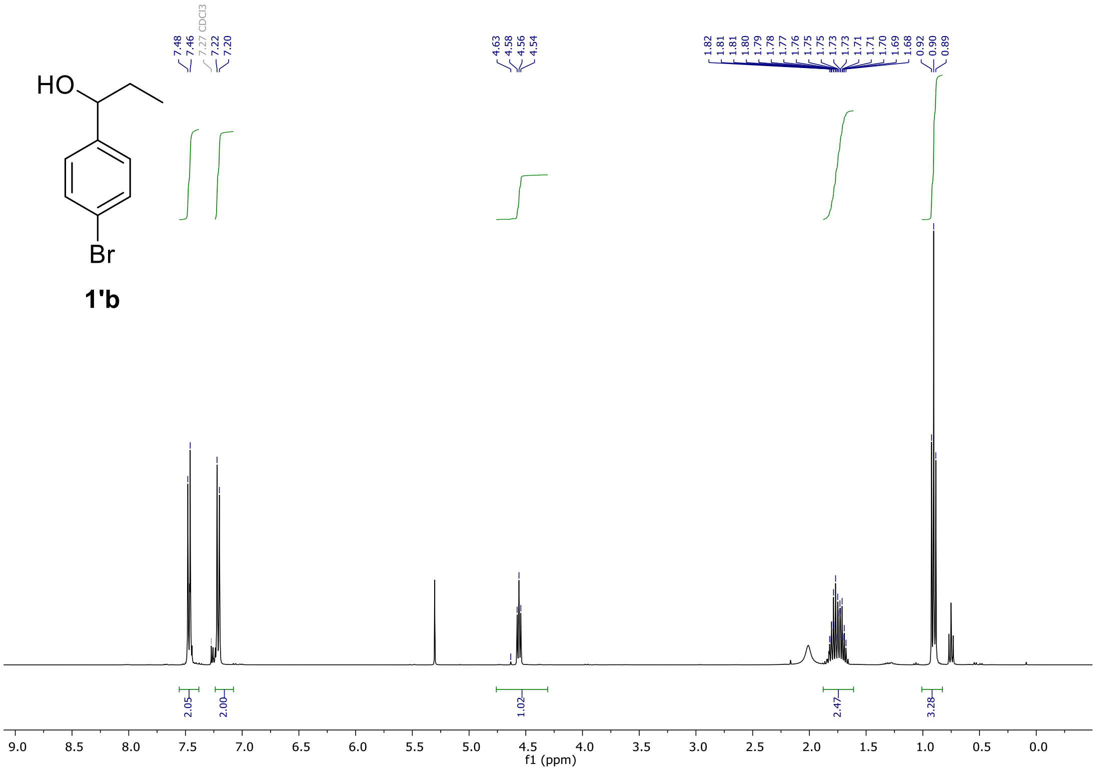

1b ^{13}C NMR (101 MHz, $CDCl_3$)

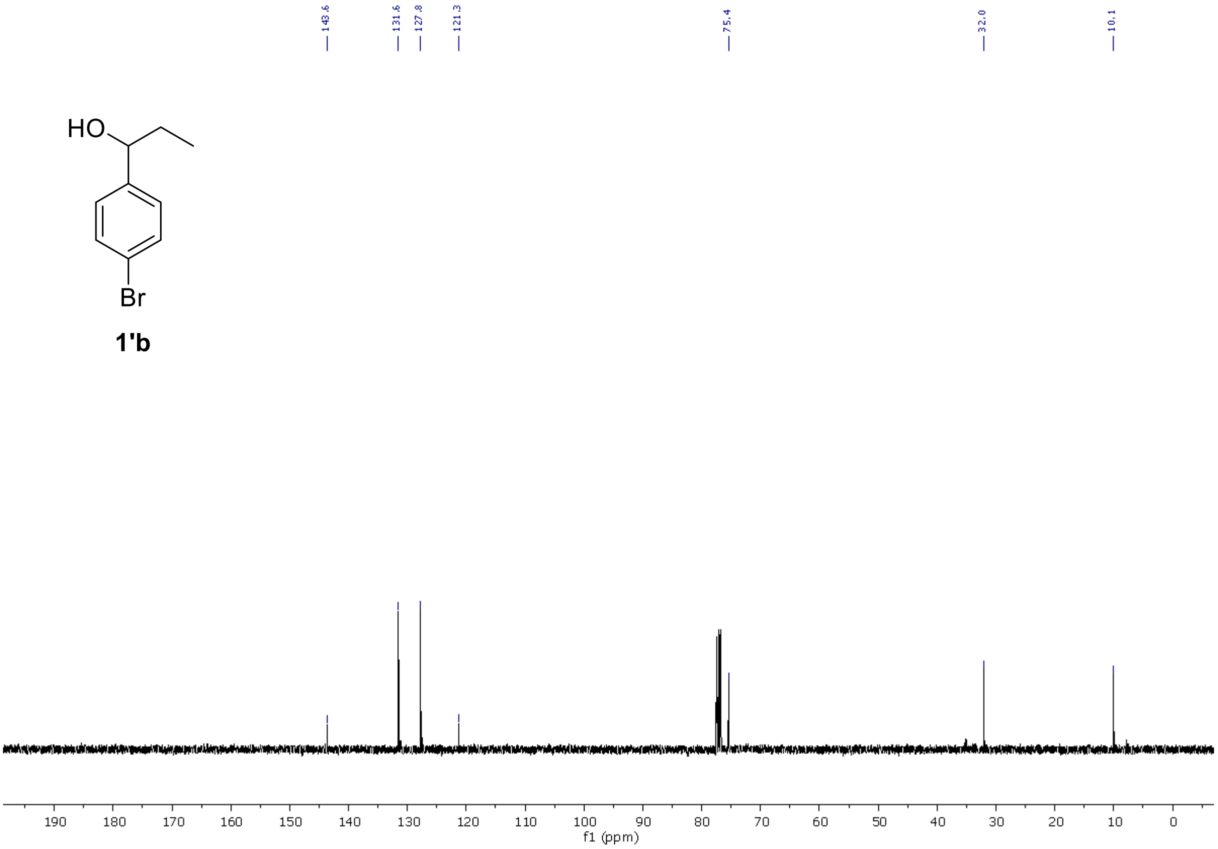

1’n ^{1}H NMR (400 MHz, $CDCl_3$)

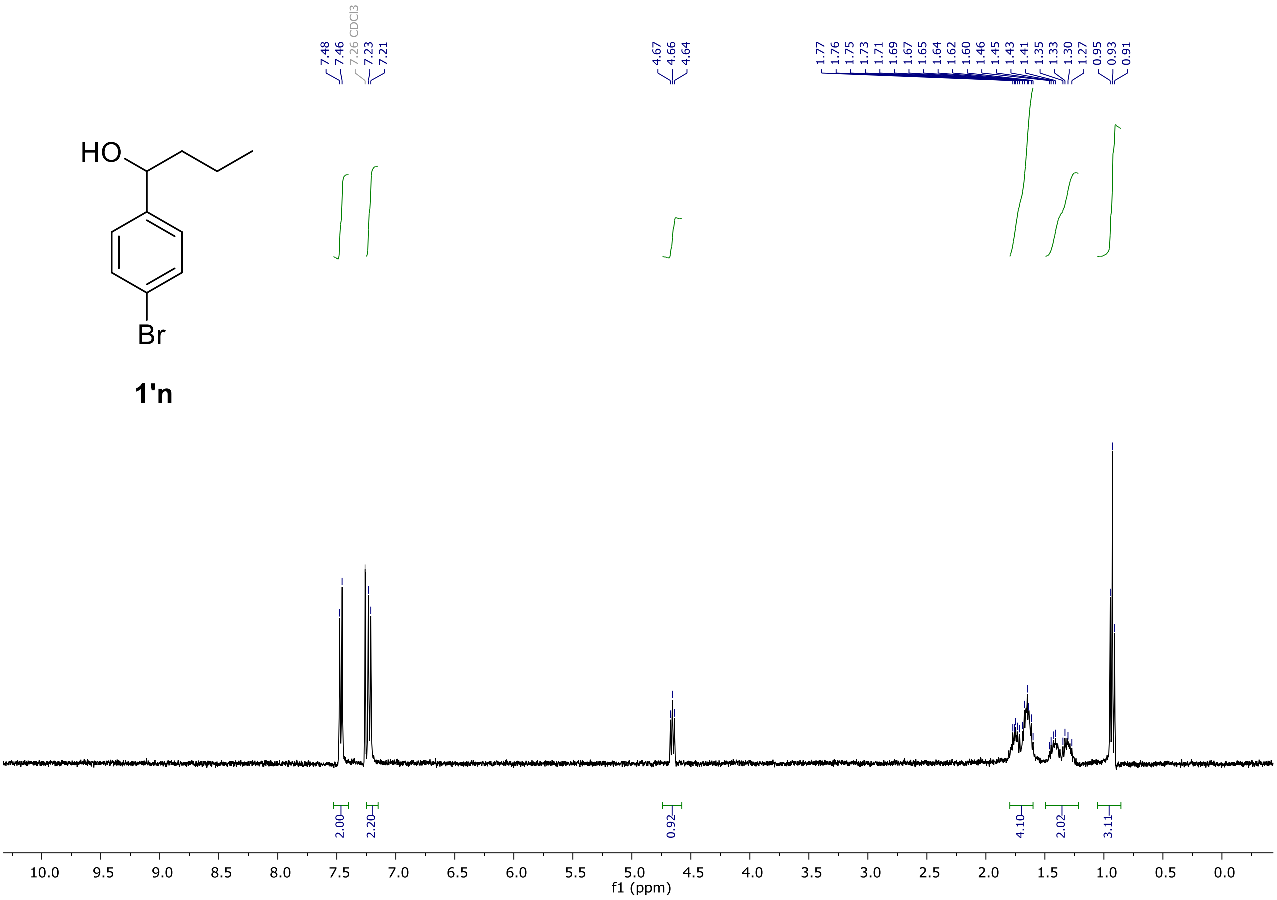

1’n ^{13}C NMR (151 MHz, $CDCl_3$)

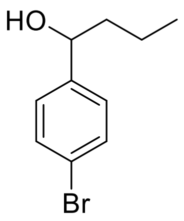

1'n

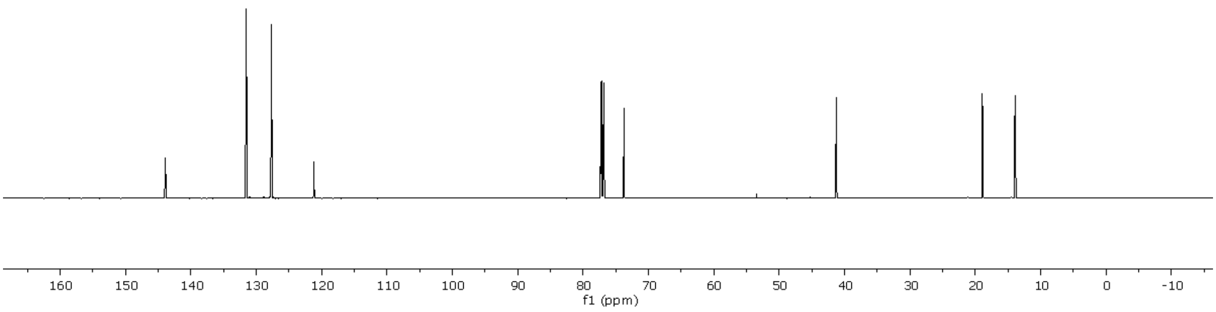

1’o ^{1}H NMR (400 MHz, $CDCl_3$)

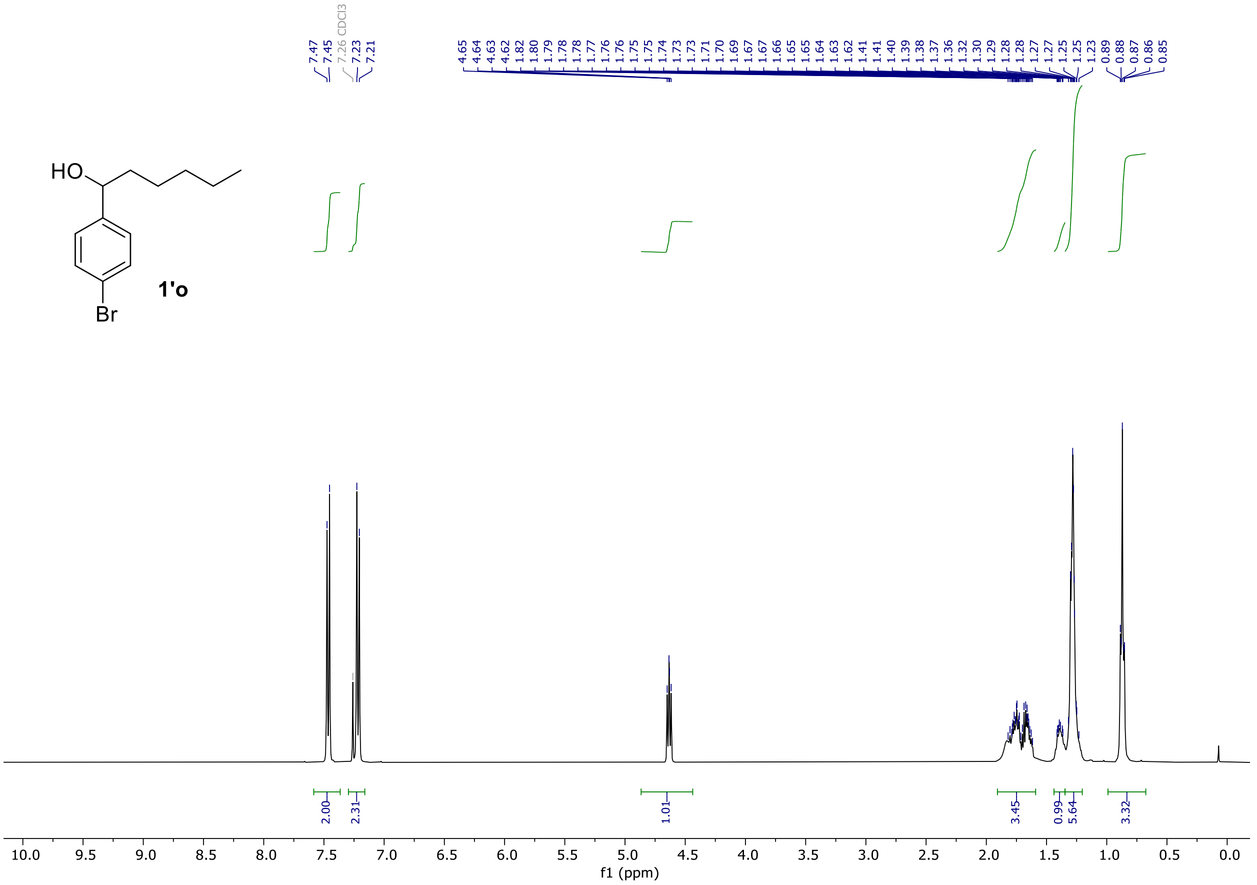

1’o ^{13}C NMR (101 MHz, $CDCl_3$)

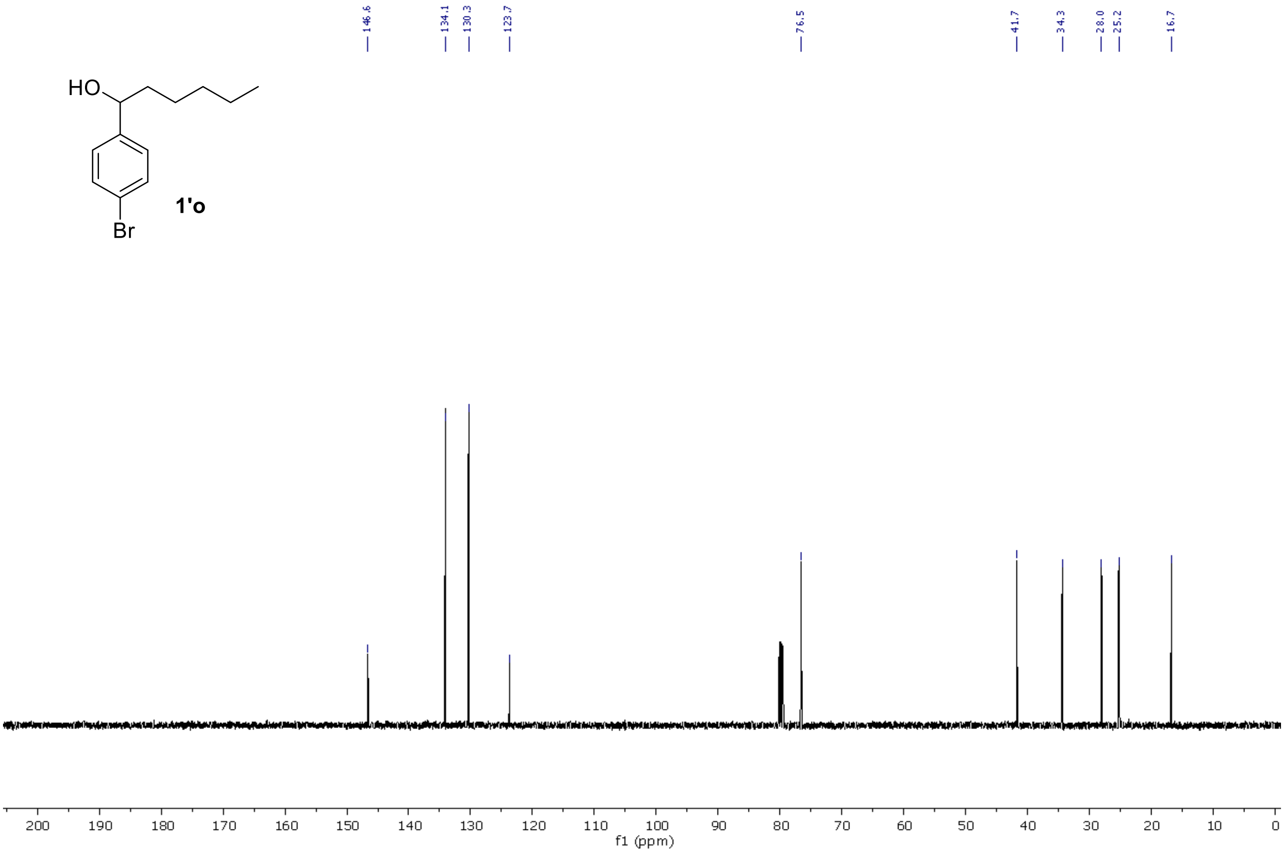

1’p ^{1}H NMR (400 MHz, $CDCl_3$)

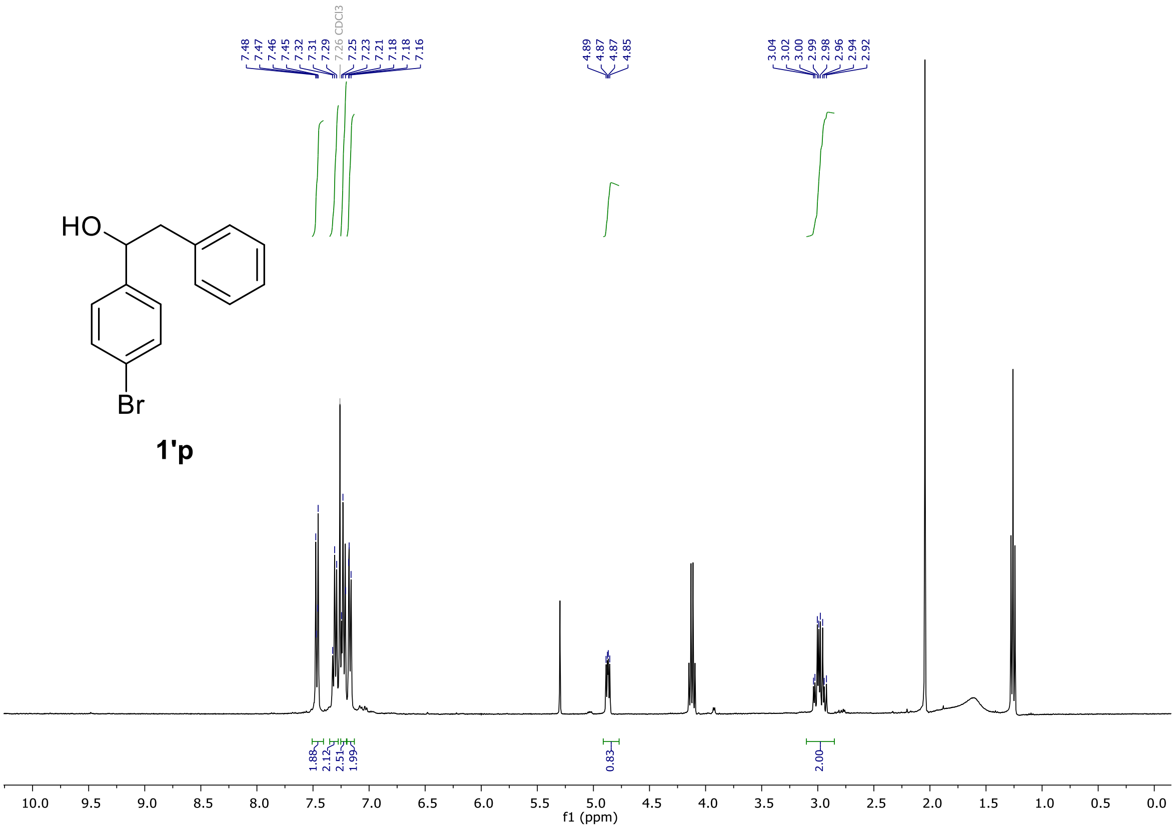

1’p 1 ^{13}C NMR (101 MHz, $CDCl_3$)

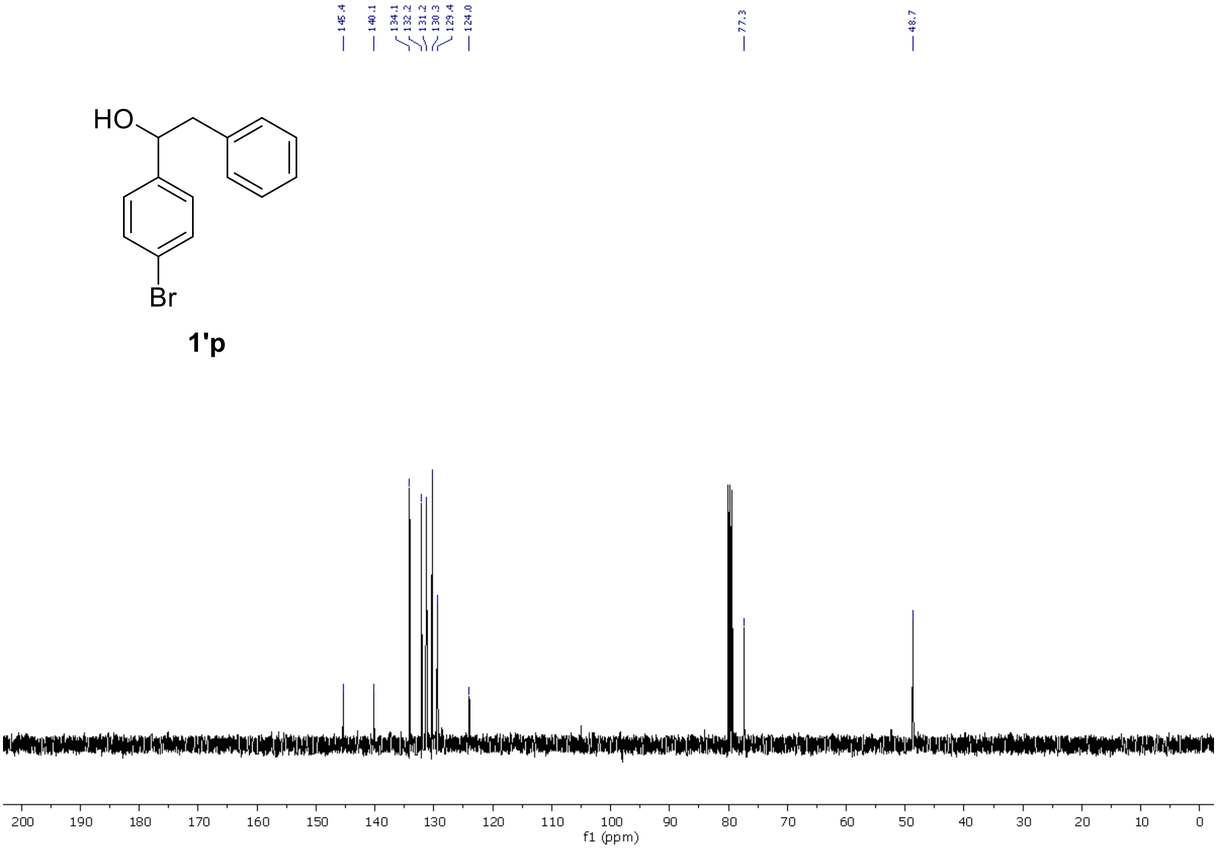

1’q ^{1}H NMR (400 MHz, $CDCl_3$)

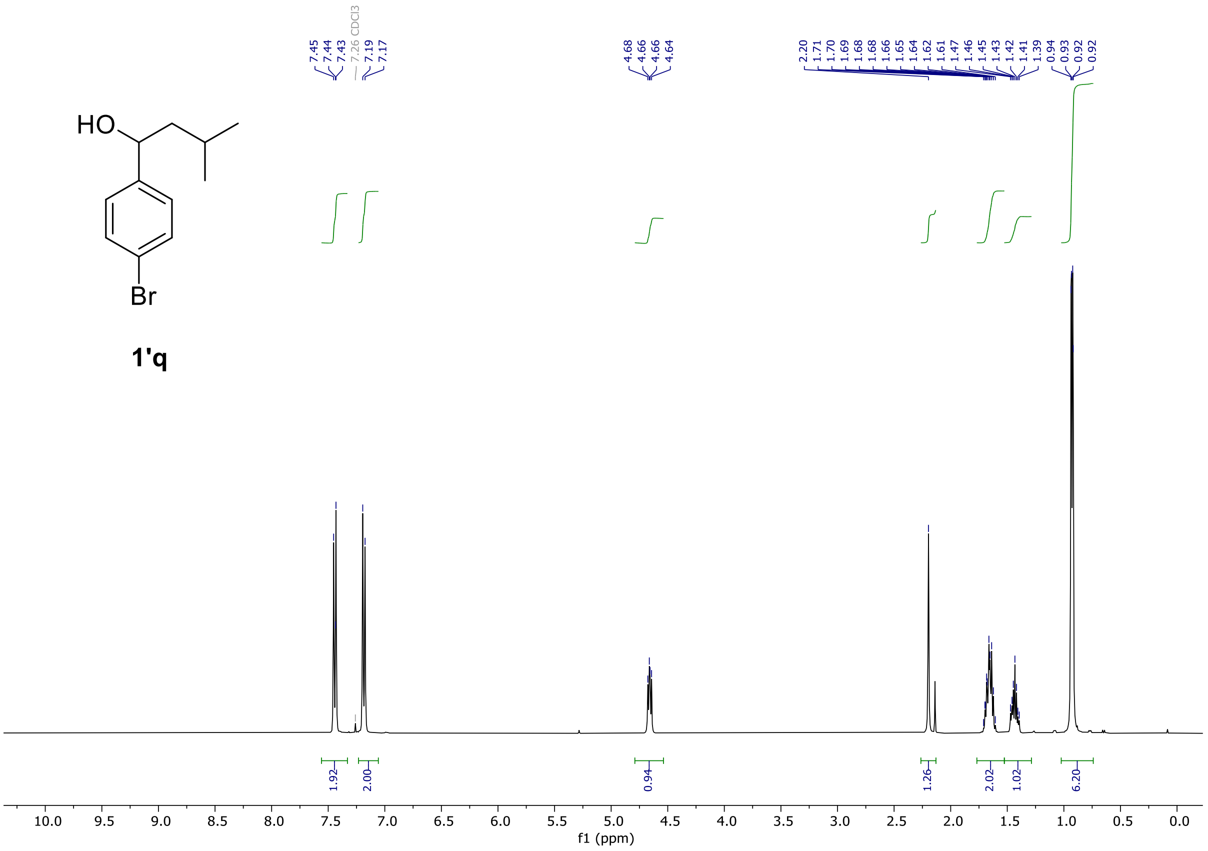

1’q ^{13}C NMR (151 MHz, $CDCl_3$)

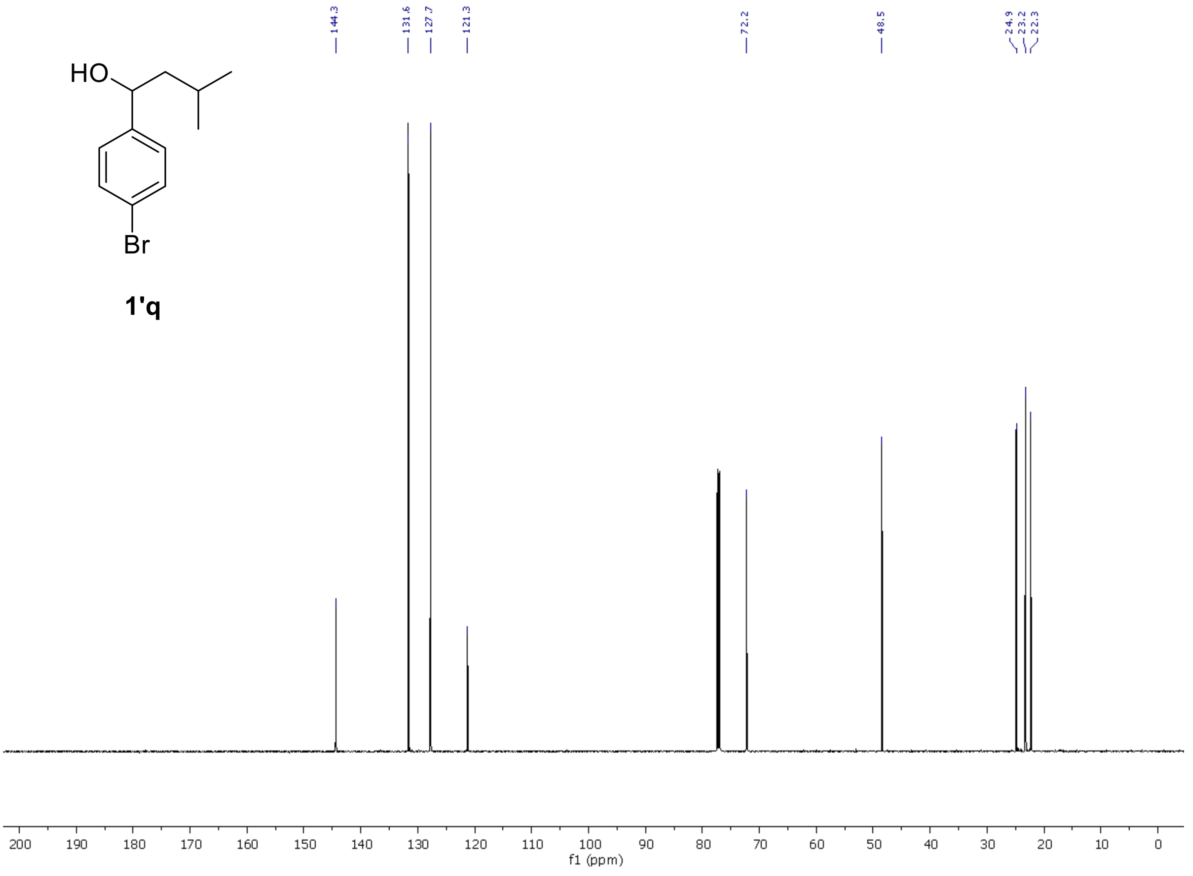

1’r ^{1}H NMR (400 MHz, $CDCl_3$)

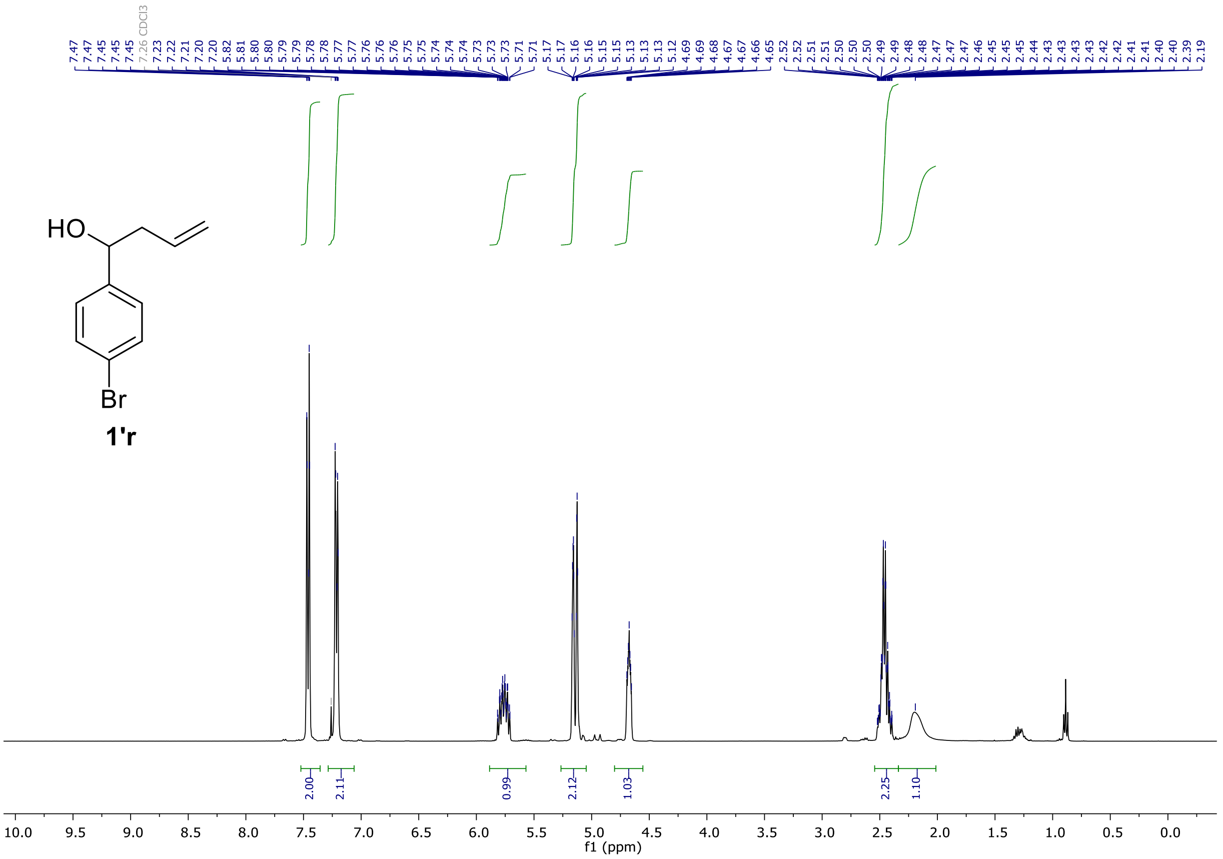

1’r ^{13}C NMR (75 MHz, $CDCl_3$)

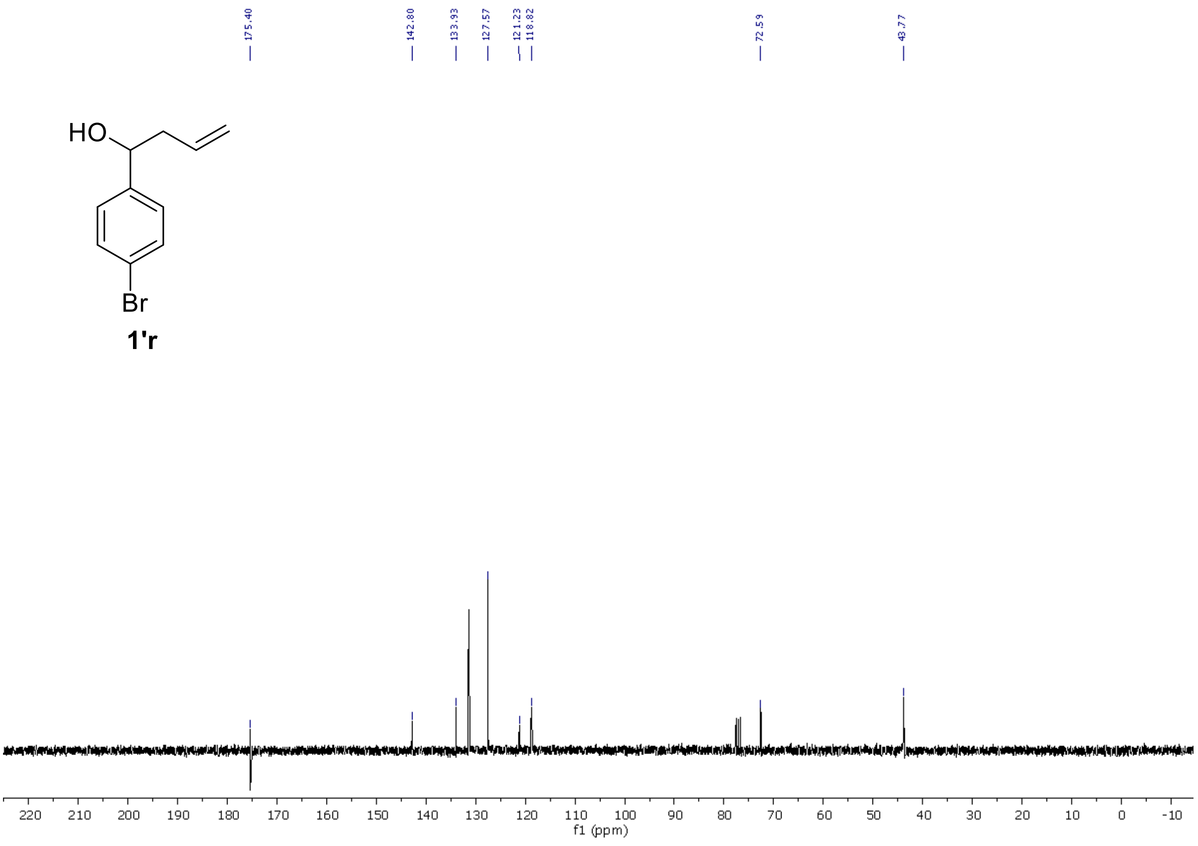

1's ^{1}H NMR (400 MHz, $CDCl_3$)

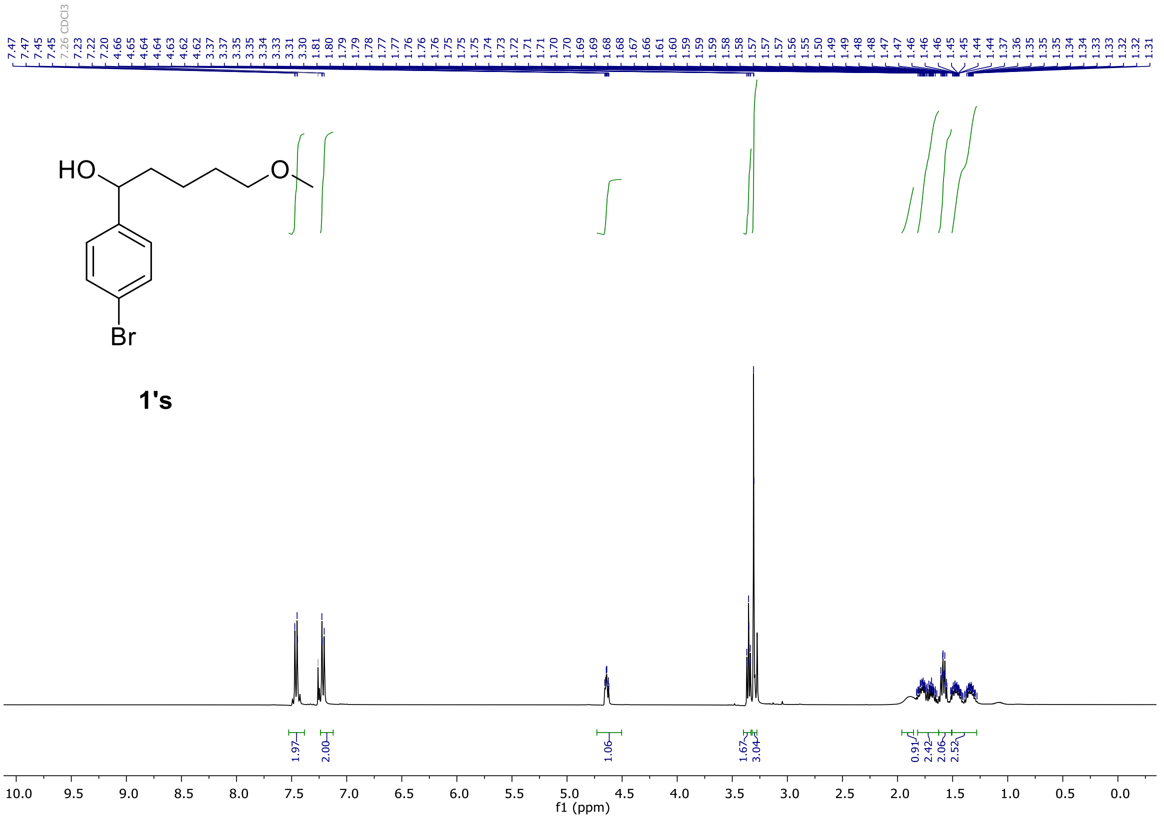

1's ^{13}C NMR (101 MHz, $CDCl_3$)

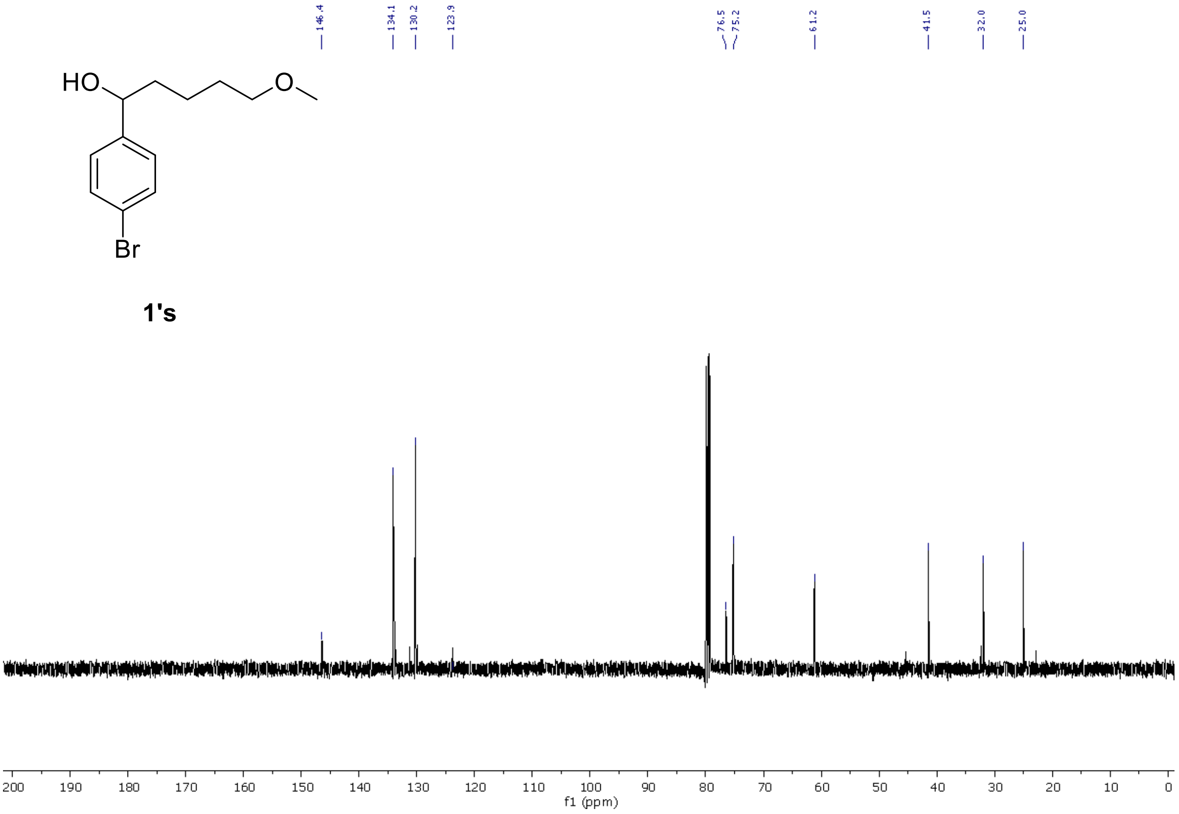

1b ^{1}H NMR (400 MHz, $CDCl_3$)

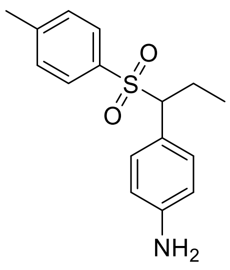

1b

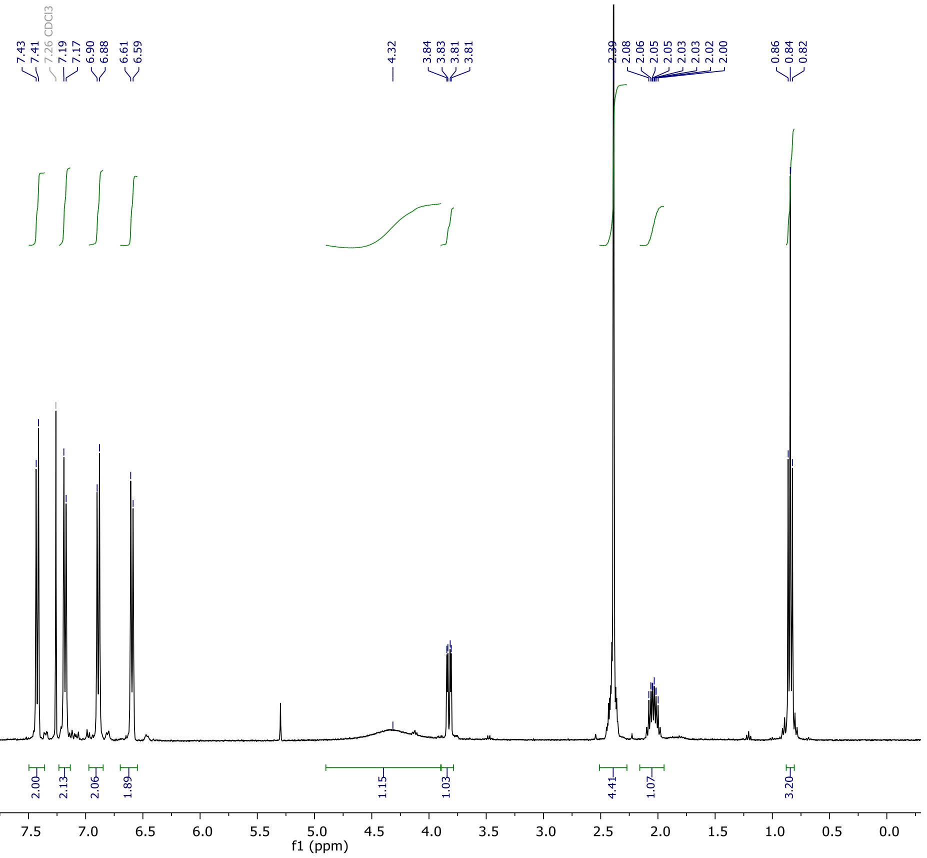

1b ^{13}C NMR (151 MHz, $CDCl_3$)

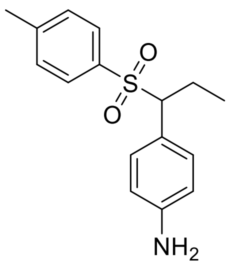

1b

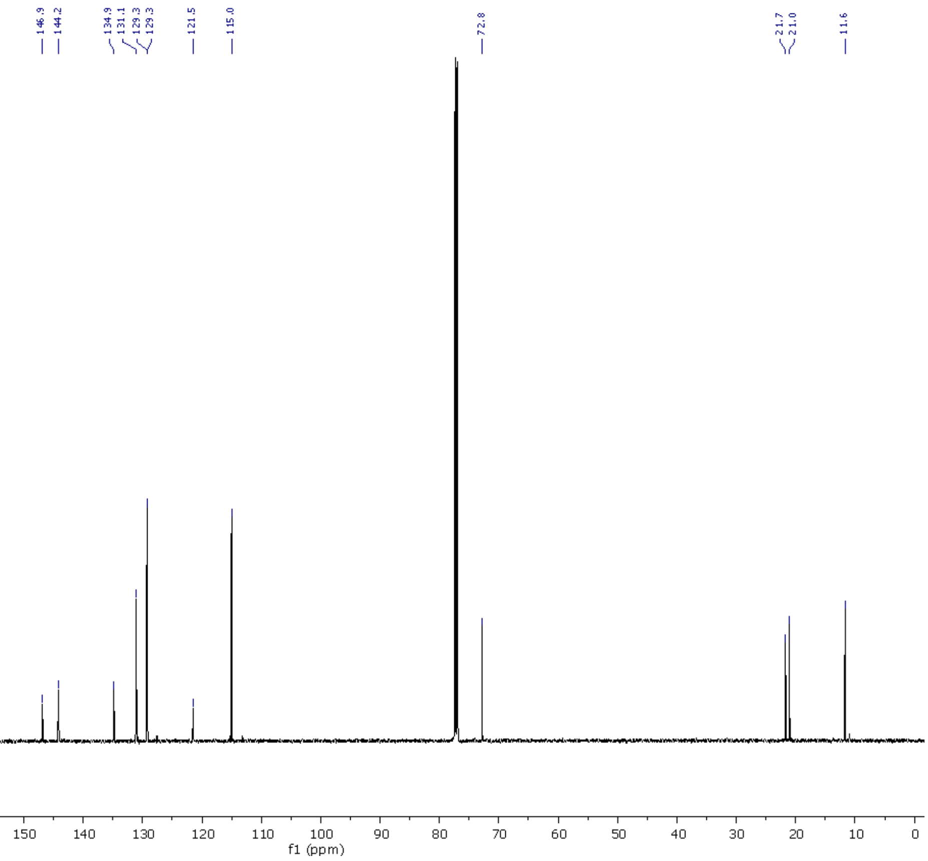

1n ^1H NMR (600 MHz, $CDCl_3$)

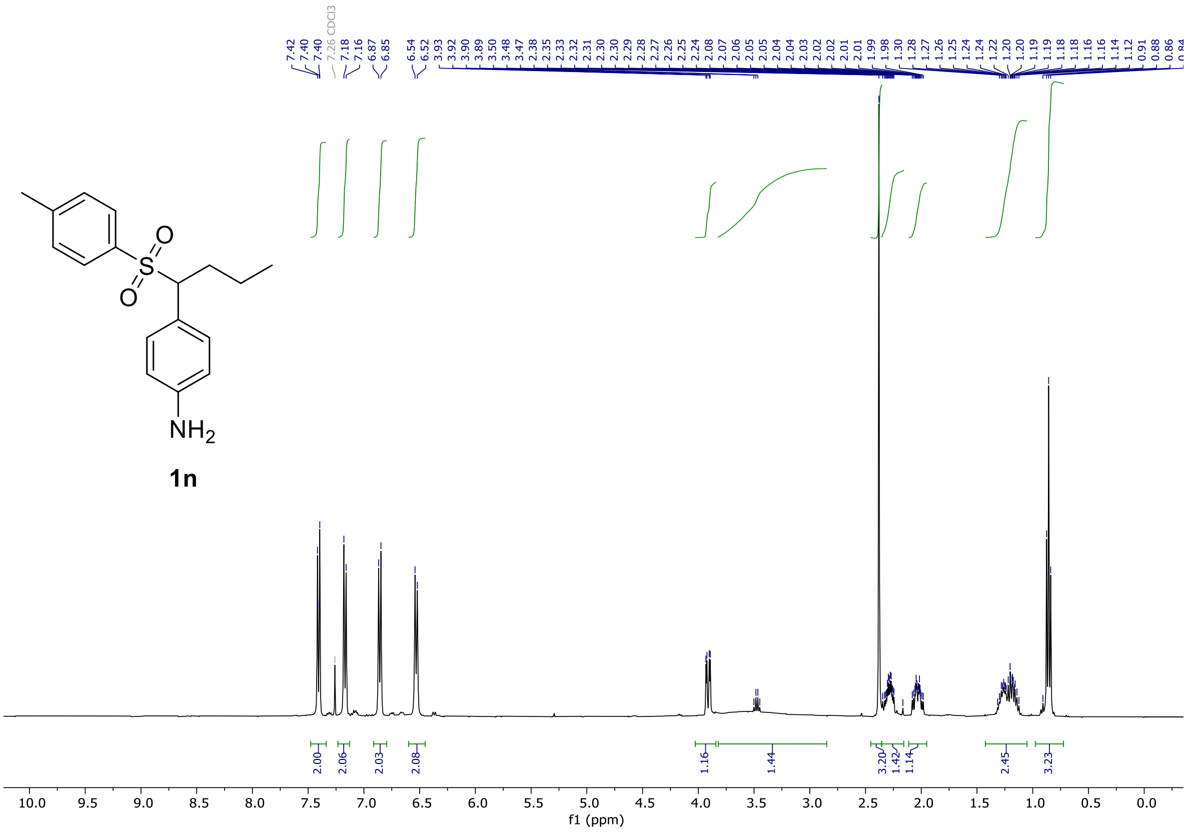

1n ^{13}C NMR (151 MHz, $CDCl_3$)

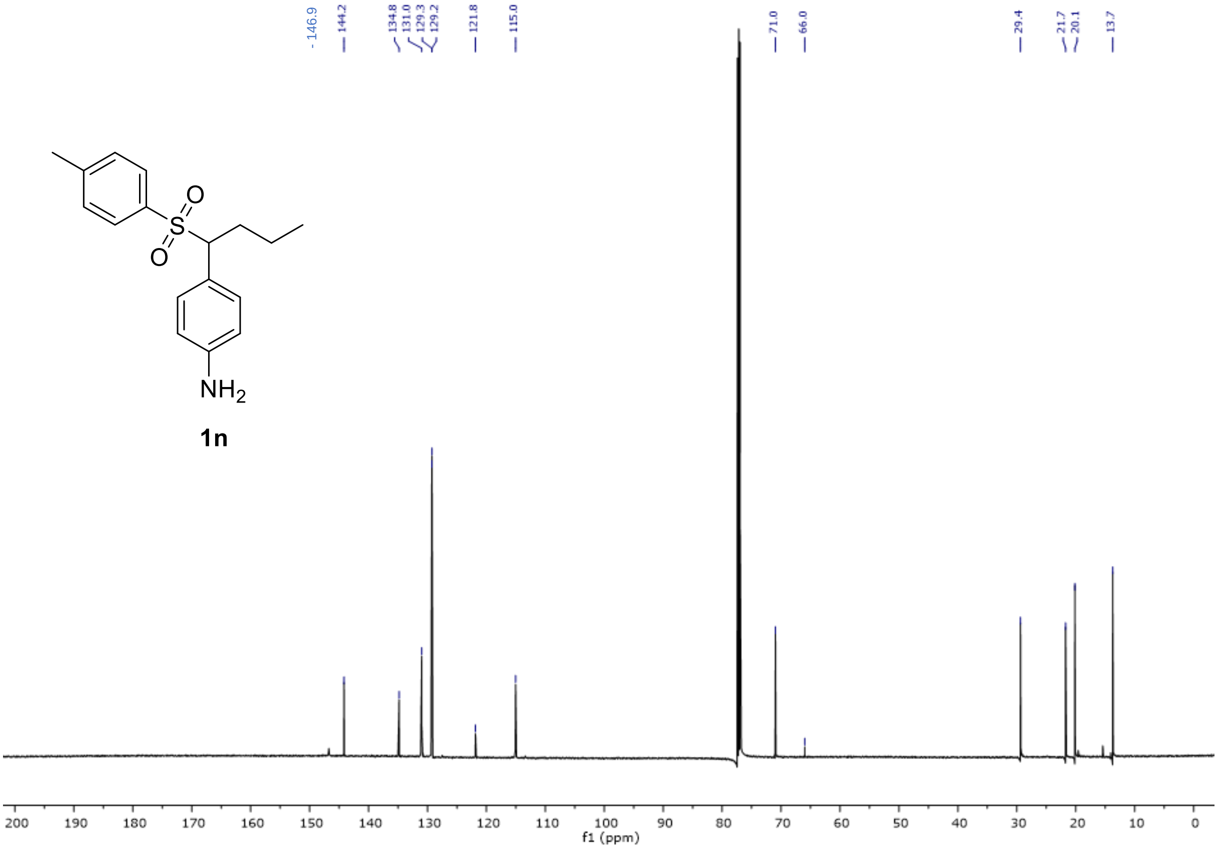

1o ^{1}H NMR (400 MHz, $CDCl_3$)

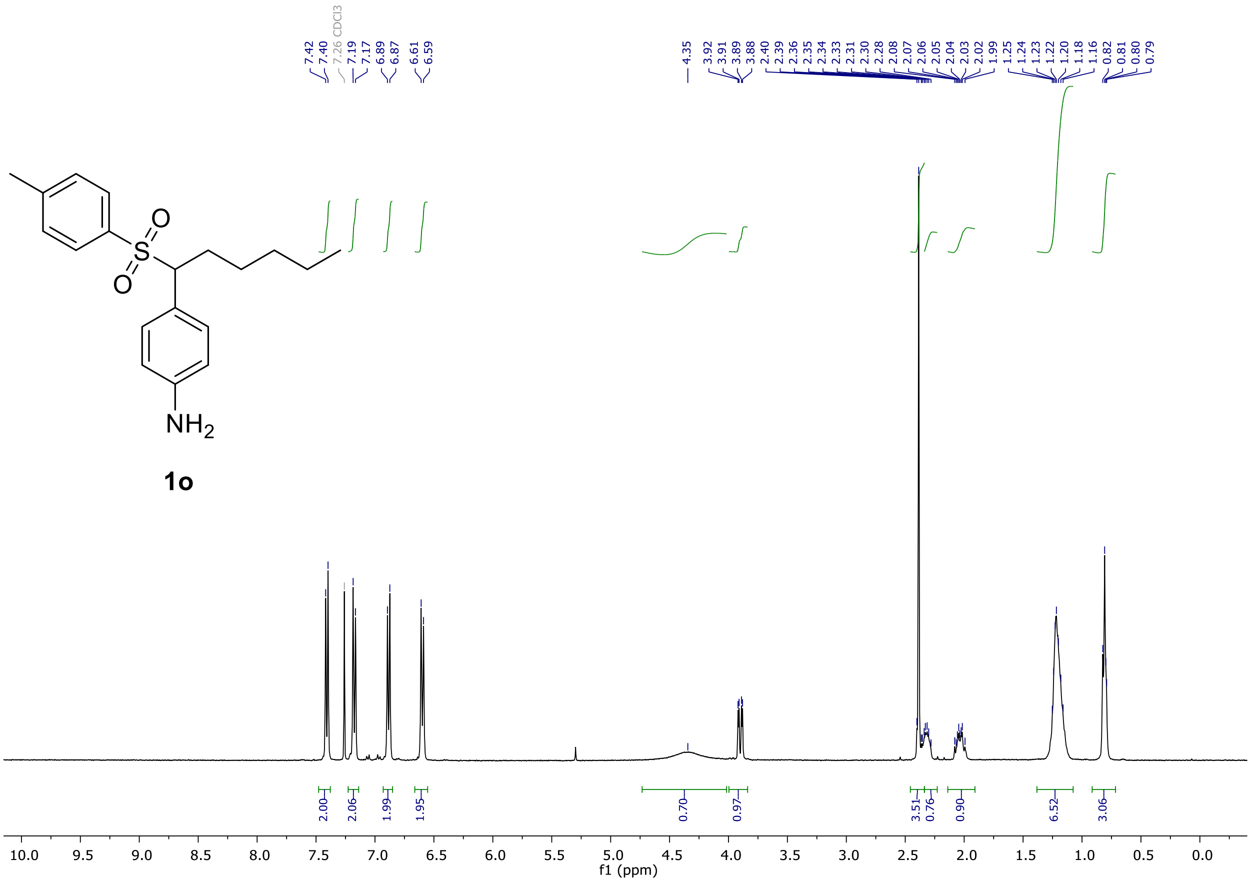

1o ^{13}C NMR (101 MHz, CDCl3)

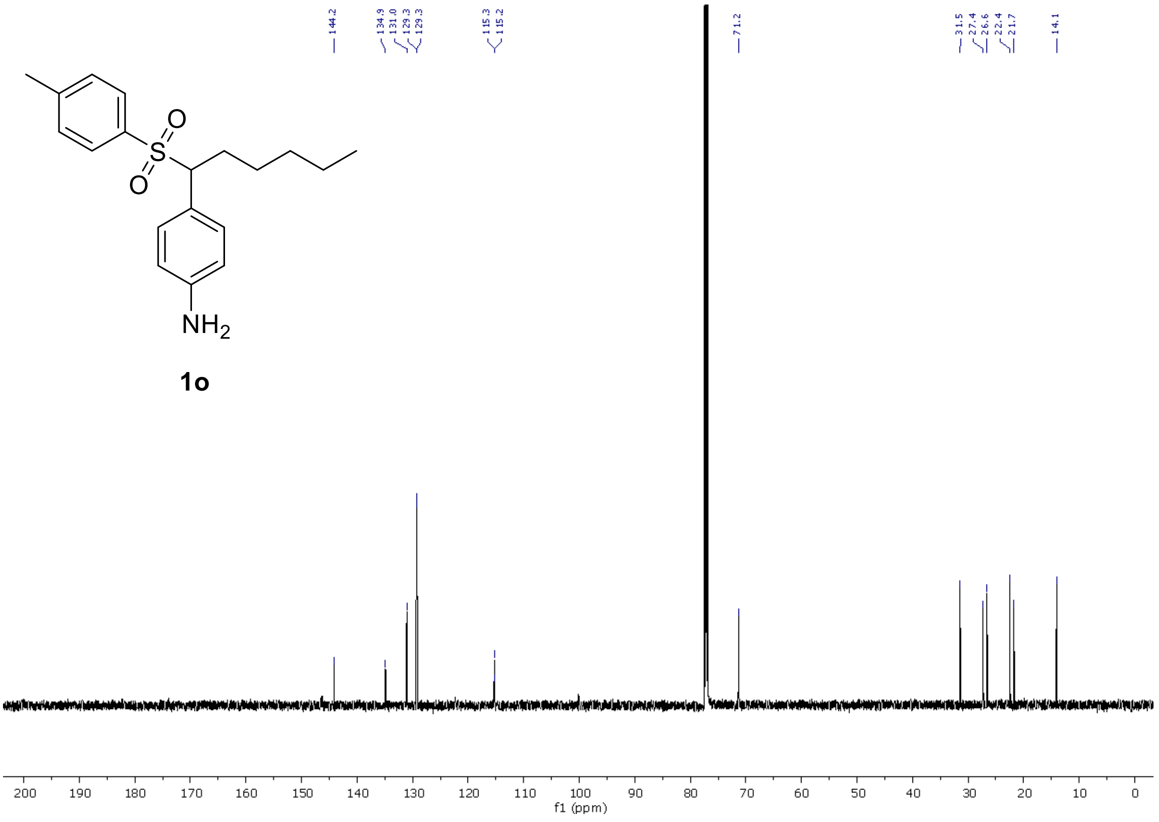

1p ^{1}H NMR (400 MHz, DMSO-*d6*)

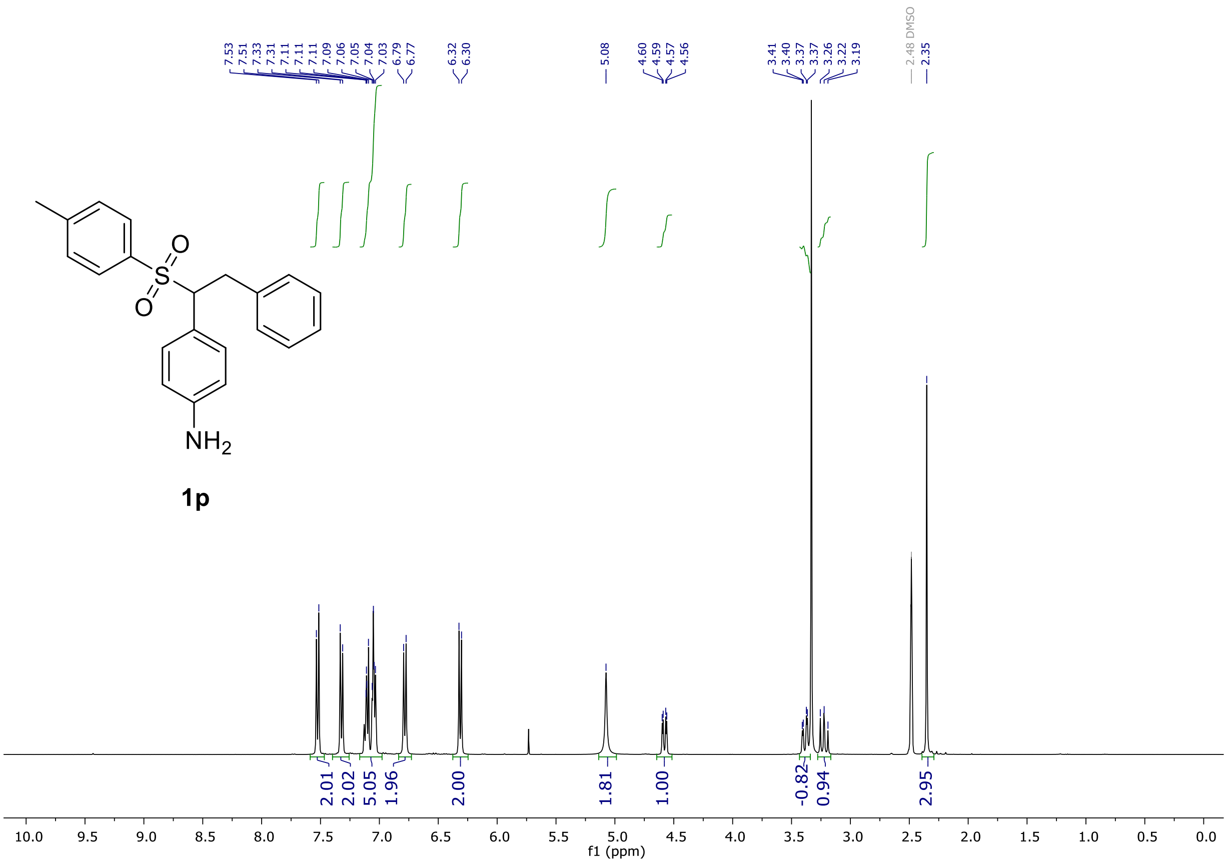

1p ^{13}C NMR (101 MHz, DMSO-*d6*)

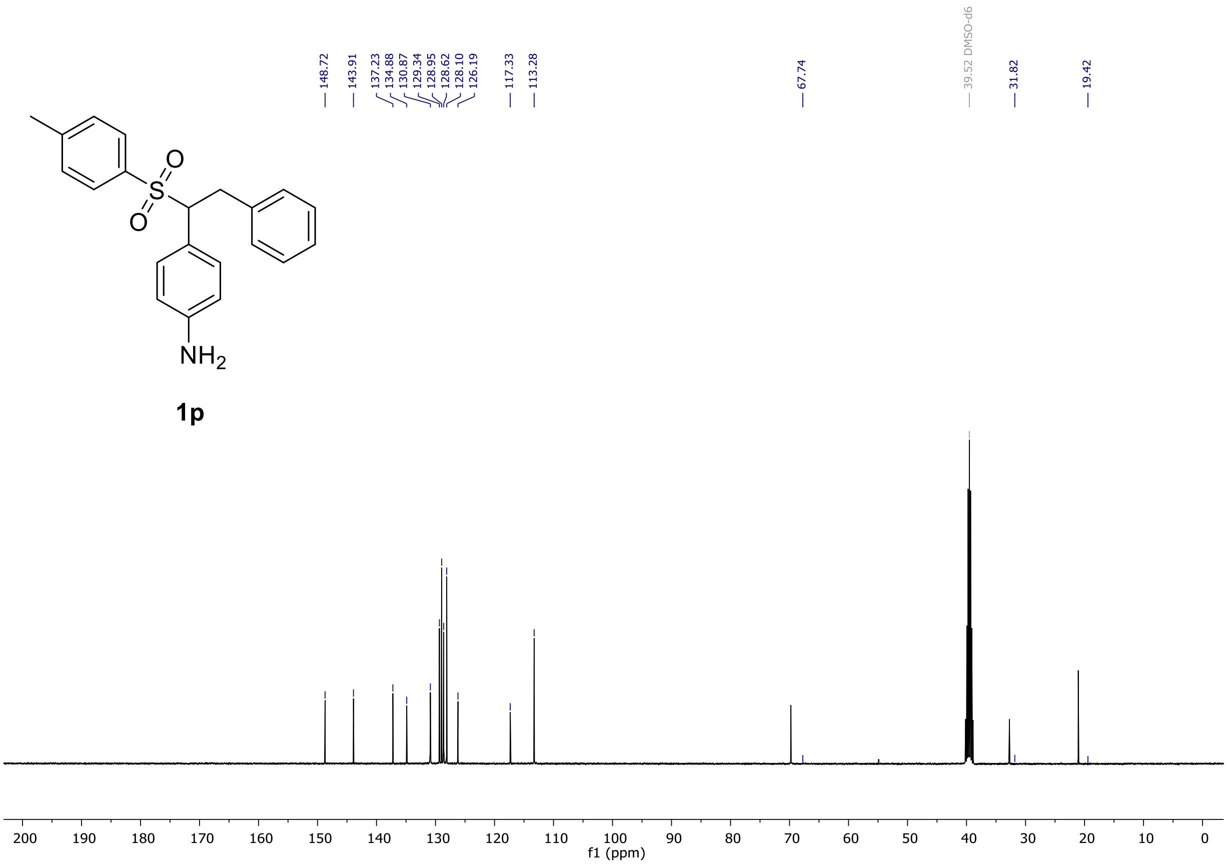

1q ^{1}H NMR (600 MHz, $CDCl_3$)

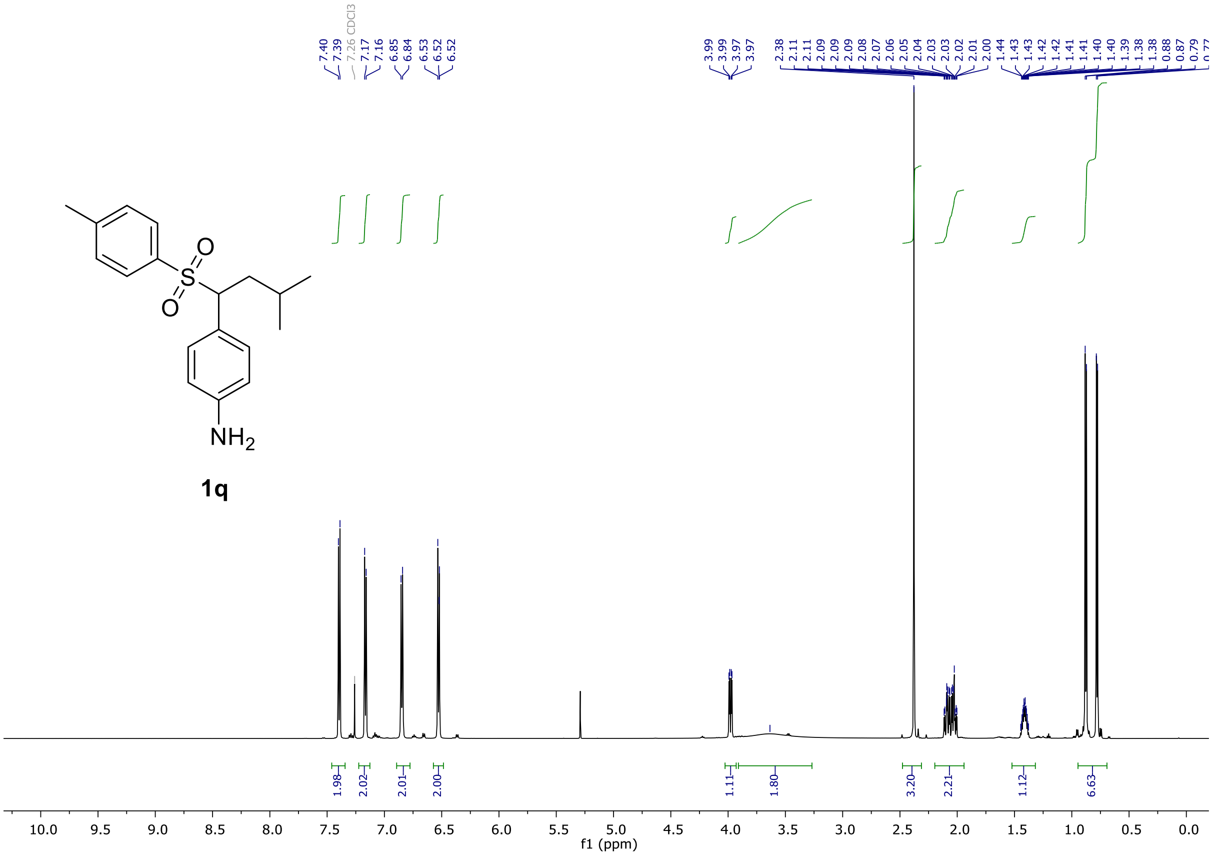

1q ^{13}C NMR (151 MHz, $CDCl_3$)

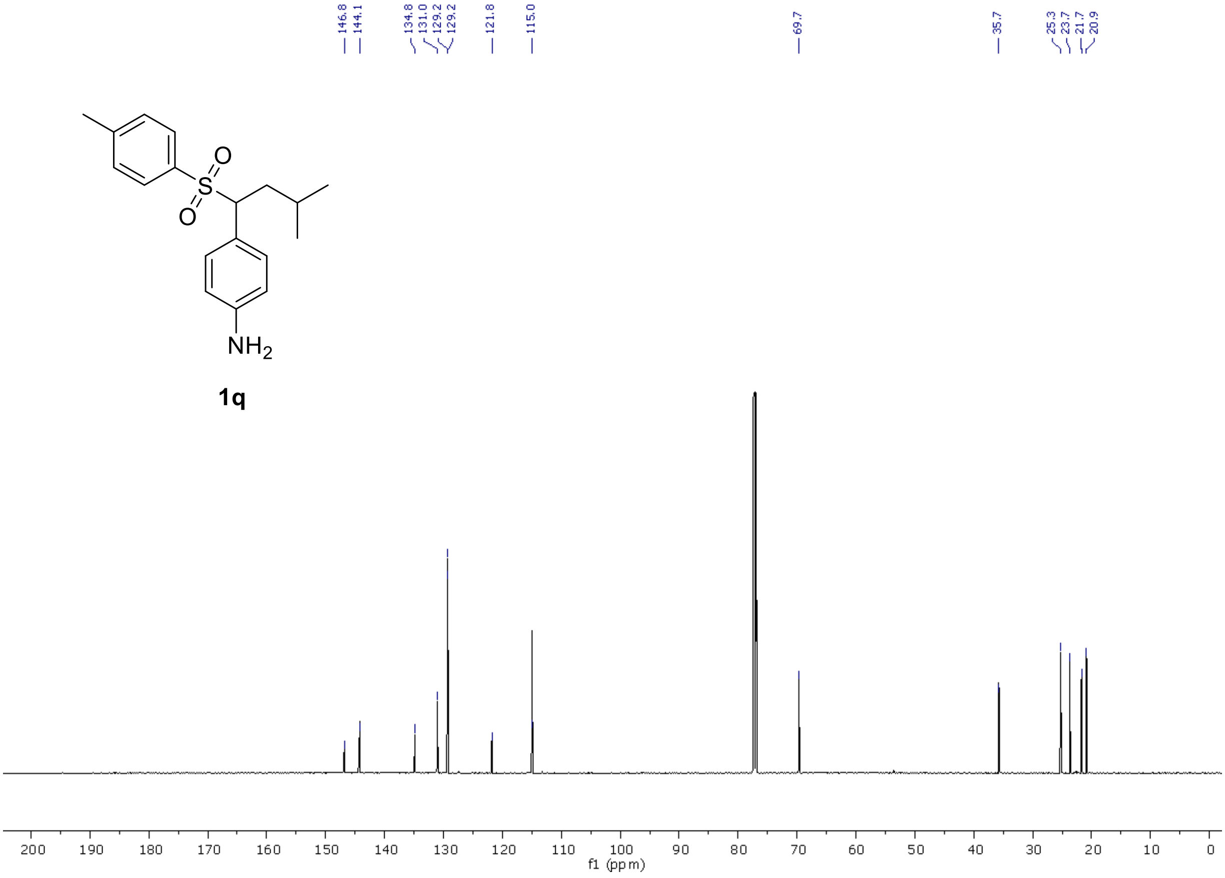

1r ^{1}H NMR (400 MHz, $CDCl_3$)

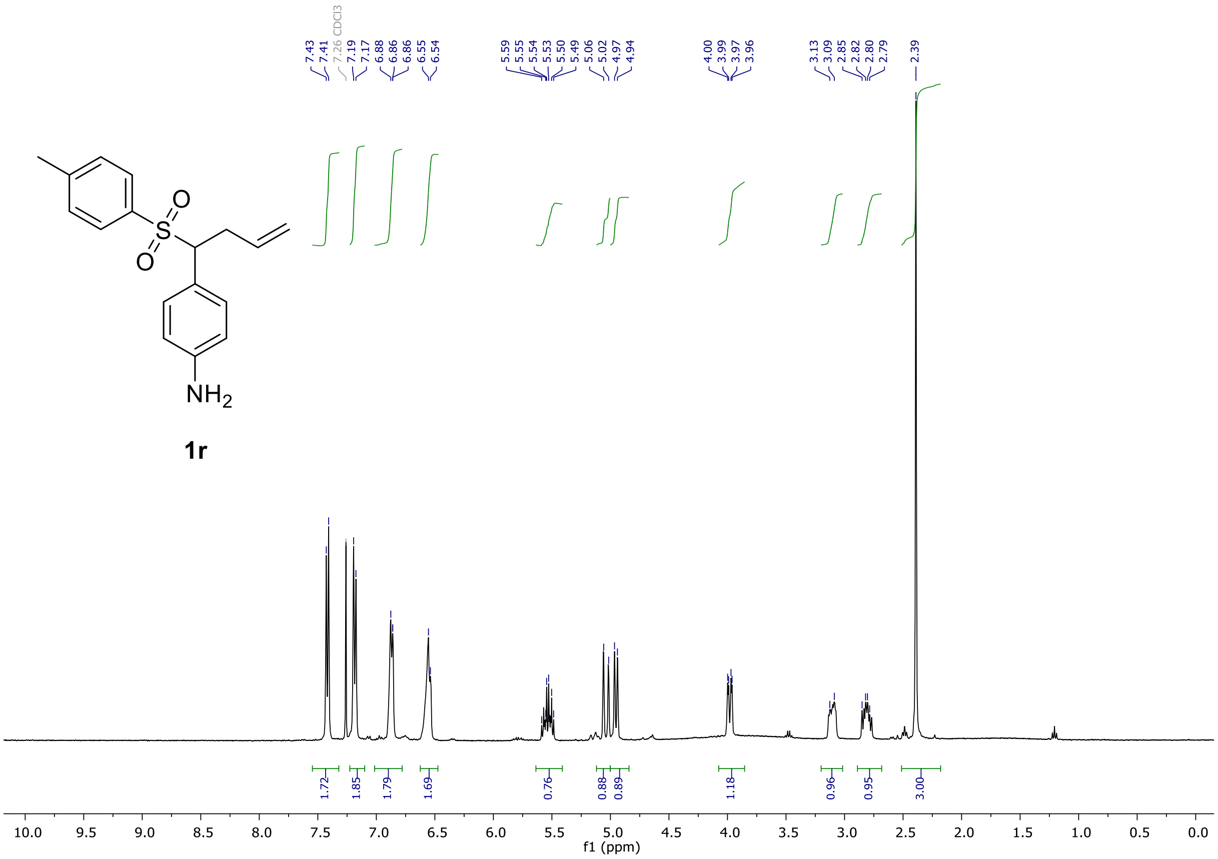

1r ^{13}C NMR (101 MHz, $CDCl_3$)

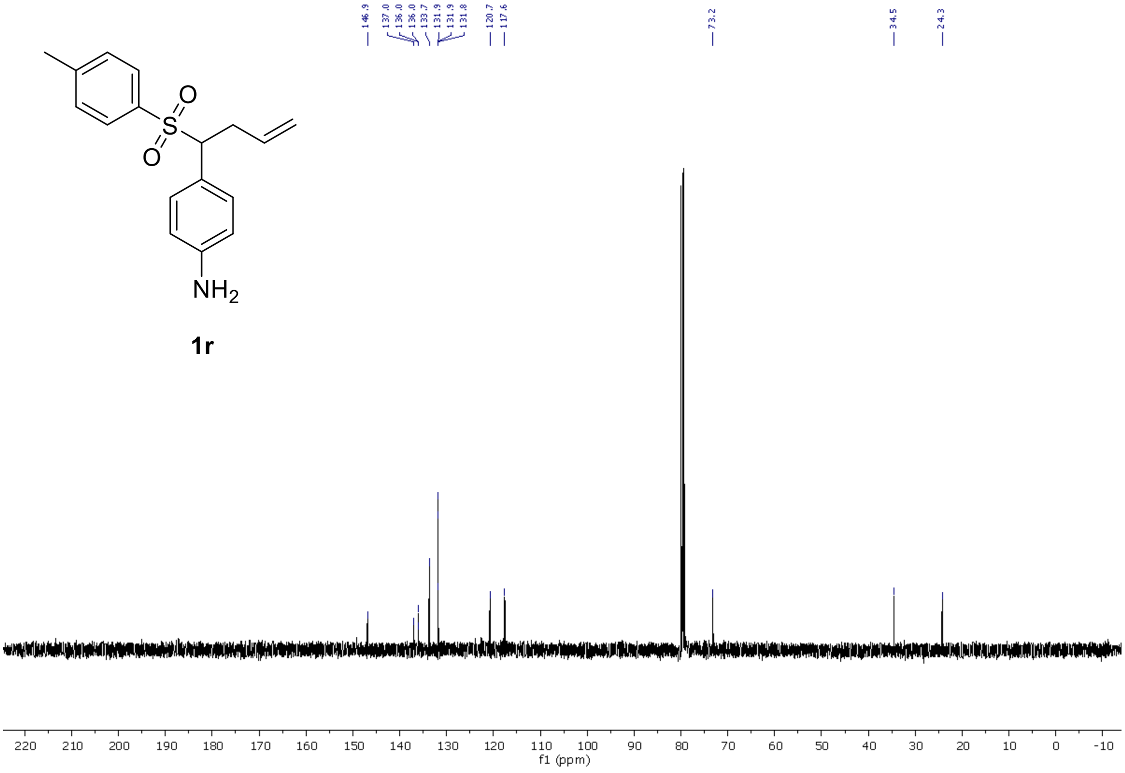

1s ^{1}H NMR (400 MHz, $CDCl_3$)

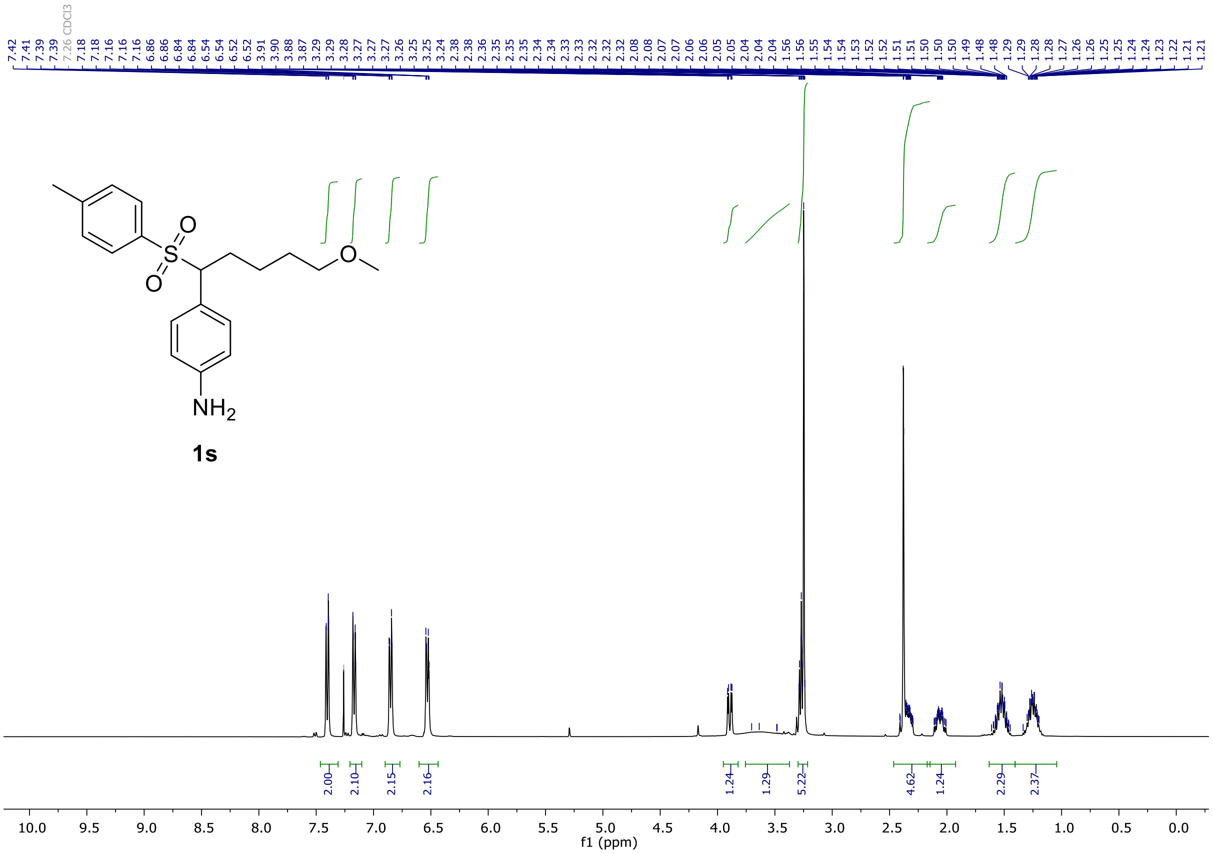

1s ^{13}C NMR (101 MHz, $CDCl_3$)

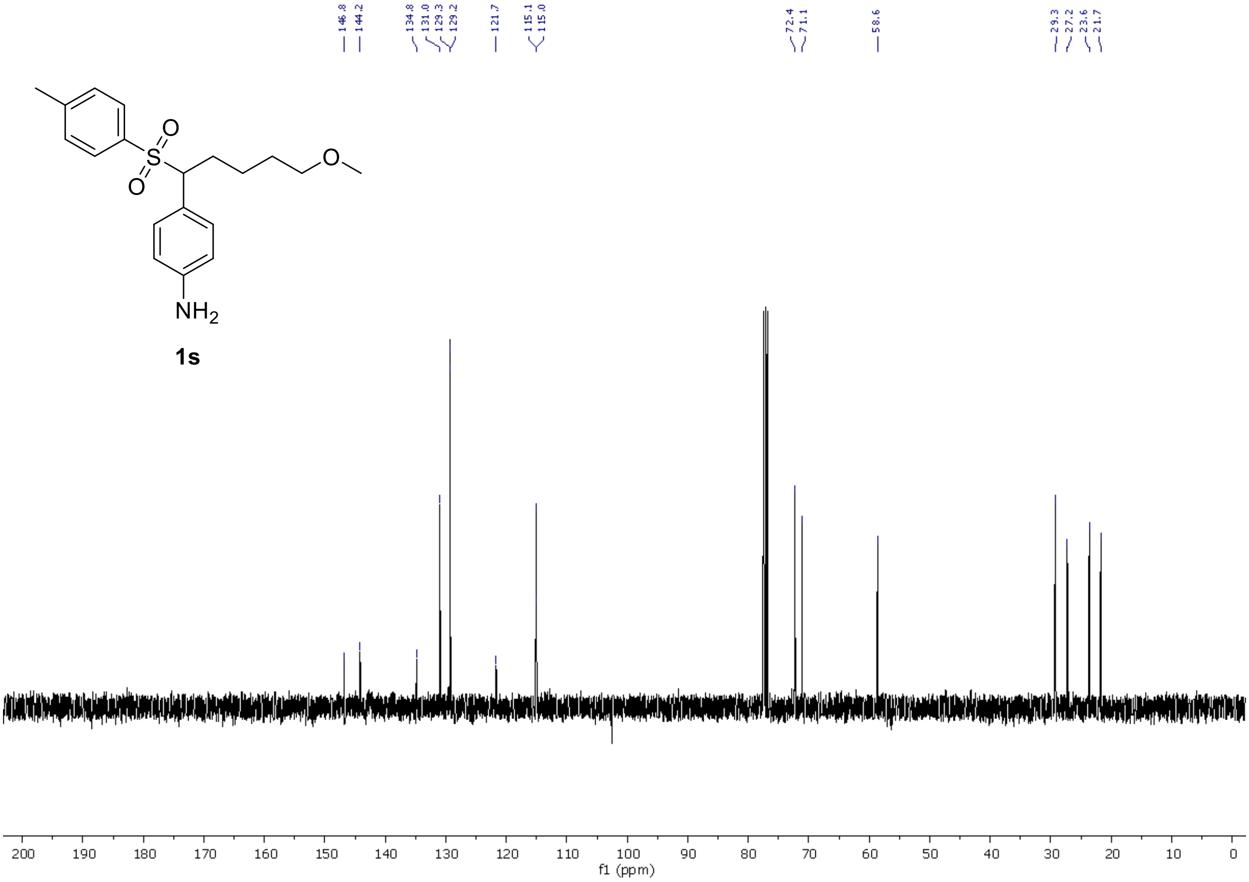

1a ^{1}H NMR (400 MHz, $CDCl_3$)

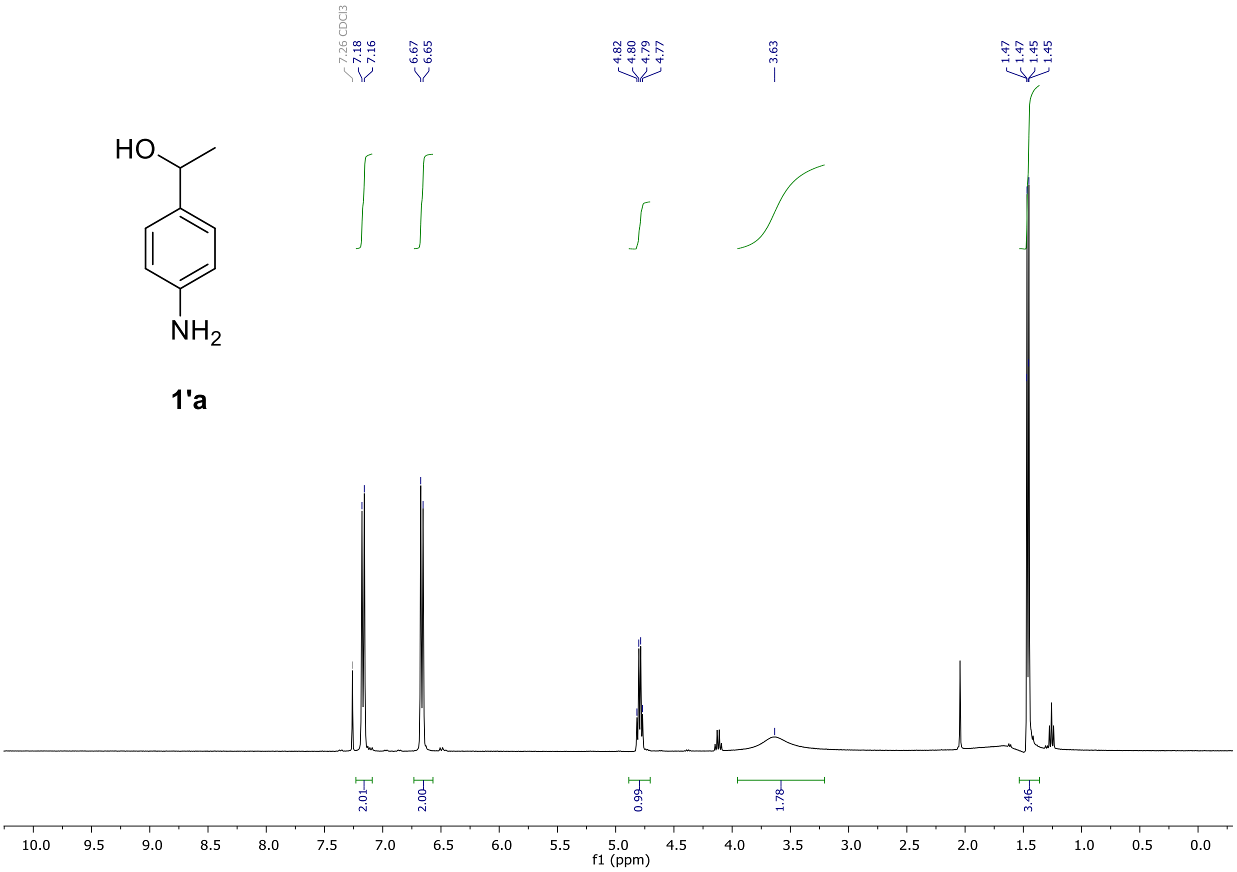

1’a ^{1}H NMR (101 MHz, $CDCl_3$)

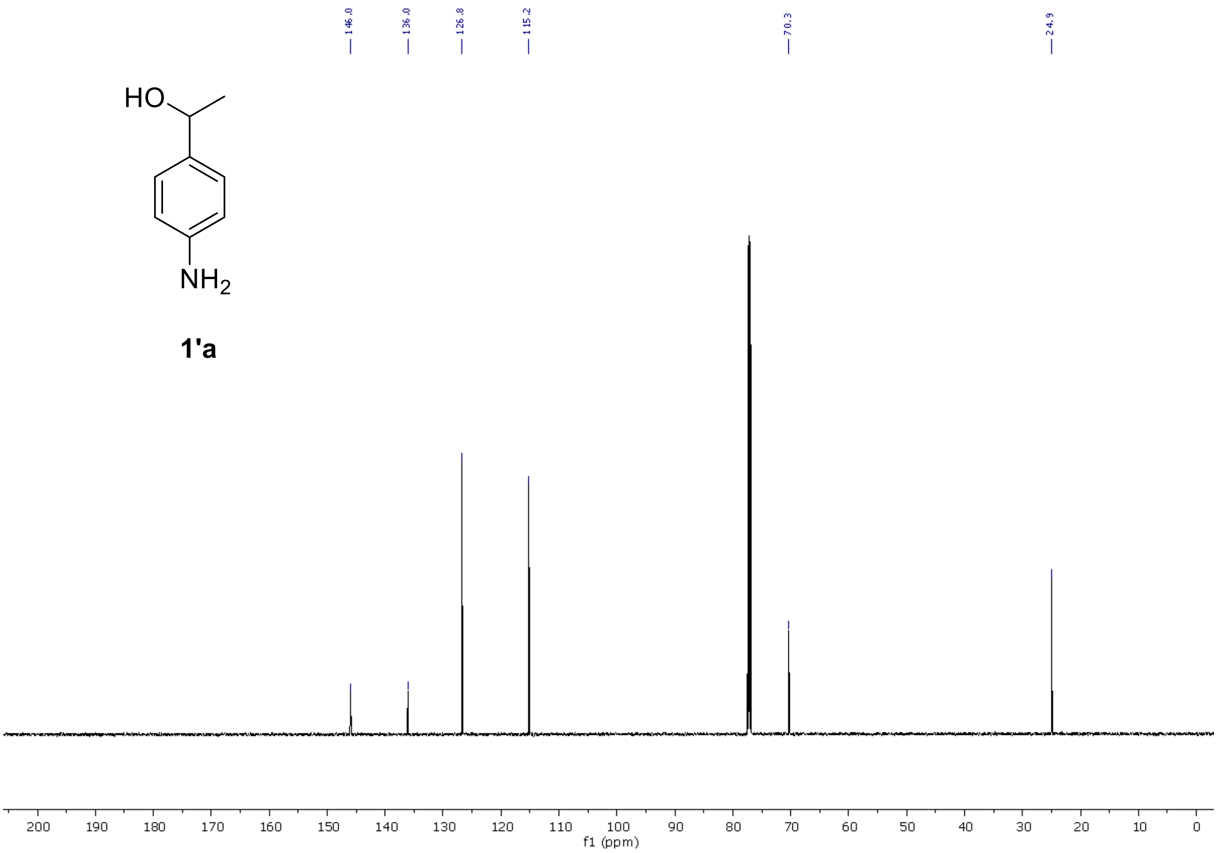

1a ^{1}H NMR (600 MHz, $CDCl_3$)

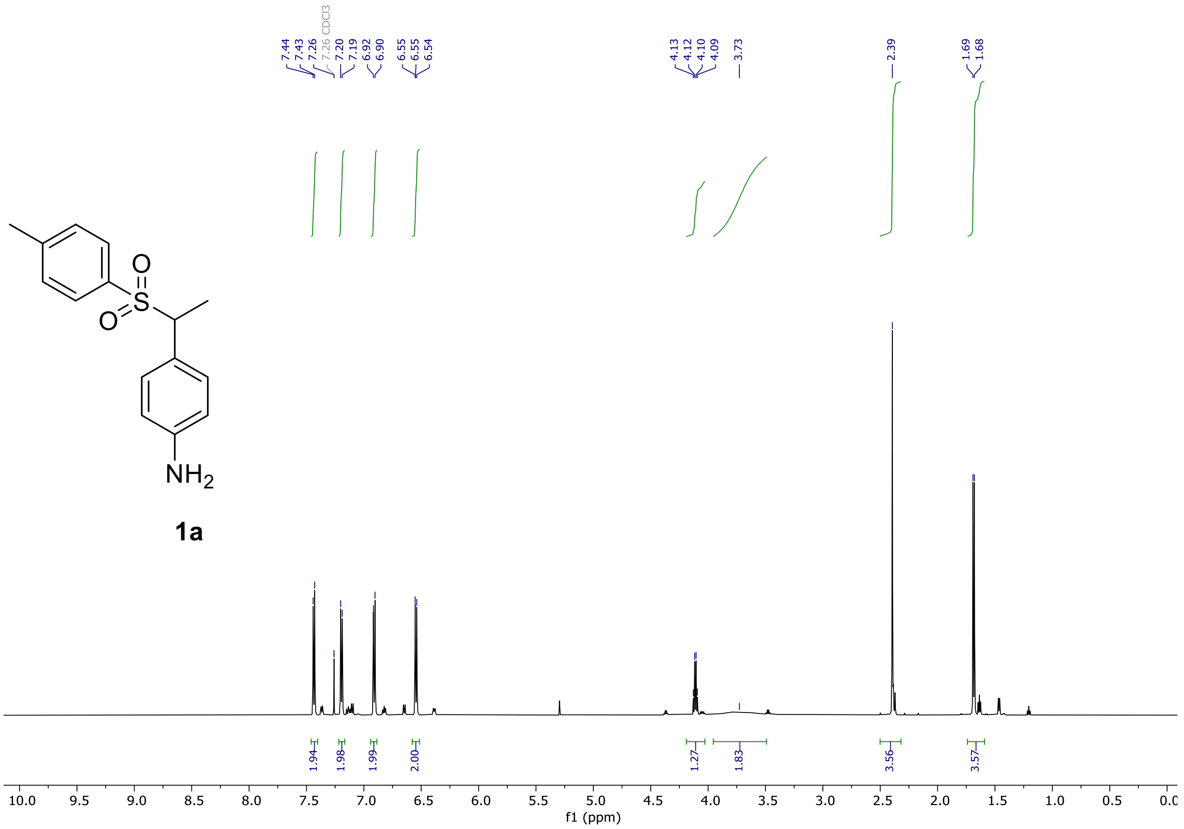

1a ^{13}C NMR (151 MHz, $CDCl_3$)

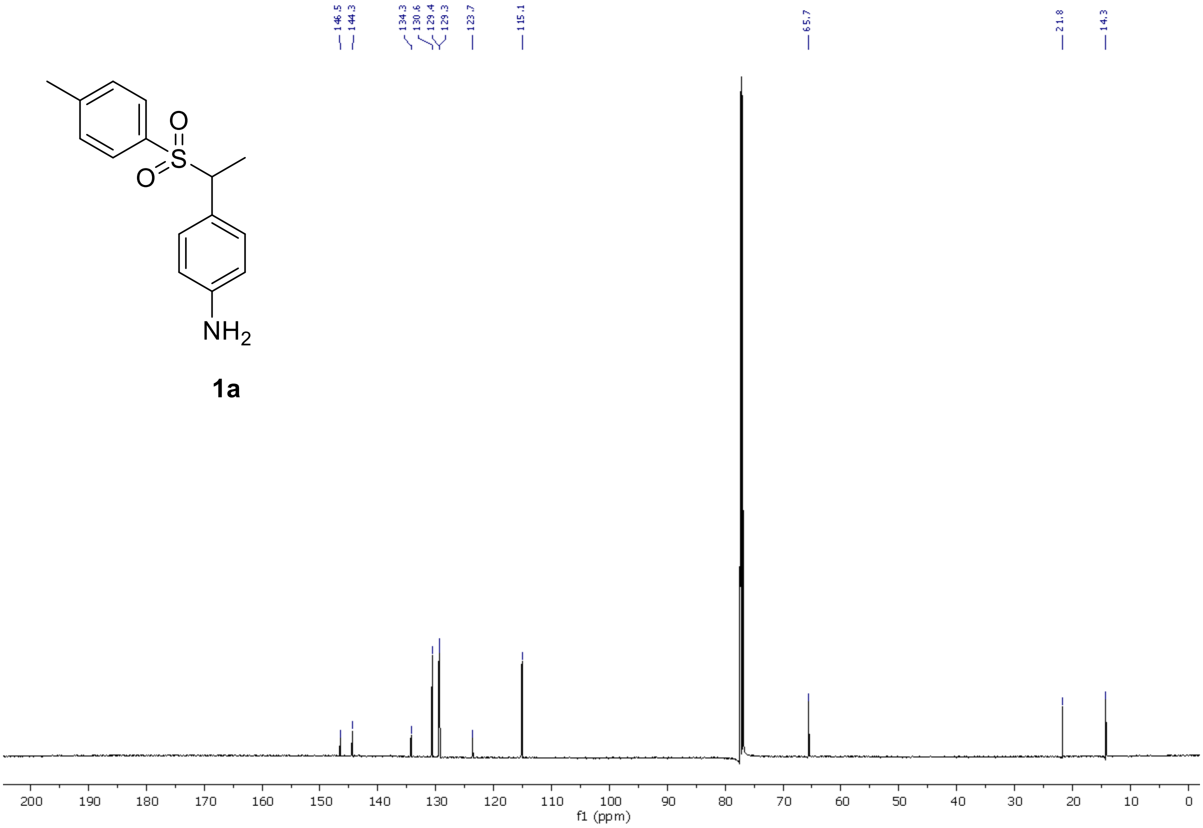

1aa ^{1}H NMR (600 MHz, $CDCl_3$)

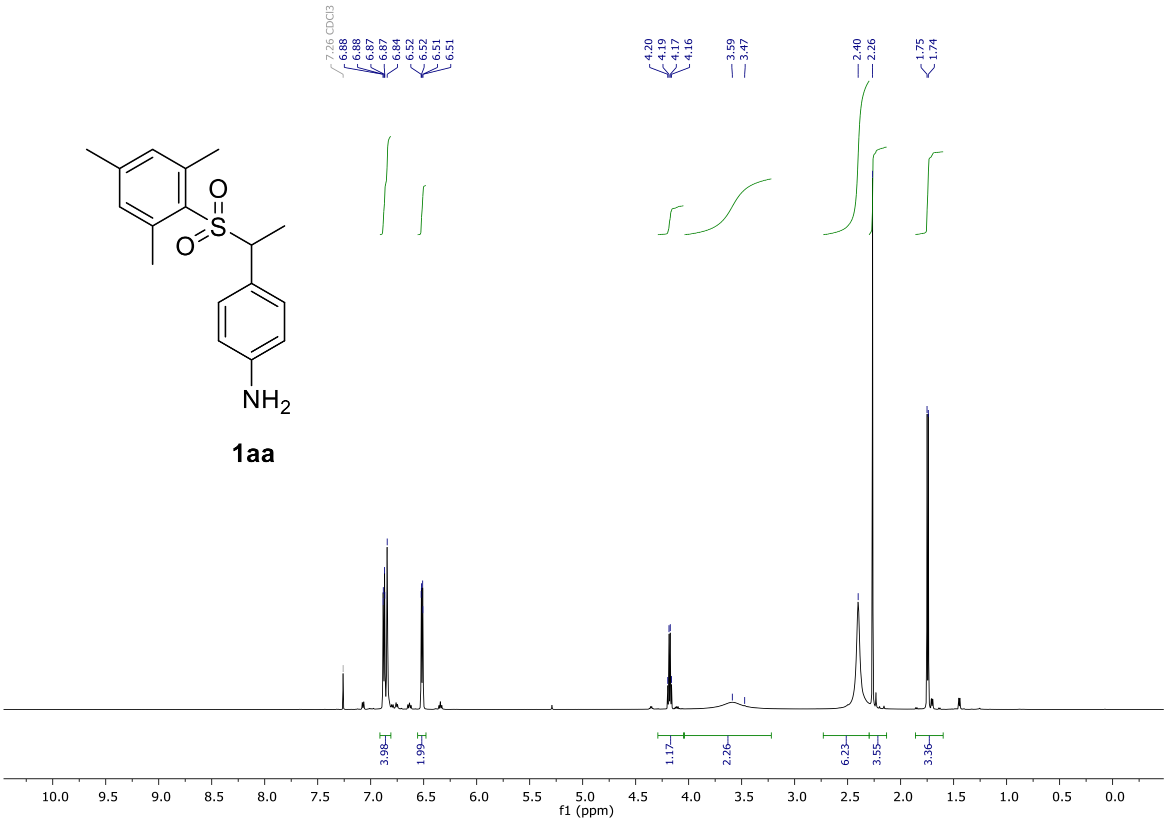

1aa ^{13}C NMR (151 MHz, $CDCl_3$)

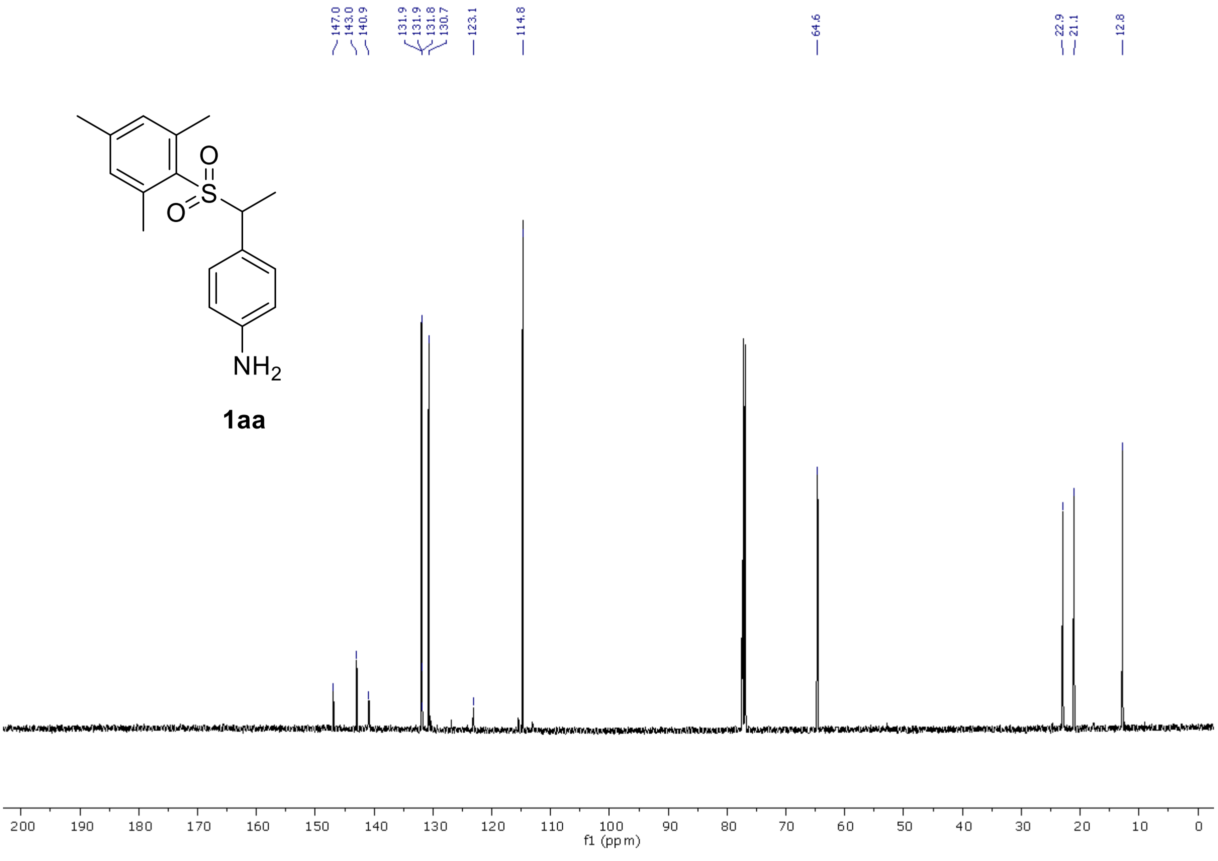

1ab ^{1}H NMR (600 MHz, $CDCl_3$)

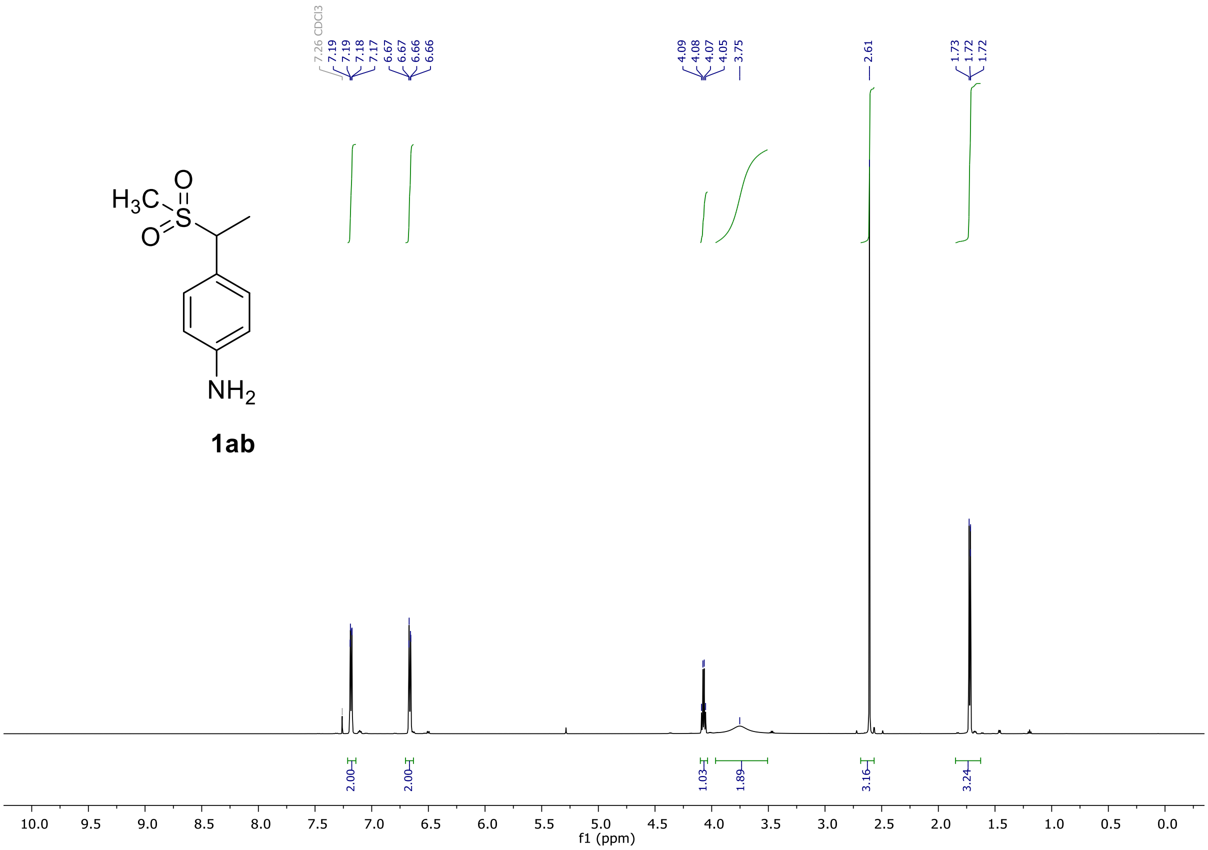

1ab ^{13}C NMR (151 MHz, $CDCl_3$)

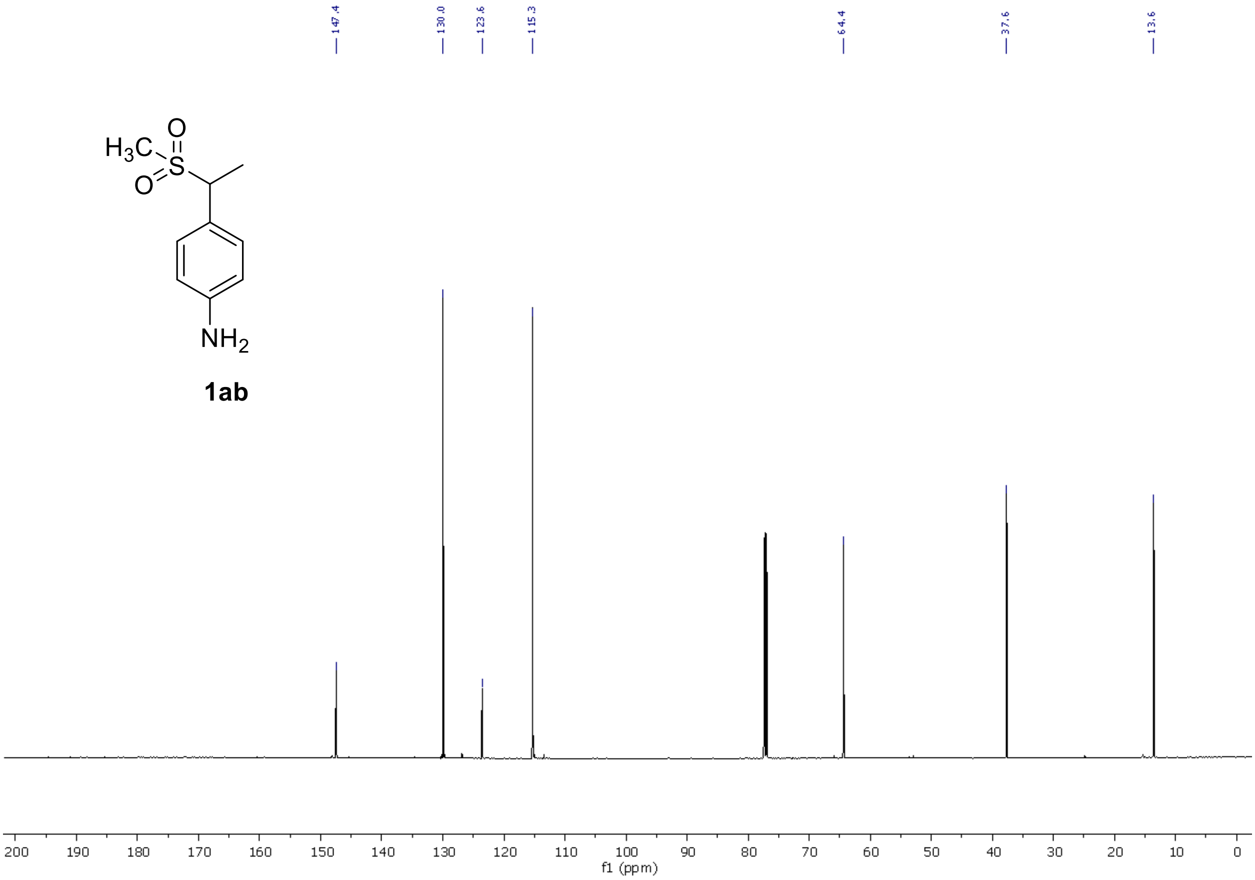

1’t ^{1}H NMR (400 MHz, $CDCl_3$)

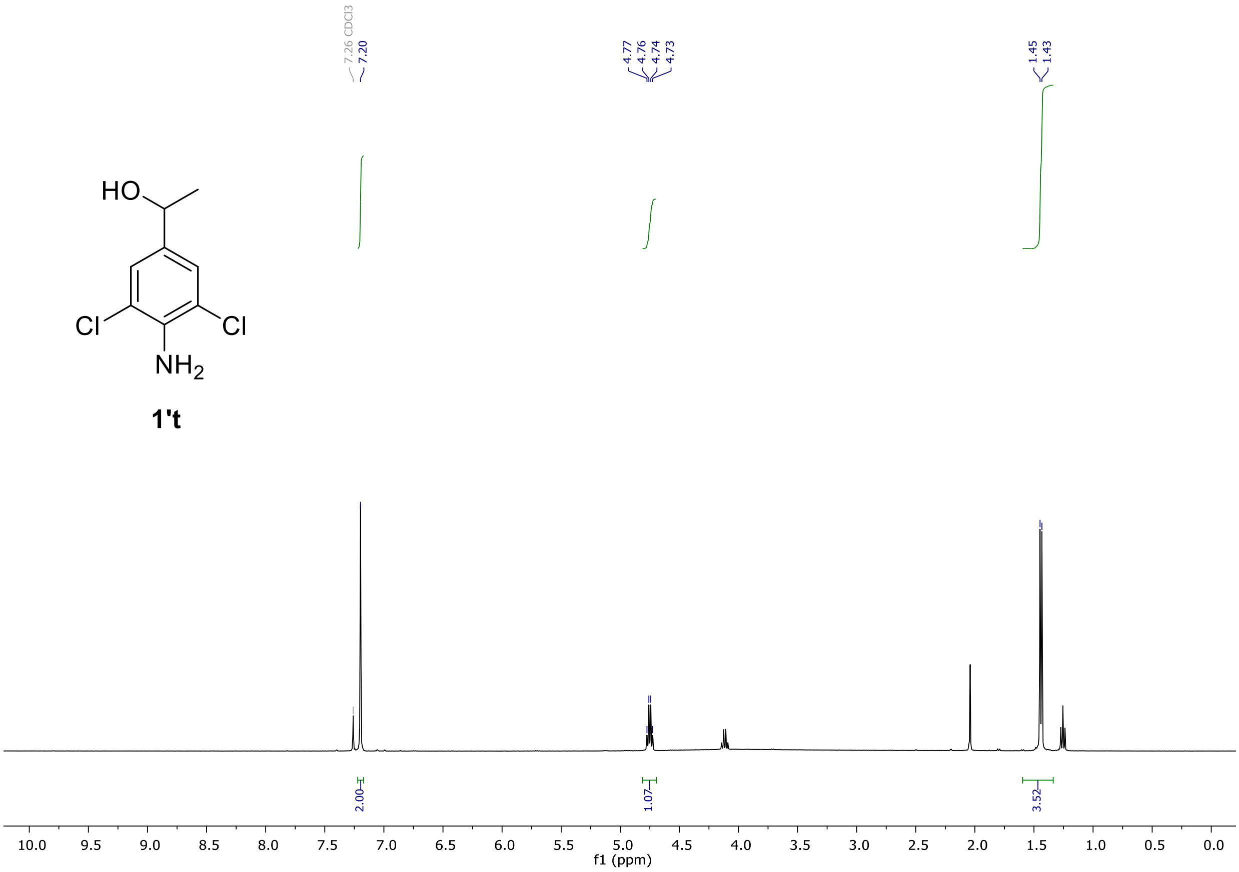

1’t ^{13}C NMR (151 MHz, $CDCl_3$)

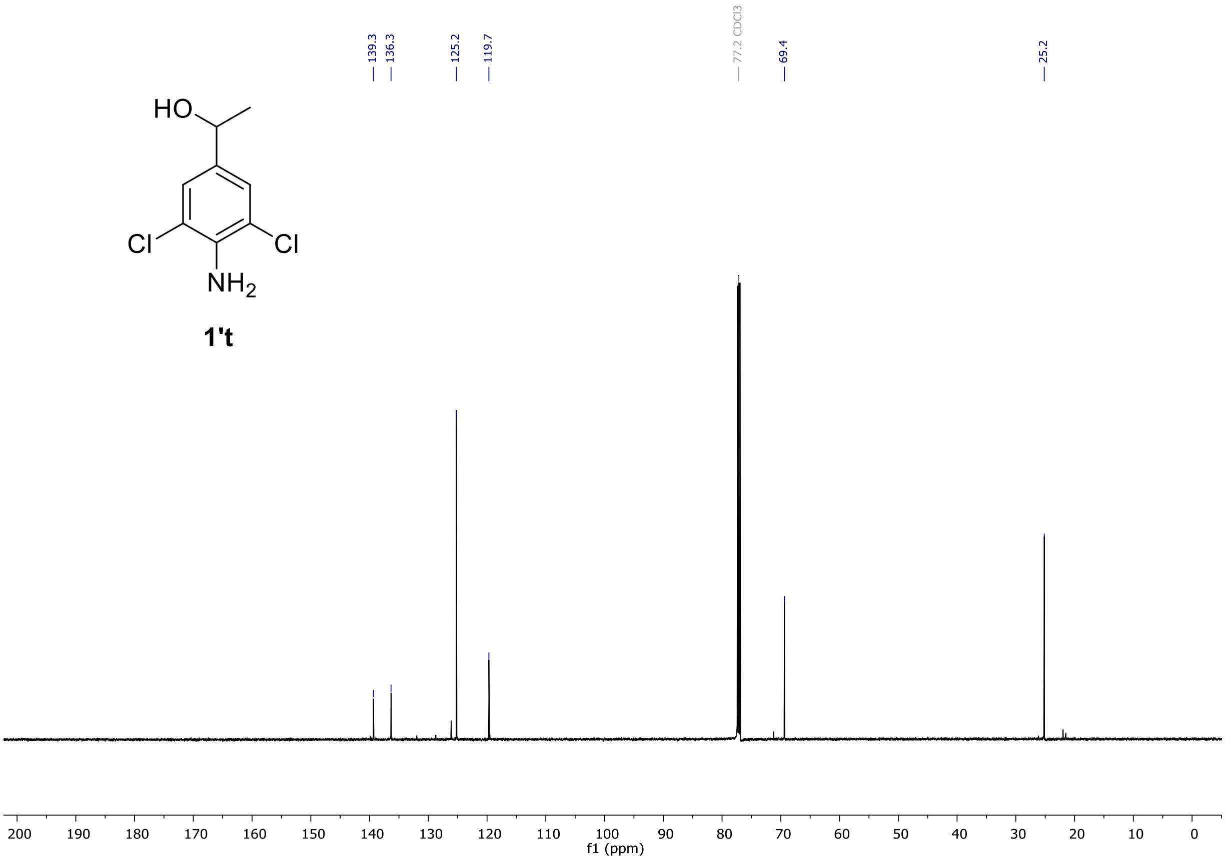

1t ^{1}H NMR (600 MHz, $CDCl_3$)

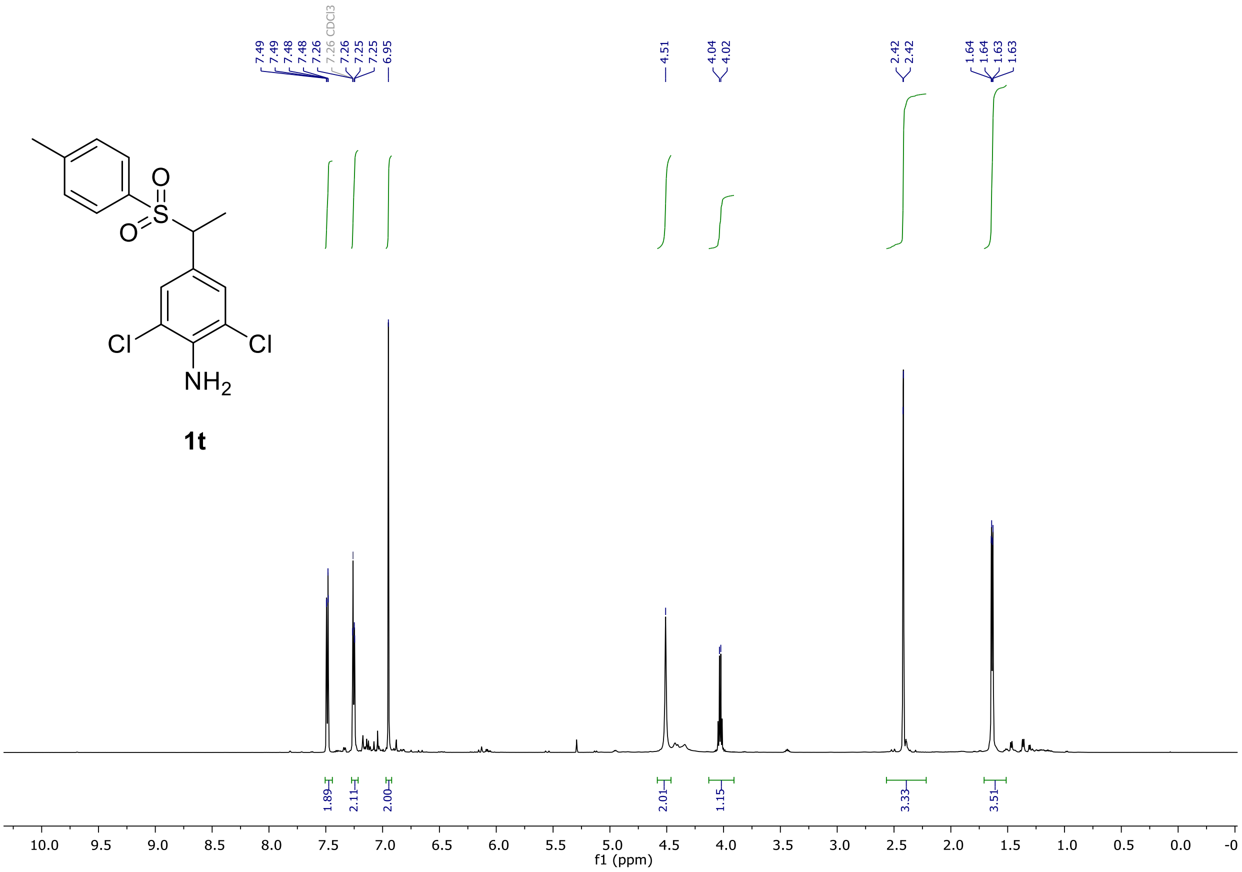

1t ^{13}C NMR (151 MHz, $CDCl_3$)

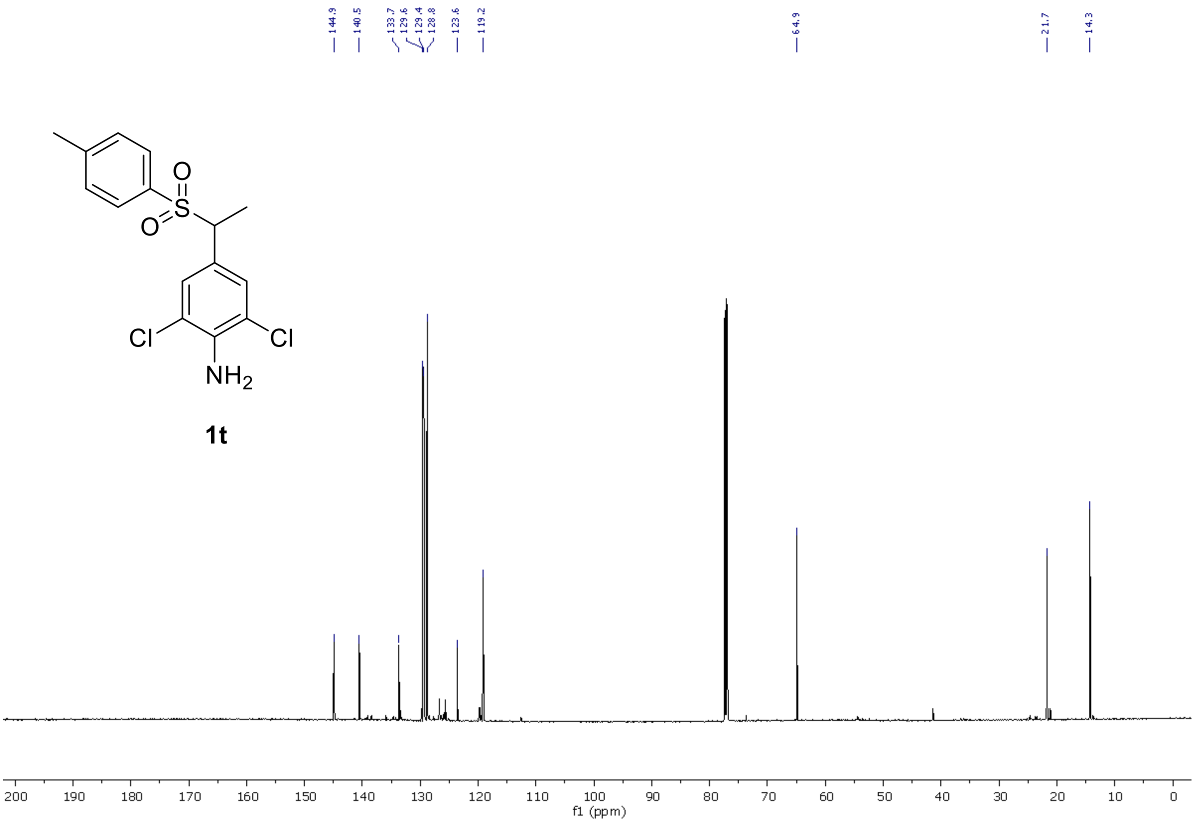

2b ^{1}H NMR (600 MHz, $CDCl_3$)

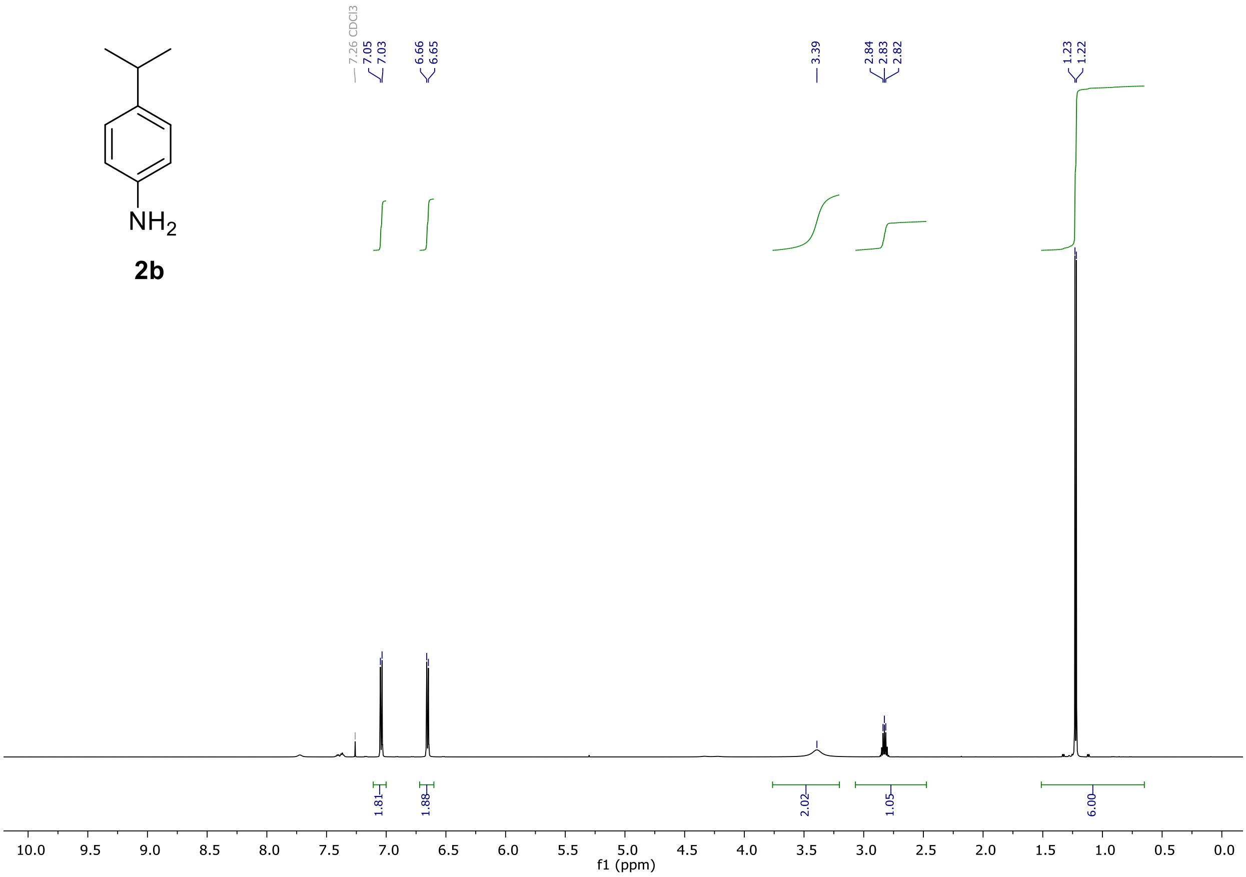

2b ^{13}C NMR (151 MHz, $CDCl_3$)

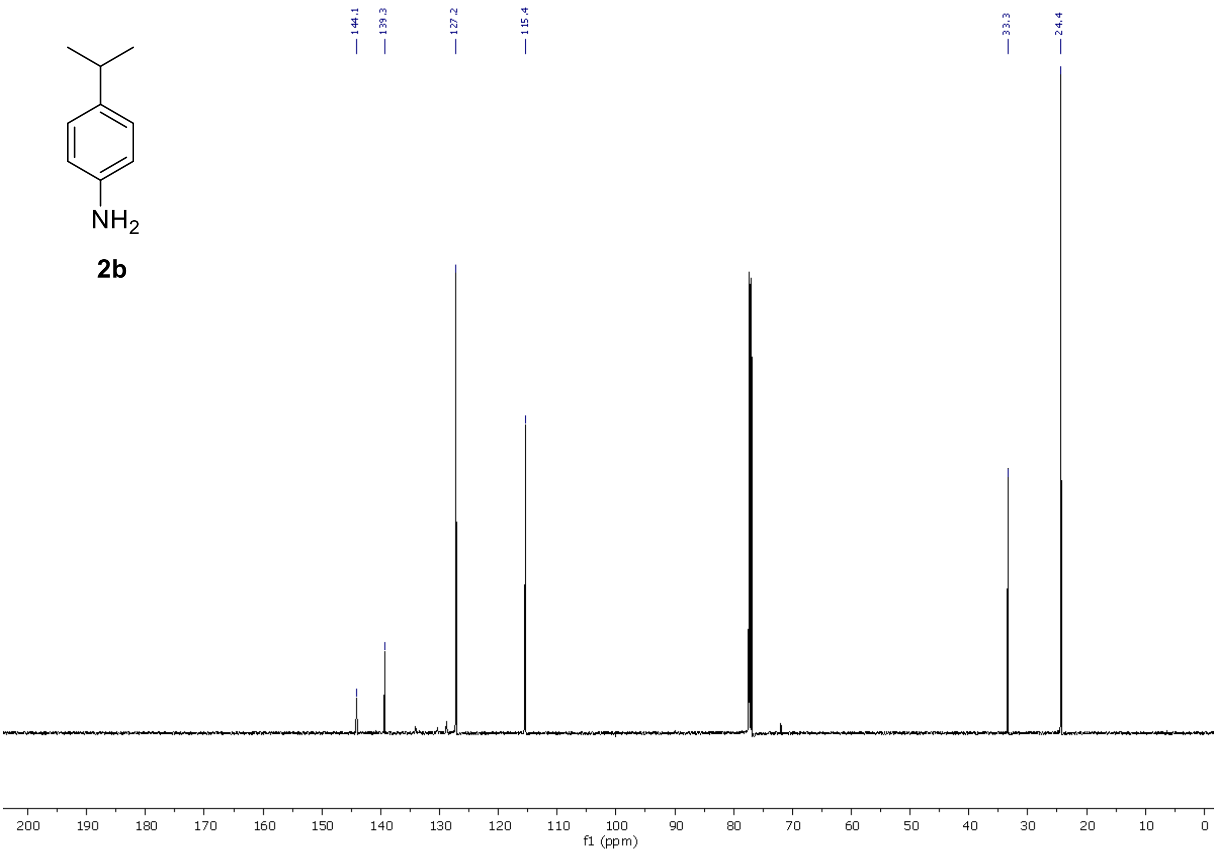

2c ^{1}H NMR (600 MHz, $CDCl_3$)

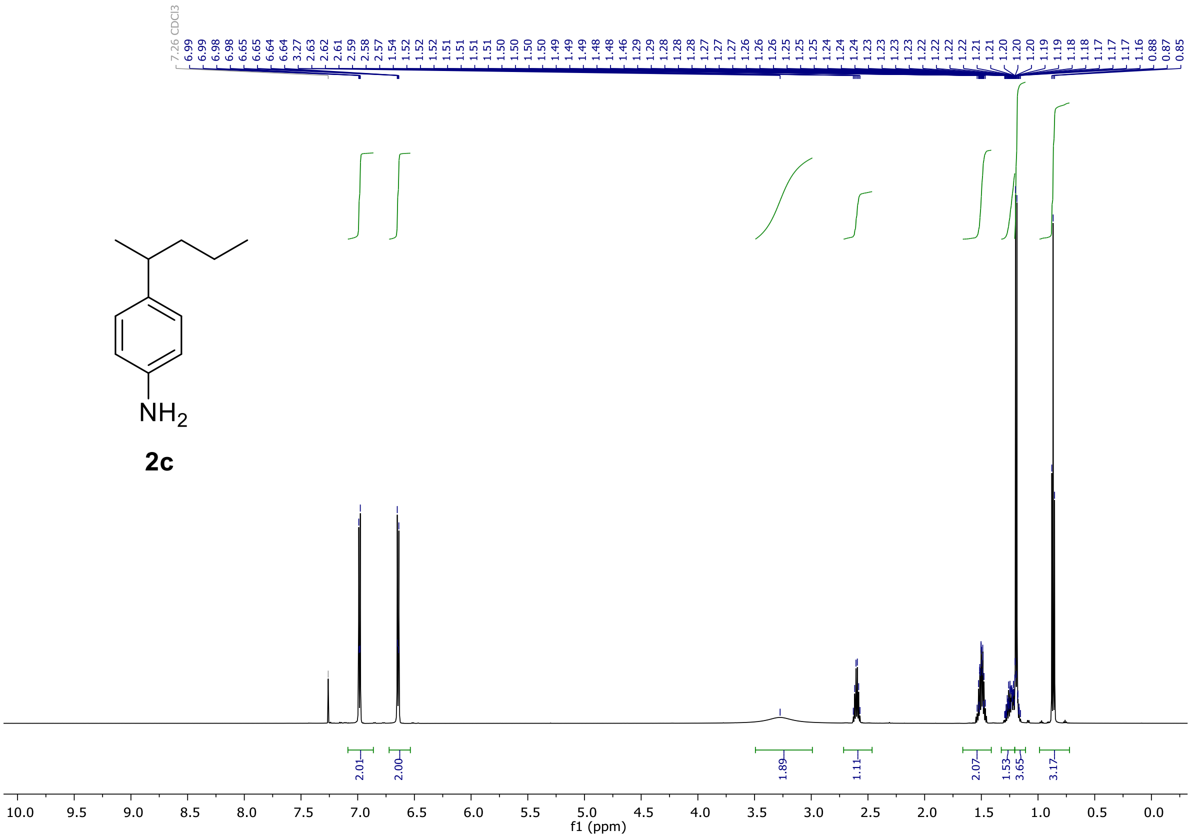

2c ^{13}C NMR (151 MHz, $CDCl_3$)

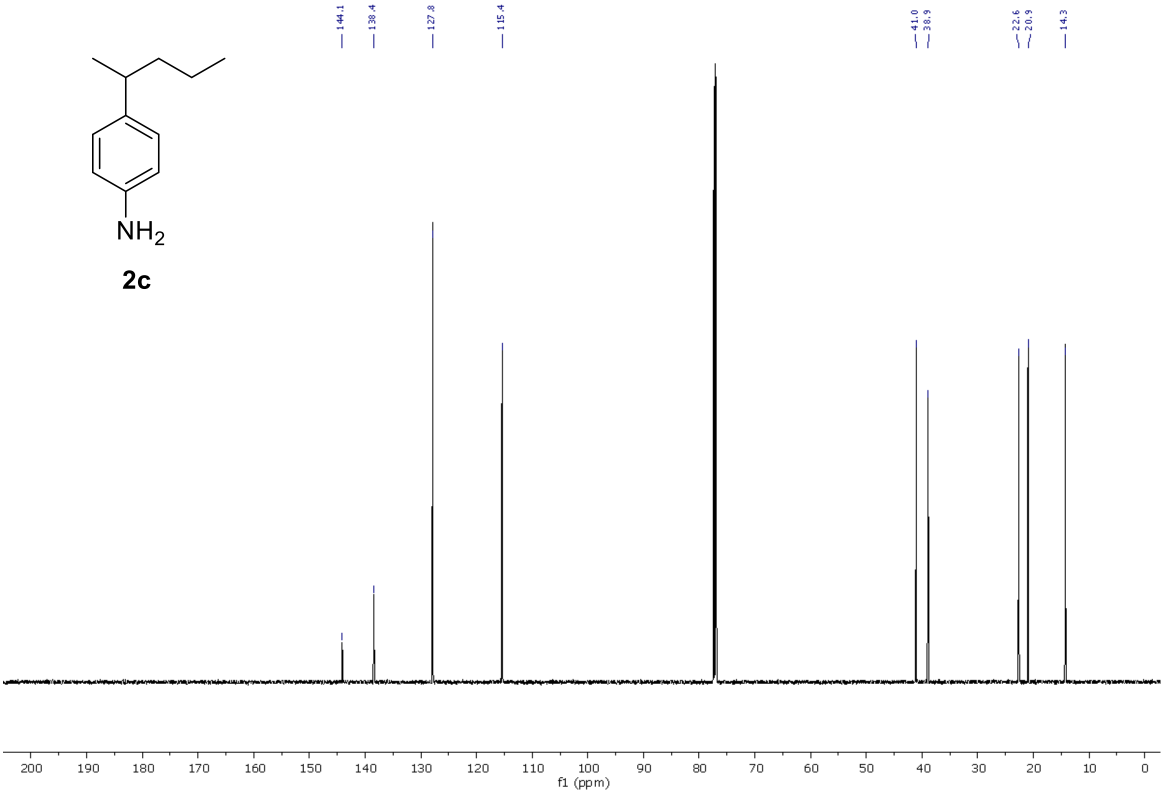

2e ^{1}H NMR (600 MHz, $CDCl_3$)

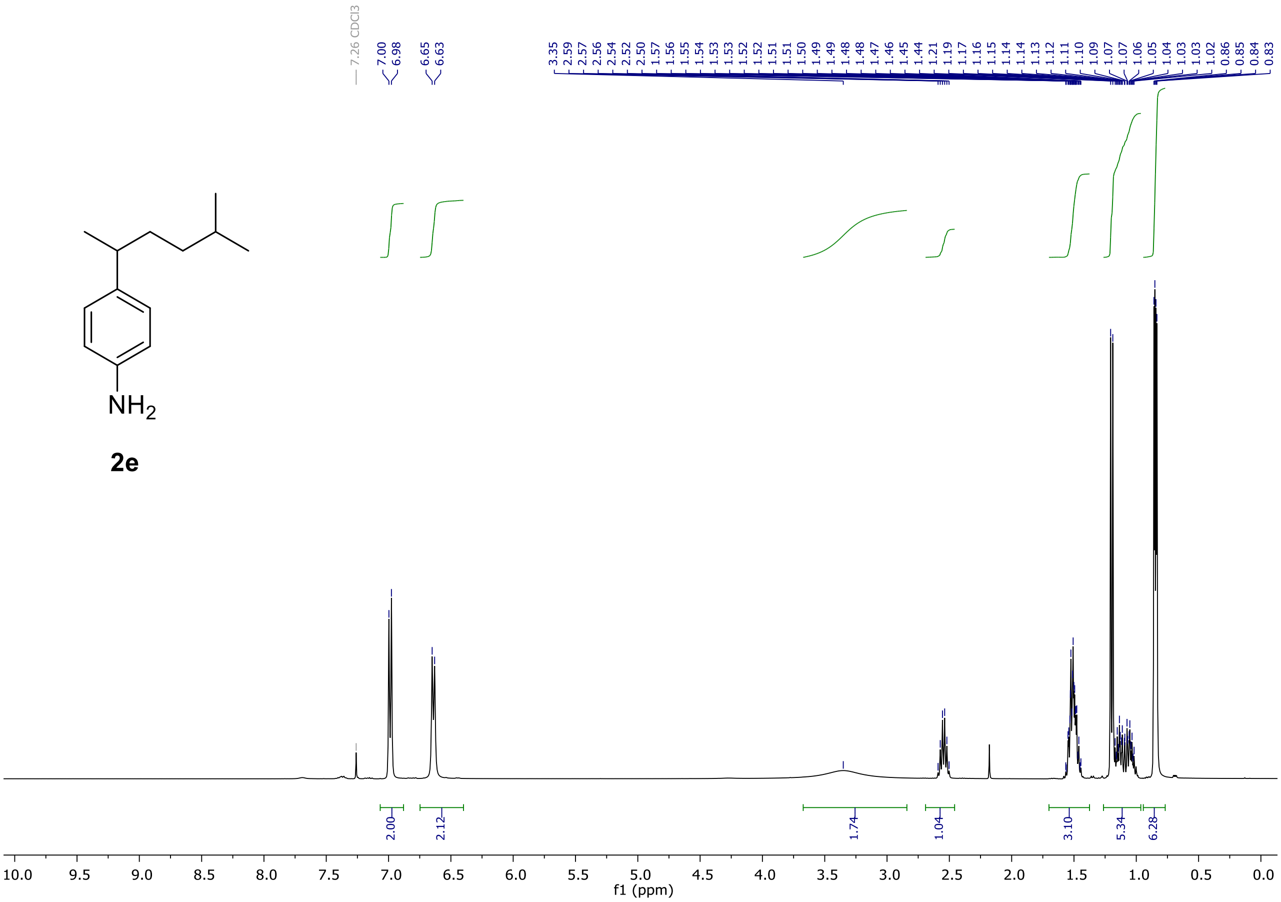

2e ^{13}C NMR (151 MHz, $CDCl_3$)

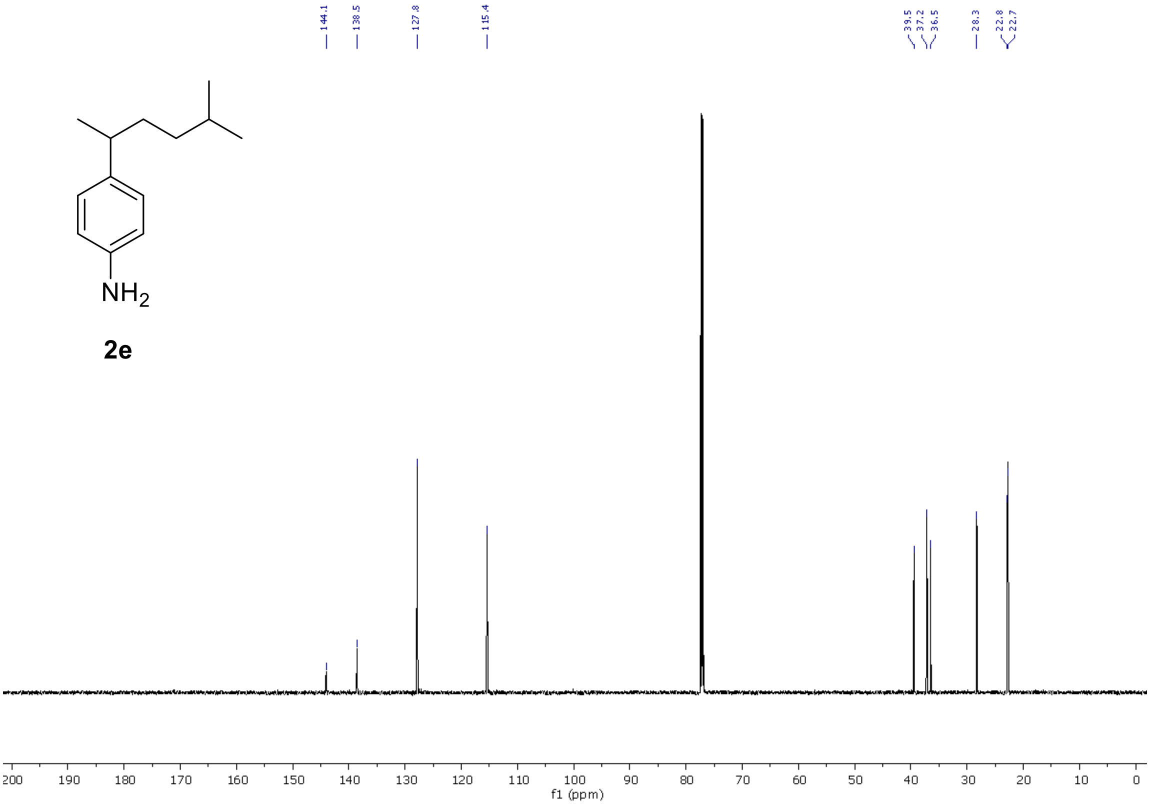

2f ^{1}H NMR (600 MHz, $CDCl_3$)

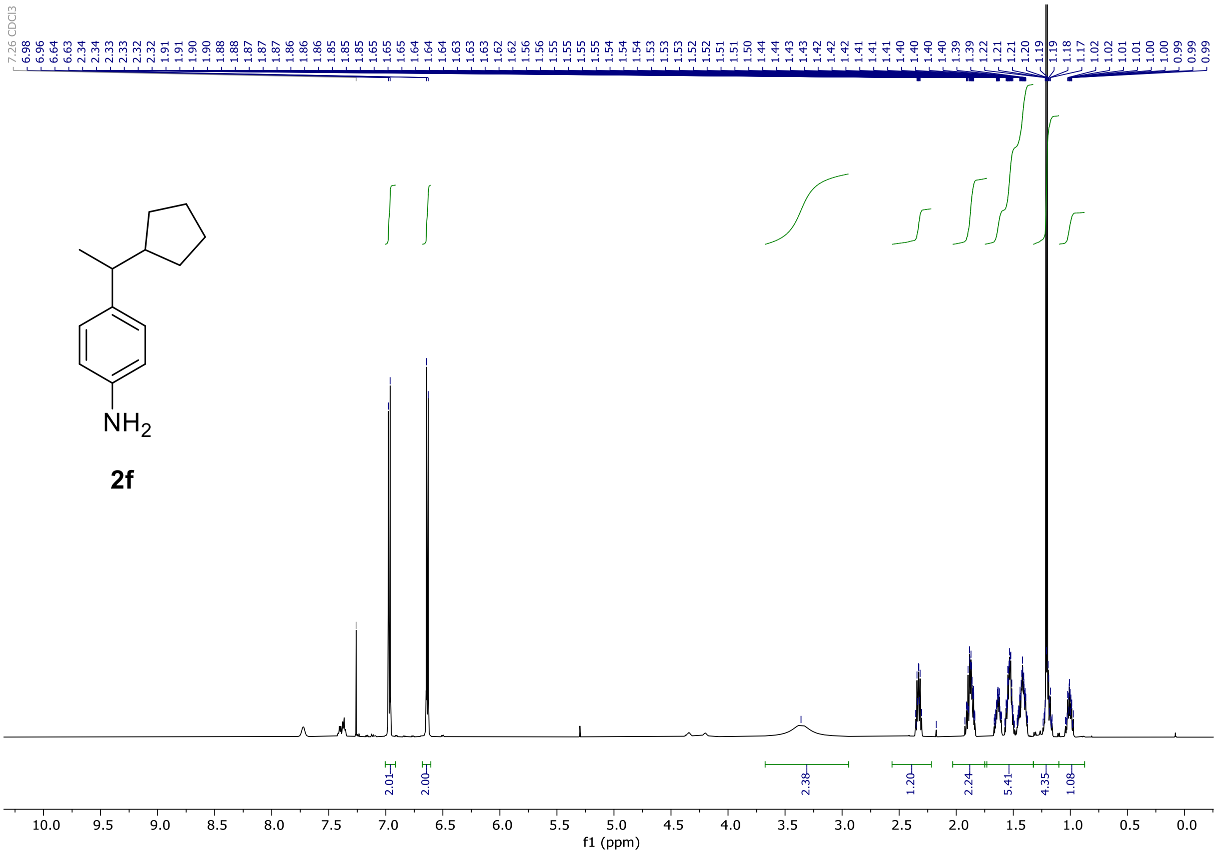

2f ^{13}C NMR (151 MHz, $CDCl_3$)

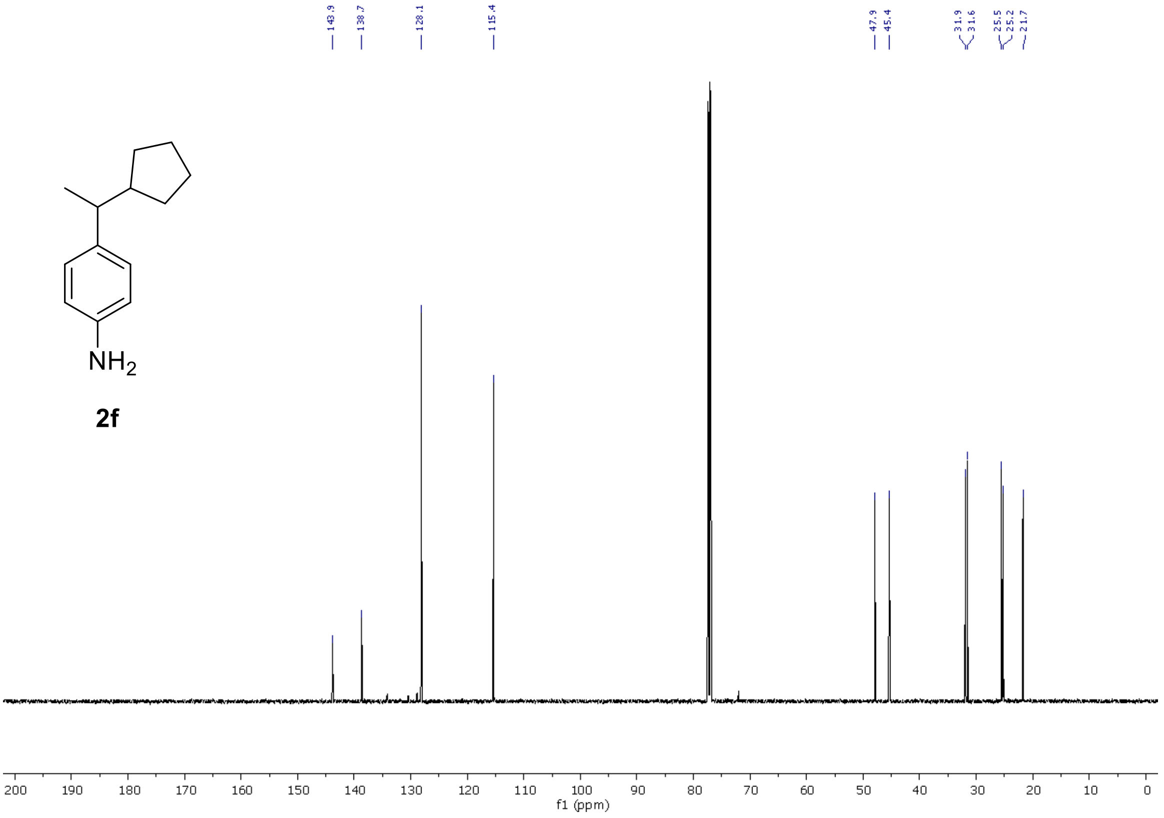

2g ¹H NMR (600 MHz, $CDCl_3$)

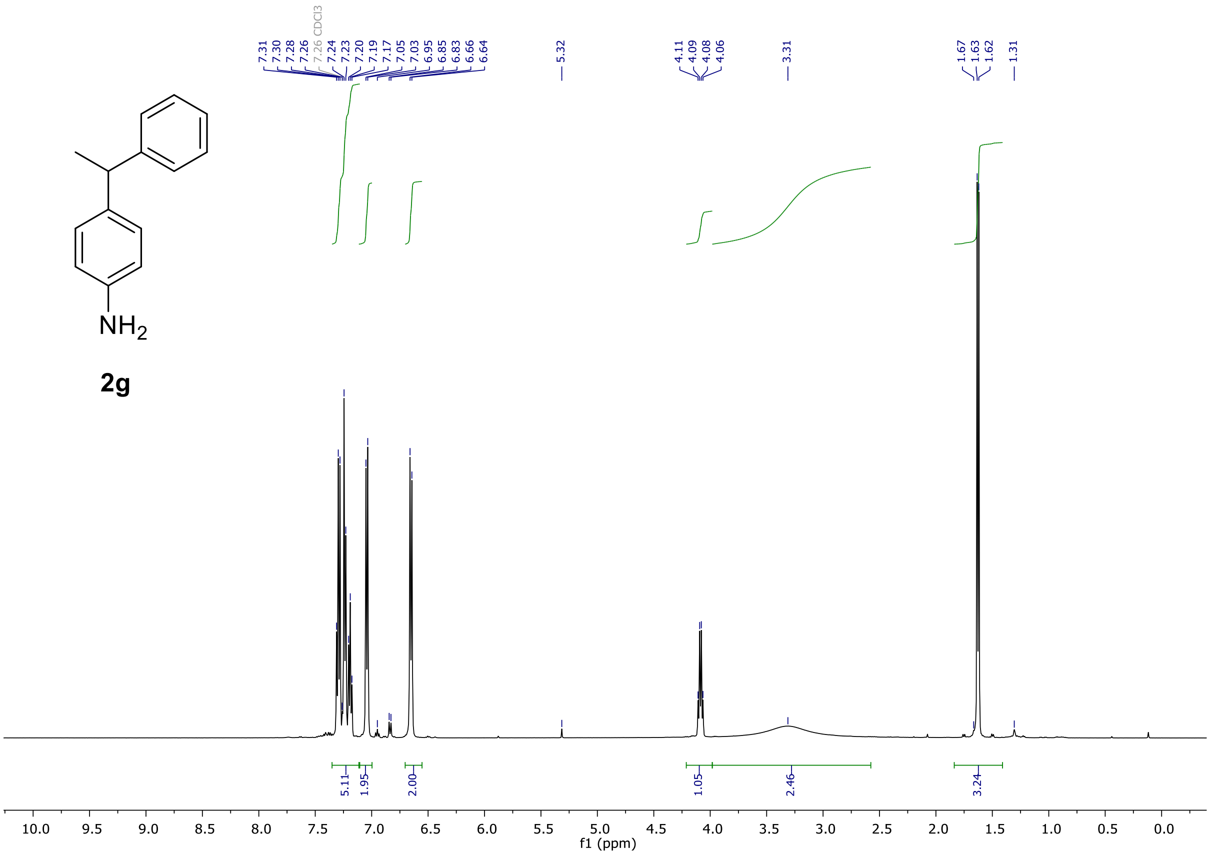

2g ¹³C NMR (151 MHz, $CDCl_3$)

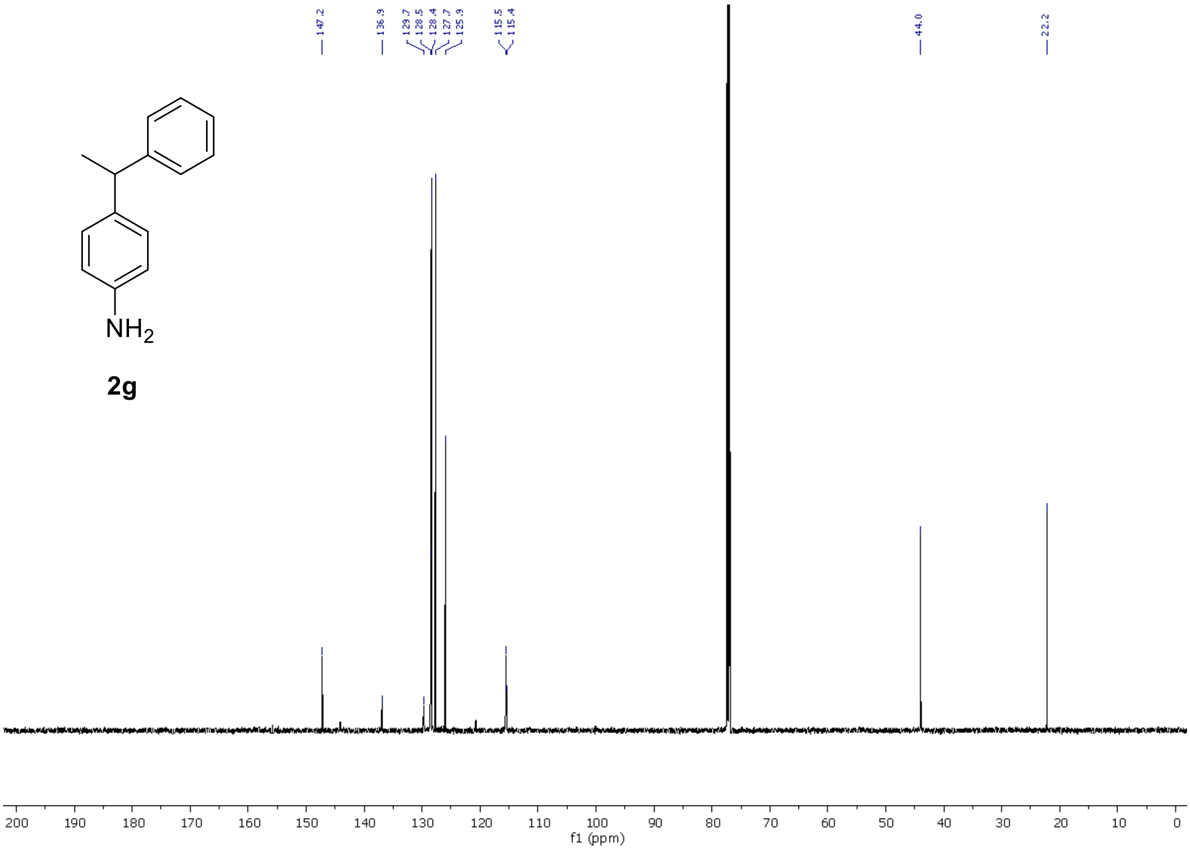

2h ^{1}H NMR (600 MHz, $CDCl_3$)

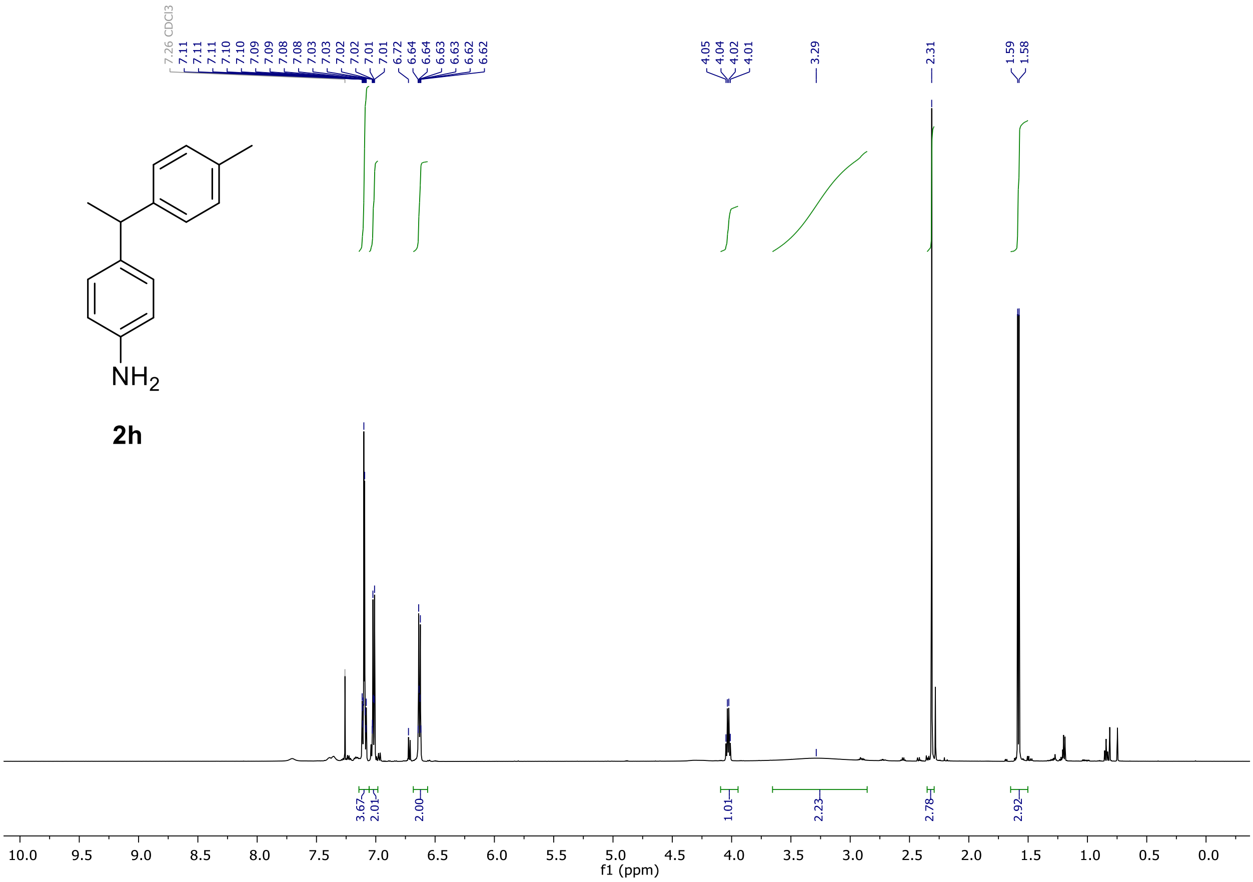

2h ^{13}C NMR (151 MHz, $CDCl_3$)

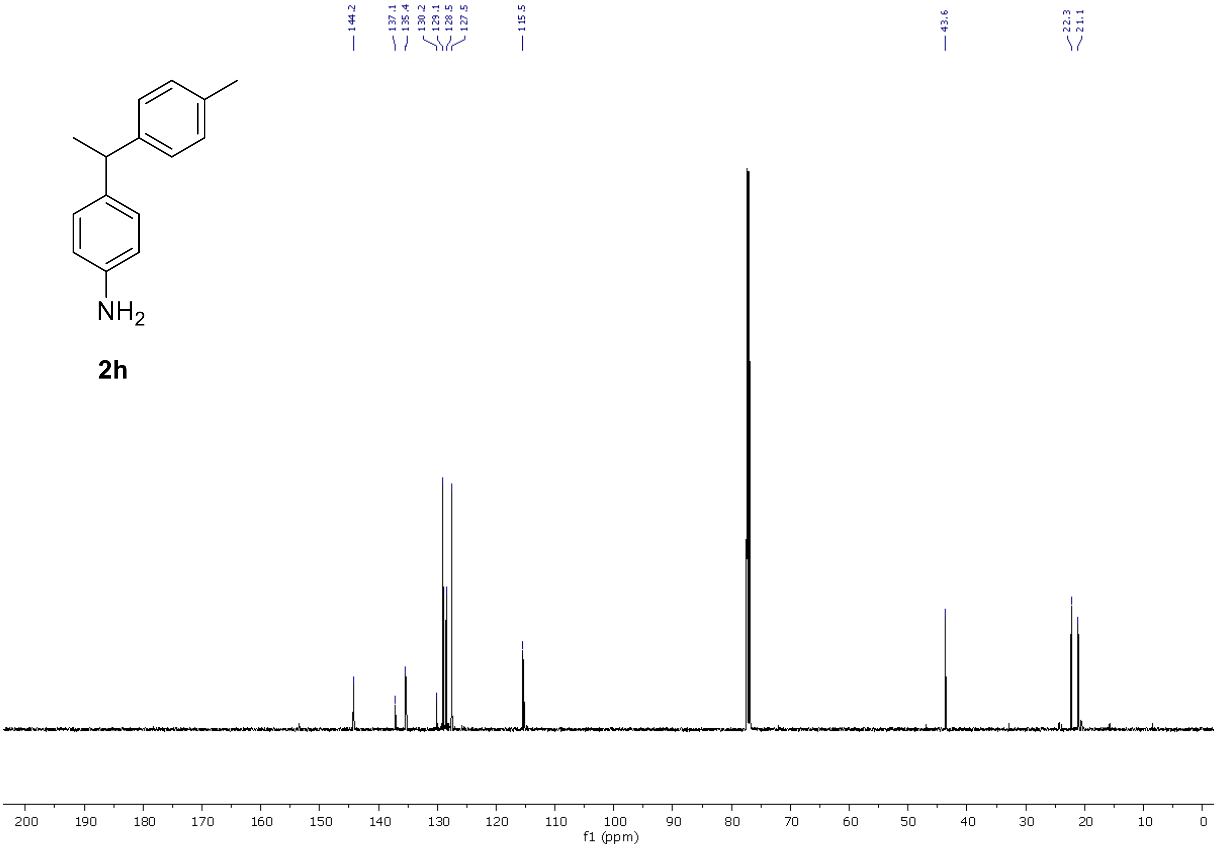

2i ^{1}H NMR (600 MHz, $CDCl_3$)

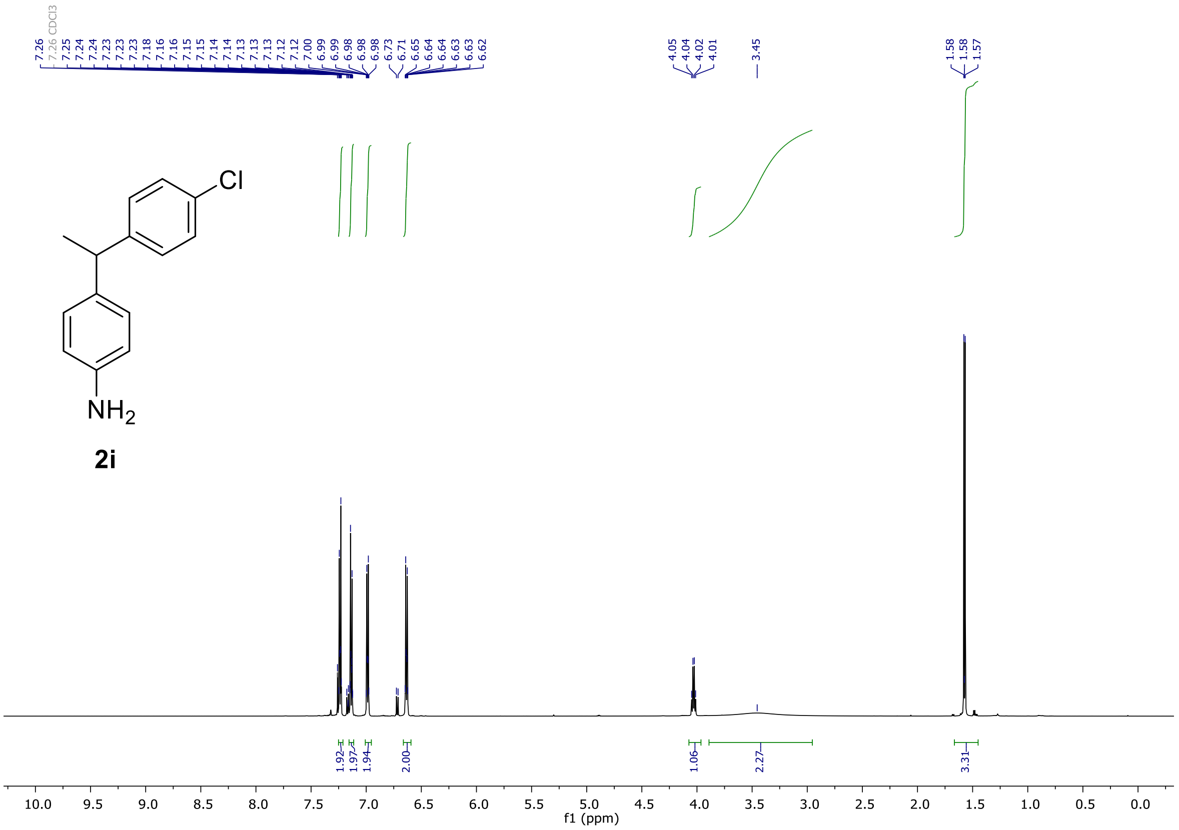

2i ^{13}C NMR (151 MHz, $CDCl_3$)

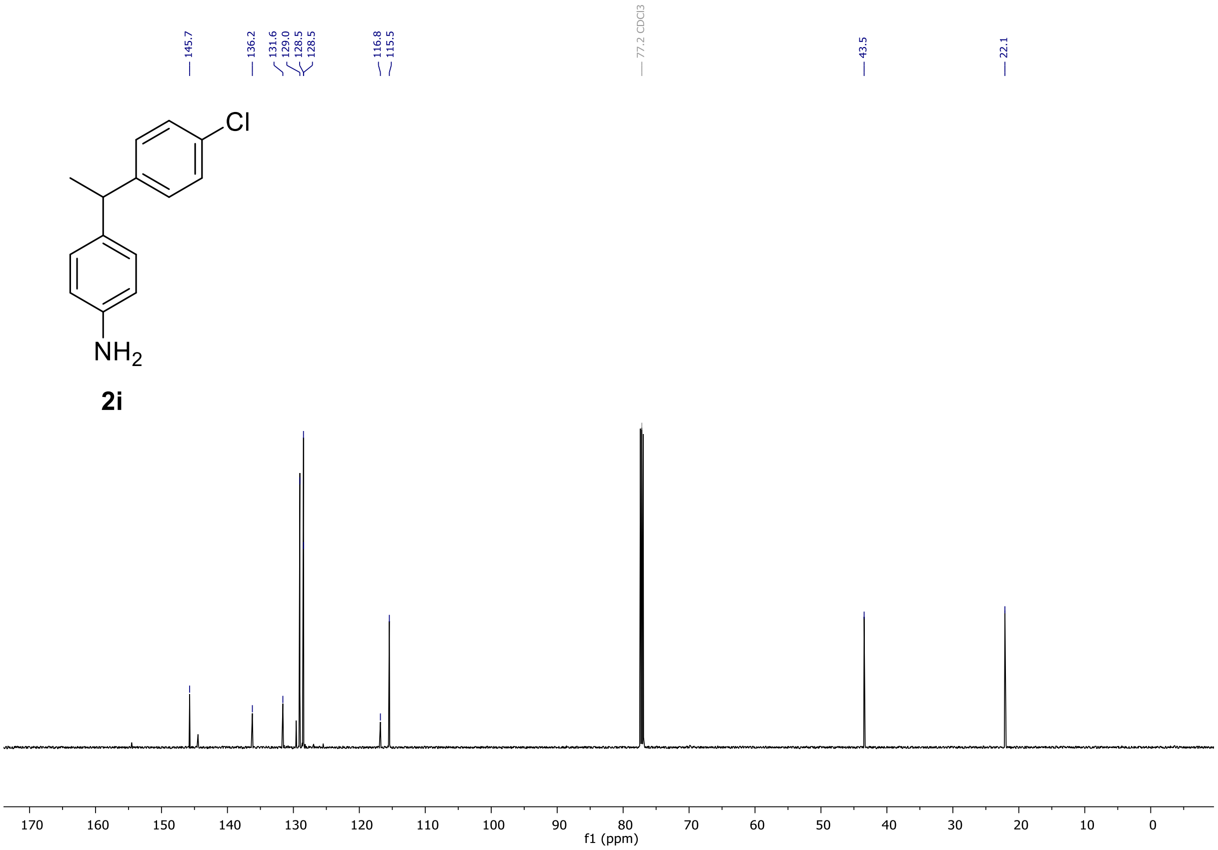

2j ^{1}H NMR (600 MHz, $CDCl_3$)

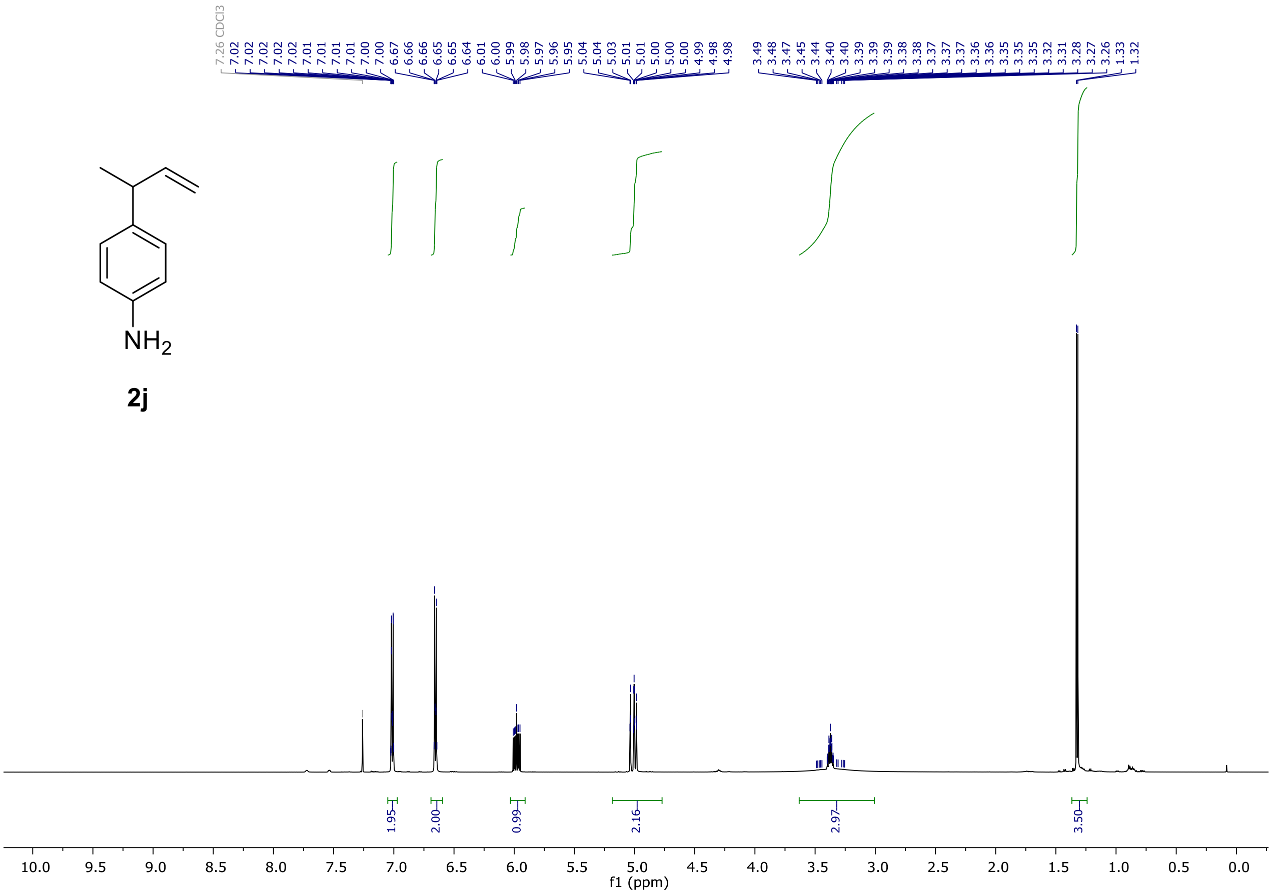

2j ^{13}C NMR (151 MHz, $CDCl_3$)

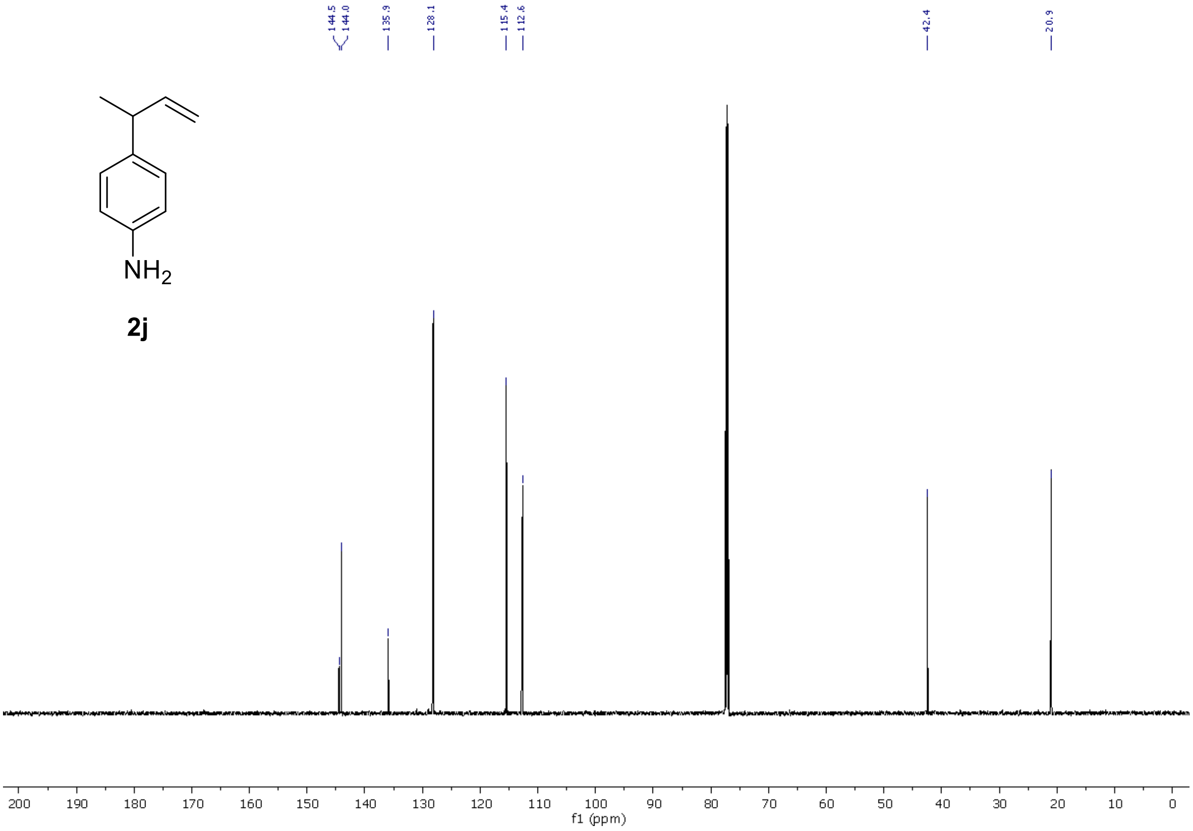

2k ^{1}H NMR (600 MHz, $CDCl_3$)

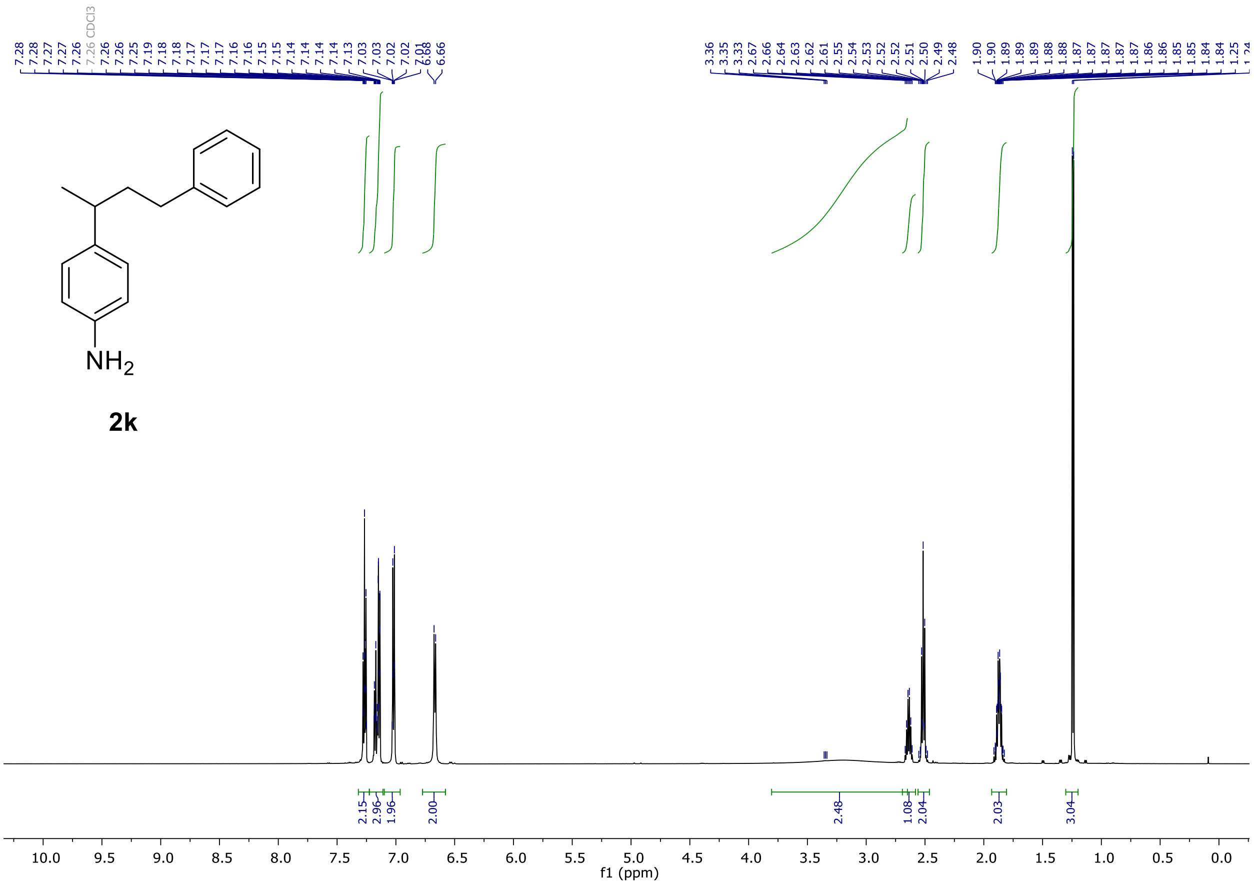

2k ^{13}C NMR (151 MHz, $CDCl_3$)

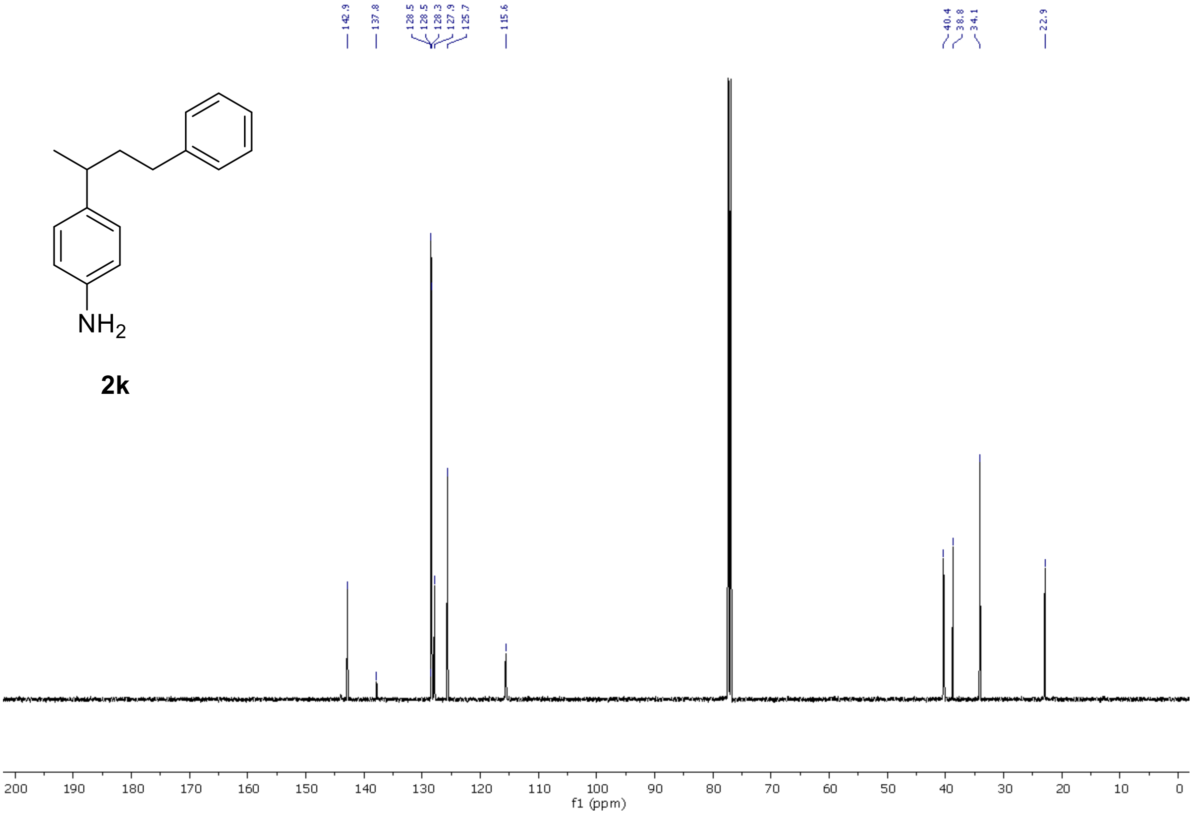

2l ^{1}H NMR (600 MHz, $CDCl_3$)

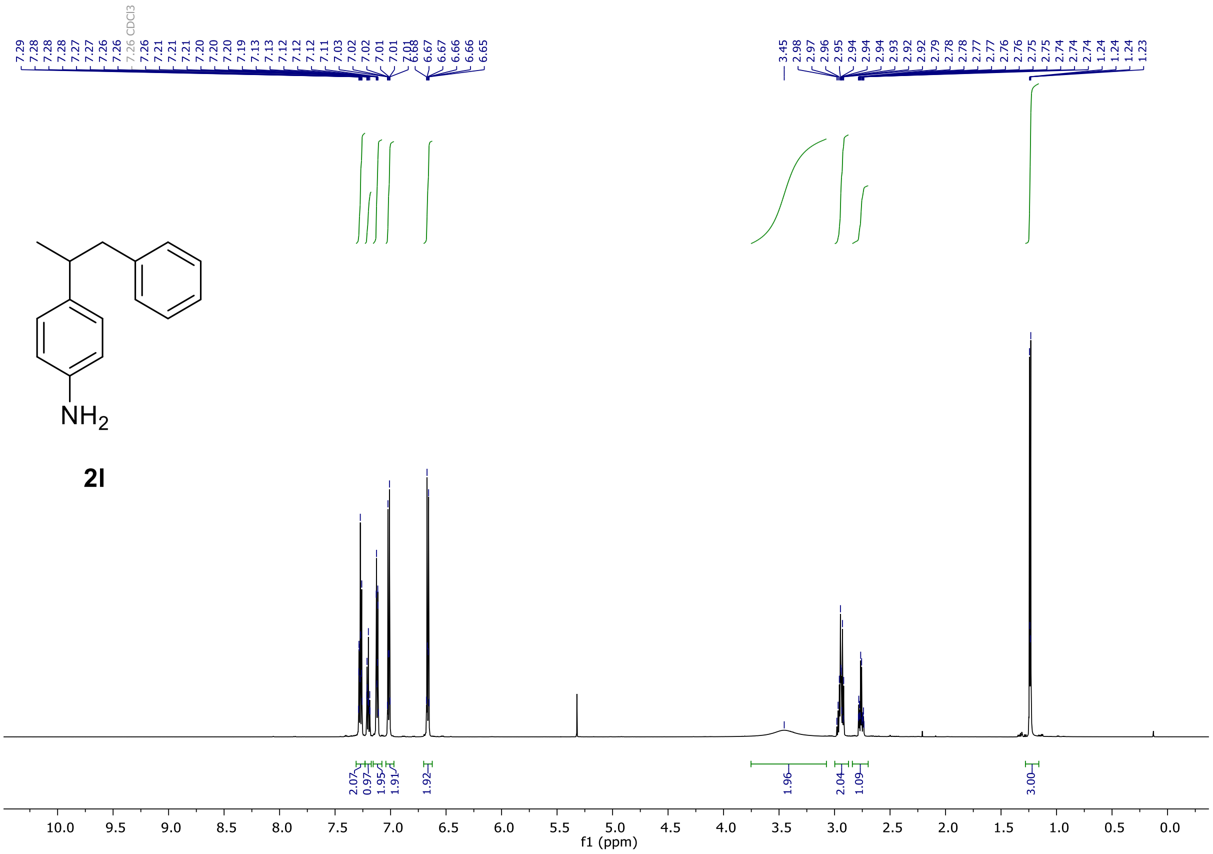

2l ^{13}C NMR (151 MHz, $CDCl_3$)

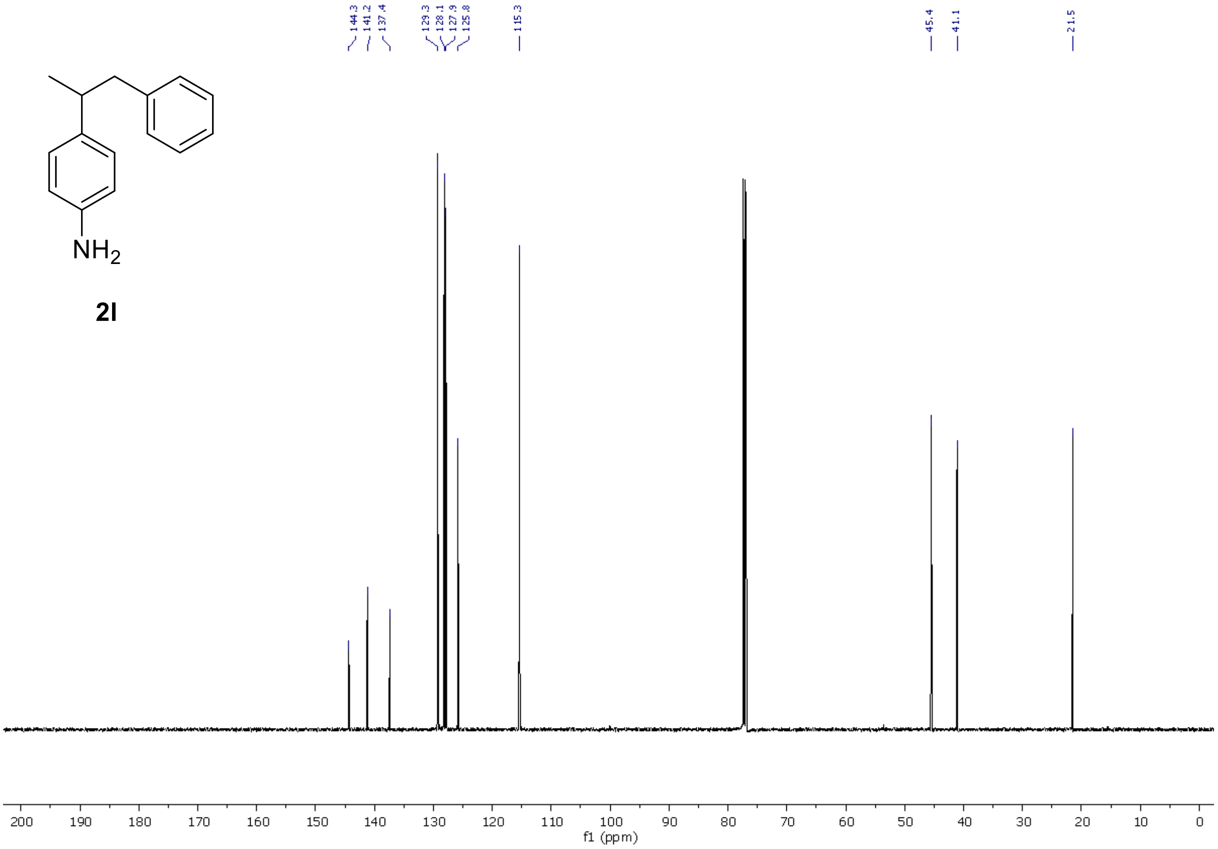

2m ^{1}H NMR (600 MHz, $CDCl_3$)

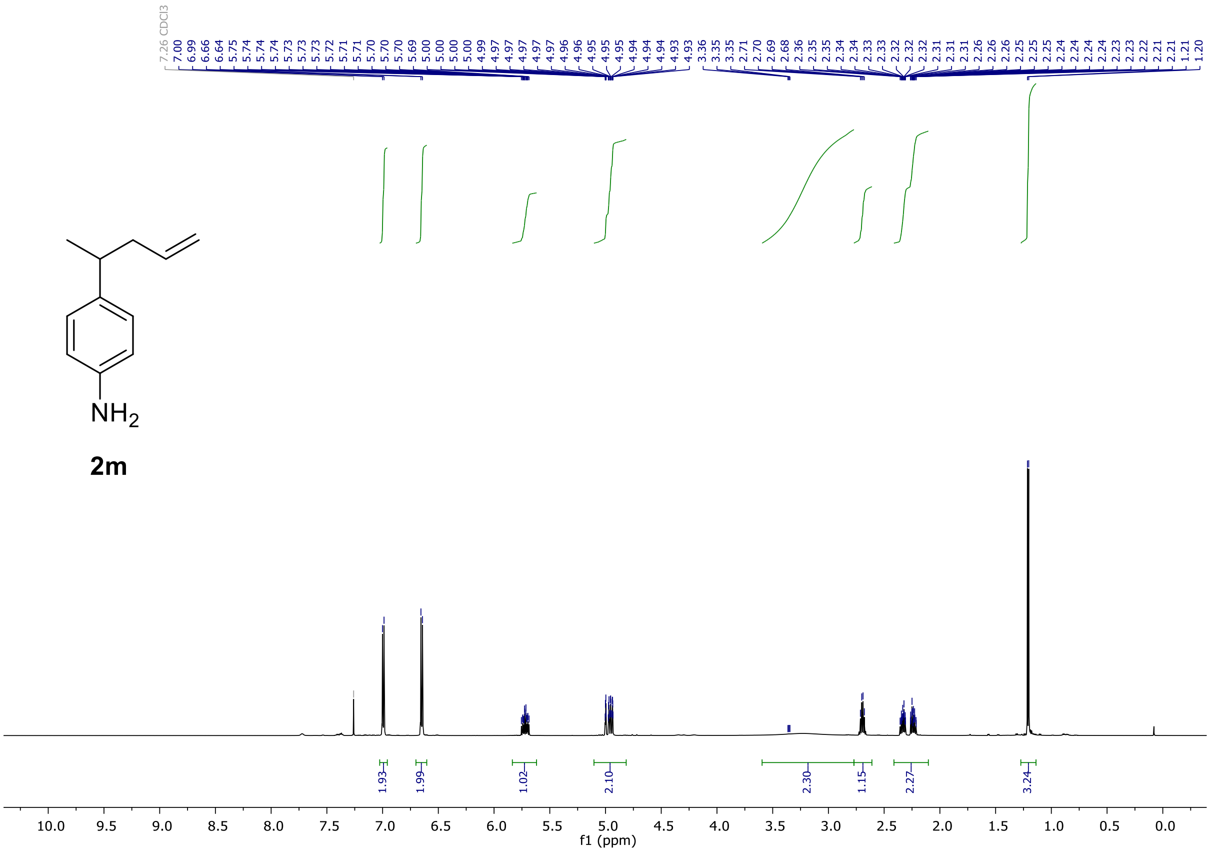

2m ^{13}C NMR (151 MHz, $CDCl_3$)

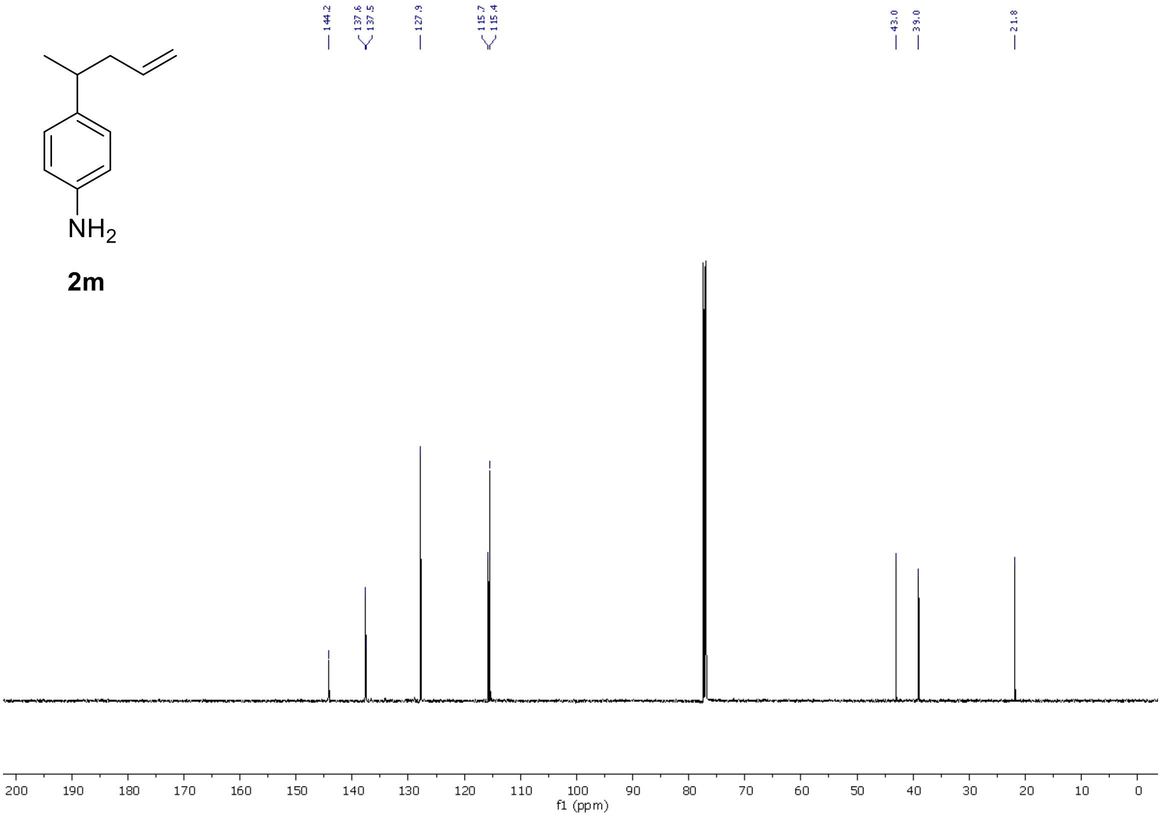

2a ^{1}H NMR (400 MHz, $CDCl_3$)

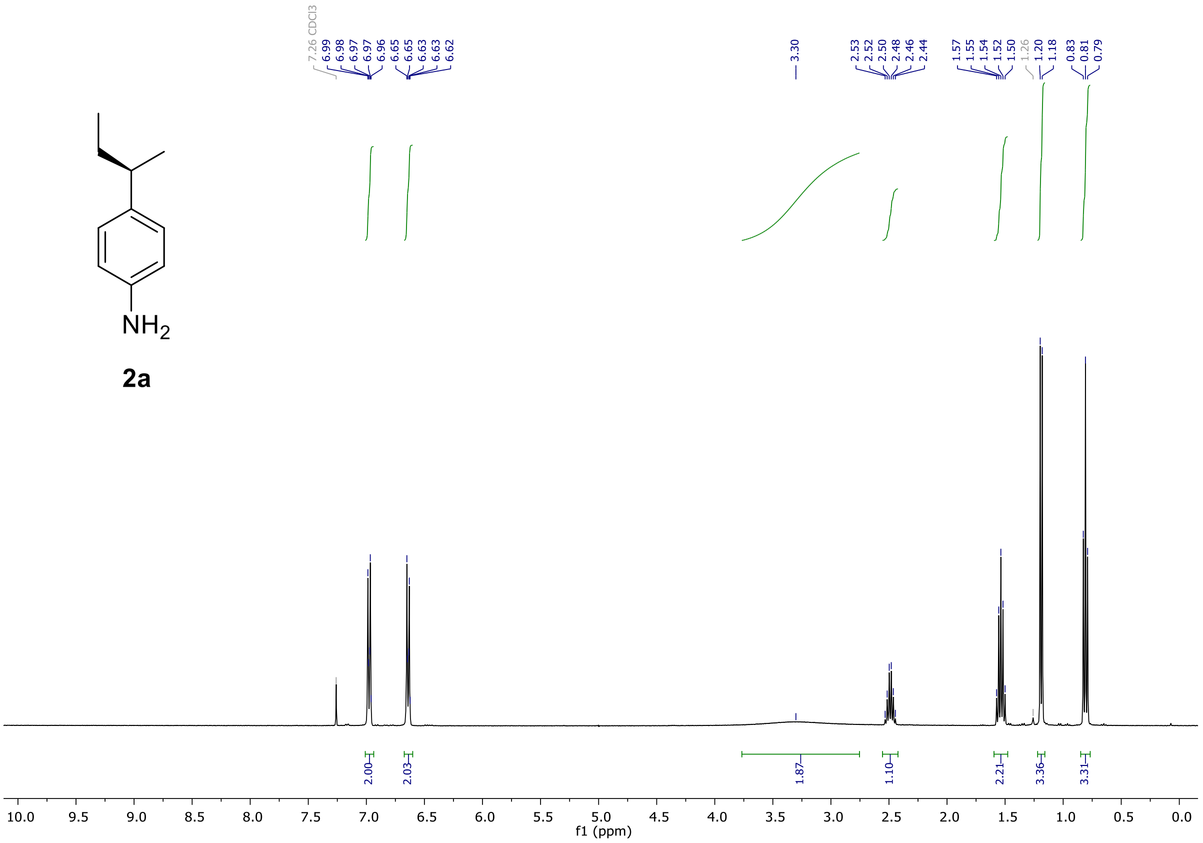

2a ^{13}C NMR (151 MHz, $CDCl_3$)

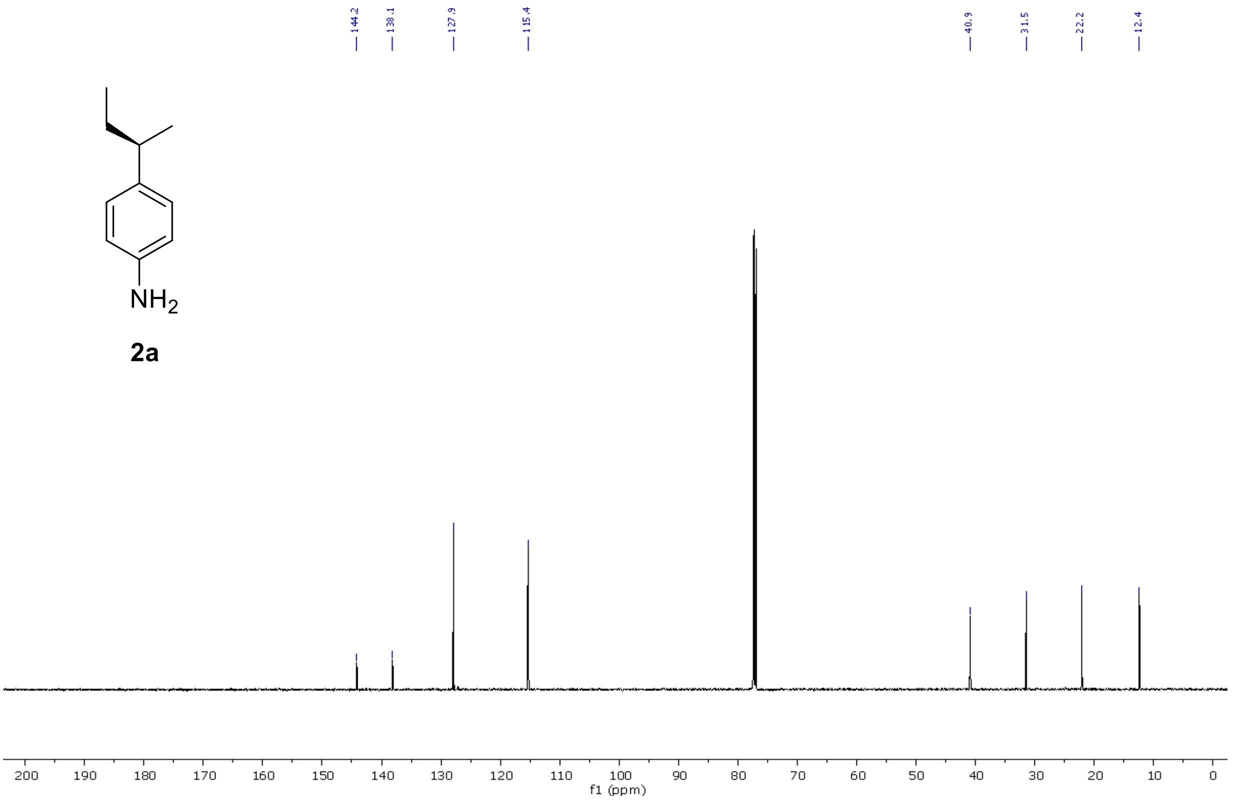

2n ^{1}H NMR (600 MHz, $CDCl_3$)

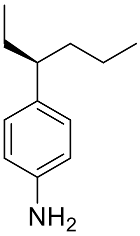

2n

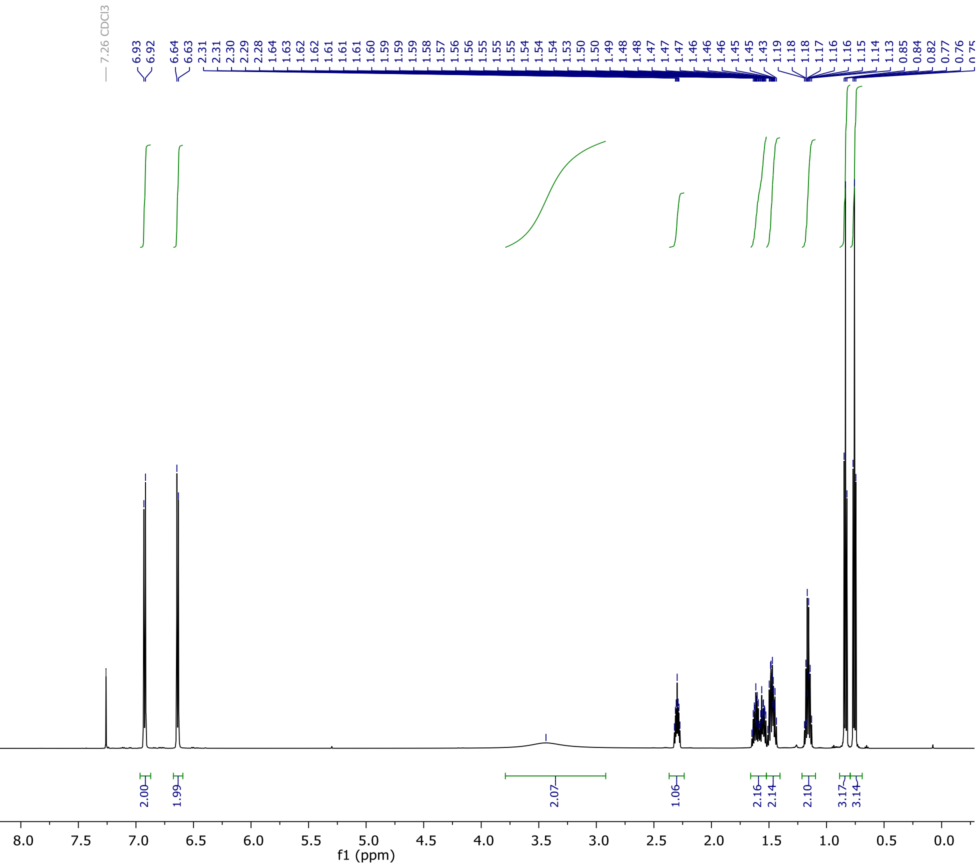

2n ^{13}C NMR (151 MHz, $CDCl_3$)

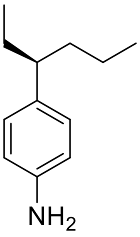

2n

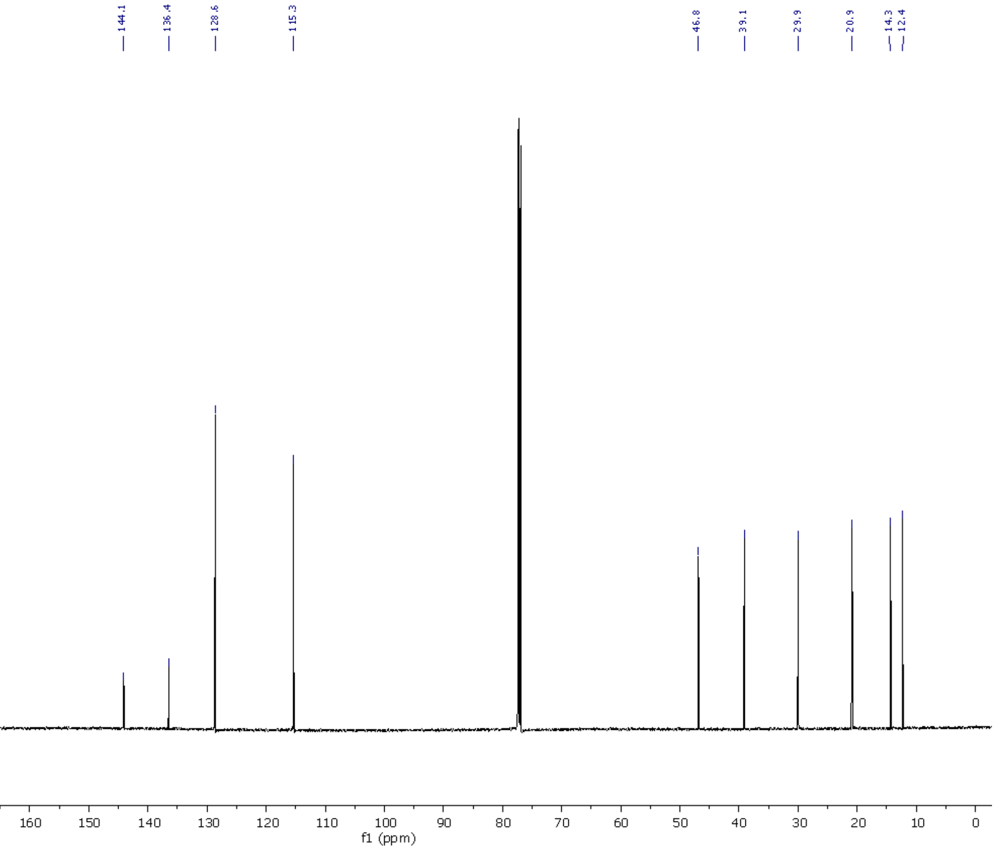

2o ^{1}H NMR (400 MHz, CDCl$_3$)

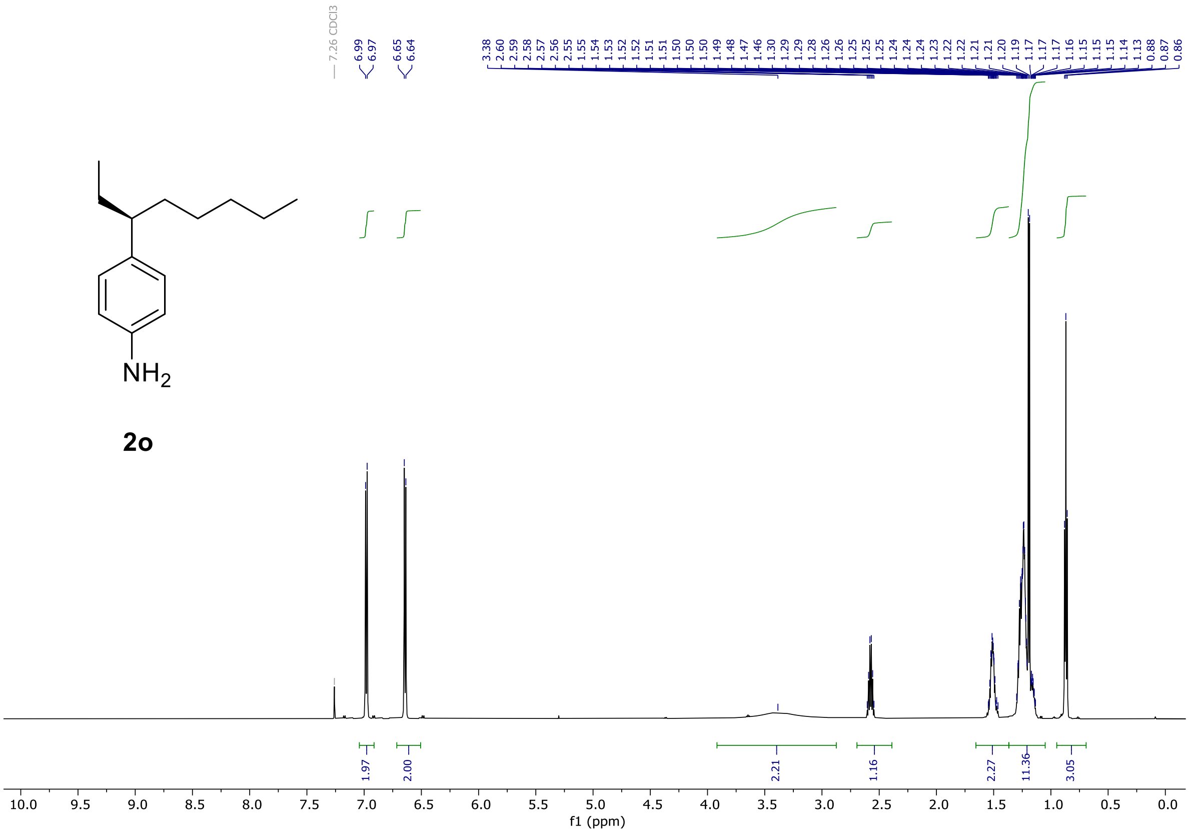

2o ^{13}C NMR (151 MHz, CDCl$_3$)

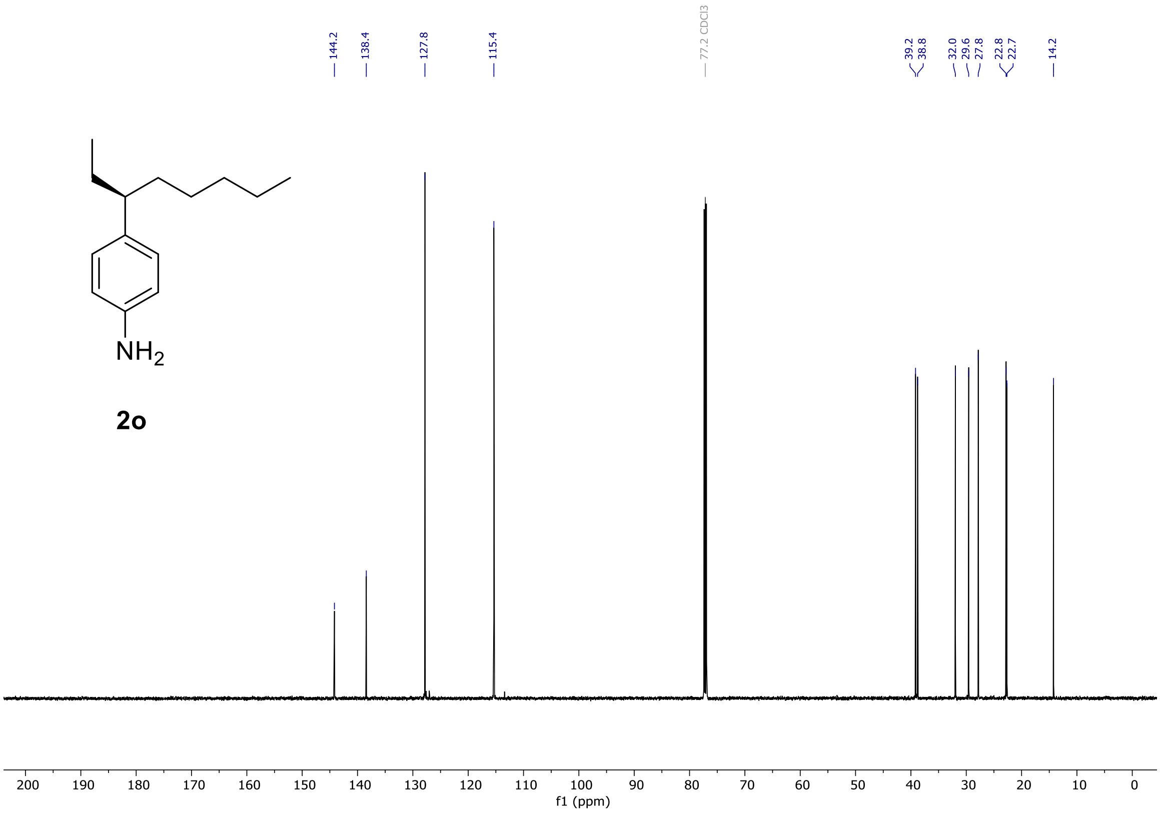

2p ^{1}H NMR (400 MHz, $CDCl_3$)

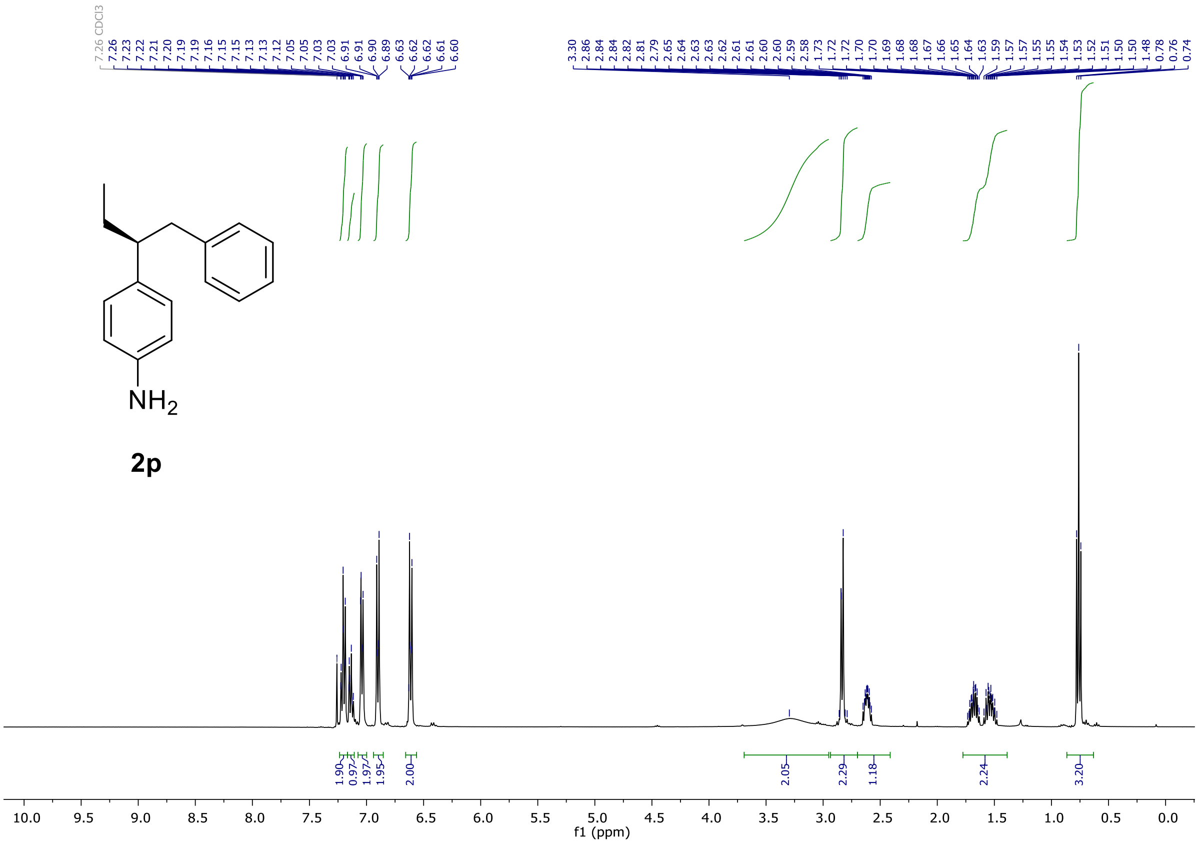

2p ^{13}C NMR (101 MHz, $CDCl_3$)

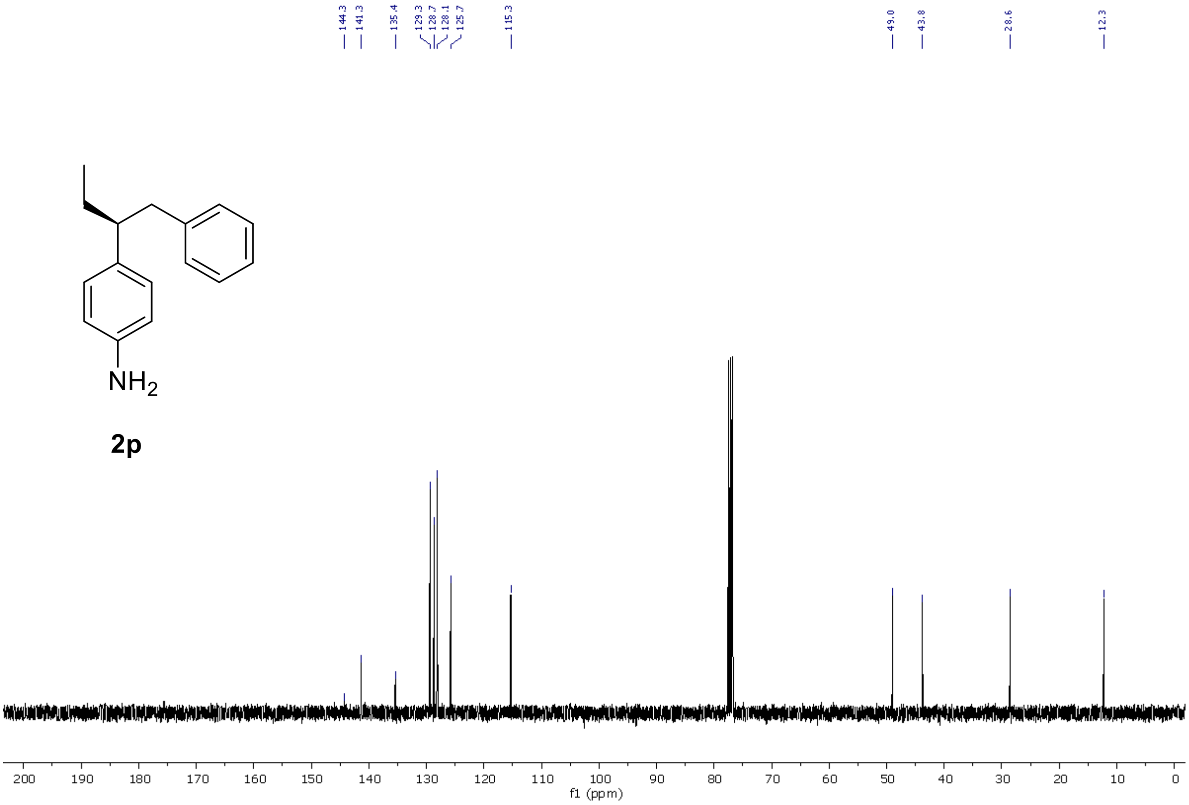

2q ^{1}H NMR (400 MHz, $CDCl_3$)

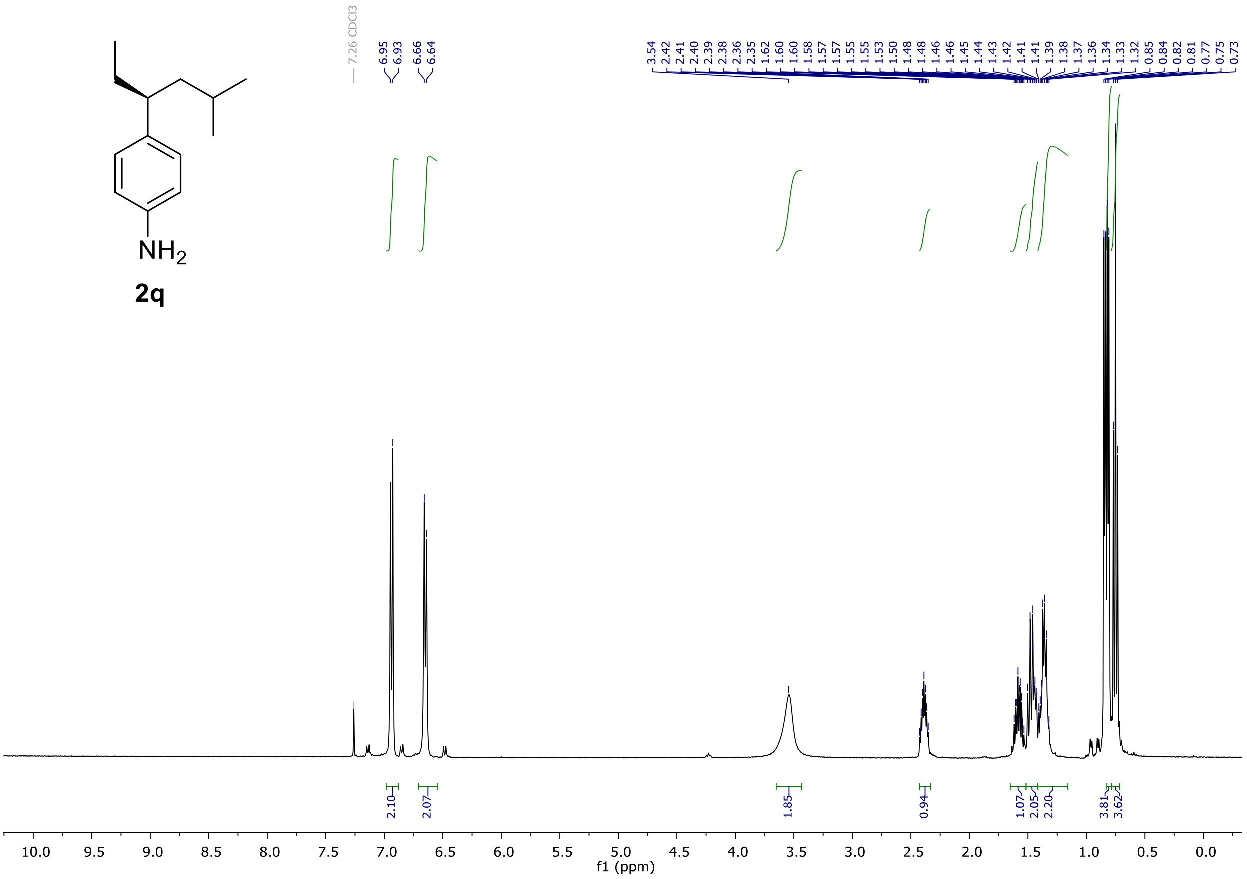

2q ^{13}C NMR (151 MHz, $CDCl_3$)

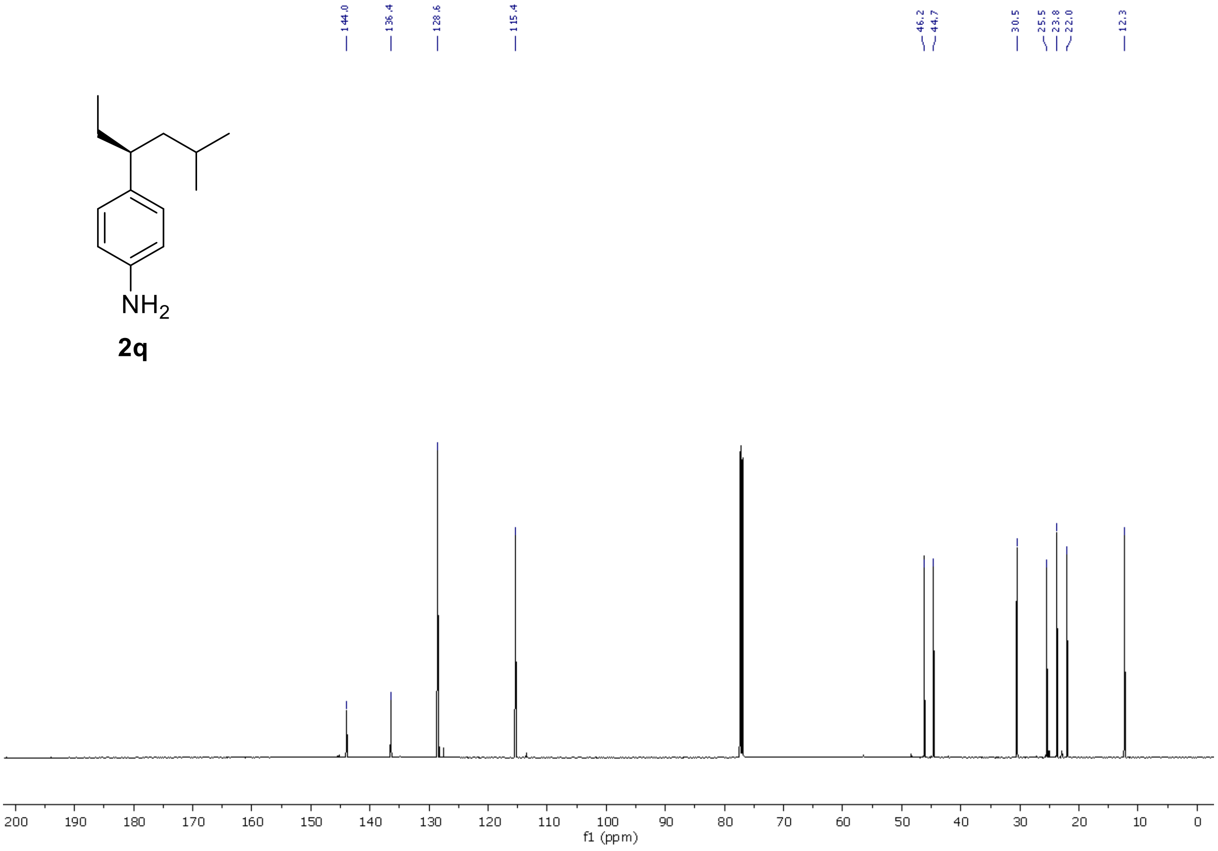

2r ^{1}H NMR (600 MHz, $CDCl_3$)

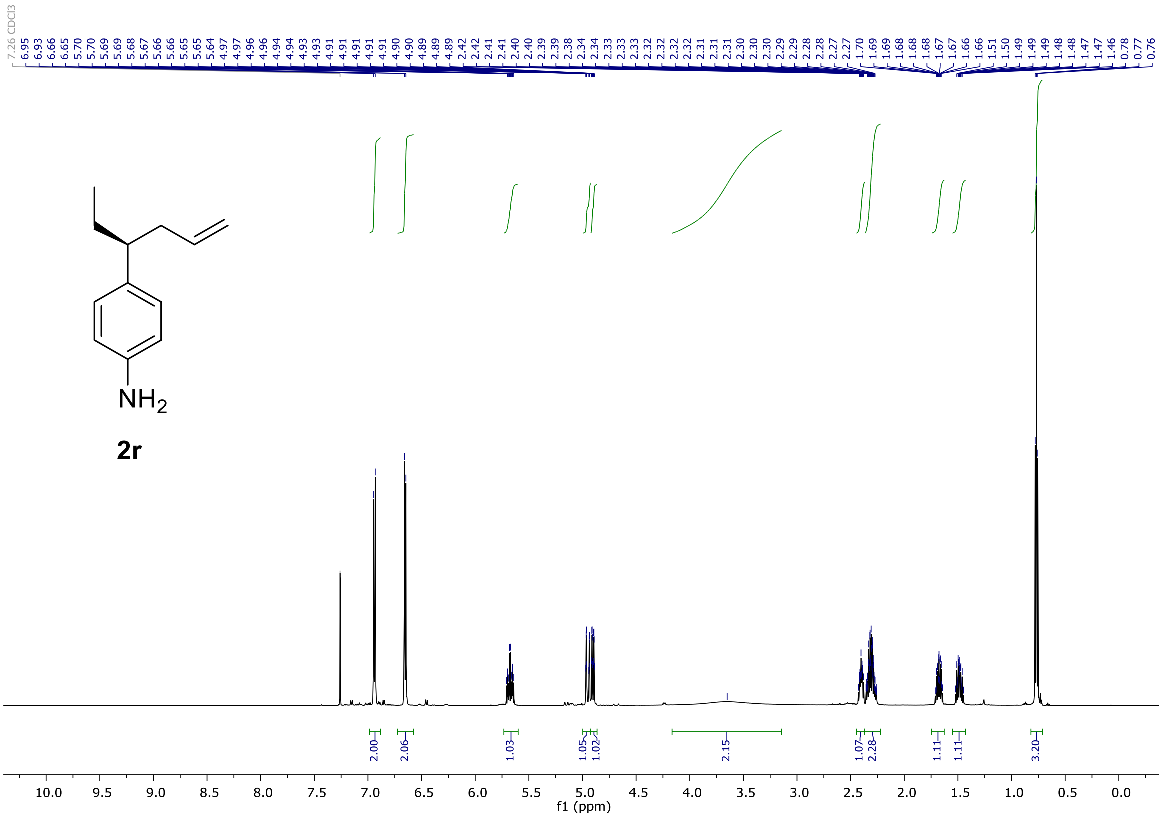

2r ^{13}C NMR (151 MHz, $CDCl_3$)

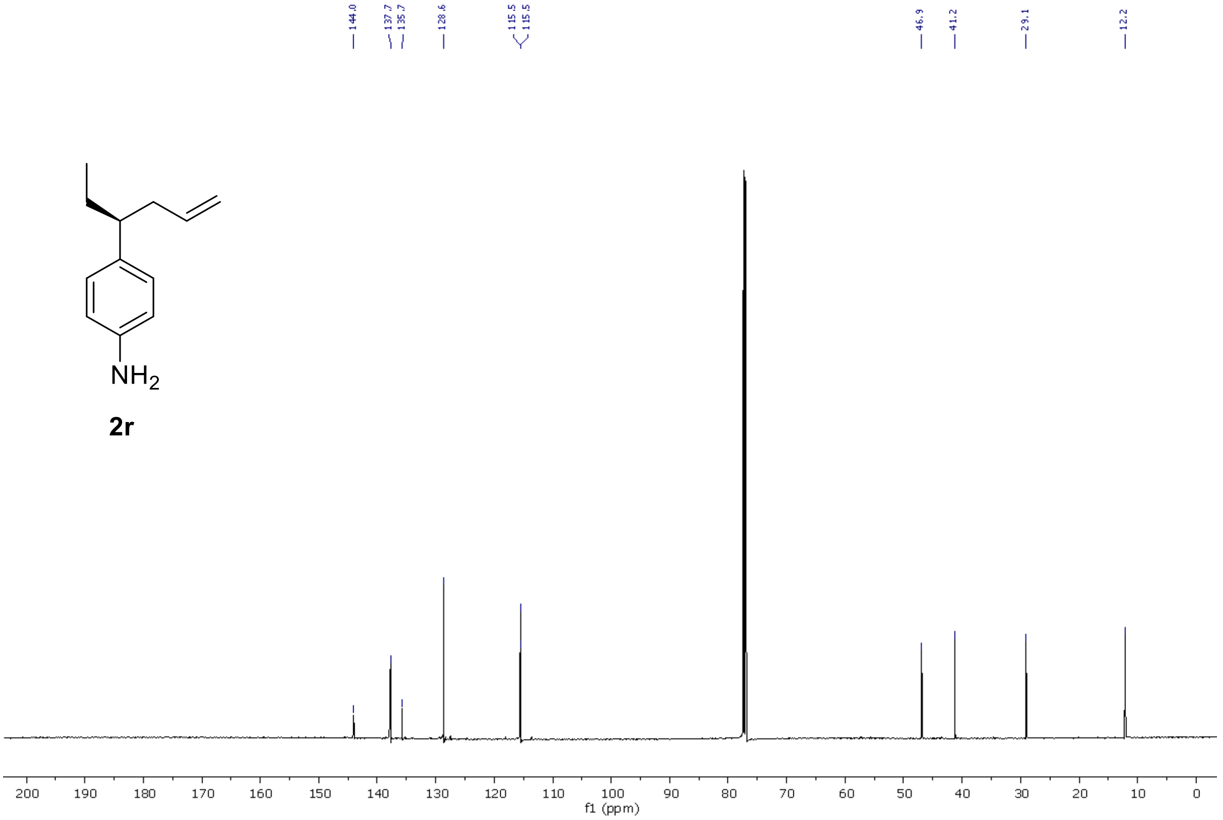

2s ^{1}H NMR (600 MHz, $CDCl_3$)

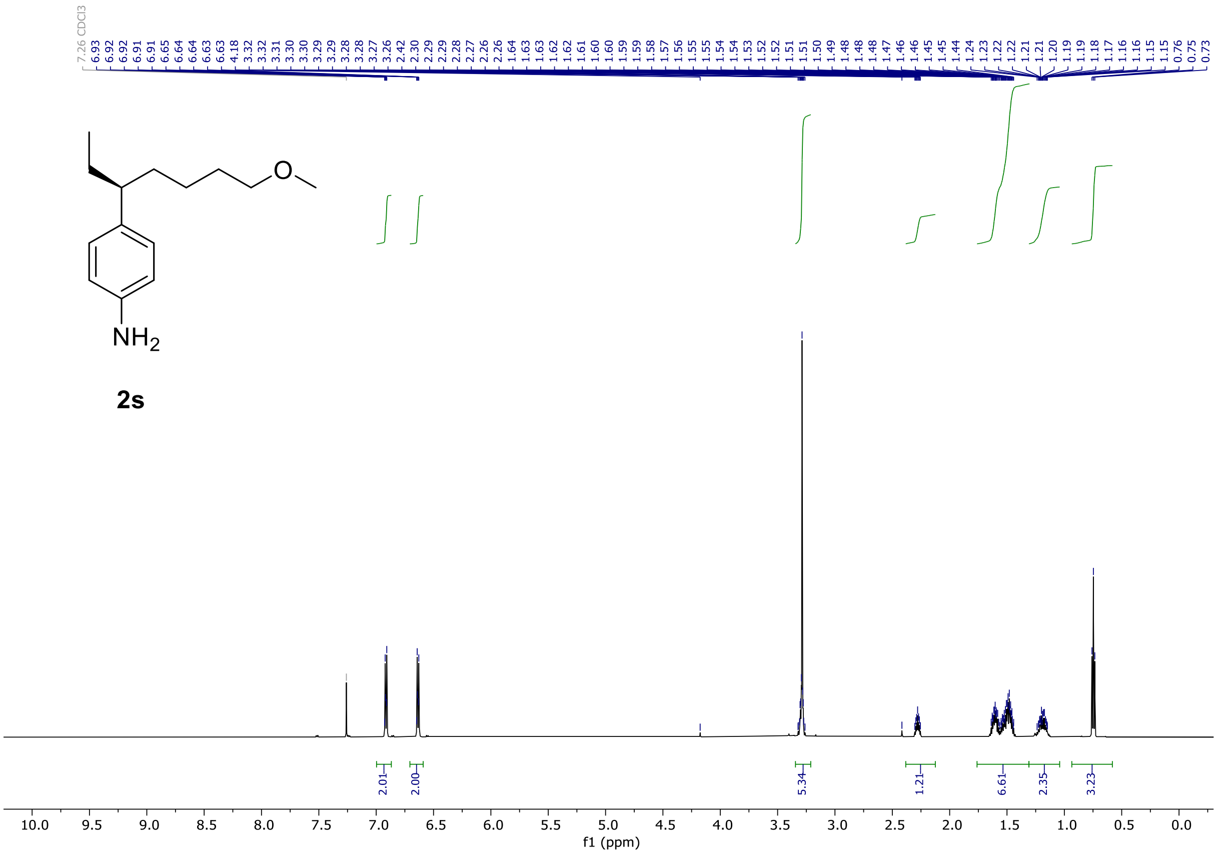

2s ^{13}C NMR (151 MHz, $CDCl_3$)

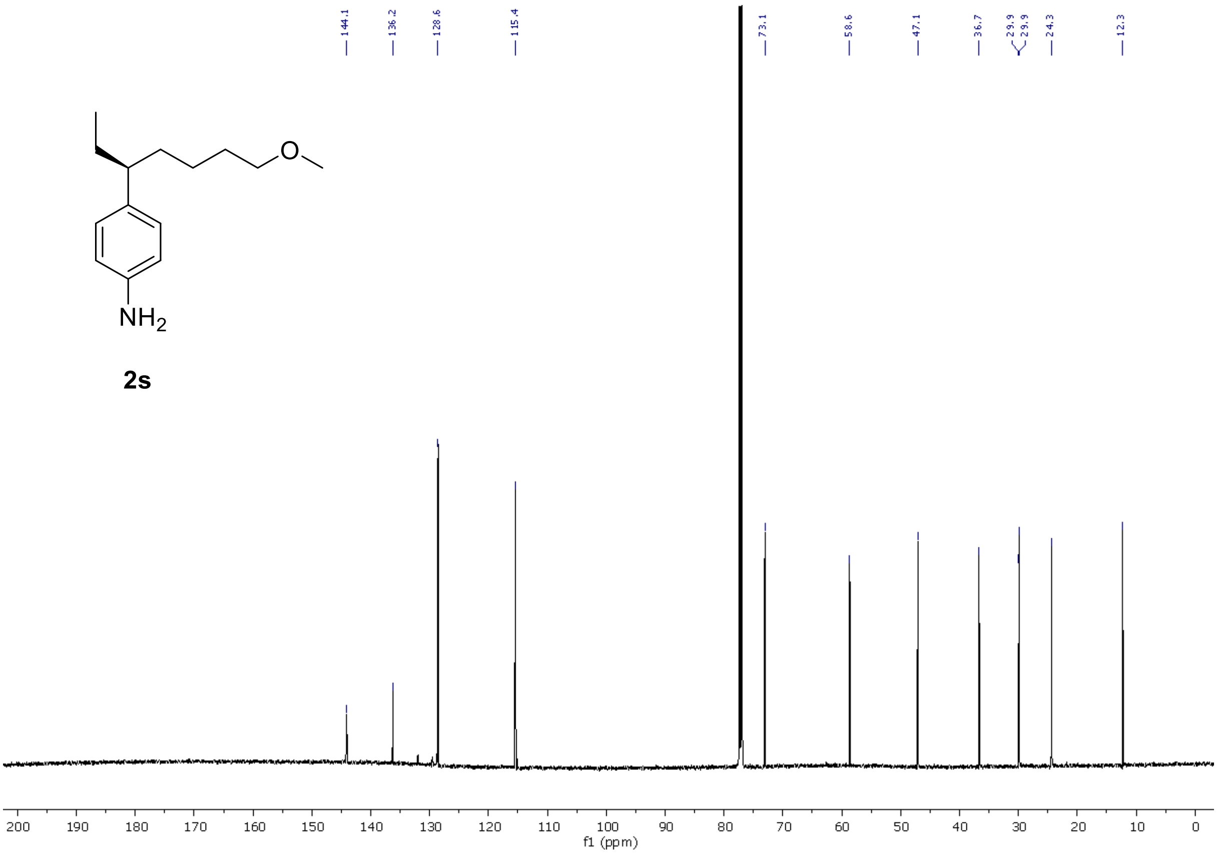

2t ^{1}H NMR (600 MHz, $CDCl_3$)

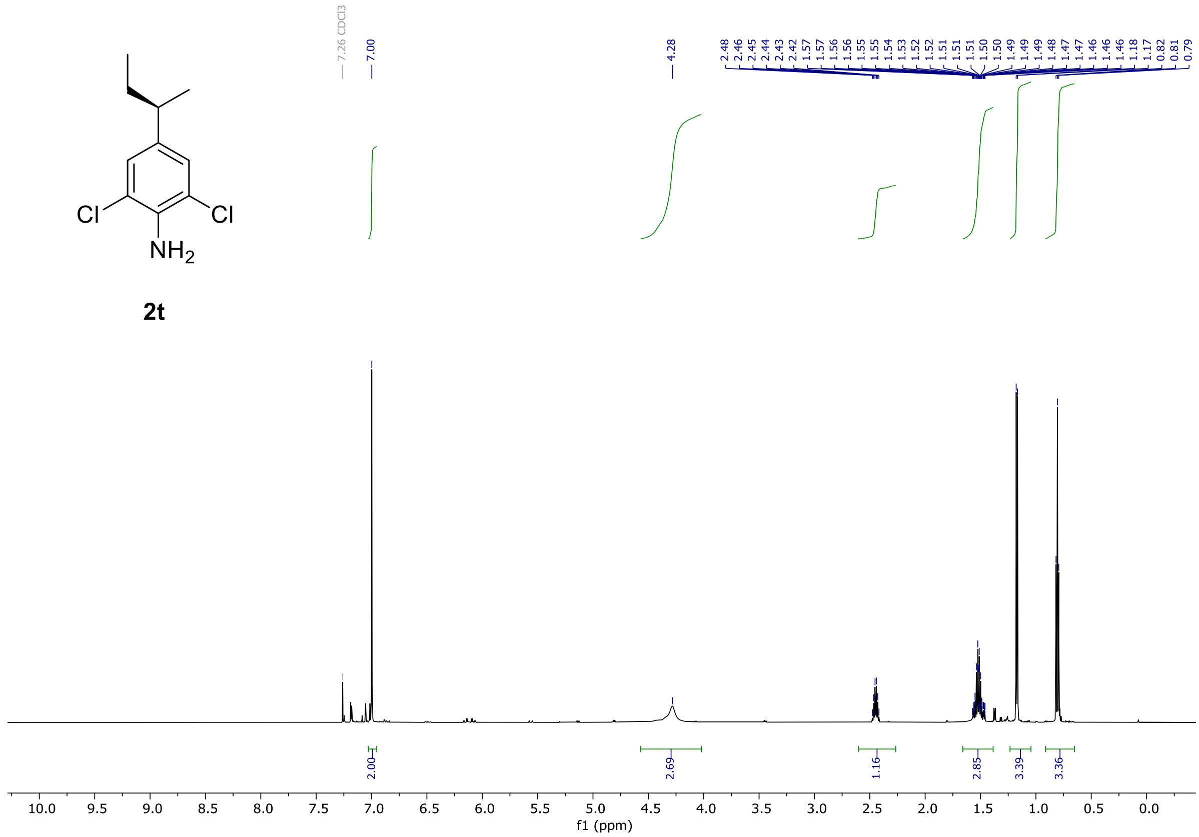

2t ^{13}C NMR (151 MHz, $CDCl_3$)

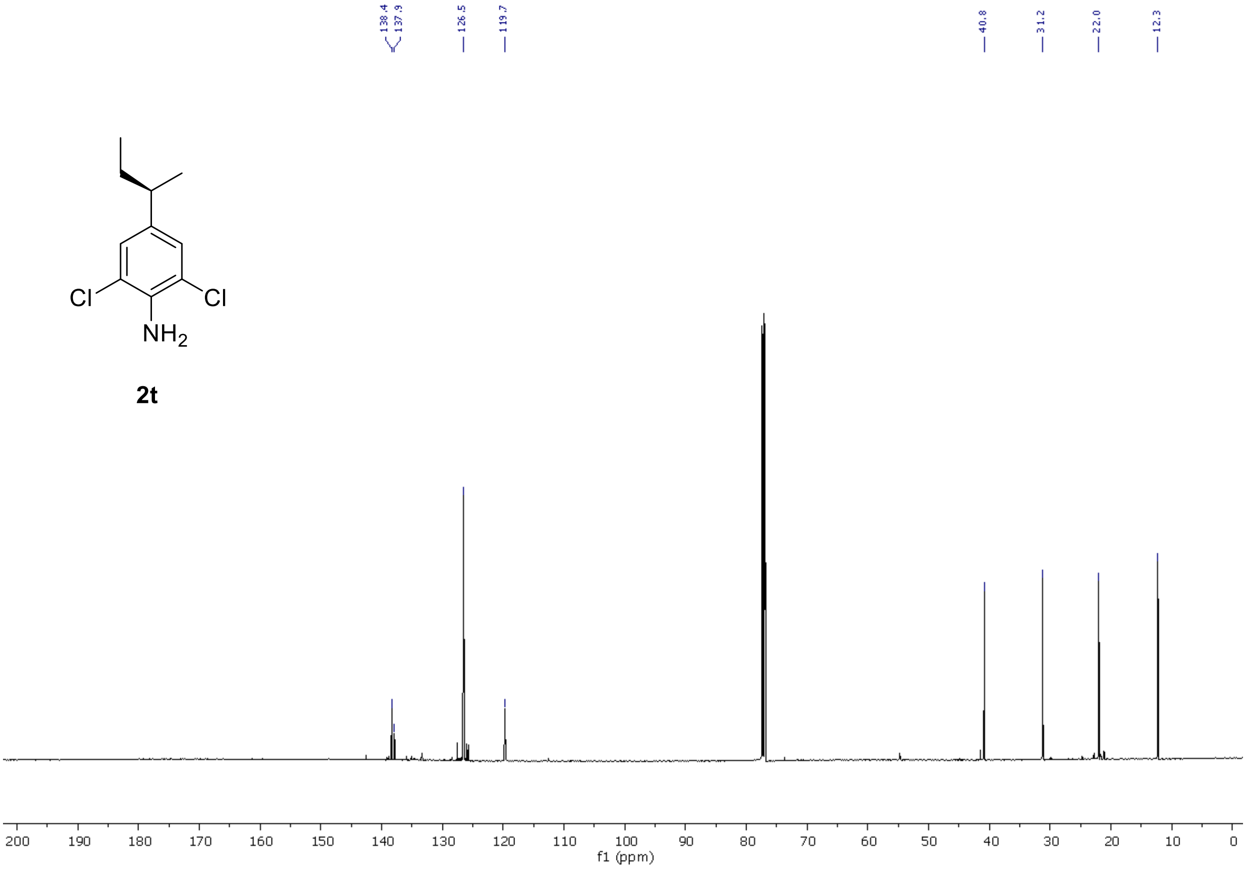

2d ^{1}H NMR (600 MHz, $CDCl_3$)

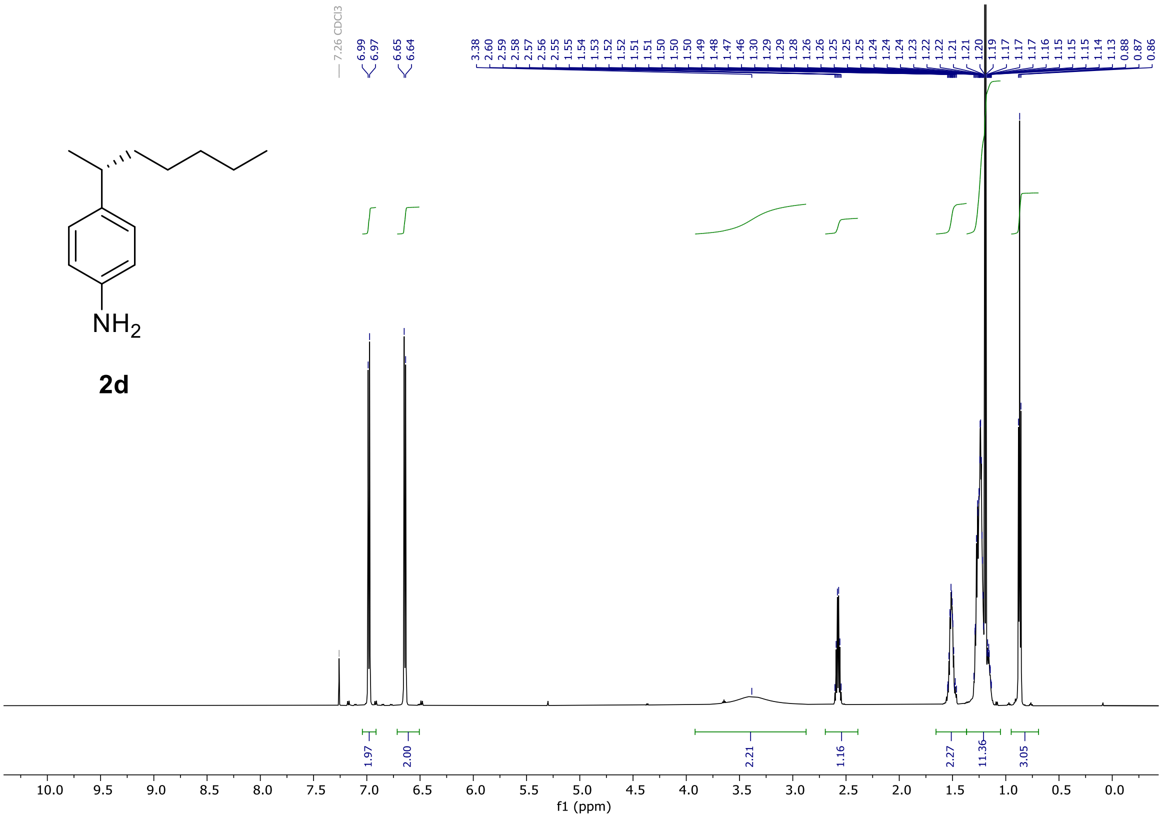

2d ^{13}C NMR (151 MHz, $CDCl_3$)

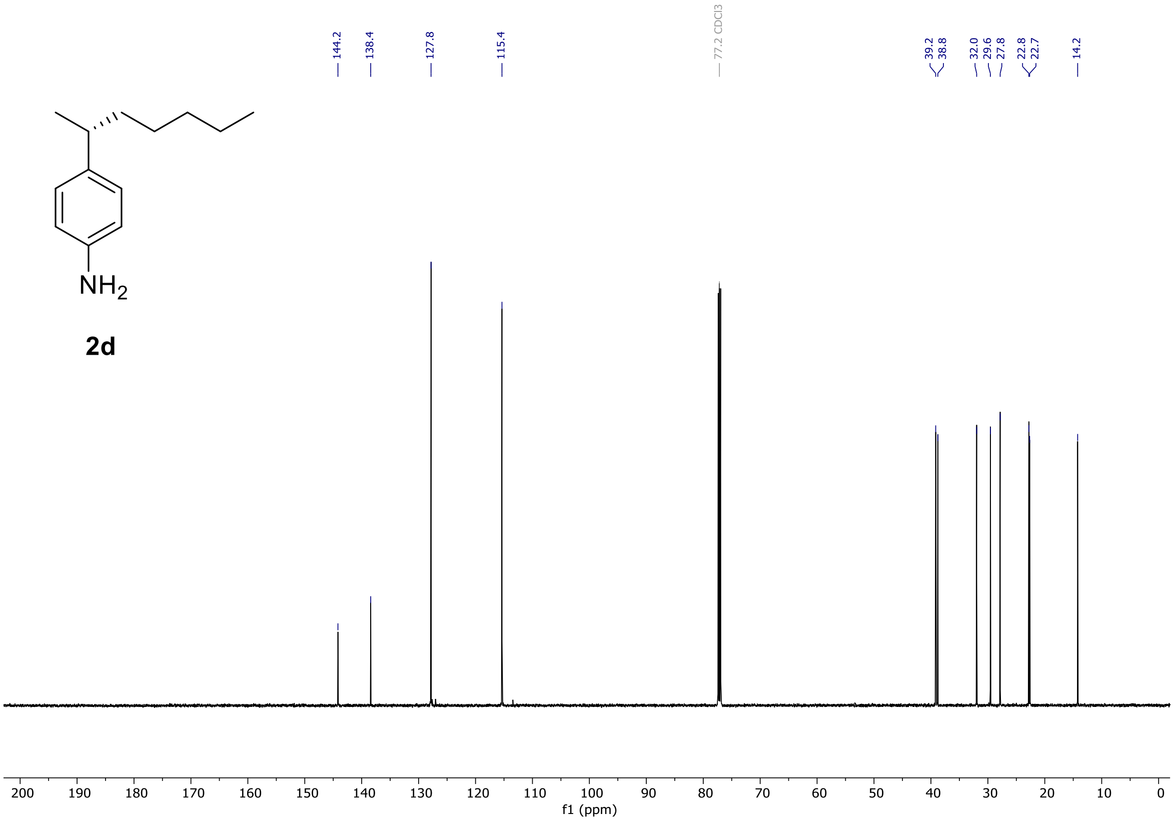

2e ^{1}H NMR (400 MHz, $CDCl_3$)

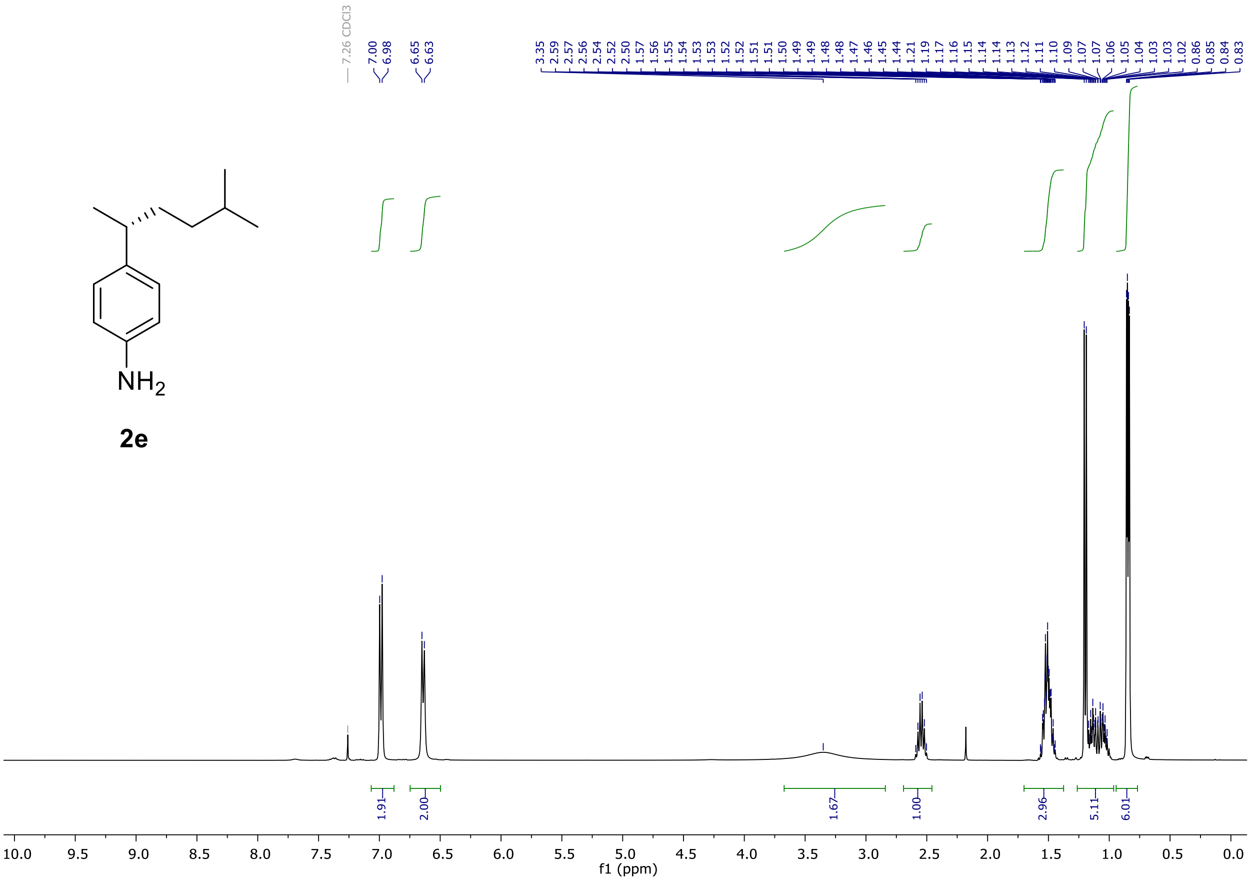

2e ^{13}C NMR (151 MHz, $CDCl_3$)

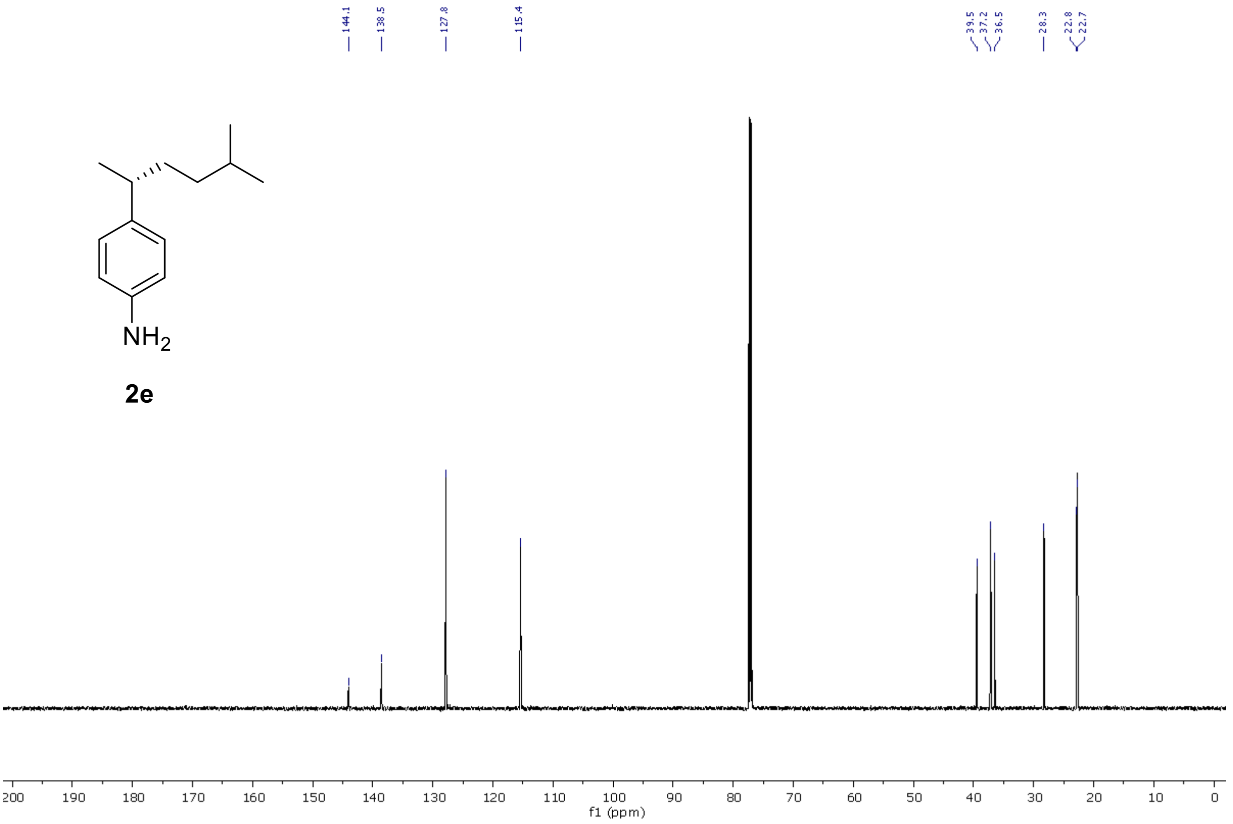

3 ^{1}H NMR (600 MHz, $CDCl_3$)

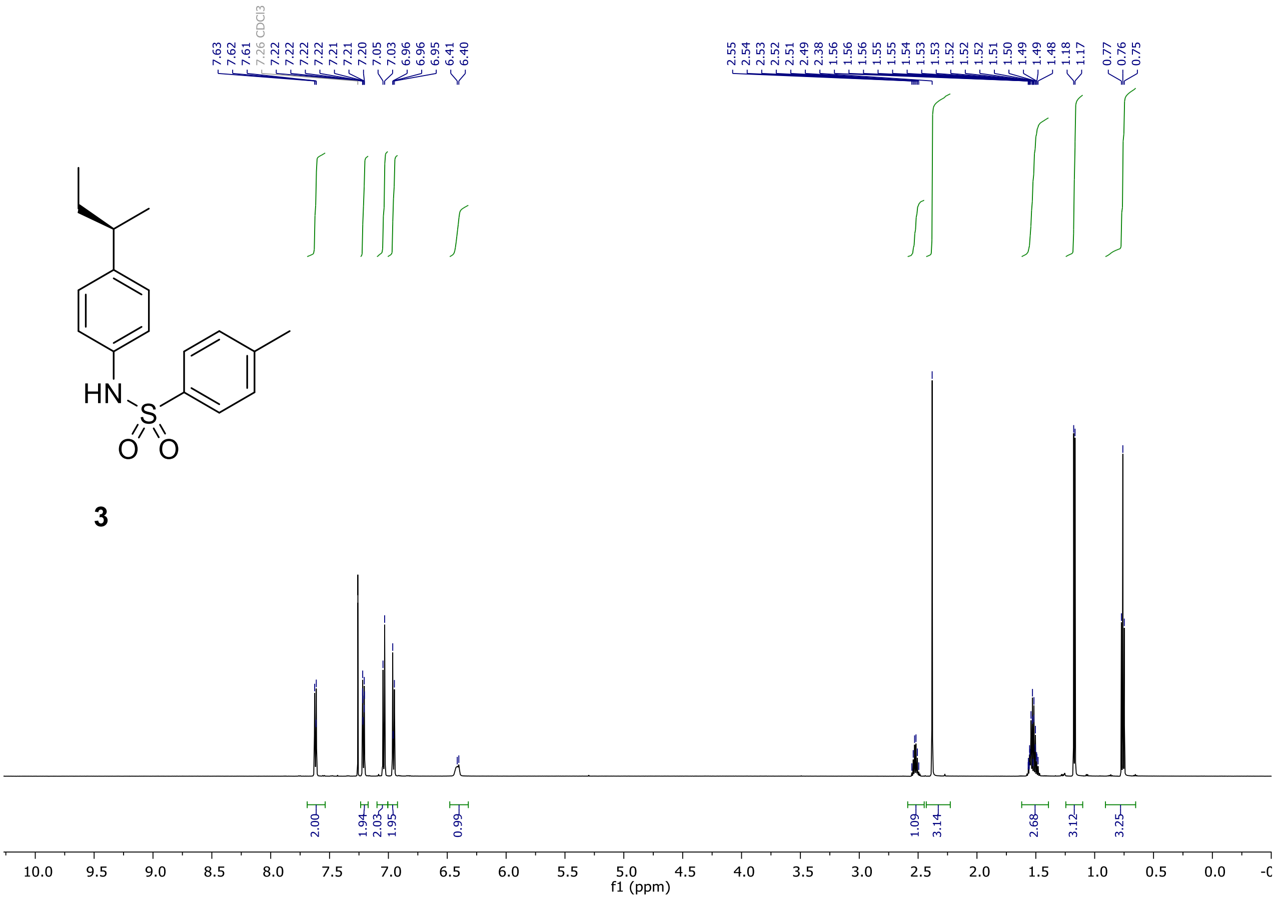

3 ^{13}C NMR (151 MHz, $CDCl_3$)

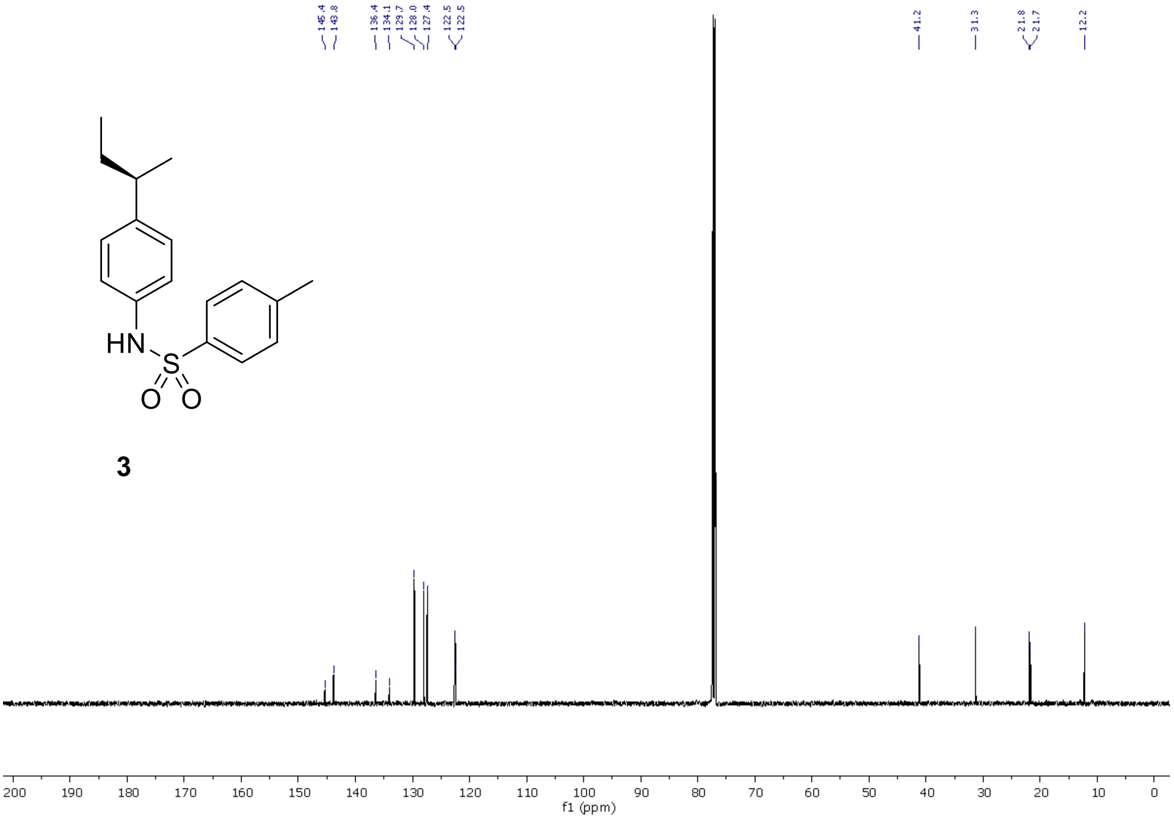

Supplement: Supplementary file 1 — ol2c02786_si_001.pdf [file ol2c02786_si_001.pdf]
